# Supplementary material for: Comparison of patient exit interviews with unannounced standardised patients for assessing HIV service delivery in Zambia: a study nested within a cluster randomised trial
Source: BMJ Open. 2023 Jul 5;13(7):e069086. doi: 10.1136/bmjopen-2022-069086 (PMC10335575; doi:10.1136/bmjopen-2022-069086)
Supplement: Supplementary data [file bmjopen-2022-069086supp003.pdf]

Supplementary Tables S1

Supplementary Table S1. Mixed effect Poisson regression comparing 10 questions for Trained Exit Clients vs Untrained Exit Clients. Adjusted for age, sex, education, and study period.

Trained Exit Clients	Prevalence ratio (PR) Unadjusted	P value	95% Confidence Interval (CI)	PR-Adjusted	P value	95% Confidence Interval (CI)	N
Sum score (Rate ratio)	1.73	<0.01	1.47-2.02	1.64	<0.01	1.39-1.94	3480
Did your HIV care provider greet you in a way that made you feel comfortable?	1.74	0.01	1.24-2.44	1.71	<0.01	1.20-2.44	3526
Did your HIV care provider listen to what you said?	1.77	0.09	0.91-3.45	1.71	0.09	0.93-3.16	3510
Did your HIV care provider give you as much information about your health as you wanted?	1.82	<0.01	1.43-2.33	1.72	<0.01	1.37-2.15	3526
Did your HIV care provider allow you to ask questions?	1.44	<0.01	1.20-1.73	1.34	<0.01	1.12-1.6	3517
Did your HIV care provider spend the right amount of time with you?	1.94	<0.01	1.66-2.27	1.85	<0.01	1.58-2.17	3520
Overall, how did you feel about the care you received today?	1.51	0.02	1.06-2.16	1.54	0.02	1.07-2.21	3515
Overall, were you satisfied with all your HIV care providers today?	2.12	<0.01	1.68-2.66	2.06	<0.01	1.61-2.63	3522
I witnessed HIV care providers behaving rudely during my visit today	2.39	<0.01	1.73-3.32	2.28	<0.01	1.63-3.19	3524
Were your lab results lost?	0.99	0.98	0.84-1.19	0.99	0.93	0.78-1.26	3522
Were you able to pick up your medicine today?	1.04	0.90	0.57-1.89	1.26	0.55	0.59-2.71	3525
