# Supplementary material for: Comparison of patient exit interviews with unannounced standardised patients for assessing HIV service delivery in Zambia: a study nested within a cluster randomised trial
Source: BMJ Open. 2023 Jul 5;13(7):e069086. doi: 10.1136/bmjopen-2022-069086 (PMC10335575; doi:10.1136/bmjopen-2022-069086)
Supplement: Supplementary data [file bmjopen-2022-069086supp001.pdf]

Supplementary Figure 1.

Impact of Training on Identifying Care Lapses Stratified by Subgroups for 10 questions. We observed some level of interaction for care status, age category, education category and facility size. Panel **a)** Greet you in a way that made you feel comfortable **b)** Listen to what you said **c)** Give you as much information about your health as you wanted **d)** Allowed you to ask questions, responded, happy q456 **e)** spend the right amount of time with you **f)** feel about the care you received today **g)** satisfied with all your HIV care providers today **h)** witnessed HIV care providers behaving rudely during my visit today **i)** lost lab results **j)** pick up meds

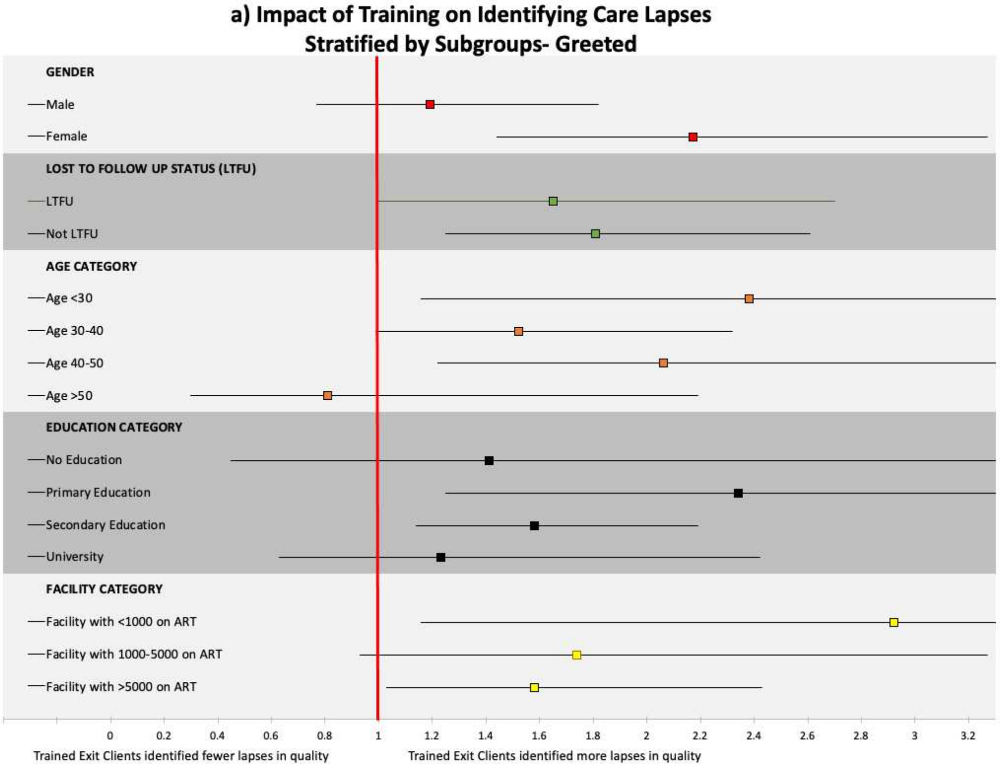

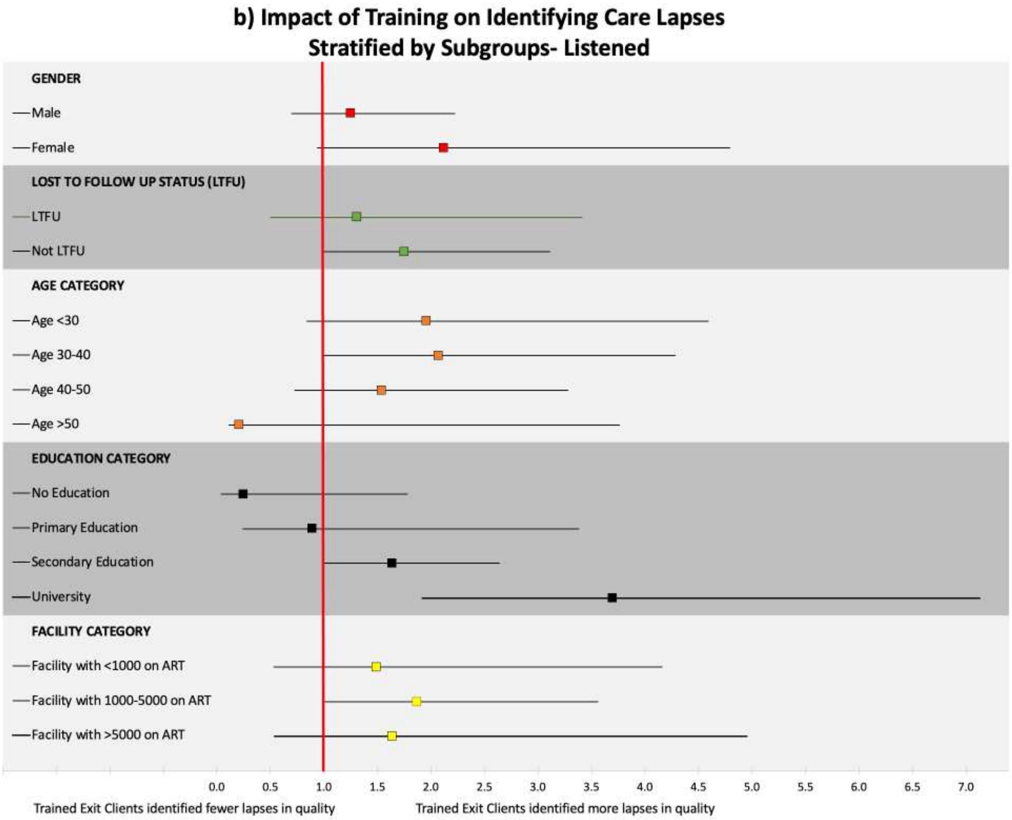
b) Impact of Training on Identifying Care Lapses
Stratified by Subgroups- Listened
GENDER
Male
Female
LOST TO FOLLOW UP STATUS (LTFU)
LTFU
Not LTFU
AGE CATEGORY
Age <30
Age 30-40
Age 40-50
Age >50
EDUCATION CATEGORY
No Education
Primary Education
Secondary Education
University
FACILITY CATEGORY
Facility with <1000 on ART
Facility with 1000-5000 on ART
Facility with >5000 on ART
0.0 0.5 1.0 1.5 2.0 2.5 3.0 3.5 4.0 4.5 5.0 5.5 6.0 6.5 7.0
Trained Exit Clients identified fewer lapses in quality
Trained Exit Clients identified more lapses in quality

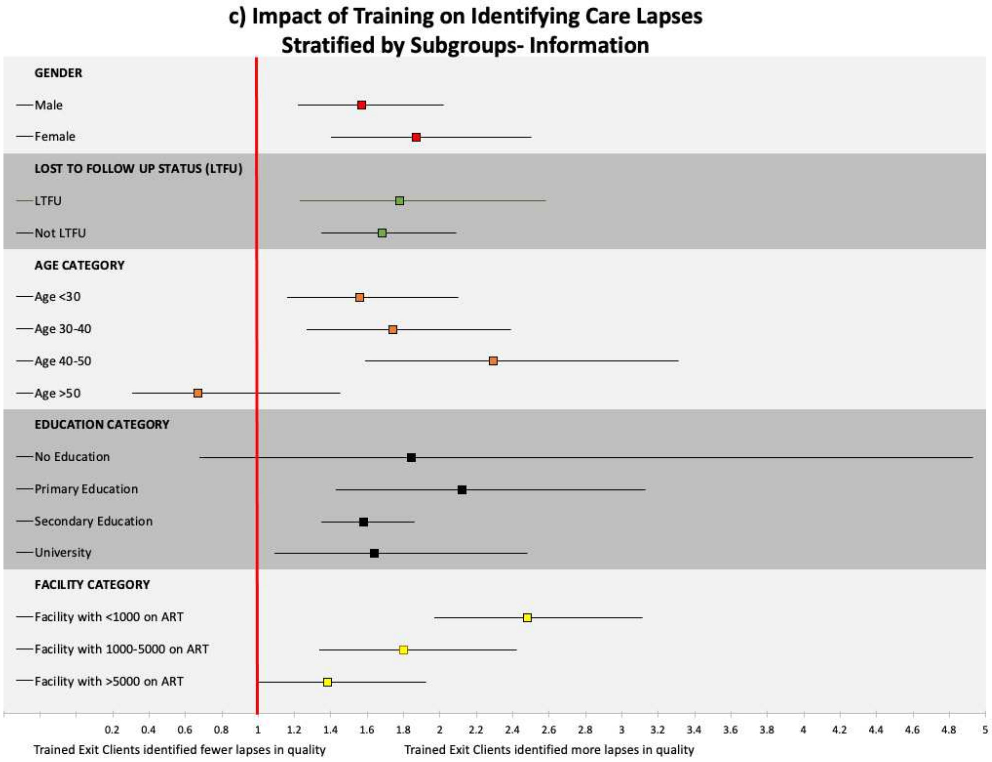
c) Impact of Training on Identifying Care Lapses
Stratified by Subgroups- Information
GENDER
Male
Female
LOST TO FOLLOW UP STATUS (LTFU)
LTFU
Not LTFU
AGE CATEGORY
Age <30
Age 30-40
Age 40-50
Age >50
EDUCATION CATEGORY
No Education
Primary Education
Secondary Education
University
FACILITY CATEGORY
Facility with <1000 on ART
Facility with 1000-5000 on ART
Facility with >5000 on ART
0.2 0.4 0.6 0.8 1 1.2 1.4 1.6 1.8 2 2.2 2.4 2.6 2.8 3 3.2 3.4 3.6 3.8 4 4.2 4.4 4.6 4.8 5
Trained Exit Clients identified fewer lapses in quality
Trained Exit Clients identified more lapses in quality

d) Impact of Training on Identifying Care Lapses Stratified by Subgroups- Question_456

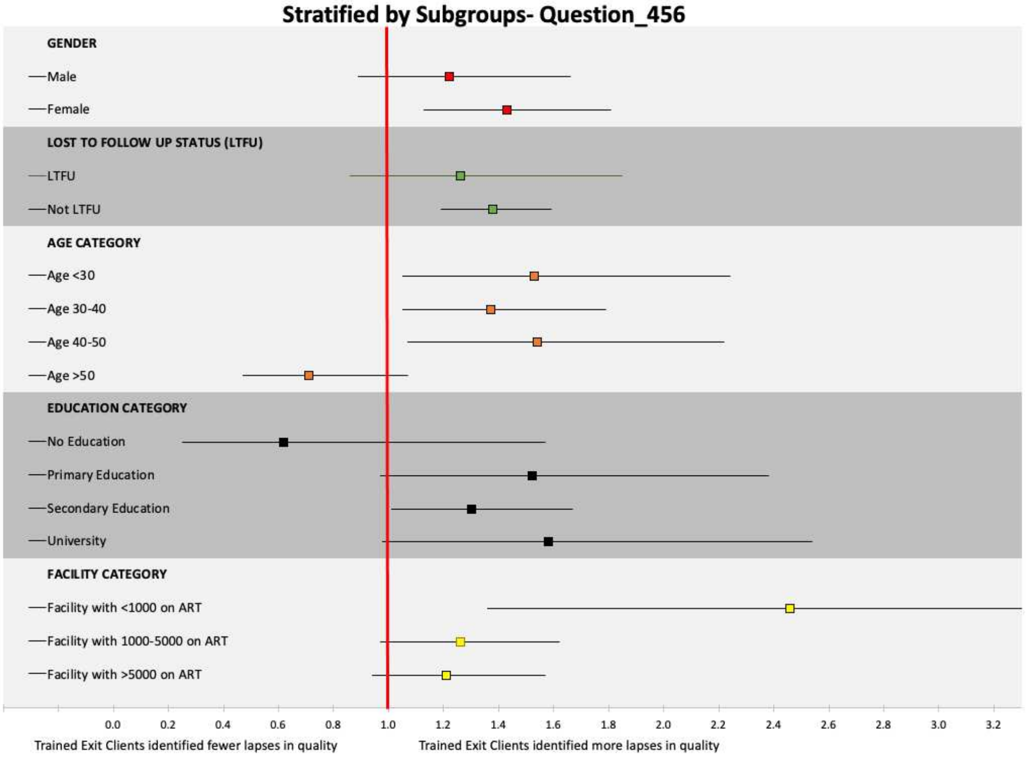

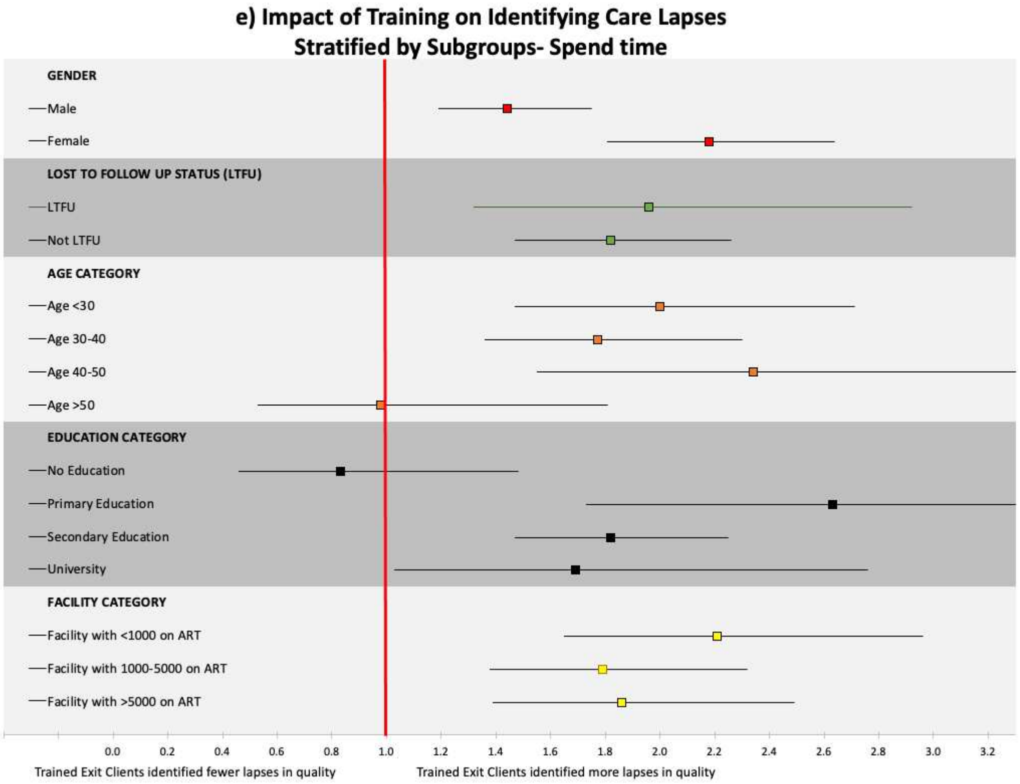
e) Impact of Training on Identifying Care Lapses
Stratified by Subgroups- Spend time
GENDER
Male
Female
LOST TO FOLLOW UP STATUS (LTFU)
LTFU
Not LTFU
AGE CATEGORY
Age <30
Age 30-40
Age 40-50
Age >50
EDUCATION CATEGORY
No Education
Primary Education
Secondary Education
University
FACILITY CATEGORY
Facility with <1000 on ART
Facility with 1000-5000 on ART
Facility with >5000 on ART
0.0 0.2 0.4 0.6 0.8 1.0 1.2 1.4 1.6 1.8 2.0 2.2 2.4 2.6 2.8 3.0 3.2
Trained Exit Clients identified fewer lapses in quality
Trained Exit Clients identified more lapses in quality

f) Impact of Training on Identifying Care Lapses Stratified by Subgroups- Feel about care

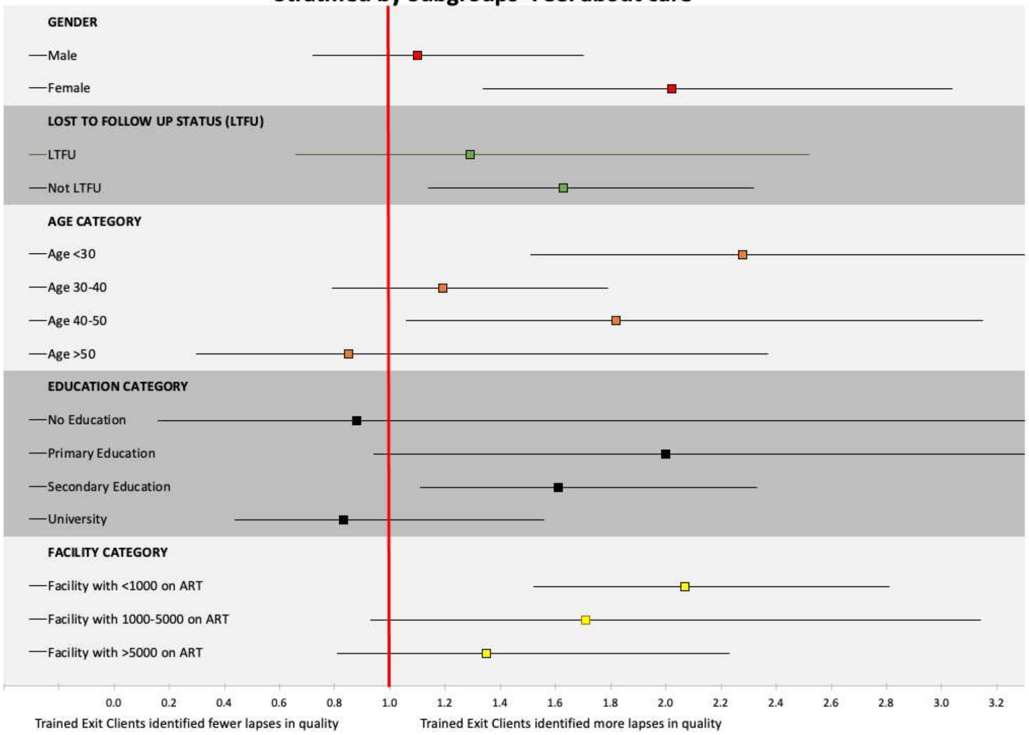

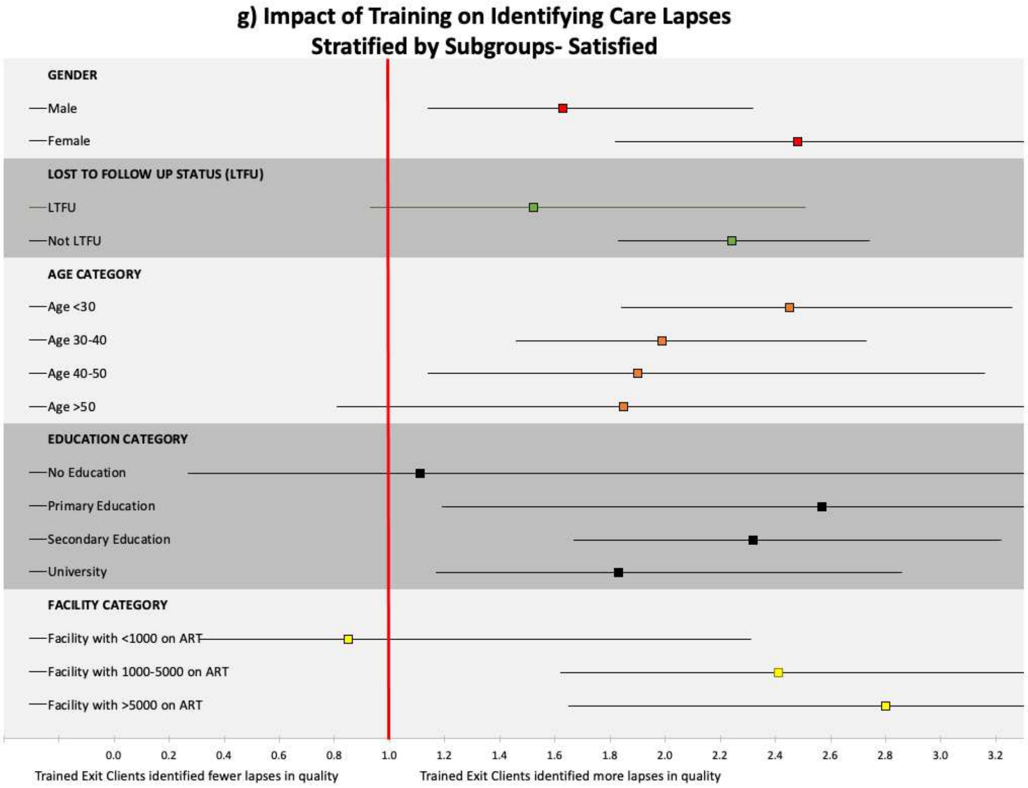
g) Impact of Training on Identifying Care Lapses
Stratified by Subgroups- Satisfied
GENDER
Male
Female
LOST TO FOLLOW UP STATUS (LTFU)
LTFU
Not LTFU
AGE CATEGORY
Age <30
Age 30-40
Age 40-50
Age >50
EDUCATION CATEGORY
No Education
Primary Education
Secondary Education
University
FACILITY CATEGORY
Facility with <1000 on ART
Facility with 1000-5000 on ART
Facility with >5000 on ART
0.0 0.2 0.4 0.6 0.8 1.0 1.2 1.4 1.6 1.8 2.0 2.2 2.4 2.6 2.8 3.0 3.2
Trained Exit Clients identified fewer lapses in quality
Trained Exit Clients identified more lapses in quality

h) Impact of Training on Identifying Care Lapses Stratified by Subgroups- Rude

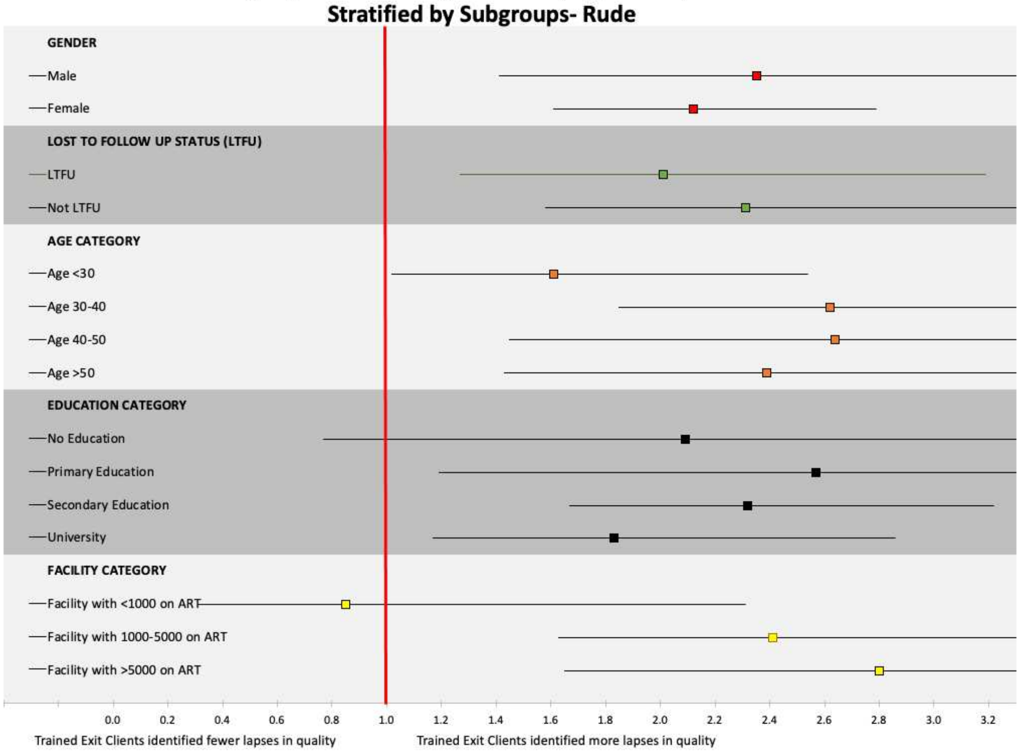

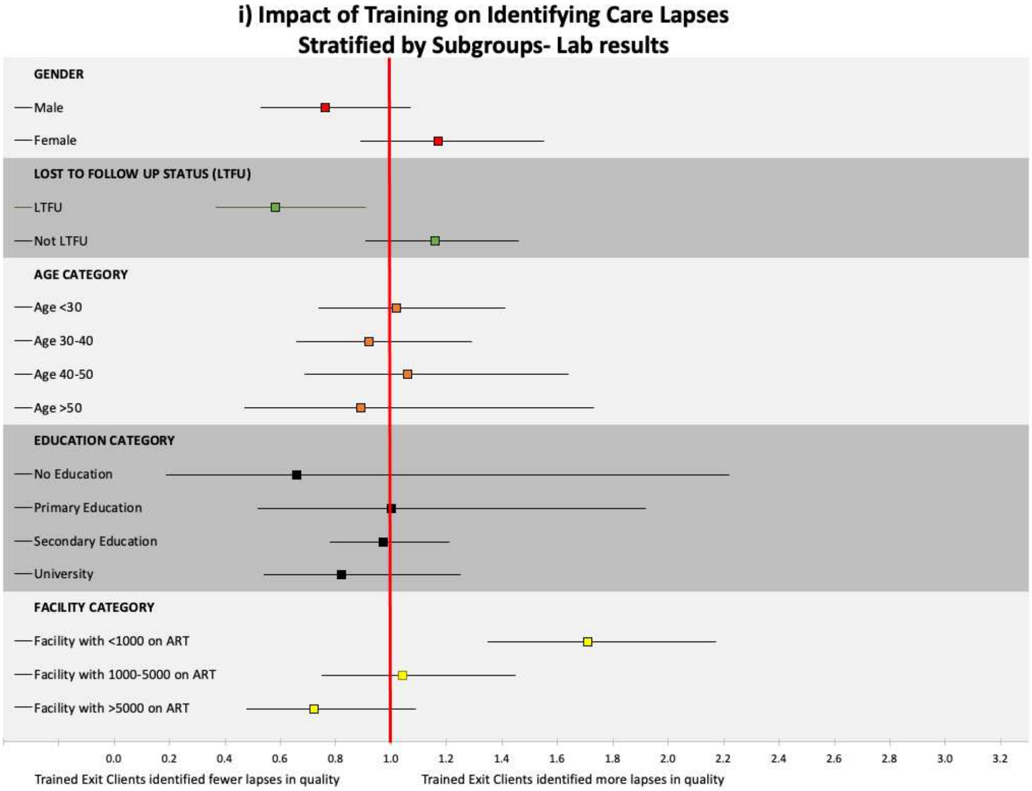
i) Impact of Training on Identifying Care Lapses
Stratified by Subgroups- Lab results
GENDER
Male
Female
LOST TO FOLLOW UP STATUS (LTFU)
LTFU
Not LTFU
AGE CATEGORY
Age <30
Age 30-40
Age 40-50
Age >50
EDUCATION CATEGORY
No Education
Primary Education
Secondary Education
University
FACILITY CATEGORY
Facility with <1000 on ART
Facility with 1000-5000 on ART
Facility with >5000 on ART
0.0
0.2
0.4
0.6
0.8
1.0
1.2
1.4
1.6
1.8
2.0
2.2
2.4
2.6
2.8
3.0
3.2
Trained Exit Clients identified fewer lapses in quality
Trained Exit Clients identified more lapses in quality

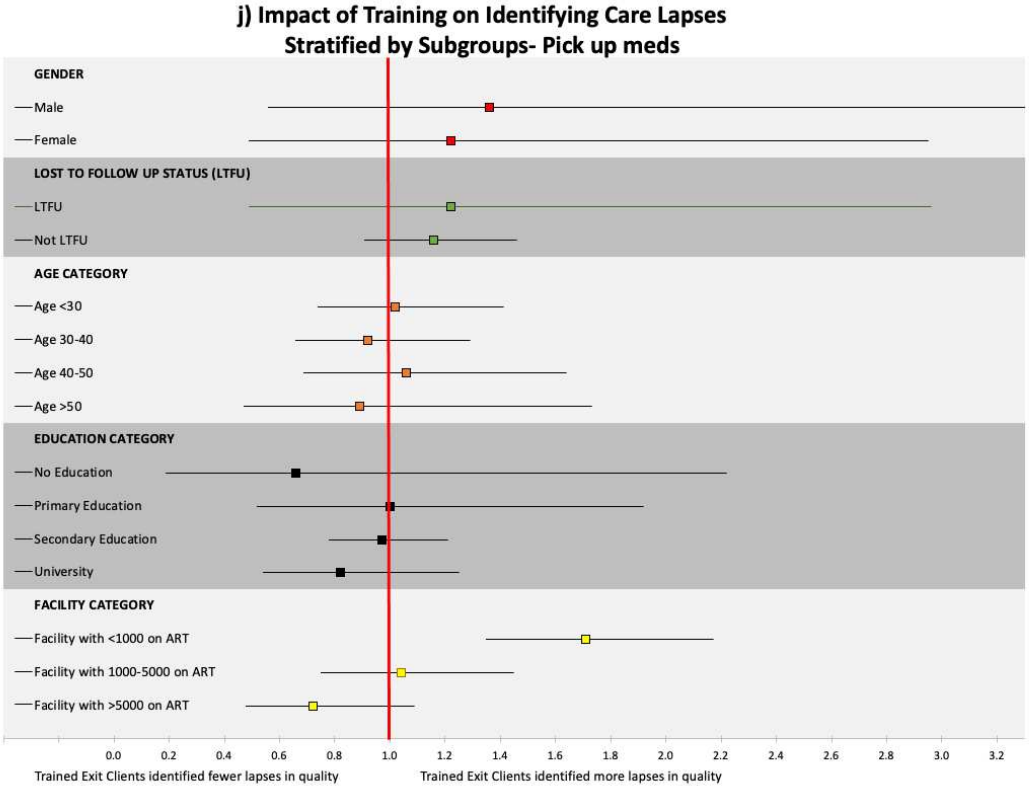
j) Impact of Training on Identifying Care Lapses
Stratified by Subgroups- Pick up meds
GENDER
Male
Female
LOST TO FOLLOW UP STATUS (LTFU)
LTFU
Not LTFU
AGE CATEGORY
Age <30
Age 30-40
Age 40-50
Age >50
EDUCATION CATEGORY
No Education
Primary Education
Secondary Education
University
FACILITY CATEGORY
Facility with <1000 on ART
Facility with 1000-5000 on ART
Facility with >5000 on ART
0.0
0.2
0.4
0.6
0.8
1.0
1.2
1.4
1.6
1.8
2.0
2.2
2.4
2.6
2.8
3.0
3.2
Trained Exit Clients identified fewer lapses in quality
Trained Exit Clients identified more lapses in quality
