# Supplementary material for: Comparison of patient exit interviews with unannounced standardised patients for assessing HIV service delivery in Zambia: a study nested within a cluster randomised trial
Source: BMJ Open. 2023 Jul 5;13(7):e069086. doi: 10.1136/bmjopen-2022-069086 (PMC10335575; doi:10.1136/bmjopen-2022-069086)
Supplement: Supplementary data [file bmjopen-2022-069086supp002.pdf]

Supplementary Figure 2

Supplementary Figure 2. Bubble plot showing Trained Exit Sum Score vs Untrained Exit Sum Score. Each bubble represents a single facilities performance. Each bubble's size indicates the number of patients at each facility with larger bubbles corresponding to larger facilities. The horizontal position notes the Untrained Exit Sum Score for all questions against the facility, and the vertical position notes the Trained Exit sum score at the same facility.

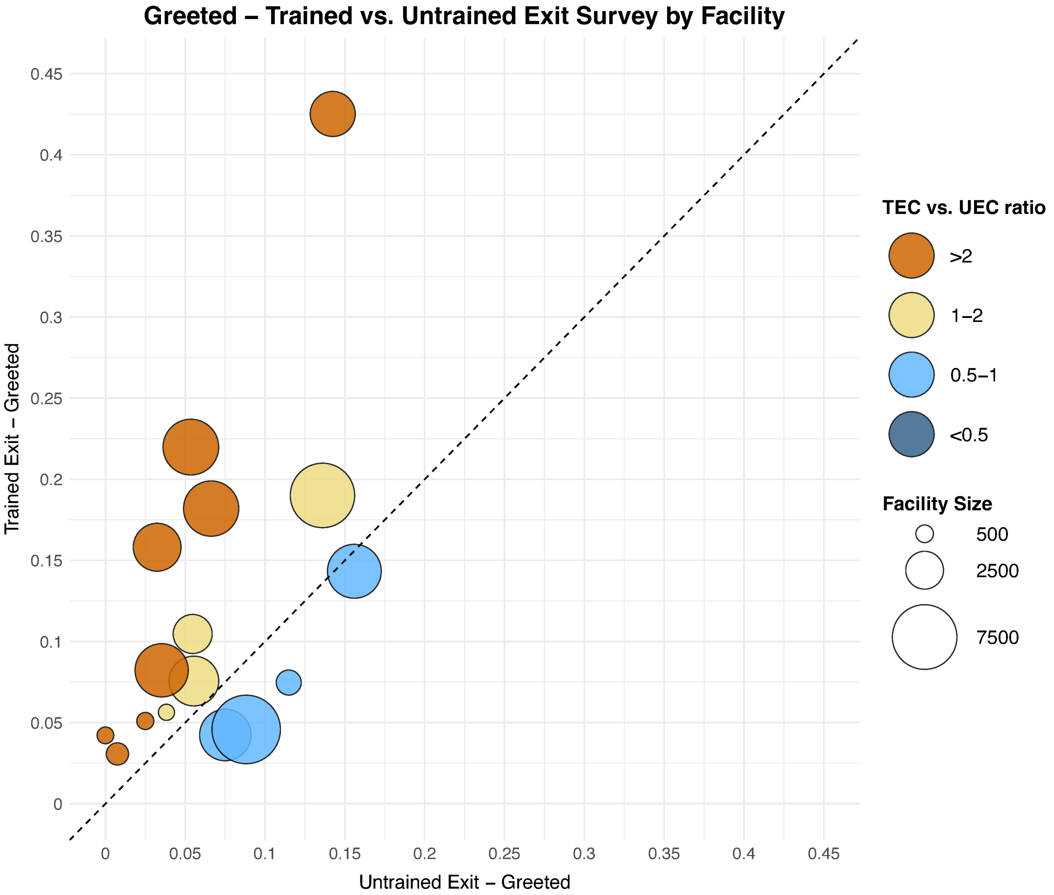

Sikombe K, *et al. BMJ Open* 2023; 13:e069086. doi: 10.1136/bmjopen-2022-069086

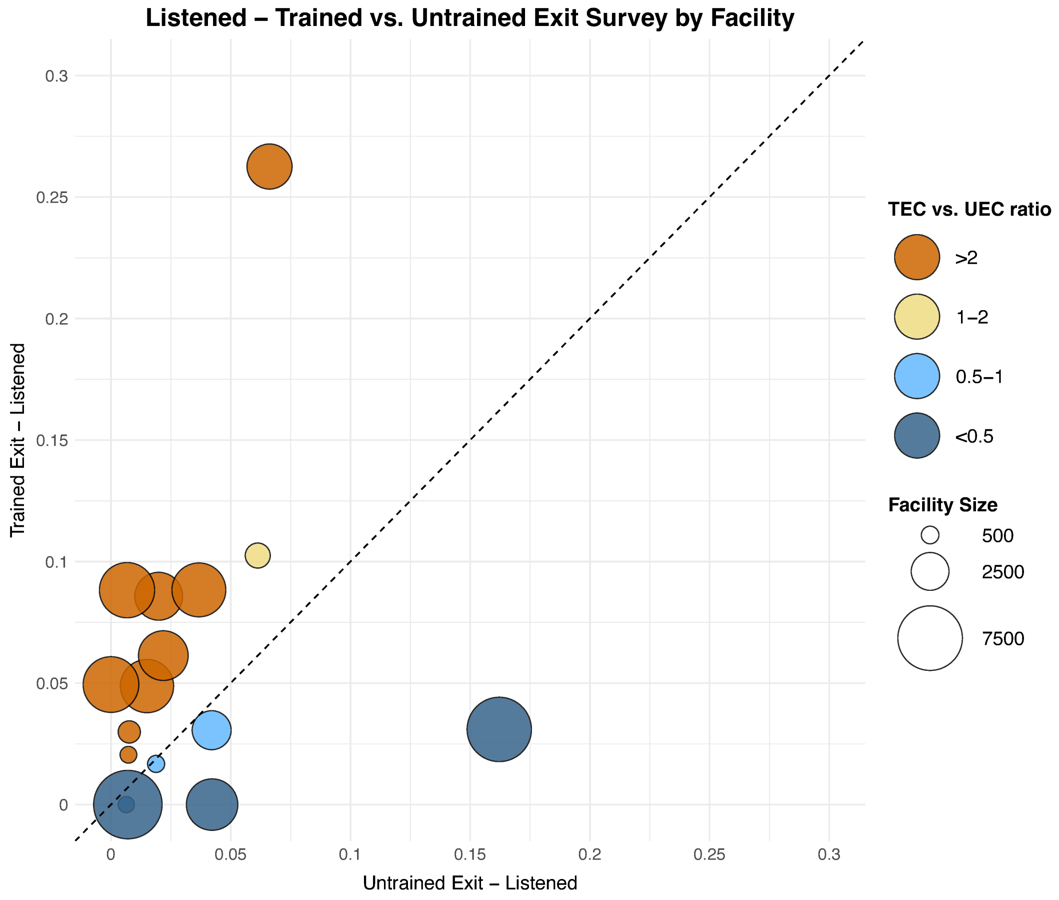

Sikombe K, *et al. BMJ Open* 2023; 13:e069086. doi: 10.1136/bmjopen-2022-069086

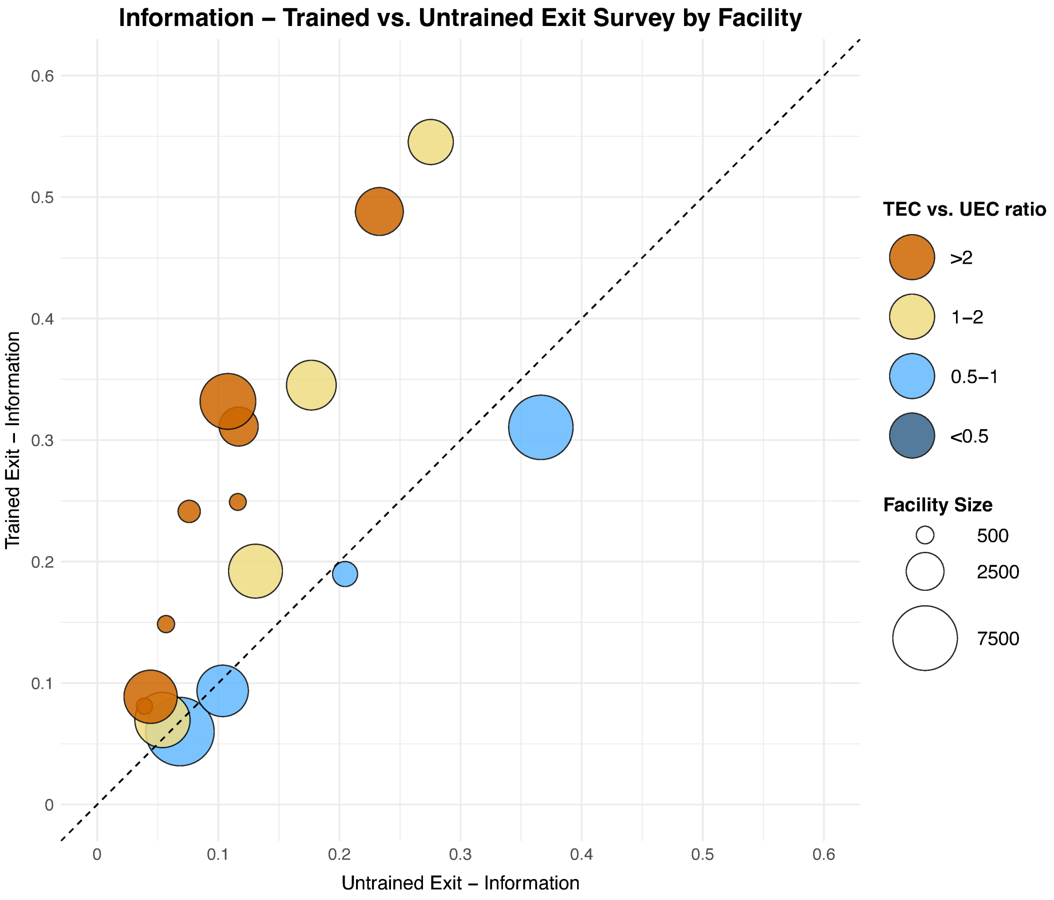
Information – Trained vs. Untrained Exit Survey by Facility
Trained Exit – Information
Untrained Exit – Information
0
0.1
0.2
0.3
0.4
0.5
0.6
TEC vs. UEC ratio
>2
1–2
0.5–1
<0.5
Facility Size
500
2500
7500

3

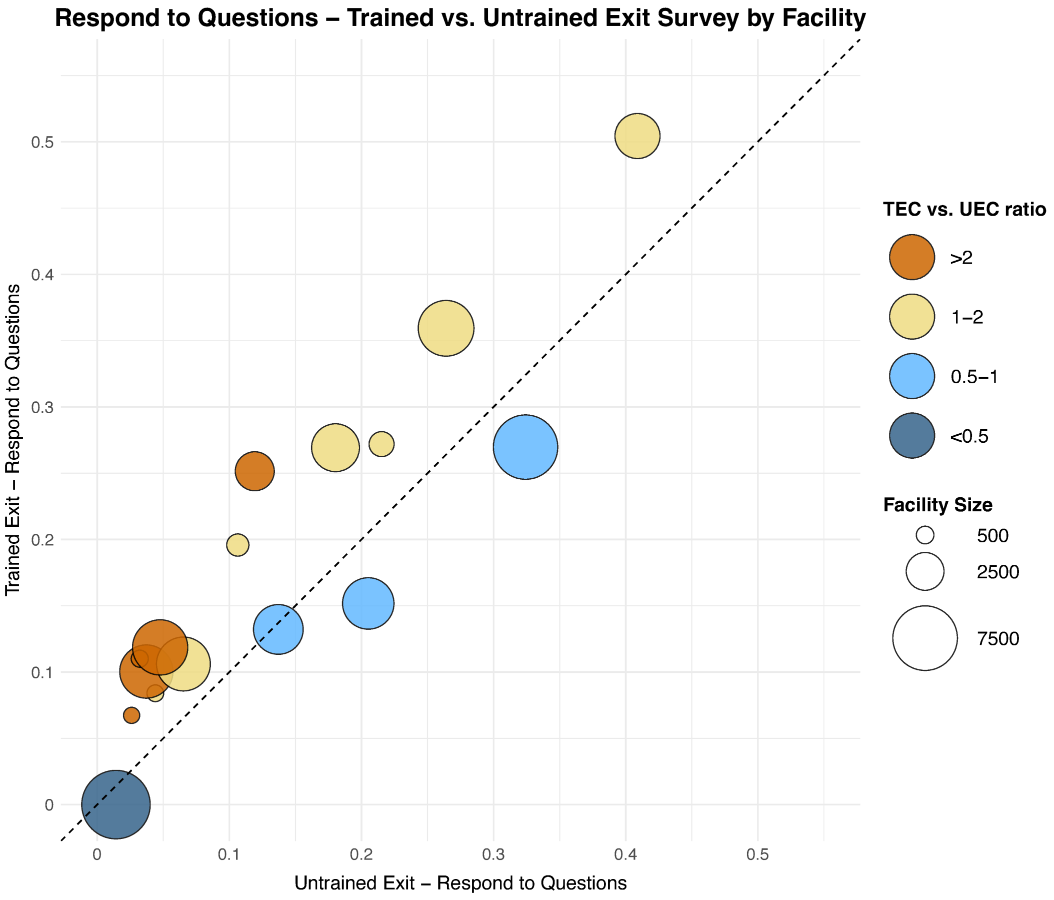
Respond to Questions – Trained vs. Untrained Exit Survey by Facility
Trained Exit – Respond to Questions
Untrained Exit – Respond to Questions
0
0.1
0.2
0.3
0.4
0.5
TEC vs. UEC ratio
>2
1–2
0.5–1
<0.5
Facility Size
500
2500
7500

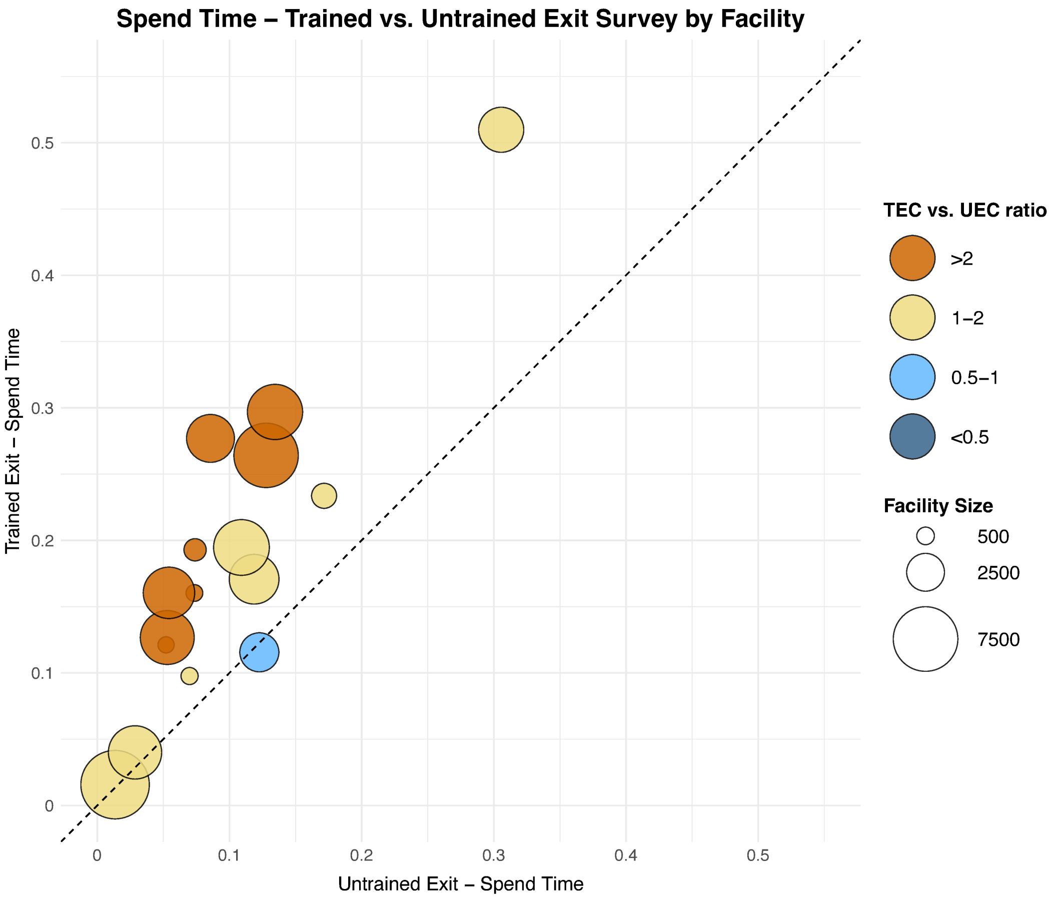
Spend Time – Trained vs. Untrained Exit Survey by Facility
Trained Exit – Spend Time
Untrained Exit – Spend Time
0
0.1
0.2
0.3
0.4
0.5
TEC vs. UEC ratio
>2
1−2
0.5−1
<0.5
Facility Size
500
2500
7500

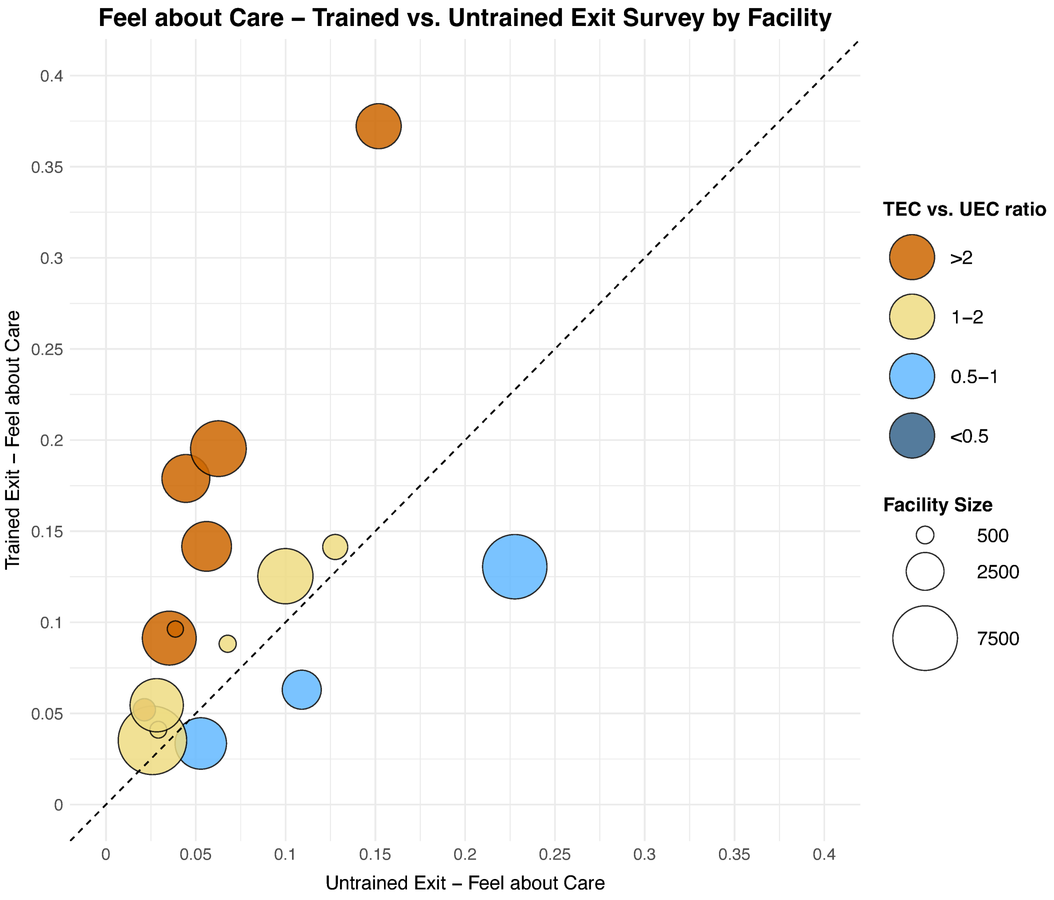
Feel about Care – Trained vs. Untrained Exit Survey by Facility
Trained Exit – Feel about Care
Untrained Exit – Feel about Care
0
0.05
0.1
0.15
0.2
0.25
0.3
0.35
0.4
TEC vs. UEC ratio
>2
1–2
0.5–1
<0.5
Facility Size
500
2500
7500

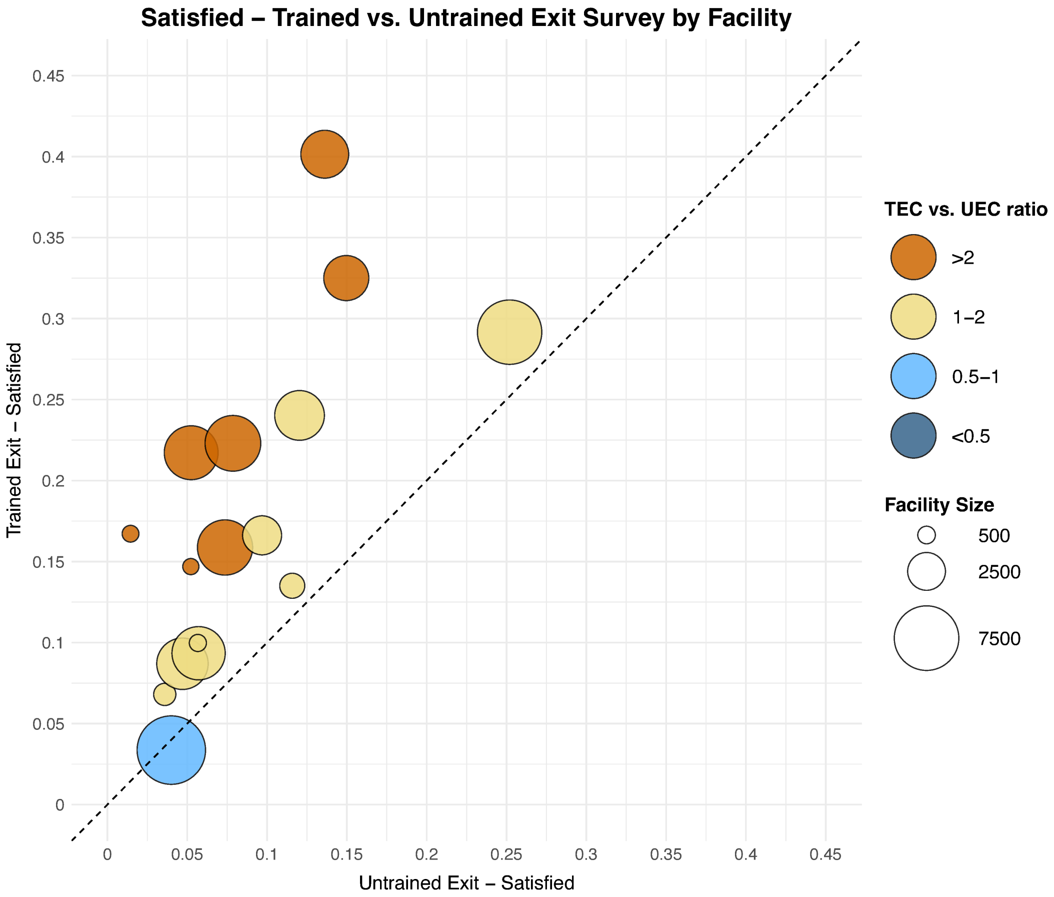

Sikombe K, *et al. BMJ Open* 2023; 13:e069086. doi: 10.1136/bmjopen-2022-069086

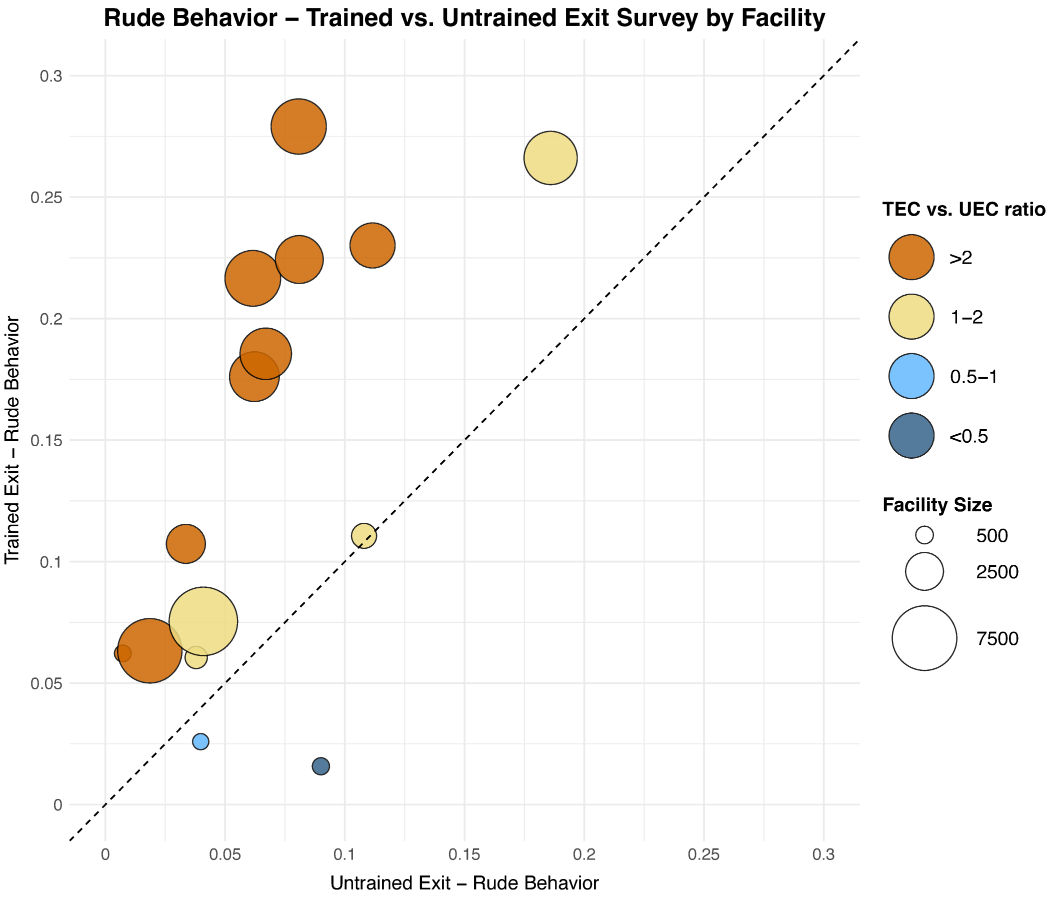
Rude Behavior – Trained vs. Untrained Exit Survey by Facility
Trained Exit – Rude Behavior
Untrained Exit – Rude Behavior
0
0.05
0.1
0.15
0.2
0.25
0.3
TEC vs. UEC ratio
>2
1–2
0.5–1
<0.5
Facility Size
500
2500
7500

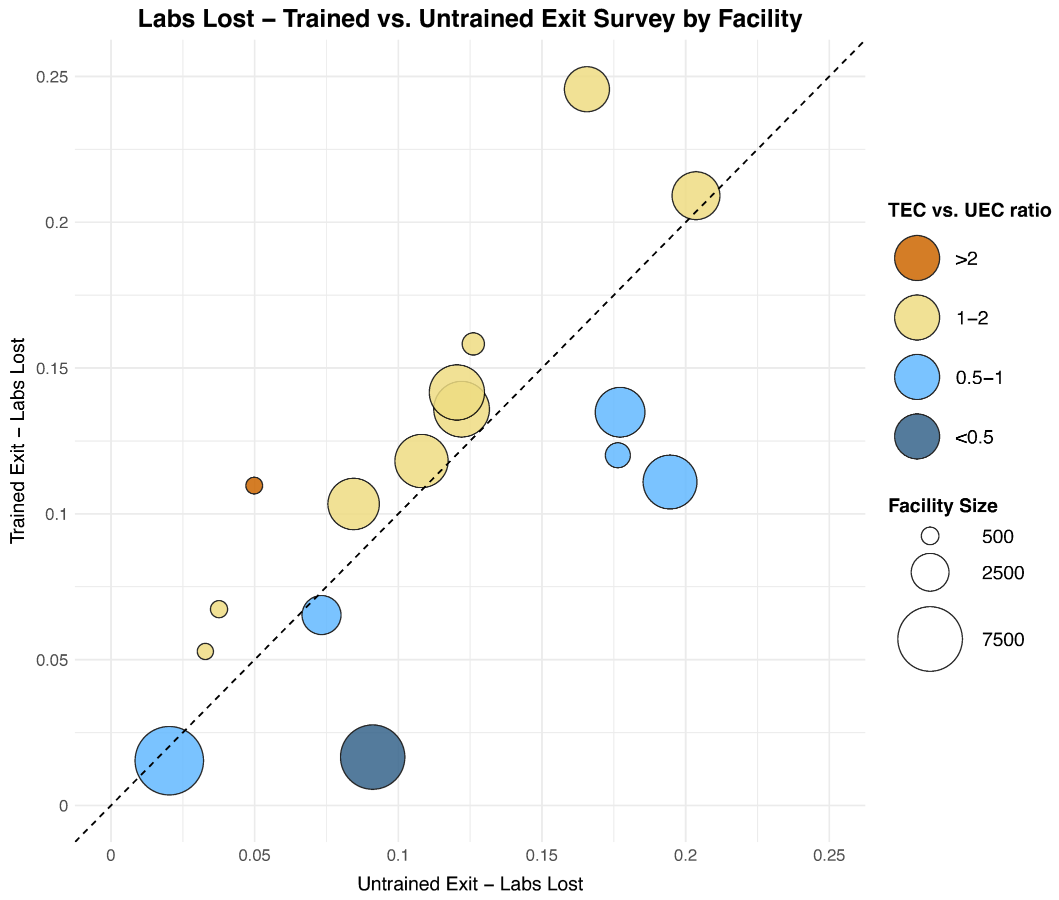

Sikombe K, *et al. BMJ Open* 2023; 13:e069086. doi: 10.1136/bmjopen-2022-069086
