# Supplementary material for: Comparison of patient exit interviews with unannounced standardised patients for assessing HIV service delivery in Zambia: a study nested within a cluster randomised trial
Source: BMJ Open. 2023 Jul 5;13(7):e069086. doi: 10.1136/bmjopen-2022-069086 (PMC10335575; doi:10.1136/bmjopen-2022-069086)
Supplement: Author's manuscript [file bmjopen-2022-069086.draft_revisions.pdf]

BMJ Open is committed to open peer review. As part of this commitment we make the peer review history of every article we publish publicly available.

When an article is published we post the peer reviewers' comments and the authors' responses online. We also post the versions of the paper that were used during peer review. These are the versions that the peer review comments apply to.

The versions of the paper that follow are the versions that were submitted during the peer review process. They are not the versions of record or the final published versions. They should not be cited or distributed as the published version of this manuscript.

BMJ Open is an open access journal and the full, final, typeset and author-corrected version of record of the manuscript is available on our site with no access controls, subscription charges or pay-per-view fees (http://bmjopen.bmj.com).

If you have any questions on BMJ Open's open peer review process please email info.bmjopen@bmj.com

BMJ Open

Understanding the patient experience to improve HIV service delivery using an adapted standardised patient approach in 16 facilities in Zambia

Journal:	*BMJ Open*
Manuscript ID	bmjopen-2022-069086
Article Type:	Original research
Date Submitted by the Author:	19-Oct-2022
Complete List of Authors:	Sikombe, Kombatende; Center for Infectious Disease Research in Zambia, Implementation Science Unit; London School of Hygiene and Tropical Medicine Faculty of Public Health and Policy, Department of Public Health, Environments and Society<br>Pry, Jake; Center for Infectious Disease Research in Zambia, Implementation Science Unit<br>Mody, Aaloke; Washington University in St Louis School of Medicine, Internal Medicine<br>Rice, Brian ; London School of Hygiene and Tropical Medicine Faculty of Public Health and Policy, Department of Public Health, Environments and Society<br>Bukankala, Chama; Center for Infectious Disease Research in Zambia, Research<br>Eshun-Wilson, Ingrid ; Washington University in St Louis School of Medicine, Internal Medicine<br>Mutale, Jacob; Center for Infectious Disease Research in Zambia, Implementation Science Unit<br>Simbeza, Sandra; Center for Infectious Disease Research in Zambia, Implementation Science Unit<br>Beres, Laura K ; Johns Hopkins University Bloomberg School of Public Health, Department of International Health<br>Mukamba, Njekwa; Center for Infectious Disease Research in Zambia, Social and Behavioural Science Research Group<br>Mukumbwa-Mwenechanya, Mpande; Center for Infectious Disease Research in Zambia, Implementation Science Unit<br>Mwamba, Daniel; Center for Infectious Disease Research in Zambia, Clinical Care Services<br>Sharma, Anjali; Center for Infectious Disease Research in Zambia, Social and Behavioural Science Research Group<br>Wringe, Alison; London School of Hygiene and Tropical Medicine Faculty of Epidemiology and Population Health<br>Hargreaves, James; London School of Hygiene and Tropical Medicine Faculty of Public Health and Policy, Department of Public Health, Environments and Society<br>Bolton-Moore, Carolyn; Center for Infectious Disease Research in Zambia, Clinical Care Services ; The University of Alabama at Birmingham Department of Medicine, Infectious Disease<br>Holmes, Charles; Georgetown University Medical Center, Center for Innovation in Global Health

For peer review only - http://bmjopen.bmj.com/site/about/guidelines.xhtml

	Sikazwe, Izukanji; Center for Infectious Disease Research in Zambia, Implementation Science Unit<br>Geng, Elvin; Washington University in St Louis School of Medicine, Internal Medicine
Keywords:	Public health < INFECTIOUS DISEASES, Organisation of health services < HEALTH SERVICES ADMINISTRATION & MANAGEMENT, Quality in health care < HEALTH SERVICES ADMINISTRATION & MANAGEMENT, HIV & AIDS < INFECTIOUS DISEASES, PUBLIC HEALTH

I, the Submitting Author has the right to grant and does grant on behalf of all authors of the Work (as defined in the below author licence), an exclusive licence and/or a non-exclusive licence for contributions from authors who are: i) UK Crown employees; ii) where BMJ has agreed a CC-BY licence shall apply, and/or iii) in accordance with the terms applicable for US Federal Government officers or employees acting as part of their official duties; on a worldwide, perpetual, irrevocable, royalty-free basis to BMJ Publishing Group Ltd ("BMJ") its licensees and where the relevant Journal is co-owned by BMJ to the co-owners of the Journal, to publish the Work in this journal and any other BMJ products and to exploit all rights, as set out in our licence.

The Submitting Author accepts and understands that any supply made under these terms is made by BMJ to the Submitting Author unless you are acting as an employee on behalf of your employer or a postgraduate student of an affiliated institution which is paying any applicable article publishing charge ("APC") for Open Access articles. Where the Submitting Author wishes to make the Work available on an Open Access basis (and intends to pay the relevant APC), the terms of reuse of such Open Access shall be governed by a Creative Commons licence – details of these licences and which Creative Commons licence will apply to this Work are set out in our licence referred to above.

Other than as permitted in any relevant BMJ Author's Self Archiving Policies, I confirm this Work has not been accepted for publication elsewhere, is not being considered for publication elsewhere and does not duplicate material already published. I confirm all authors consent to publication of this Work and authorise the granting of this licence.

For peer review only

Understanding the patient experience to improve HIV service delivery using an adapted standardised patient approach in 16 facilities in Zambia

Kombatende Sikombe[1,2], Jake M Pry[1,3], Aaloke Mody[4], Brian Rice, Chama Bukankala[1], Ingrid Eshun-Wilson[4], Jacob Mutale[1], Sandra Simbeza[1], Laura K Beres[5], Njekwa Mukamba[1], Mpande Mukumbwa-Mwenechanya[1], Daniel Mwamba[1], Anjali Sharma[1], Alison Wringe[6], James Hargreaves[1], Carolyn Bolton Moore[1,7], Charles B Holmes[8], Izukanji Sikazwe[1], Elvin Geng[4]

[1]Centre for Infectious Disease Research in Zambia, Lusaka, Zambia, [2]Faculty of Public Health and Policy, London School of Hygiene & Tropical Medicine, London, UK, [3]Department of Public Health Sciences, University of California, Davis, CA, USA, [4]Division of Infectious Diseases, Washington University in St Louis, St. Louis, MO, USA, [5]Department of International Health, Johns Hopkins Bloomberg School of Public Health, Baltimore, MD, USA, [6]Faculty of Epidemiology and Population Health, London School of Hygiene and Tropical Medicine, London, UK, [7]Department of Medicine, School of Medicine, University of Alabama at Birmingham, Birmingham, AL, USA, [8]Center for Innovation in Global Health, Georgetown University, Washington, DC, USA.

Corresponding Author: Kombatende Sikombe
Affiliations: Centre for Infectious Disease Research in Zambia, Lusaka, Zambia, Faculty of Public Health and Policy, London School of Hygiene & Tropical Medicine, London, UK
Email Address: kombatende.sikombe@cidrz.org

For peer review only - http://bmjopen.bmj.com/site/about/guidelines.xhtml

ABSTRACT

Objectives: To evaluate sub-optimal patient experiences in HIV care (e.g., unfriendly interactions with health care workers [HCW], long-waiting times, and lost laboratory results) using an adapted standardised patient approach "mystery-clients" that addresses information and social desirability biases.

Setting: Cross-sectional surveys in 16 government-operated HIV primary care clinics in Lusaka, Zambia providing antiretroviral-therapy (ART).

Participants: 3526 participants ≥18 years of age receiving ART participated in the exit surveys between August 2019 and November 2021.

Intervention: Patients systematically sampled from the clinic waiting area willing to be trained received pre-visit training and post-visit interviews. HCWs were unaware of trained patients.

Outcome measures: We assessed patient experience among patients that received a brief training prior to their care visit (explaining each patient experience construct in the exit survey, being anonymous, not altering behaviour or manipulating interactions) with those who did not undergo training on the instrument prior to their visit.

Results: Among 3526 participants who participated in the exit surveys, 2415 were untrained (56% female, median age 40 (IQR:32-47)) and 1111 were trained (50% female, median age 37 (IQR:31-45)). Compared to untrained, trained patients were more likely to report a negative care experience overall (adjusted Prevalence Ratio aPR for aggregate sum score: 1.64 [95% CI:1.39-1.94]), with a greater proportion reporting feeling unwelcomed by providers ([aPR]: 1.71 [95% CI:1.20-2.44]) and witnessing providers behaving rudely (aPR: 2.28 [95% CI:1.63-3.19]).

Conclusion: Trained patients were more likely to identify instances of sub-optimal care. They may have had a better understanding of the items solicited or felt empowered to be more critical. Unlike studies where "standardised patients" are drawn from outside of the patient population, we trained existing patients. This low-cost strategy might be used in other settings to improve patient-centred service delivery.

Trial registration: Assessment was nested within a parent study. www.pactr.org registered the parent study (PACTR202101847907585).

For peer review only - http://bmjopen.bmj.com/site/about/guidelines.xhtml

STRENGTHS AND LIMITATIONS OF THIS STUDY

- Patient experience is hard to measure rigorously and pragmatically. Approaches to assessing patient experience such as standardised patient (SP) surveys are often referred to as the golden standard. However, these methods are not feasible in HIV care settings with limited resources because they frequently require highly skilled personnel and are typically utilised for episodic care. The current procedure for SP calls for a trained individual to make one visit to many locations while acting as a simulated patient.

- We present a novel approach to measuring patient experience using an adapted version of the standardised patient approach. In our method, we trained actual patients to look out for certain facility characteristics, to evaluate key components of quality of care such as waiting times, communication, respectfulness of providers, and privacy and compared their responses to traditional exit survey responses in 16 health facilities in Zambia.

- Recipients of care who received a brief training provided more critical appraisal of care either because they were more alert to the items solicited or felt empowered to be more critical. Patient-centred care can be institutionalised further through the evaluation of what occurs at the point of contact between the patient, the healthcare facility, and the healthcare professional using this low-cost method.

For peer review only

BACKGROUND

Retention in care remains a major obstacle to improving Human Immunodeficiency Virus (HIV) treatment outcomes, and health systems in low-income settings like Zambia, have sought to shift their public health response by designing and delivering high quality and patient-centred HIV care [1–6]. Efforts to improve service quality and patient experience require systematic measurement of the patient experience to guide facility responses as poor patient experience has been shown to lead to disengagement from care [7–11]. Health policymakers and donors, such as the President's Emergency Plan for AIDS Relief (PEPFAR), have invested in clinical metrics to assess care quality in Zambia and the wider region, but to a lesser extent in non-clinical metrics like patient experience [12]. These metrics can be critical for guiding efforts to improve retention in care by ensuring an informed response to improving quality of care and patient centredness.

Accurate and pragmatic measurement of the patient experience poses a range of challenges. Patient experience exit surveys are prone to social desirability bias because of power dynamics in health care. Empirical studies of satisfaction, for example, are widely believed to over-estimate patient satisfaction [13]. This may be particularly true where provider-patient relationships are traditional and hierarchical. Delaying surveys for some time after the encounter is theorised to ameliorate social desirability bias, but in turn may exacerbate bias due to simple inability to remember — thus creating recall bias [6]. Other methods such as direct clinical observations of care pose practical difficulties [13,14]. For example, direct observations may be intrusive and therefore may not reflect everyday functionality of a health facility. Care provided under direct observations may be of higher quality as behaviour may be influenced by observation, a phenomenon often known as the “Hawthorne effect” [13,14].

For peer review only - http://bmjopen.bmj.com/site/about/guidelines.xhtml

Standardised patients (SP), also known as “mystery clients” or “simulated patients” have largely been used to assess quality of care in developed countries, as well as in assessing customer service in the retail industry[15]. SP can be resource-intensive and require training, but reduce potential for recall bias, social desirability, bias, and Hawthorne effects, providing an opportunity for optimal assessment of patient satisfaction among people receiving HIV care [6,16]. They have largely been used for episodic care where a highly skilled and well-trained person poses as a client by making one visit to multiple facilities. This approach holds promise for assessing the patient experience in HIV care but poses pragmatic challenges when assessing the quality of chronic care in which a patient makes multiple visits and may compromise efficiency at, already overburdened, facilities [17–22]. In this study, we report on the development and evaluation of a modified SP approach in which we trained real patients (trained exit clients - TEC) to report on certain characteristics of encounters, and rate key components of care such as waiting times, communication, respectfulness of providers, and privacy.

METHODS

Study design & setting

This study seeks to compare two different methods for assessing patient experience: standard exit survey and those reported by patients who had a brief training on the items before the clinical encounter and to whom the clinic was blinded. The assessment was nested within a parent study: the Leveraging Person-Centred Public Health (PCPH) to improve HIV outcomes in Zambia study (www.pactr.org PACTR202101847907585), a Stepped Wedge Cluster Randomised Trial that occurred between August 2019 and November 2021. The aim of the PCPH study was to assess the impact of introducing health care workers (HCW) to a patient-centred care (PCC) curriculum and mentoring them on PCC principles to improve retention and viral suppression in HIV care. The two

For peer review only - http://bmjopen.bmj.com/site/about/guidelines.xhtml

assessments we compare were conducted primarily to evaluate effects of the intervention. This comparative analysis is secondary analysis of the experience endpoints.

Population

The sub-study reported here included 16 health facilities in Lusaka, Zambia, operated by the Ministry of Health (MOH) and receiving technical assistance from the Centre for Infectious Diseases Research in Zambia (CIDRZ) - a Zambian non-governmental organisation (NGO) as well as a part of the larger parent study. We surveyed adults aged 18 years and over who were accessing antiretroviral therapy (ART) at study facilities. Exit survey patients were selected in a systematic sample at the time of exit from the clinic. Trained patients were recruited in the waiting room for their visit, underwent a brief training, and then answered survey questions on exit from their encounter. Participants attending an HIV care visit on the day, able to recall events and comprehend study participant recruitment details (as assessed using the comprehension assessment tool) and able to read and write (assessed using literacy tool) were eligible for inclusion.

Procedures and Measurements

Survey Instrument

For both survey methods, we developed a patient experience instrument based on a previously validated tool developed and used in Kenya: The Wachira Physician-Patient Communication Behaviours Scale [23–25]. This survey assessed elements of patient experience including how they were greeted, communicated to, and overall experience. We included additional questions to capture for example, patient reports of witnessing rude behaviour, receiving appropriate medications and availability of lab results. Prior to use in this study, we performed cognitive interviews among twenty participants to assess consistency in understanding questions in English, Bemba and Nyanja.

For peer review only - http://bmjopen.bmj.com/site/about/guidelines.xhtml

Surveys were forward and back translated to ensure consistency across the three languages. The survey tools for trained and untrained clients were identical. For both trained and untrained clients, all interviews and surveys were conducted in either English, Bemba or Nyanja depending on the participant's preference.

For the untrained exit surveys, among patients leaving the facility after attending the clinic on the survey day, we took a systematic (every kth, varied by facility size) sample of patients. Patients were approached by study staff after the visit using a recruitment script to determine their eligibility and were administered the survey after granting consent. Participants received a snack during the survey administration, but no other financial incentive.

Additional procedures for Trained Exit Client

Efforts to "standardise" assessment of the quality and nature of care in HIV care differs from most previously standardised patient or mystery client work in that HIV care is longitudinal as opposed to episodic or acute care. Under these circumstances, the more conventional standardised patient where a single trained actor can present to multiple different care facilities as a simulated patient with a defined set of symptoms or complaints to assess a single episode of care is not feasible. For example, a patient would have to either register as a new patient or have a false "file" introduced into the paper and electronic medical records — which was deemed infeasible and undesirable.

Instead of simulated patients, we recruited existing patients already receiving care at a particular facility and then subsequently trained them on the concepts of quality of care according to the MOH manual on Quality Improvement for HCWs in Zambia. To avoid disclosing their trained status, patients were recruited prior to them entering the triage area (i.e., the first point of contact with HCWs). Those who consented underwent a single training session for 40 to 60 minutes where they were sensitised to the study instrument (which was the same for both TEC and untrained exit clients

For peer review only - http://bmjopen.bmj.com/site/about/guidelines.xhtml

(UEC)), the MOH care standards, and strategies on being natural yet observant during their clinic visit for that day according to the standard SP approach. These procedures were meant to ensure patients had a clear and uniform understanding on what they should expect during a high-quality patient visit and were attentive to these critical aspects relative to these standards. After this training, the TEC presented themselves to their facility and completed their visit as they normally would. After their clinic encounter, participants then completed the exit survey. Given the extra time commitments required for the training, TEC participants were given K100 (~$5) for the time spent during training. Research assistants were trained by the first author and were available for recruitment, training and administering of the TEC and UEC survey in all 16 facilities. The provincial and district health management teams were informed about the unannounced TEC survey as well as the UEC survey. The study team sensitised all facility staff at the start of the study, but HCWs were not aware of who specific TECs were.

Statistical Analysis

To assess the association between training and response for each question, we conducted unadjusted and adjusted poisson regression for each question separately [26]. We then assessed the overall association between training and total sum score. We used descriptive statistics to characterise patient characteristics and report survey responses. In these analyses, most of the survey responses were reverse coded to identify when respondents reported a negative experience. Results for individual questions (binary response) represent prevalence ratios for reporting a lapse in care. To assess the sum score (count data) we used Poisson regression, estimating the rate ratio for reporting lapses in care. All models were adjusted, given potential differences in survey participants related to different recruitment strategies using mixed-effects regression, adjusted for age, sex,

For peer review only - http://bmjopen.bmj.com/site/about/guidelines.xhtml

education, care status at the time (i.e., continuously retained in care versus returning to care after disengagement/ lost to follow up [LTFU]), secular time (using cubic splines), allowing random effects at the facility level. We present these results for the overall population as well as stratified by different patient subgroups. Lastly, we used bubble plots to compare summary assessments of the patient experience at the facility-level using TECs versus UECs. All analyses were performed using STATA 14MP (StataCorp, College Station, TX, USA). This sub-study represents a secondary analysis and no formal power calculations were performed for this outcome.

Statement of Ethics Approval

Ethics approval to conduct this research was granted by the Zambian Ministry of Health, National Health Research Authority, and the institutional review boards of the University of Zambia (008-03-19), the University of Alabama at Birmingham (300003282) and the London School of Hygiene and Tropical Medicine (21384).

Patient and Public Involvement

Survey questions were developed through a cognitive process with recipients of care. Study implementation guidance was conducted as part of routine CIDRZ partnership with the Zambian MOH through a Human Centered Design workshop. CIDRZ engages with implementing partners and affected communities in health facilities, including people living with HIV often represented by neighbourhood health representatives. Although patients were not directly involved in the design of the parent study intervention or the analysis presented here, all study activities were guided by a Scientific Advisory Board with representation from the MOH and a representative of recipients of HIV care. Dissemination of study results is ongoing.

For peer review only - http://bmjopen.bmj.com/site/about/guidelines.xhtml

RESULTS

Characteristics of health facilities and patients

We approached 4375 clients (2955 in the untrained and 1420 in the trained), and 3526 participated, of which 2415 (55.2%) completed experience surveys as untrained exit clients (UEC) (56% female, median age was 40 years (interquartile range [IQR]:32-47 years)) and 1111 (32%) completed experience surveys as trained exit clients (TEC) (50% female with a median age 37 years (IQR:31-45 years). Reasons for non-participation included unavailability at the time due to other commitments. Sixteen percent (16%) of UECs and 40% of TECs who had been lost to care and were returning to care on the day of the survey. Education levels differed between UEC and TEC with 47% and 58% reporting completion of secondary level of education, respectively (Table 1). UEC and TEC were similar for HIV enrolment WHO stage with the largest proportion enrolling at WHO stage 1 and similar in terms of marital status.

Table 1. Socio-demographic characteristics of untrained exit and trained exit clients

Characteristics	Level	Untrained Exit Clients n=2415 (68%)	Trained Exit Clients n=1111 (32%)
**Sex, n (%)**			
	**Female**	1355 (56)	553 (50)
	**Male**	1060 (44)	558 (50)
**Age, Median (IQR)**			
		40 (32-47)	37 (31-45)
**Age category, n (%)**			
	**<30 years**	453 (19)	258 (23)
	**30-40 years**	828 (34)	416 (37)
	**40-50 years**	815 (34)	304 (27)
	**>50 years**	319 (13)	133 (12)
**Education category**			
	**None**	132 (5)	36 (3)
	**Primary**	654 (27)	166 (15)
	**Secondary**	1134 (47)	645 (58)
	**University**	150 (6)	100 (9)
	**Missing**	307 (13)	151 (14)

For peer review only - http://bmjopen.bmj.com/site/about/guidelines.xhtml

**HIV Enrollment Stage**			
	**WHO Stage 1**	1173 (49)	533 (48)
	**WHO Stage 2**	314 (13)	147 (13)
	**WHO Stage 3**	355 (15)	162 (15)
	**WHO Stage 4**	27 (1)	7 (1)
	**Missing**	546 (23)	262 (24)
**Care status at survey visit**			
	**In care**	2038 (84)	664 (60)
	**Returning to care**	377 (16)	447 (40)
**Marital Status**			
	**Single**	257 (11)	167 (15)
	**Married**	1361 (56)	575 (52)
	**Divorced**	248 (10)	108 (10)
	**Widowed**	173 (7)	81 (7)
	**Unknown**	41 (2)	20 (2)
	**Missing**	335 (14)	160 (14)
**Facility size**			
	**< 1000 patients**	591 (25)	245 (22)
	**1000-5000 patients**	897 (37)	485 (44)
	**> 5000 patients**	927 (38)	381 (34)

Table 2 shows the absolute responses for TEC and UEC. Although most patients reported a good experience, across the questions between 5% and 25% of patients reported poor experiences in care. For example, when asked if their HIV care provider gave them as much information about their health as they wanted, 13.4% (UEC) vs 24.6% (TEC) of patients reported not being provided with sufficient information about their health. Similarly, between 9.6% vs 18.8% patients reported that their HIV care provider was not spending the right amount of time with them at their visit, and 6.8% vs 16.4% reported witnessing rude behaviour.

For peer review only

Table 2. Survey responses by training status

Factor	Level	Untrained Exit Client n (%)	Trained Exit Client n (%)
**Did your HIV care provider greet you in a way that made you feel comfortable?**	Yes	2249 (93.1)	980 (88.2)
	No	166 (6.9)	131 (11.8)
**Did your HIV care provider listen to what you said?**	Yes	2328 (96.4)	1039 (93.5)
	No	79 (3.3)	64 (5.8)
	Refused	8 (0.3)	8 (0.7)
**Did your HIV care provider give you as much information about your health as you wanted?**	Yes	2092 (86.6)	838 (75.4)
	No	323 (13.4)	273 (24.6)
**Did your HIV care provider allow you to ask questions?**	Yes	2082 (86.2)	887 (79.8)
	No	326 (13.5)	222 (20)
	Refused	7 (0.3)	2 (0.2)
**Did your HIV care provider spend the right amount of time with you?**	Yes	2179 (90.2)	900 (81)
	No	232 (9.6)	209 (18.8)
	Refused	4 (0.2)	2 (0.2)
**Overall, how did you feel about the care you received today?**	Happy	2231 (92.4)	983 (88.5)
	Unhappy	178 (7.4)	123 (11.1)
	Refused	6 (0.2)	5 (0.4)
**Overall, were you satisfied with all your HIV care providers today?**	Yes	2206 (91.4)	906 (81.5)
	No	208 (8.6)	202 (18.2)
	Refused	1 (0.0)	3 (0.3)
**I witnessed HIV care providers behaving rudely during my visit today**	No	2251 (93.2)	928 (83.5)
	Yes	163 (6.8)	182 (16.4)
	Refused	1 (0.0)	1 (0.1)
**Were your lab results lost?**	No	2143 (88.7)	985 (88.7)
	Yes	268 (11.1)	126 (11.3)
	Not picking up	4 (0.2)	0 (0)
**Were you able to pick up your medicine today?**	Yes	2366 (98.0)	1087 (97.8)
	No	48 (2.0)	24 (2.2)
	Not Picking Up Meds	1 (0.0)	0 (0)

Effects of training on response patterns: sum score and prevalence ratios

In adjusted models, TECs overall reported poor experiences in care: 1.64 times as frequently as UEC respondents (Sum Score Rate Ratio [RR]: 1.64 [95% CI: 1.39-1.94] (Fig 1, Supplementary Table S1), and reported an increased prevalence of poor experiences in care quality compared to

For peer review only - http://bmjopen.bmj.com/site/about/guidelines.xhtml

312 untrained across almost all questions. For example; among TECs compared to UECs, there was an increased prevalence of reports of not being greeted in a way that made them feel welcome (adjusted Prevalence Ratio [aPR]: 1.71 [95% CI: 1.20-2.44]), reporting being dissatisfied with all their HIV care providers during their HIV care visit (aPR: 2.06 [95% CI: 1.61-2.63]) and witnessing any providers behaving rudely during their visit (aPR: 2.28 [95% CI: 1.63-3.19]) (Fig 1, Supplementary Table S1).

Impact of training across age, sex, and gender to differences in responses

In stratified analysis of the impact of training on the sum score, training was consistently associated with increased identification of poor experiences in care across all subgroups apart from those aged 50 years or older and those with no education. We also observed that training had a larger impact among females compared to males, those with a primary education only, and among individuals presenting at smaller facilities (Fig 2). We observed similarities in responses on the impact of training on different age categories, sex, care status and different levels of education when we looked at individual questions except for the question on providers spending the right amount of time where we found that females were twice as likely to report lapses with care compared to males (Supplementary Figure 1). Using TECs gave worse assessments of patient experience at the facility-level regardless of facility size compared to UECs (Fig 3, Supplementary Figure 2).

DISCUSSION

Disengaged patients often express a disconnect between their care expectations and the provider's style, hence experience is bound to vary across facilities [7]. This disconnect can lead to dissatisfaction with HIV services which can often lead to patients dropping out of care[7,10,27]. A

For peer review only

brief training for patients living with HIV on how to evaluate the quality and experience of routine care changed patient experience reports compared to untrained patients using the same instrument. Patients who underwent a brief training identified more lapses in care across most questions. Women and young people were more likely to report critical responses after training - consistent with the idea that those who feel least empowered underwent the biggest change. Differences were also bigger for questions in which social desirability is likely to operate. For example, larger differences were observed for witnessing rude behaviour, while no differences were observed for more objective questions such as whether lab results were lost.

Improving HIV health outcomes requires new strategies that minimise methodological biases and includes everyone the patient encounters during their visit, including clinical officers, doctors, nurses, data clerks, and lay HCWs. Our TEC approach could contribute to getting a true reflection of how much value patients place on things such as effective communication, being greeted appropriately, or being treated with care and respect at all these different touch points. Involving patients in their own care and design of health services has been linked to improved HIV care retention and patient outcomes, such as higher viral suppression rates [28–30]. As progress is being made towards UNAIDS 95-95-95 targets, the global HIV sector is constantly reviewing priorities and challenges for optimal engagement in care [31,32]. Patient experience is a key indicator of healthcare quality for meeting the 95-95-95 targets: delivering services patients need, can access, and address wider determinants of poor health. Clinicians and health systems must address HIV patients' needs from diagnosis to death to ensure healthy ageing and viral suppression. Other outcomes in Zambia [10,33,34] show that lifelong needs vary by facility, highlighting the importance of metrics that measure patient experience accurately. We have shown that it is feasible

For peer review only - http://bmjopen.bmj.com/site/about/guidelines.xhtml

to involve patients in assessing the quality of care and this could potentially lead to involvement of patients in the redesign of healthcare services.

Because HIV care is longitudinal, SP, who are often used to evaluate episodic care, require highly skilled people to pose as a simulated patient making one visit to multiple clinics, posing practical implementation challenges in our setting[17–22]. Contrary to SP, we evaluated care quality without using simulated patients and administered the survey once among people in long term care. Using real patients instead of simulated ones drawn from outside the true patient population, we would argue, made our TEC approach more applicable and reproducible in clinical settings. We were able to record HCW behaviour in a typical HIV context using this concealment method, potentially reducing the impact of the Hawthorne effect. Our TECs also consistently identified more lapses in care, potentially reducing social desirability bias and ability to identify issues at the facility. Even though training takes time, the increased quality of our measurement allows one to perform fewer surveys. With traditional approaches like exit surveys, one would require a larger sample size, but this does not address bias [35].

Our findings are consistent with a study done in South Africa which found that non-clinical dimensions of care play a bigger role in determining an overall satisfactory experience for standardised patients when compared to untrained patients[35]. However, our findings may contradict previous suggestions that tailoring support to individuals to build skills and confidence through patient activation can lead to trained/informed patients reporting a better experience than untrained/ uninformed [36]. TECs cared about the following non-clinical aspects of care: rude providers, being satisfied with HIV care providers, and spending enough time with providers. This finding is consistent with a previous study in Zambia, where patients reported rude HCWs deterring HIV care engagement [7,9,10]. This could mean that studies assessing patient experience with TEC

For peer review only

could focus on a few questions to save time and resources. Questions like, "Did you pick up your medicine or lab results at your visit?" may not add much to a TEC survey because they are definitive, and training appears to influence subjective care dimensions.

Women TECs were generally more critical about the care they received and would likely provide a more accurate reflection of the health system, possibly because they have better health-seeking behaviour than men, which may be strongly influenced by local gender norms and health service structures designed to engage women of reproductive age [37]. There is some consistency with other findings that women may be more interested in their care than men, especially in facilities that provide integrated services for women and their children [8,38]. Despite longer wait times, women were more satisfied with integrated facilities [39]. In addition, middle-aged people between 40-50 benefited the most from training. Compared to older people over 50, younger people under 30 were less satisfied with the care they received and often felt they were not greeted by a HCW during their visit. This finding is consistent with cultural norms where younger people are less respected[40]. Given the current strategy of targeting young people, who account for most new infections, these findings suggest an important new approach to identifying what young people value most. Education level was among the strongest predictors of patient experience feedback. Well-educated patients were found to have a less critical/better HIV care visit experience compared to participants with lower levels of educational attainment. This difference in care experience report may be associated, at least in part, with the HCW perception of the patient in the facility. Research conducted in Nigeria discovered that people with higher levels of education are frequently given better and more considerate treatment by HCW, hence limited by a form of discrimination/ socioeconomic status bias [41,42].

For peer review only

For peer review only - http://bmjopen.bmj.com/site/about/guidelines.xhtml

The observed effect of training on patient experience is likely multifaceted potentially stemming from increased attention and recall to the exit survey items which solicited a feeling of empowerment to be more critical of the care received. In future studies, patient activation should be measured as an outcome to see how training changes the patient's engagement with their care over time [36]. Further research is required into why women TECs reported poorer experiences with care than men. Other studies that have used SP to assess medical students' performance showed that women were more critical on certain aspects of care. These studies also recommend matching of SPs to clinicians by sex [43], something we were not able to do given the nature of our study in primary health facilities where we assessed interpersonal communication with HCWs at all levels. Perhaps our findings call for more investigation into the integration of women's services, such as family planning and children's services with HIV care given some studies have shown this can improve patient satisfaction.

Limitations

Our findings should be interpreted with caution due to the following limitations. Because this was the first time such a study was done, we recruited educated participants who were able to read and write, perceived to have good recall ability and were able to comprehend things. Our study was only done in Lusaka province in facilities that were largely urban except for one facility which was peri-urban hence it is hard to generalise these findings. Another limitation in our approach is the one-time cross-sectional nature of our measurements among people in long term HIV care. If more measures were collected from each TEC, we may well see them being activated in a way that results in an improvement in their experience based on the skills they develop to seek better care from providers which ultimately would improve their retention in care. Despite its limitations, the TEC method provides valuable information about healthcare quality, even though it is limited to

For peer review only

situations where "walk-ins" are permitted. Our approach only focused on real patients accessing care and we did not manipulate any patient files, so it is possible that some TECs were known to the facility as patients accessing chronic care. Our approach does require a trained interviewer to speak with TECs after their visits, but this is not any different to what already exists. In future, it may be worth using the domains in the national HIV guidelines as the gold standard, but we did not do this as our aim was to come up with a low-cost approach that can easily be rolled out. In addition, the concept of patient centred care is still catching on in Zambia. Our TEC approach can be used to further the knowledge in provider attitudes to other relatively new approaches to delivering quality HIV care such as differentiated service delivery (DSD) for stable patients by assessing whether HCWs follow guidelines when offering this [32]. We also see an opportunity to assess provider patient communication of viral load laboratory results by use of a universal script for each TEC to assess if they are communicated to and if unsuppressed but adherent, what procedures followed.

Conclusion

TEC offers pragmatic methods for health systems in low-income countries to assess non-clinical dimensions of care (communication, respect, and autonomy) which are grounded on the concept of health-system responsiveness and could be critical to the transformation of low-quality health systems to high quality ones[44]. Hawthorne effects and social desirability biases may be mitigated using TECs. We were able to capture HCWs behaviour in a normal day to day setting [45]. Our findings suggest that TECs provide a more critical appraisal of some aspects of the quality of HIV care. It provides new insights in the Zambian context on what patients' value when they interact with the health system. This could be important given the need to reduce loss to follow up among new ART clients who disengage within the first 6 months of treatment due to a bad first

For peer review only - http://bmjopen.bmj.com/site/about/guidelines.xhtml

encounter with the health system. Our TEC approach could be used to assess reengagement interventions. The fact that TECs had a better understanding of the items solicited or felt empowered to be more critical shows that the training we provided worked. This low-cost method could be reproduced in other routine settings and presents an opportunity to further institutionalise patient centred care by evaluating what happens at the point of contact between the patient, the health facility, and the health provider. The implications are that it provides an opportunity to improve HIV care, meet patients' expectations and can serve as a monitoring tool for healthcare performance. Coupled with the recent approaches to client led monitoring in HIV care, our approach can be used to enhance decision making that considers patients' involvement.

For peer review only

For peer review only - http://bmjopen.bmj.com/site/about/guidelines.xhtml

Data Availability Statement

The Government of Zambia allows data sharing when applicable local conditions are satisfied. In this case, the data from the study will be made available to any interested researchers upon request. The CIDRZ Ethics and Compliance Committee is responsible for approving such request. To request data access, one must write to the Secretary to the Committee/Head of Research Operations, Mrs. Hope Chinganya (Hope.Chinganya@cidrz.org) mentioning the intended use for the data. The Committee will then facilitate review and authorization to release the data as requested. Data requests must include contact information, a research project title, and a description of the intended use.

Funding

This work was supported, in whole or in part, by the Bill & Melinda Gates Foundation [INV-010563]. Under the grant conditions of the Foundation, a Creative Commons Attribution 4.0 Generic License has already been assigned to the Author Accepted Manuscript version that might arise from this submission. This work was also supported by the National Center for Advancing Translational Sciences (grant KL2 TR002346 to Dr Mody) and the National Institute of Allergy and Infectious Diseases (grant K24 AI134413 to Dr Geng). The funders had no role in study design, data collection and analysis, decision to publish, or preparation of the manuscript.

Disclaimer

The contents of this paper are the sole responsibility of the authors and do not necessarily reflect the views of the Bill & Melinda Gates Foundation.

Competing interest: Non declared

For peer review only

Acknowledgements

We would like to thank recipients of care for taking part in this study. We would like to thank the Zambian Ministry of Health, the US President's Emergency Plan for AIDS Relief (PEPFAR) through the US Centers for Disease Control and Prevention/Zambia (CDC) and the Centre for Infectious Disease Research in Zambia for their leadership in ensuring those in HIV care continue to receive life-saving HIV treatment. We would also like to thank the healthcare workers who continued to faithfully deliver HIV care in the face of the COVID-19 pandemic

Contributors

KS: guarantor, lead author, conducted all analyses, led data management activities, field coordination of activities and designed data collection tools. JMP: field coordination of data, assisted with analysis and revising it critically for important intellectual content, designed data collection tools. AM: assisted with analysis, framing, and revising it critically for important intellectual content. BR: final approval for publication, assisted with framing and revising it critically for important intellectual content CB: field coordination of data quality processes and data acquisition IEW: drafted statistical analysis plan, assisted with conceptualisation and interpretation of data. JM: assisted with data acquisition and cleaning, field coordination of data quality processes. SS: led intervention implementation, project administration and data curation. LKB, NM, AS: cognitively tested data collection tools, assisted with conceptualization, underlying data processes, and assisted with writing and data interpretation. DM, MM-M: advised implementation details. AW, JH: assisted with framing and revising it critically for important intellectual content. CBM: lead for underlying data processes and assisted with funding acquisition. CBH: funding acquisition, assisted with conceptualisation and advised regarding intervention implementation details. IS: funding

For peer review only

For peer review only - http://bmjopen.bmj.com/site/about/guidelines.xhtml

acquisition, assisted with conceptualisation and manuscript writing. EHG: funding acquisition, led conceptualisation and advised on all analyses, final approval for publication.

Statement of Ethics Approval

Ethics approval to conduct this research was granted by the Zambian Ministry of Health, National Health Research Authority, and the institutional review boards of the University of Zambia (008-03-19), the University of Alabama at Birmingham (300003282) and the London School of Hygiene and Tropical Medicine (21384).

For peer review only

REFERENCES

1 Institute of Medicine & Committee on Quality of Health Care in America. *Crossing the Quality Chasm: A New Health System for the 21st Century*. 2001. doi:10.1136/bmj.323.7322.1192

2 World Health Organization. Maintaining and Improving Quality of Care Within HIV Clinical Services. 2019. http://apps.who.int/bookorders. (accessed 11 Mar 2020).

3 Edmonds KA, Aspiras OG, Rose JP, *et al.* Cross-sectional evaluation of perceived health care provider engagement, self-efficacy, and ART adherence in people living with HIV/AIDS. *AIDS Care* 2021;**33**:154–8. doi:10.1080/09540121.2019.1703889

4 Bakken S, Holzemer WL, Brown MA, *et al.* Relationships between perception of engagement with health care provider and demographic characteristics, health status, and adherence to therapeutic regimen in persons with HIV/AIDS. *AIDS Patient Care STDS* 2000;**14**:189–97. doi:10.1089/108729100317795

5 Okoli C, Brough G, Allan B, *et al.* Shared Decision Making Between Patients and Healthcare Providers and its Association with Favorable Health Outcomes Among People Living with HIV. *AIDS Behav* 2021;**25**:1384–95. doi:10.1007/s10461-020-02973-4

6 Kwan A, Daniels B, Bergkvist S, *et al.* Use of standardised patients for healthcare quality research in low- and middle-income countries. *BMJ Glob Health* 2019;**4**:e001669. doi:10.1136/bmjgh-2019-001669

7 Mwamba C, Sharma A, Mukamba N, *et al.* 'They care rudely!': resourcing and relational health system factors that influence retention in care for people living with HIV in Zambia. *BMJ Glob Health* 2018;**3**:e001007. doi:10.1136/bmjgh-2018-001007

8 Topp SM, Mwamba C, Sharma A, *et al.* Rethinking retention: Mapping interactions between multiple factors that influence long-term engagement in HIV care. *PLoS One* 2018;**13**:e0193641.https://doi.org/10.1371/journal.pone.0193641

9 Zanolini A, Sikombe K, Sikazwe I, *et al.* Understanding preferences for HIV care and treatment in Zambia: Evidence from a discrete choice experiment among patients who have been lost to follow-up. *PLoS Med* 2018;**15**:e1002636. doi:10.1371/JOURNAL.PMED.1002636

10 Sikazwe I, Eshun-Wilson I, Sikombe K, *et al.* Patient-reported Reasons for Stopping Care or Switching Clinics in Zambia: A Multi-site, Regionally Representative estimate using a multi-stage sampling-based approach in Zambia. *Clinical Infectious Diseases* Published Online First: 2020. doi:10.1093/cid/ciaa1501

11 Mody A, Sikombe K, Beres LK, *et al.* Profiles of HIV Care Disruptions Among Adult Patients Lost to Follow-up in Zambia. *JAIDS Journal of Acquired Immune Deficiency Syndromes* 2020;**Publish Ah**. doi:10.1097/qai.0000000000002530

12 Porter LE, Bouey PD, Curtis S, *et al.* Beyond indicators: Advances in global HIV monitoring and evaluation during the PEPFAR era. *J Acquir Immune Defic Syndr (1988)* 2012;**60**. doi:10.1097/QAI.0b013e31825cf345

13 MILLER FRANCO L, FRANCO C, KUMWENDA N, *et al.* Methods for assessing quality of provider performance in developing countries. *International Journal for Quality in Health Care* 2002;**14**:17–24. doi:10.1093/intqhc/14.suppl_1.17

14 Onishi J, Gupta S, Peters DH. Comparative analysis of exit interviews and direct clinical observations in Pediatric Ambulatory Care Services in Afghanistan. *International Journal for Quality in Health Care* 2011;**23**:76–82. doi:10.1093/intqhc/mzq074

15 Luck J, Peabody JW. Using standardised patients to measure physicians' practice: validation study using audio recordings. *BMJ* 2002;**325**:679. doi:10.1136/bmj.325.7366.679

For peer review only - http://bmjopen.bmj.com/site/about/guidelines.xhtml

16 Beullens J, Rethans JJ, Goedhuys J, *et al.* The use of standardized patients in research in general practice. *Fam Pract* 1997;**14**:58–62. doi:10.1093/fampra/14.1.58
17 Weiner SJ, Schwartz A. Directly observed care: Can unannounced standardized patients address a gap in performance measurement? *J Gen Intern Med* 2014;**29**:1183–7. doi:10.1007/s11606-014-2860-7
18 Nestel D, Bearman M. *Simulated Patient Methodology: Theory, Evidence and Practice*. 2014. doi:10.1002/9781118760673
19 Larsen A, Wilson KS, Kinuthia J, *et al.* Standardised patient encounters to improve quality of counselling for pre-exposure prophylaxis (PrEP) in adolescent girls and young women (AGYW) in Kenya: study protocol of a cluster randomised controlled trial. *BMJ Open* 2020;**10**:e035689–e035689. doi:10.1136/bmjopen-2019-035689
20 Daniels B, Dolinger A, Bedoya G, *et al.* Use of standardised patients to assess quality of healthcare in Nairobi, Kenya: A pilot, cross-sectional study with international comparisons. *BMJ Glob Health* Published Online First: 2017. doi:10.1136/bmjgh-2017-000333
21 Zabar S, Hanley K, Stevens D, *et al.* Unannounced standardized patients: A promising method of assessing patient-centered care in your health care system. *BMC Health Serv Res* 2014;**14**. doi:10.1186/1472-6963-14-157
22 Boffa J, Moyo S, Chikovore J, *et al.* Quality of care for tuberculosis and HIV in the private health sector: a cross-sectional, standardised patient study in South Africa. *BMJ Glob Health* 2021;**6**:e005250. doi:10.1136/bmjgh-2021-005250
23 Wachira J, Middlestadt S, Reece M, *et al.* Psychometric assessment of a physician-patient communication behaviors scale: The perspective of adult HIV patients in Kenya. *AIDS Res Treat* 2013;**2013**. doi:10.1155/2013/706191
24 Campbell C, Lockyer J, Laidlaw T, *et al.* Assessment of a matched-pair instrument to examine doctor‒patient communication skills in practising doctors. *Med Educ* 2007;**41**:123–9. doi:https://doi.org/10.1111/j.1365-2929.2006.02657.x
25 Bieber C, Müller KG, Nicolai J, *et al.* How Does Your Doctor Talk with You? Preliminary Validation of a Brief Patient Self-Report Questionnaire on the Quality of Physician–Patient Interaction. *J Clin Psychol Med Settings* 2010;**17**:125–36. doi:10.1007/s10880-010-9189-0
26 Zou G. A Modified Poisson Regression Approach to Prospective Studies with Binary Data. *Am J Epidemiol* 2004;**159**:702–6. doi:10.1093/aje/kwh090
27 Shabalala FS, Vernooij E, Pell C, *et al.* Understanding reasons for discontinued antiretroviral treatment among clients in test and treat: a qualitative study in Swaziland. *J Int AIDS Soc* 2018;**21**:e25120. doi:10.1002/jia2.25120
28 Grimsrud A, Bygrave H, Doherty M, *et al.* Reimagining HIV service delivery : the role of differentiated care from prevention to suppression. *J Acquir Immune Defic Syndr* 2016;:10–2. doi:10.7448/IAS.19.1.21484
29 Camlin CS, Neilands TB, Odeny TA, *et al.* Patient-reported factors associated with reengagement among HIV-infected patients disengaged from care in East Africa. *AIDS* 2015;:1. doi:10.1097/QAD.0000000000000931
30 Geng EH, Bangsberg DR, Musinguzi N, *et al.* Understanding Reasons for and Outcomes of Patients Lost to Follow-Up in Antiretroviral Therapy Programs in Africa Through a Sampling-Based Approach. *JAIDS Journal of Acquired Immune Deficiency Syndromes* 2010;**53**:405–11. doi:10.1097/QAI.0b013e3181b843f0
31 Ford N, Geng E, Ellman T, *et al.* Emerging priorities for HIV service delivery. *PLoS Med* 2020;**17**:e1003028. doi:10.1371/JOURNAL.PMED.1003028

For peer review only

32 Ehrenkranz P, Grimsrud A, Holmes CB, *et al.* Expanding the Vision for Differentiated Service Delivery: A Call for More Inclusive and Truly Patient-Centered Care for People Living With HIV. *J Acquir Immune Defic Syndr* 2021;**86**:147. doi:10.1097/QAI.0000000000002549
33 Holmes CB, Sikazwe I, Sikombe K, *et al.* Estimated mortality on HIV treatment among active patients and patients lost to follow-up in 4 provinces of Zambia: Findings from a multistage sampling-based survey. *PLoS Med* 2018;**15**:e1002489. doi:10.1371/journal.pmed.1002489
34 Sikazwe I, Eshun-Wilson I, Sikombe K, *et al.* Retention and viral suppression in a cohort of HIV patients on antiretroviral therapy in Zambia: Regionally representative estimates using a multistage-sampling-based approach. *PLoS Med* 2019;**16**:1–17. doi:10.1371/journal.pmed.1002811
35 Hompashe DM, Gerdtham U-G, Christian CS, *et al.* 'The nurse did not even greet me': how informed versus non-informed patients evaluate health systems responsiveness in South Africa. *BMJ Glob Health* 2021;**6**:e004360. doi:10.1136/bmjgh-2020-004360
36 Hibbard JH, Greene J. What The Evidence Shows About Patient Activation: Better Health Outcomes And Care Experiences; Fewer Data On Costs. *Health Aff* 2013;**32**:207–14. doi:10.1377/hlthaff.2012.1061
37 Colvin CJ. Strategies for engaging men in HIV services. Lancet HIV. 2019;**6**:e191–200. doi:10.1016/S2352-3018(19)30032-3
38 Kerkhoff AD, Sikombe K, Eshun-Wilson I, *et al.* Mortality estimates by age and sex among persons living with HIV after ART initiation in Zambia using electronic medical records supplemented with tracing a sample of lost patients: A cohort study. *PLoS Med* 2020;**17**:e1003107.https://doi.org/10.1371/journal.pmed.1003107
39 Narasimhan M, Yeh PT, Haberlen S, *et al.* Integration of HIV testing services into family planning services: a systematic review. *Reprod Health* 2019;**16**:61. doi:10.1186/s12978-019-0714-9
40 Godia PM, Olenja JM, Lavussa JA, *et al.* Sexual reproductive health service provision to young people in Kenya; Health service providers' experiences. *BMC Health Serv Res* 2013;**13**:1–13. doi:10.1186/1472-6963-13-476/TABLES/2
41 Roberts BW, Puri NK, Trzeciak CJ, *et al.* Socioeconomic, racial and ethnic differences in patient experience of clinician empathy: Results of a systematic review and meta-analysis. *PLoS One* 2021;**16**:e0247259.https://doi.org/10.1371/journal.pone.0247259
42 Aloh HE, Onwujekwe OE, Aloh OG, *et al.* Impact of socioeconomic status on patient experience on quality of care for ambulatory healthcare services in tertiary hospitals in Southeast Nigeria. *BMC Health Serv Res* 2020;**20**:473. doi:10.1186/s12913-020-05332-0
43 Humphrey-Murto S, Touchie C, Wood TJ, *et al.* Does the gender of the standardised patient influence candidate performance in an objective structured clinical examination? *Med Educ* 2009;**43**:521–5. doi:10.1111/j.1365-2923.2009.03336.x
44 Kruk ME, Gage AD, Arsenault C, *et al.* High-quality health systems in the Sustainable Development Goals era: time for a revolution. Lancet Glob Health. 2018;**6**:e1196–252. doi:10.1016/S2214-109X(18)30386-3
45 King JJC, Das J, Kwan A, *et al.* How to do (or not to do) … using the standardized patient method to measure clinical quality of care in LMIC health facilities. *Health Policy Plan* 2019;**34**:625–34. doi:10.1093/heapol/czz078

Figure 1. Forest plot comparing responses from Trained Exit Clients (TEC) relative to Untrained Exit Clients (UEC) on 10 measures of clinic experience. Points indicate the rate

For peer review only - http://bmjopen.bmj.com/site/about/guidelines.xhtml

ratio (for sum score) or prevalence ratio (for all others) for identifying a lapse in care in TEC surveys as compared to UEC. The sum score represents the total number of binary responses (yes vs no) across all clients in one group shown as a rate ratio. The red line indicates a rate or prevalence ratio of 1 and values greater than this indicates more lapses in care identified in TECs. Results are based on mixed-effects models adjusted for age, sex, education with a random effect at the facility.

Figure 2. Impact of Training on Identifying Care Lapses Stratified by Subgroups (N=3480). When all questions were collapsed into a Sum score among TEC, females were more likely to report lapses in care quality than males. We observed some level of interaction for care status, age category, education category and facility size.

Figure 3. Bubble plot showing Trained Exit Sum Score vs Untrained Exit Sum Score. Each bubble represents a single facility's performance. Each bubble's size indicates the number of patients at each facility with larger bubbles corresponding to larger facilities. The horizontal position notes the Untrained Exit Sum Score for all questions against the facility, and the vertical position notes the Trained Exit sum score at the same facility.

For peer review only

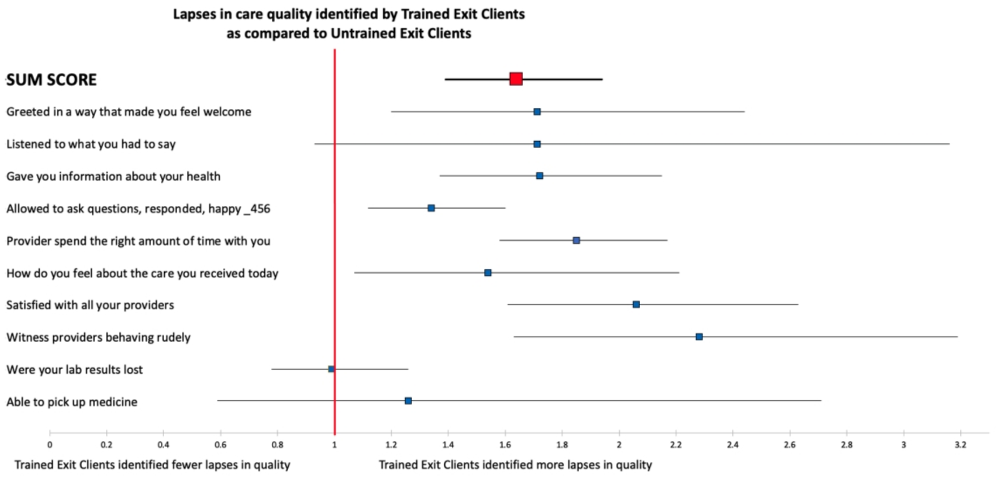

674x330mm (72 x 72 DPI)

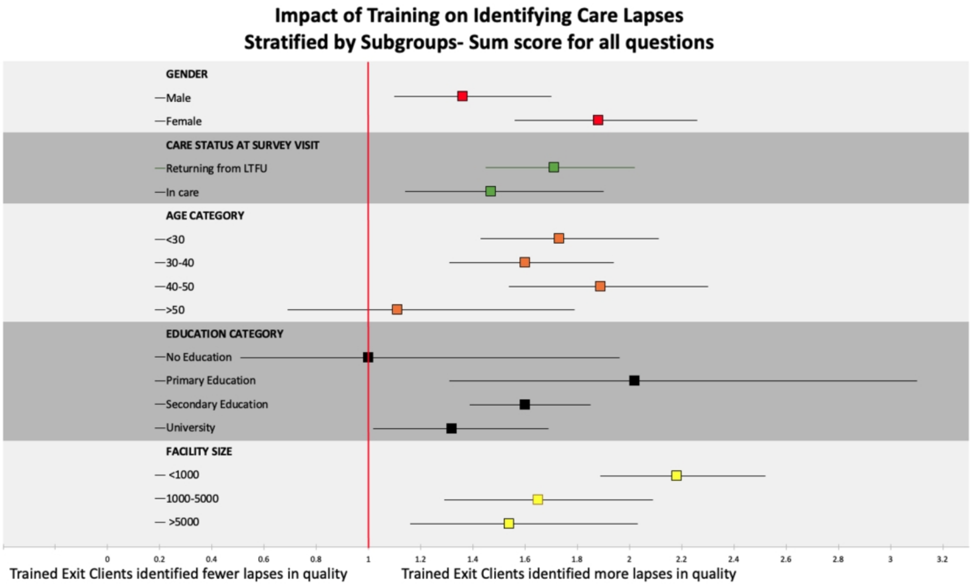

629x374mm (72 x 72 DPI)

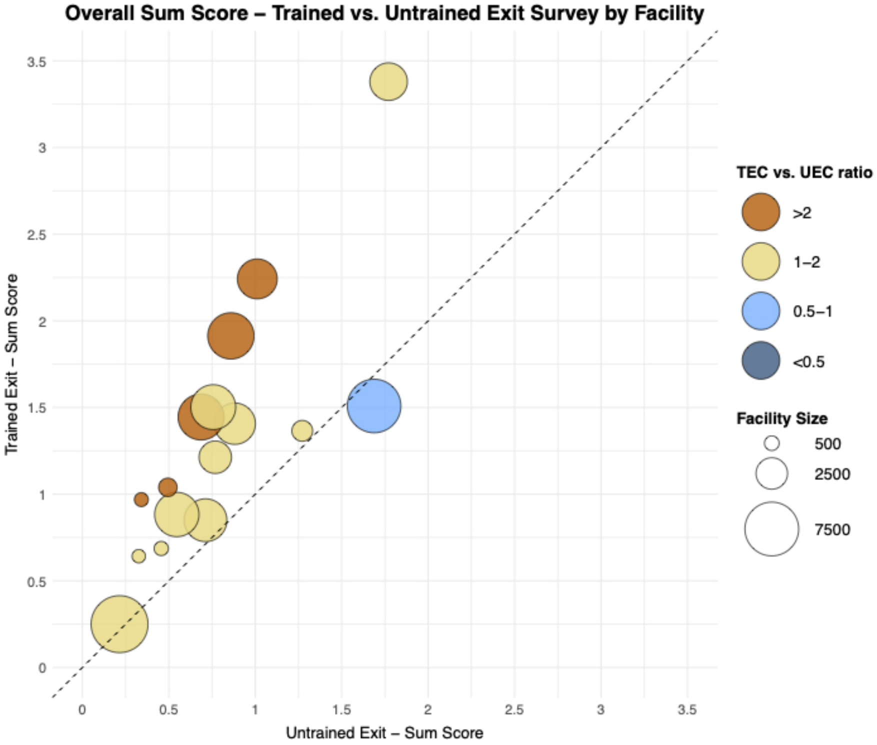

279x215mm (72 x 72 DPI)

Supplementary Tables S1

Supplementary Table S1. Mixed effect Poisson regression comparing 10 questions for Trained Exit Clients vs Untrained Exit Clients. Adjusted for age, sex, education, and study period.

Trained Exit Clients	Prevalence ratio (PR) Unadjusted	P value	95% Confidence Interval (CI)	PR-Adjusted	P value	95% Confidence Interval (CI)	N
Sum score (Rate ratio)	1.73	<0.01	1.47-2.02	1.64	<0.01	1.39-1.94	3480
Did your HIV care provider greet you in a way that made you feel comfortable?	1.74	0.01	1.24-2.44	1.71	<0.01	1.20-2.44	3526
Did your HIV care provider listen to what you said?	1.77	0.09	0.91-3.45	1.71	0.09	0.93-3.16	3510
Did your HIV care provider give you as much information about your health as you wanted?	1.82	<0.01	1.43-2.33	1.72	<0.01	1.37-2.15	3526
Did your HIV care provider allow you to ask questions?	1.44	<0.01	1.20-1.73	1.34	<0.01	1.12-1.6	3517
Did your HIV care provider spend the right amount of time with you?	1.94	<0.01	1.66-2.27	1.85	<0.01	1.58-2.17	3520
Overall, how did you feel about the care you received today?	1.51	0.02	1.06-2.16	1.54	0.02	1.07-2.21	3515
Overall, were you satisfied with all your HIV care providers today?	2.12	<0.01	1.68-2.66	2.06	<0.01	1.61-2.63	3522
I witnessed HIV care providers behaving rudely during my visit today	2.39	<0.01	1.73-3.32	2.28	<0.01	1.63-3.19	3524
Were your lab results lost?	0.99	0.98	0.84-1.19	0.99	0.93	0.78-1.26	3522
Were you able to pick up your medicine today?	1.04	0.90	0.57-1.89	1.26	0.55	0.59-2.71	3525

For peer review only

For peer review only

Supplementary Figure 1.

Impact of Training on Identifying Care Lapses Stratified by Subgroups for 10 questions. We observed some level of interaction for care status, age category, education category and facility size. Panel **a)** Greet you in a way that made you feel comfortable **b)** Listen to what you said **c)** Give you as much information about your health as you wanted **d)** Allowed you to ask questions, responded, happy q456 **e)** spend the right amount of time with you **f)** feel about the care you received today **g)** satisfied with all your HIV care providers today **h)** witnessed HIV care providers behaving rudely during my visit today **i)** lost lab results **j)** pick up meds

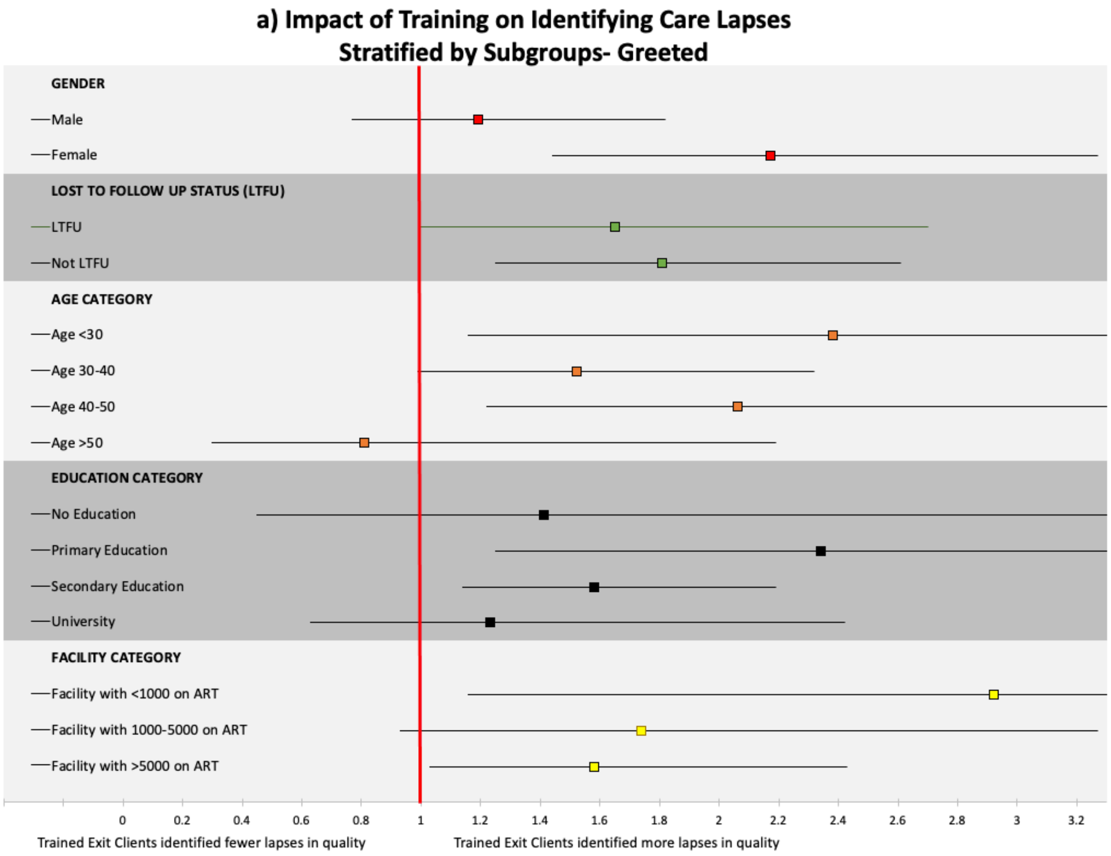

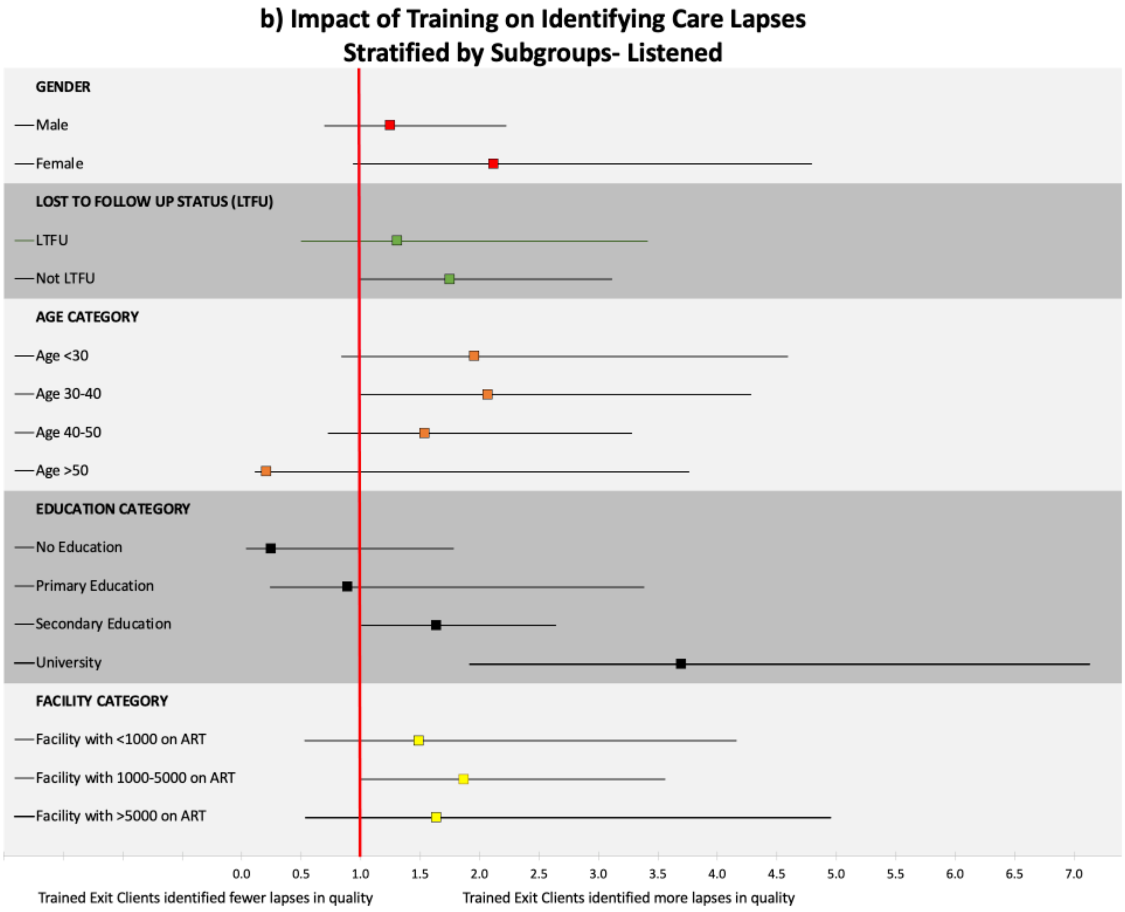

For peer review only - http://bmjopen.bmj.com/site/about/guidelines.xhtml

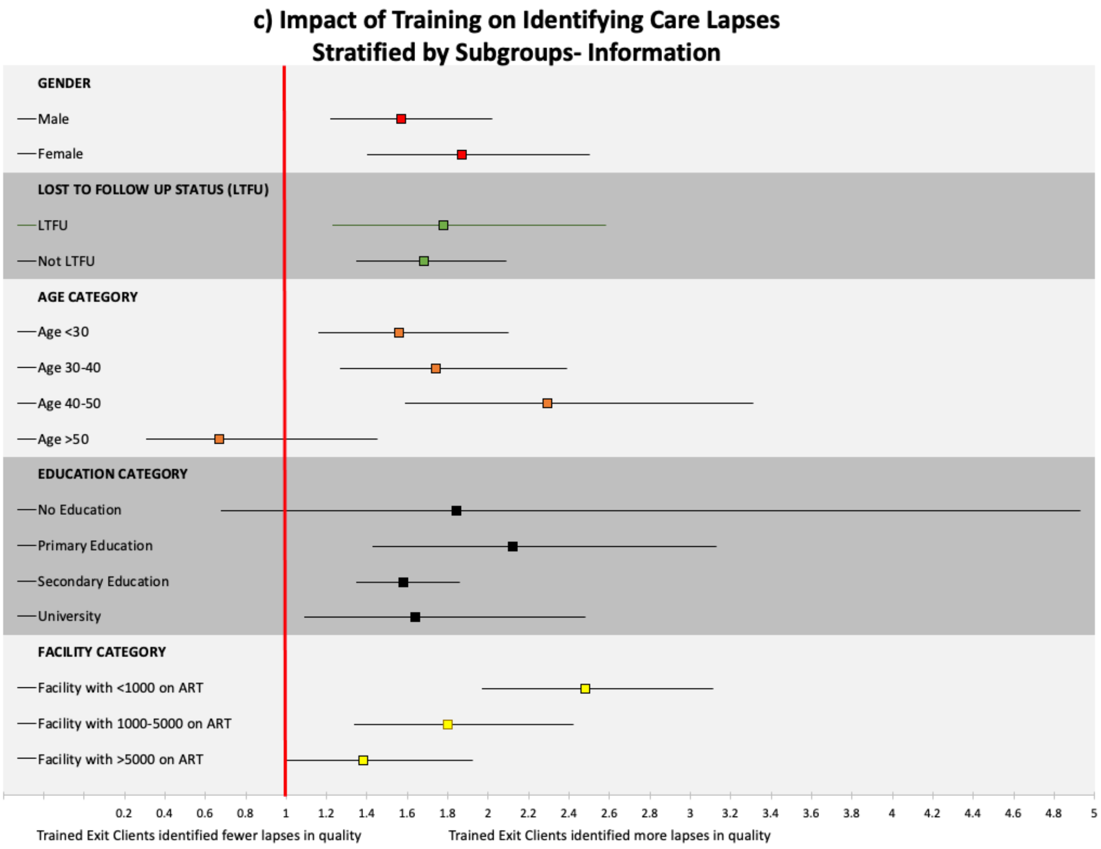

For peer review only

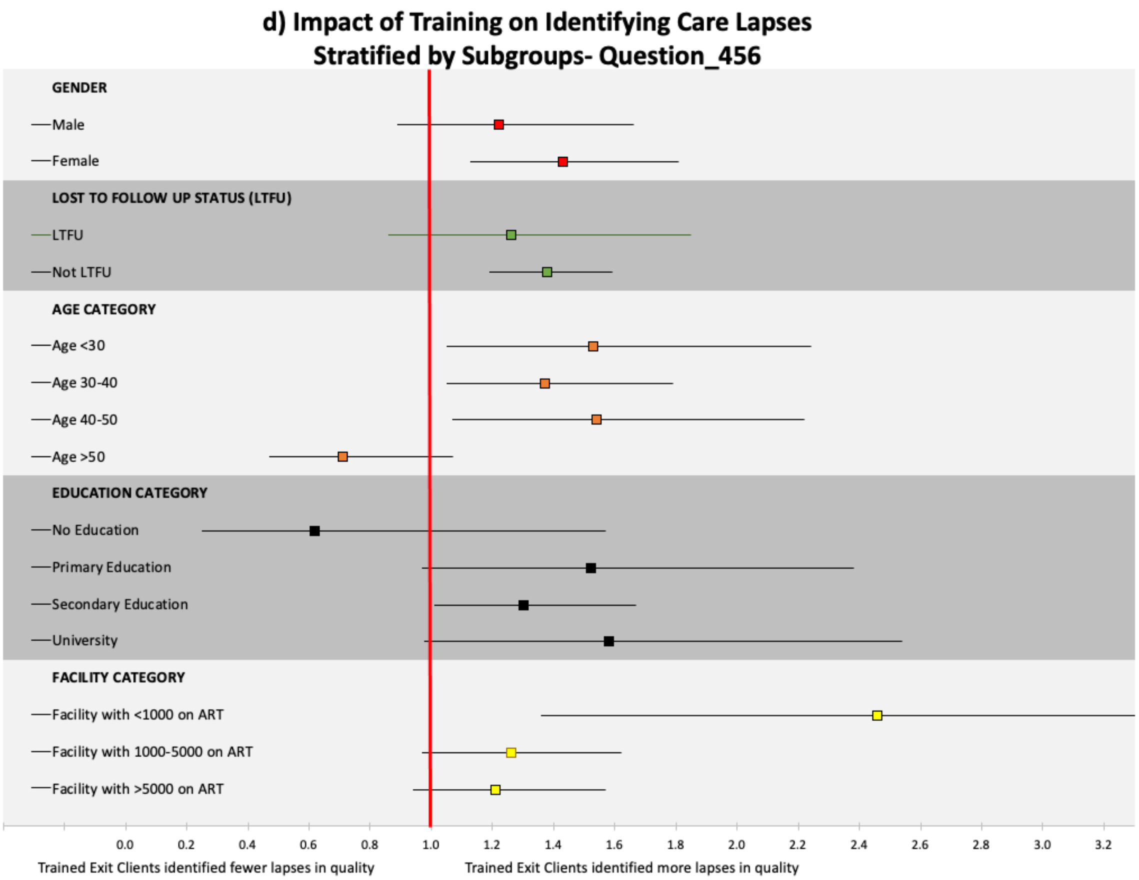
d) Impact of Training on Identifying Care Lapses
Stratified by Subgroups- Question_456
GENDER
Male
Female
LOST TO FOLLOW UP STATUS (LTFU)
LTFU
Not LTFU
AGE CATEGORY
Age <30
Age 30-40
Age 40-50
Age >50
EDUCATION CATEGORY
No Education
Primary Education
Secondary Education
University
FACILITY CATEGORY
Facility with <1000 on ART
Facility with 1000-5000 on ART
Facility with >5000 on ART
0.0 0.2 0.4 0.6 0.8 1.0 1.2 1.4 1.6 1.8 2.0 2.2 2.4 2.6 2.8 3.0 3.2
Trained Exit Clients identified fewer lapses in quality
Trained Exit Clients identified more lapses in quality

view only

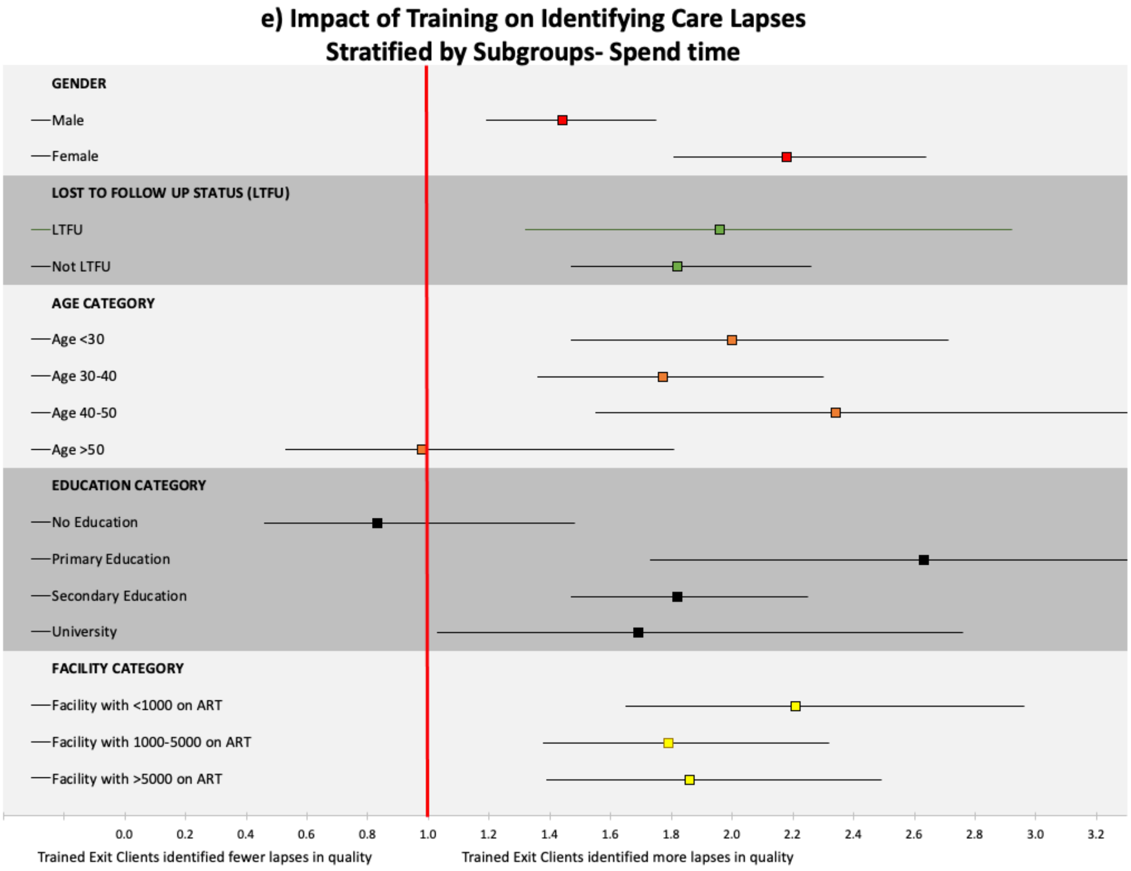
e) Impact of Training on Identifying Care Lapses
Stratified by Subgroups- Spend time
GENDER
Male
Female
LOST TO FOLLOW UP STATUS (LTFU)
LTFU
Not LTFU
AGE CATEGORY
Age <30
Age 30-40
Age 40-50
Age >50
EDUCATION CATEGORY
No Education
Primary Education
Secondary Education
University
FACILITY CATEGORY
Facility with <1000 on ART
Facility with 1000-5000 on ART
Facility with >5000 on ART
0.0
0.2
0.4
0.6
0.8
1.0
1.2
1.4
1.6
1.8
2.0
2.2
2.4
2.6
2.8
3.0
3.2
Trained Exit Clients identified fewer lapses in quality
Trained Exit Clients identified more lapses in quality

f) Impact of Training on Identifying Care Lapses Stratified by Subgroups- Feel about care

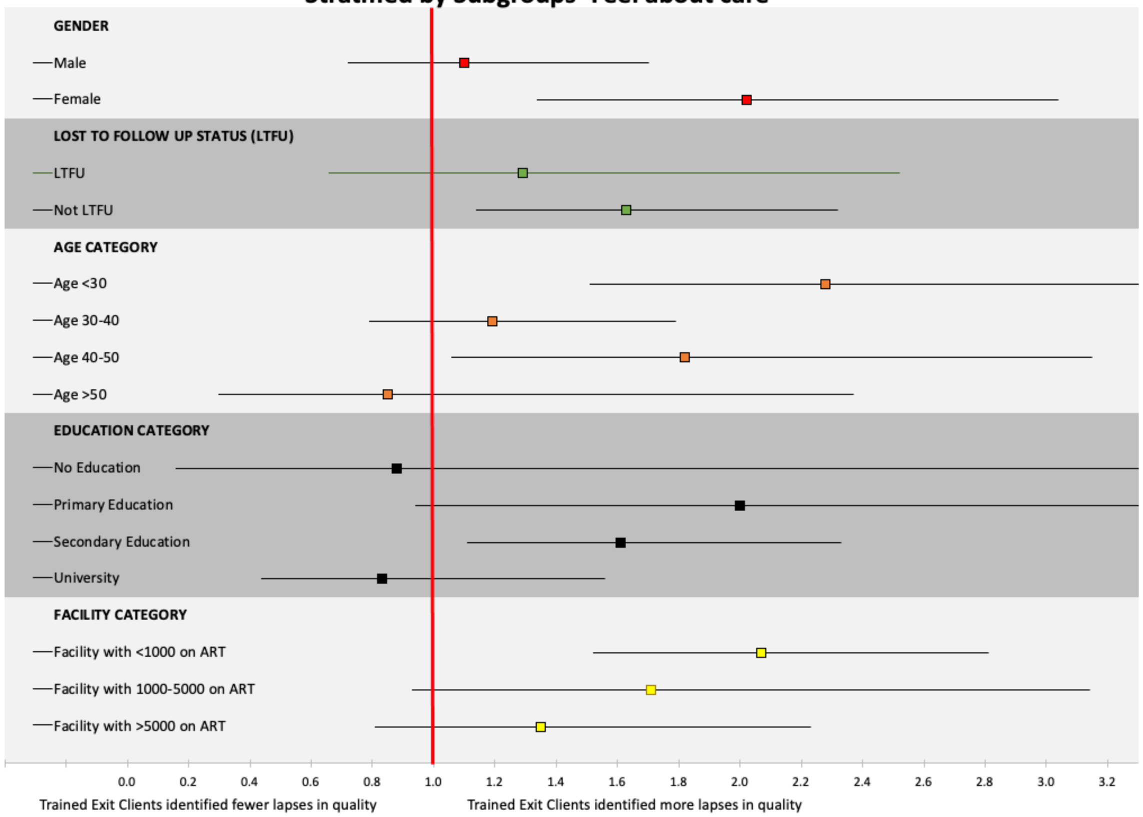

For peer review only - http://bmjopen.bmj.com/site/about/guidelines.xhtml

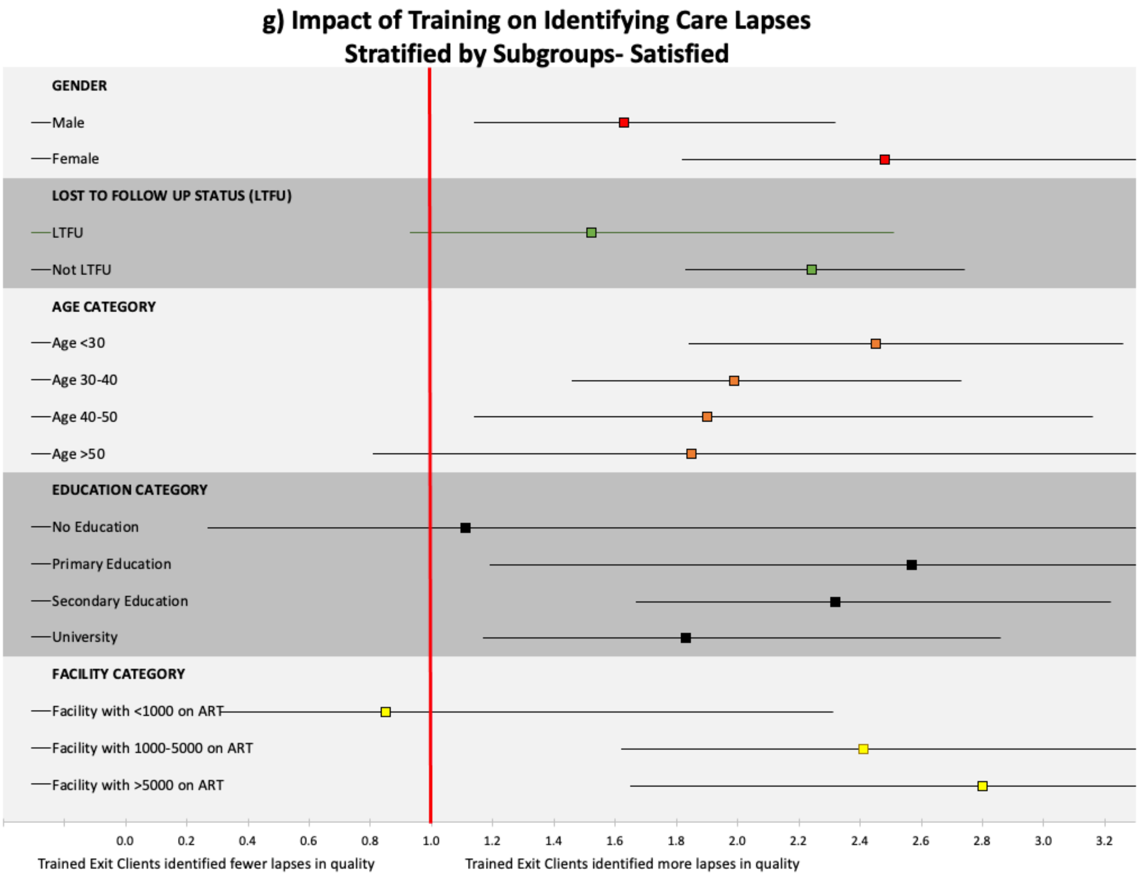
g) Impact of Training on Identifying Care Lapses
Stratified by Subgroups- Satisfied
GENDER
Male
Female
LOST TO FOLLOW UP STATUS (LTFU)
LTFU
Not LTFU
AGE CATEGORY
Age <30
Age 30-40
Age 40-50
Age >50
EDUCATION CATEGORY
No Education
Primary Education
Secondary Education
University
FACILITY CATEGORY
Facility with <1000 on ART
Facility with 1000-5000 on ART
Facility with >5000 on ART
0.0
0.2
0.4
0.6
0.8
1.0
1.2
1.4
1.6
1.8
2.0
2.2
2.4
2.6
2.8
3.0
3.2
Trained Exit Clients identified fewer lapses in quality
Trained Exit Clients identified more lapses in quality

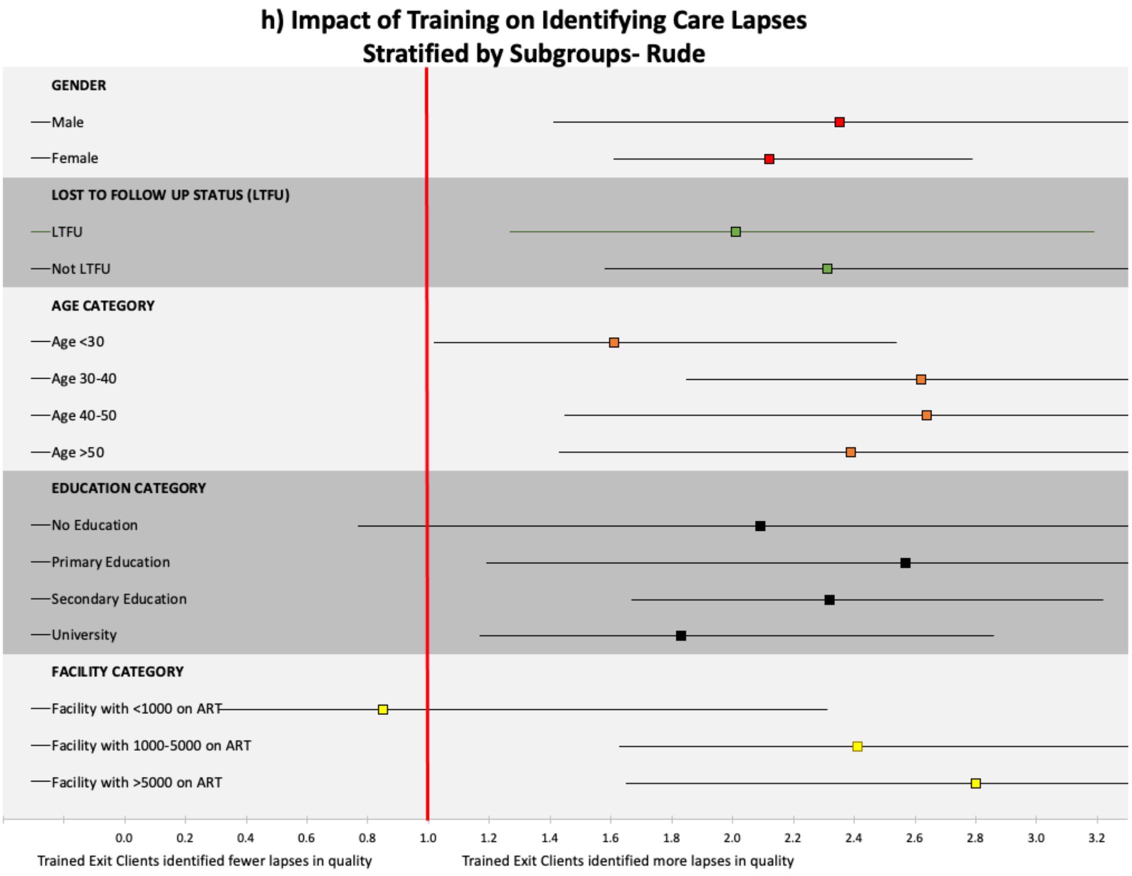
h) Impact of Training on Identifying Care Lapses
Stratified by Subgroups- Rude
GENDER
Male
Female
LOST TO FOLLOW UP STATUS (LTFU)
LTFU
Not LTFU
AGE CATEGORY
Age <30
Age 30-40
Age 40-50
Age >50
EDUCATION CATEGORY
No Education
Primary Education
Secondary Education
University
FACILITY CATEGORY
Facility with <1000 on ART
Facility with 1000-5000 on ART
Facility with >5000 on ART
0.0 0.2 0.4 0.6 0.8 1.0 1.2 1.4 1.6 1.8 2.0 2.2 2.4 2.6 2.8 3.0 3.2
Trained Exit Clients identified fewer lapses in quality
Trained Exit Clients identified more lapses in quality

view only

i) Impact of Training on Identifying Care Lapses Stratified by Subgroups- Lab results

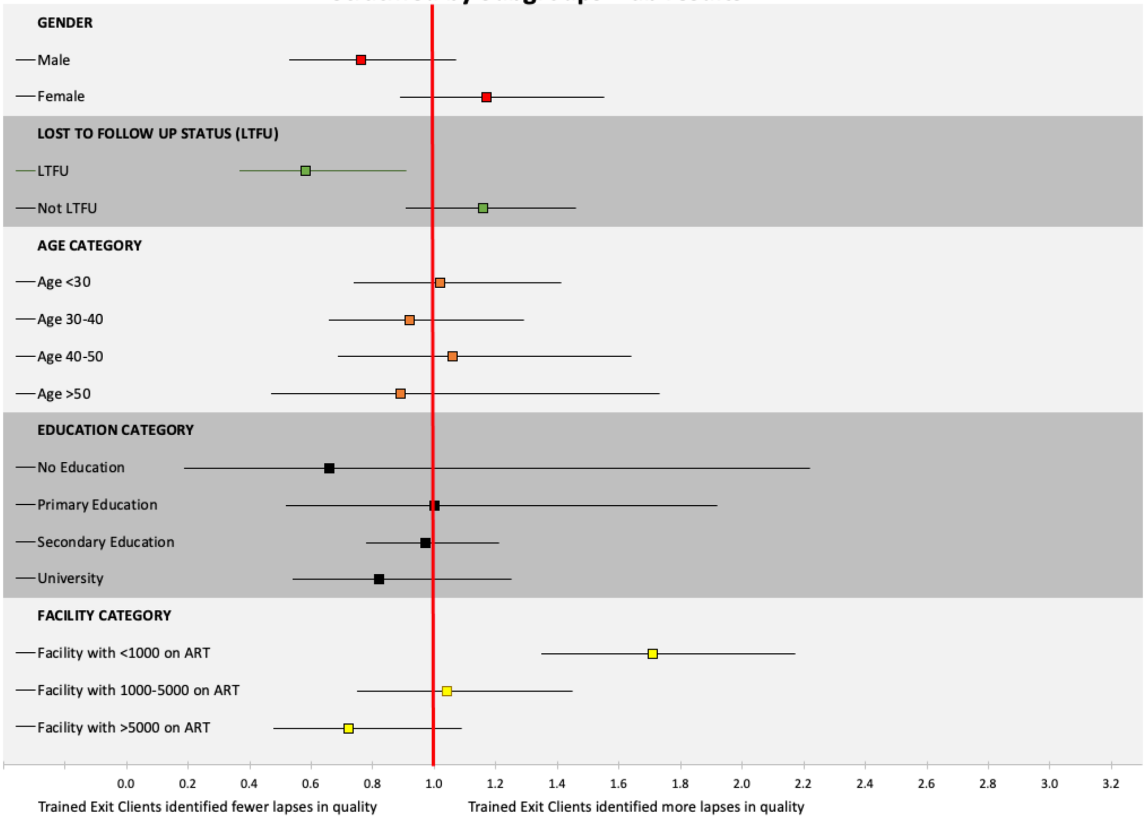

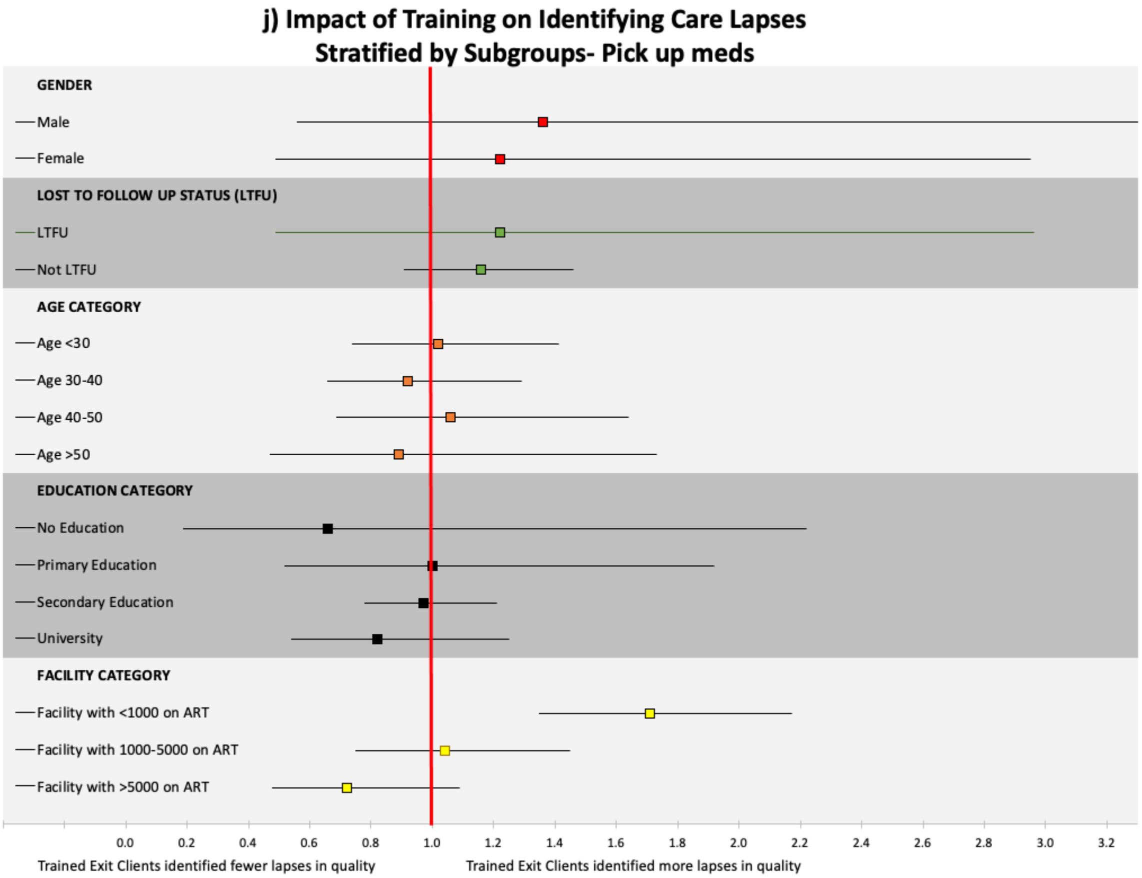
j) Impact of Training on Identifying Care Lapses
Stratified by Subgroups- Pick up meds
GENDER
Male
Female
LOST TO FOLLOW UP STATUS (LTFU)
LTFU
Not LTFU
AGE CATEGORY
Age <30
Age 30-40
Age 40-50
Age >50
EDUCATION CATEGORY
No Education
Primary Education
Secondary Education
University
FACILITY CATEGORY
Facility with <1000 on ART
Facility with 1000-5000 on ART
Facility with >5000 on ART
0.0 0.2 0.4 0.6 0.8 1.0 1.2 1.4 1.6 1.8 2.0 2.2 2.4 2.6 2.8 3.0 3.2
Trained Exit Clients identified fewer lapses in quality
Trained Exit Clients identified more lapses in quality

Supplementary Figure 2

Supplementary Figure 2. Bubble plot showing Trained Exit Sum Score vs Untrained Exit Sum Score. Each bubble represents a single facilities performance. Each bubble's size indicates the number of patients at each facility with larger bubbles corresponding to larger facilities. The horizontal position notes the Untrained Exit Sum Score for all questions against the facility, and the vertical position notes the Trained Exit sum score at the same facility.

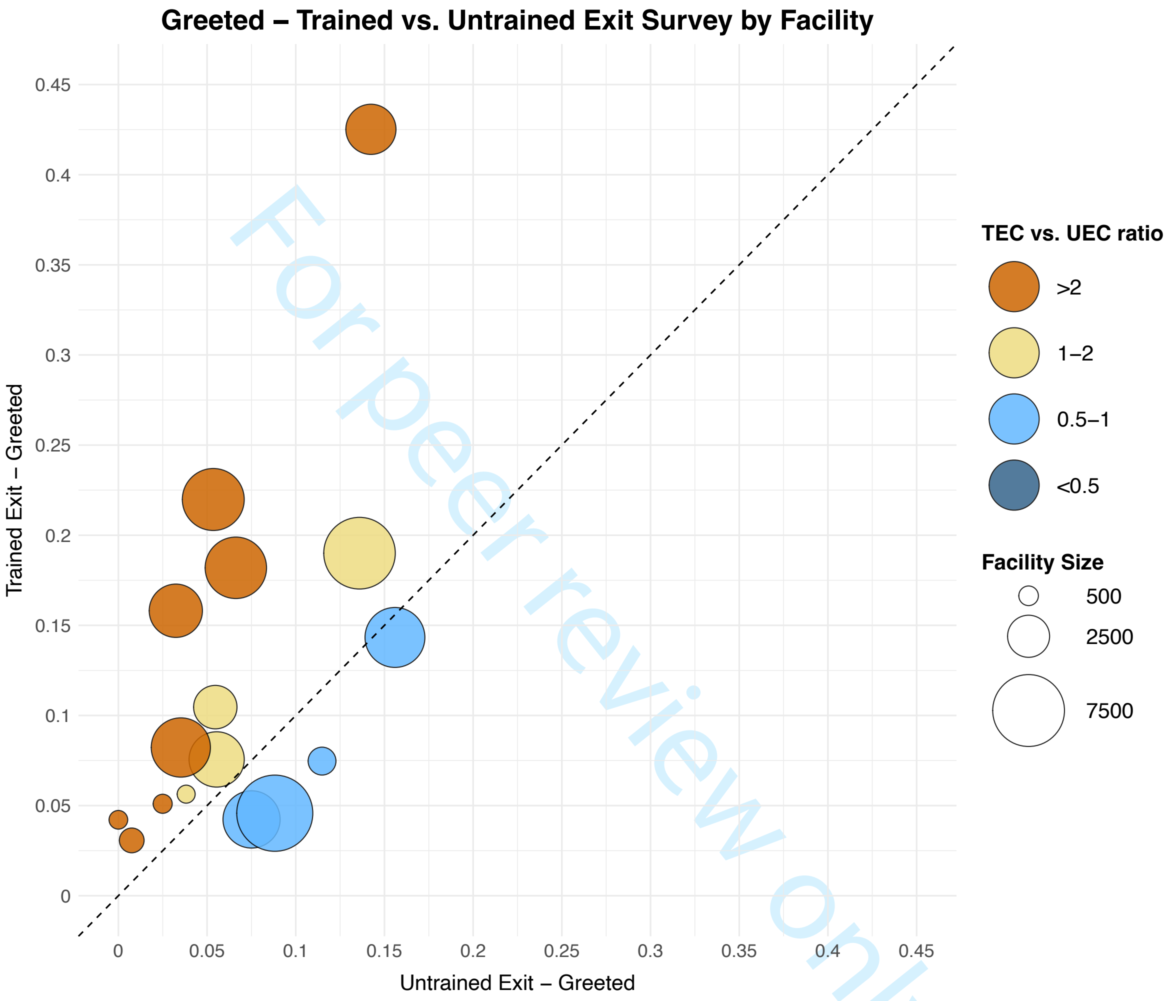

For peer review only - http://bmjopen.bmj.com/site/about/guidelines.xhtml

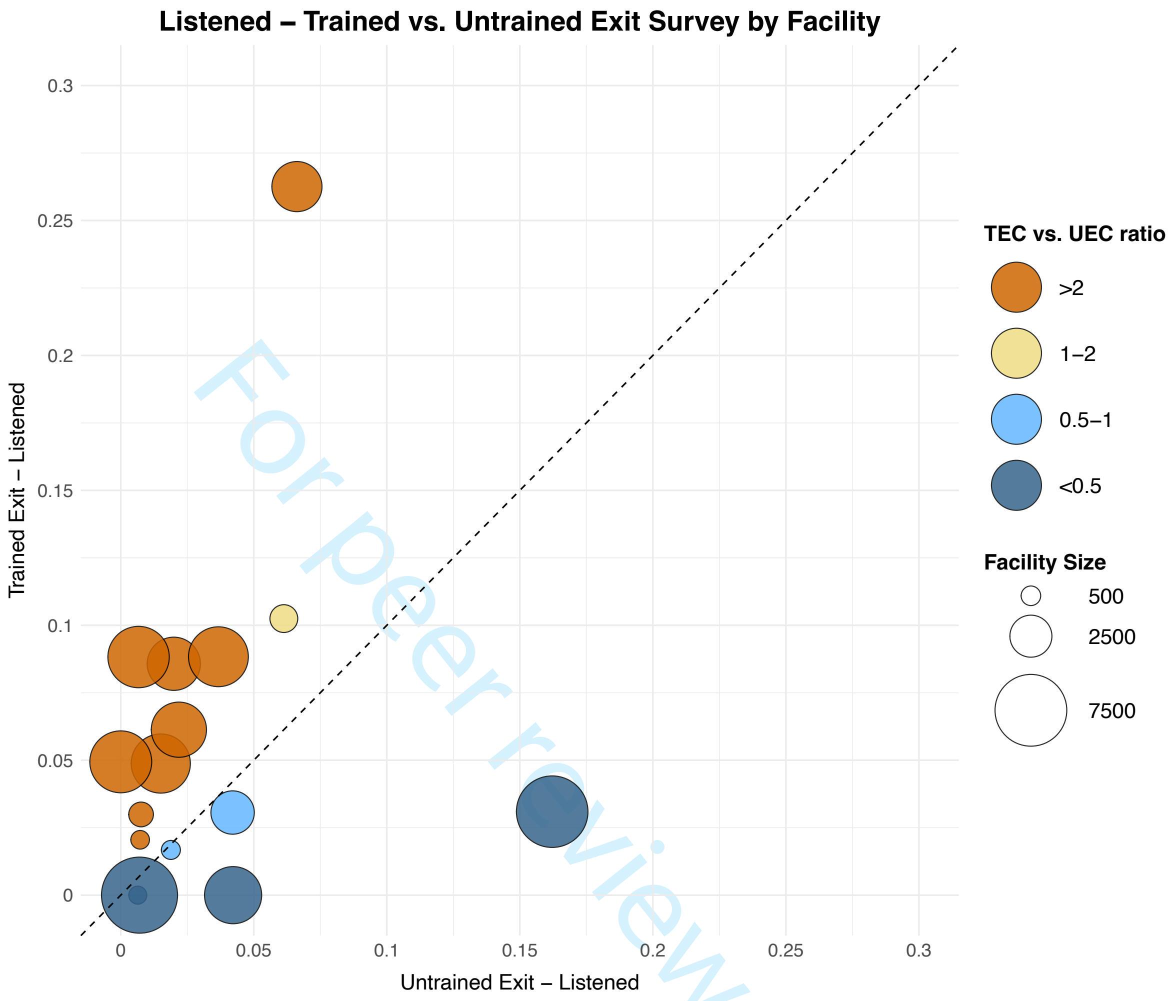

For peer review only

For peer review only - http://bmjopen.bmj.com/site/about/guidelines.xhtml

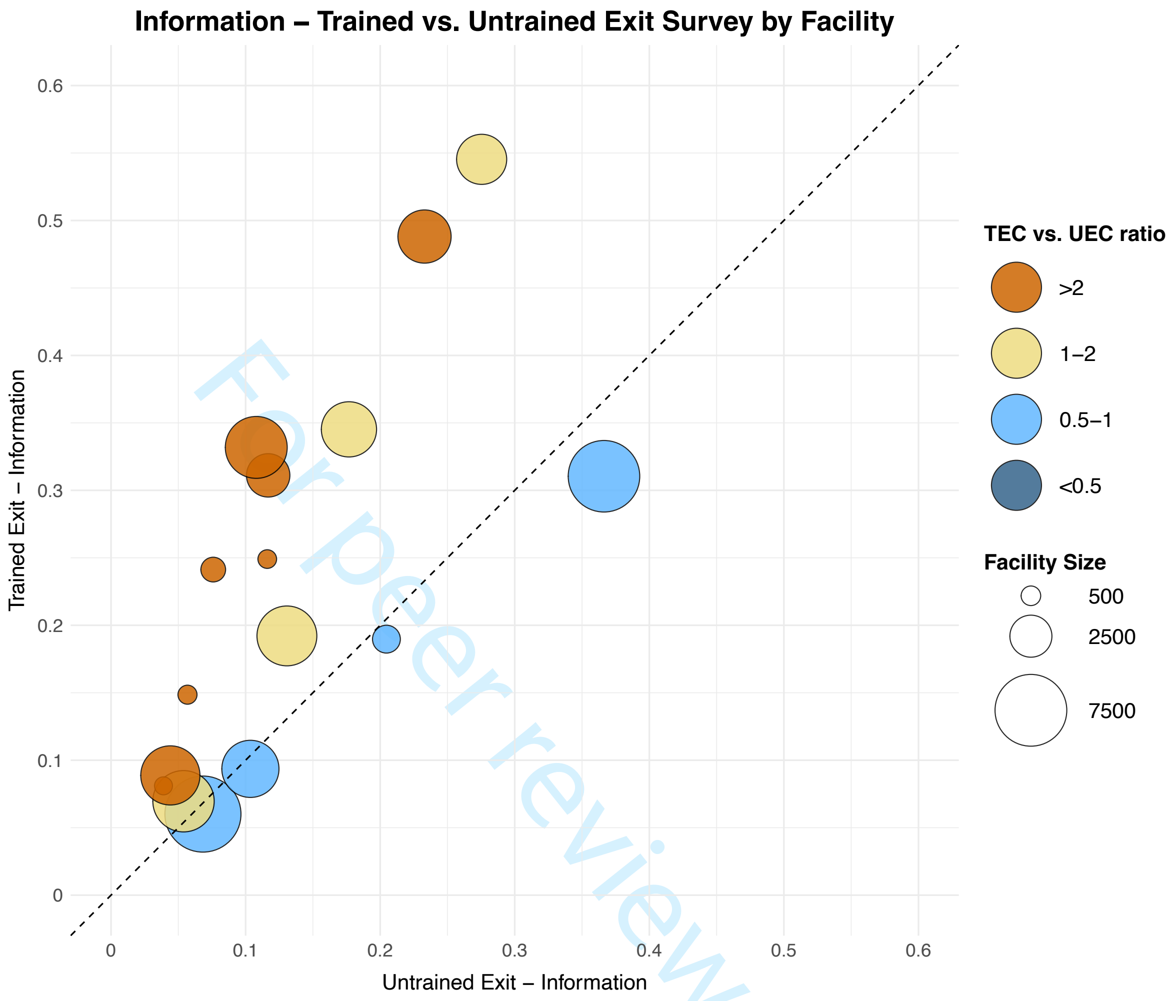

For peer review only - http://bmjopen.bmj.com/site/about/guidelines.xhtml

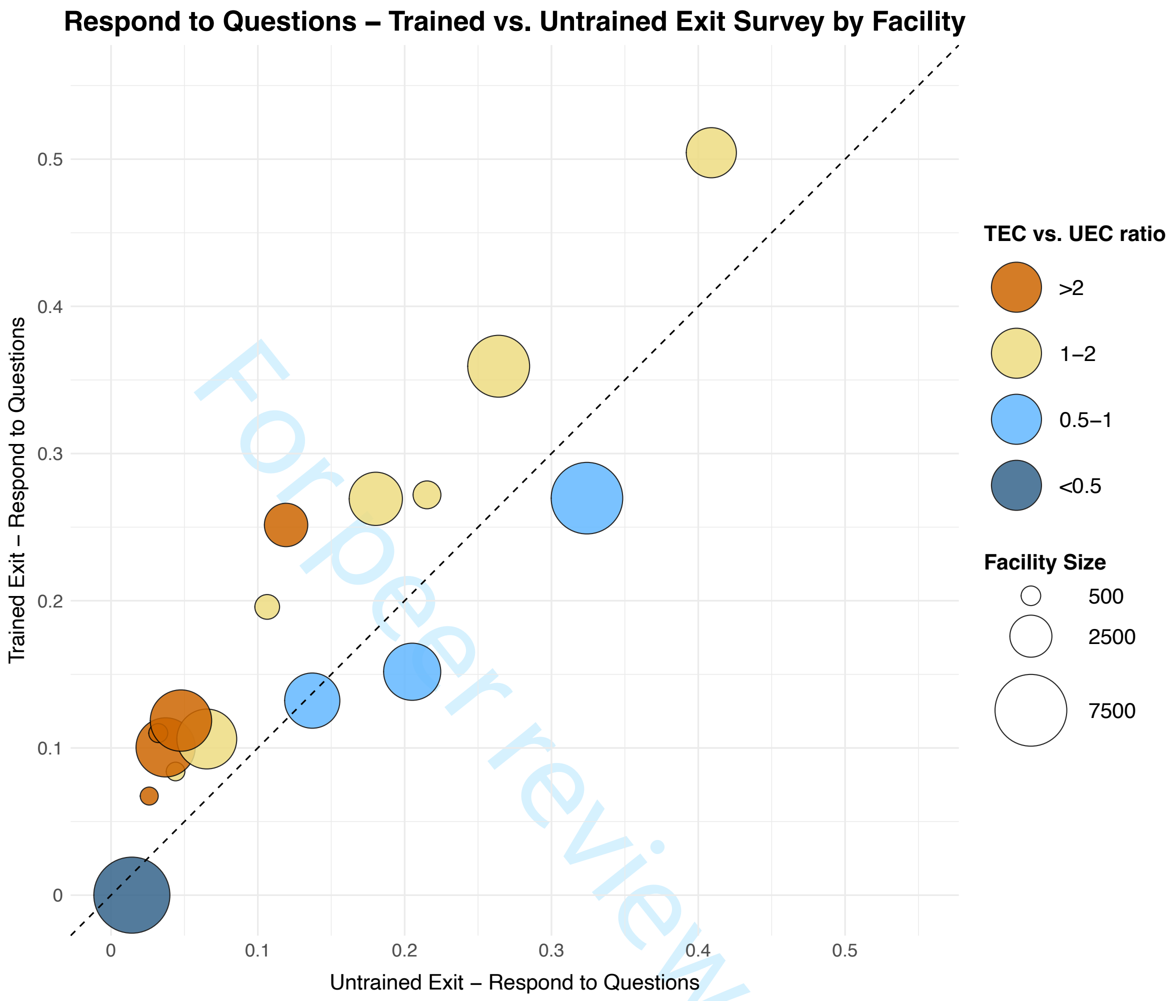

For peer review only - http://bmjopen.bmj.com/site/about/guidelines.xhtml

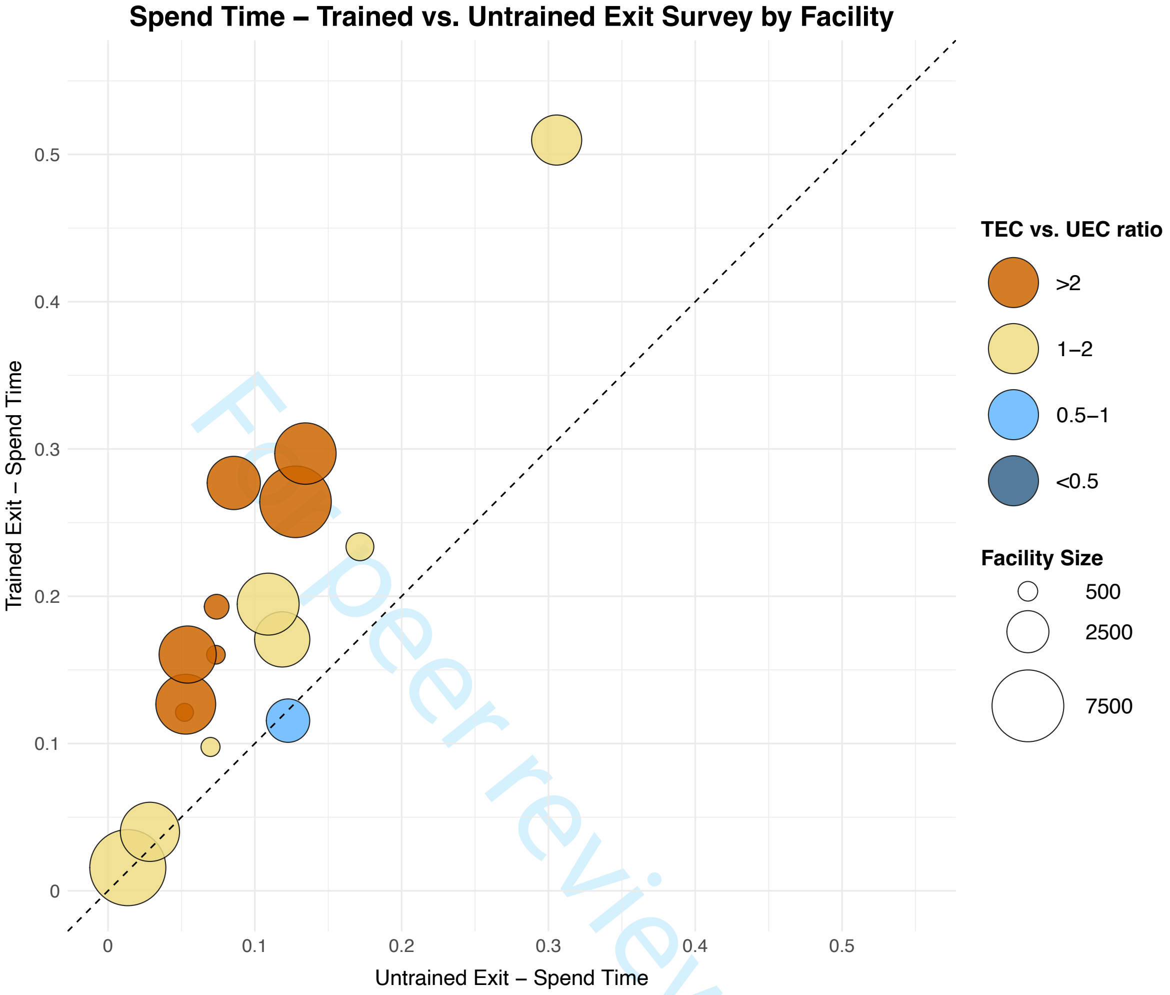

For peer review only

For peer review only - http://bmjopen.bmj.com/site/about/guidelines.xhtml

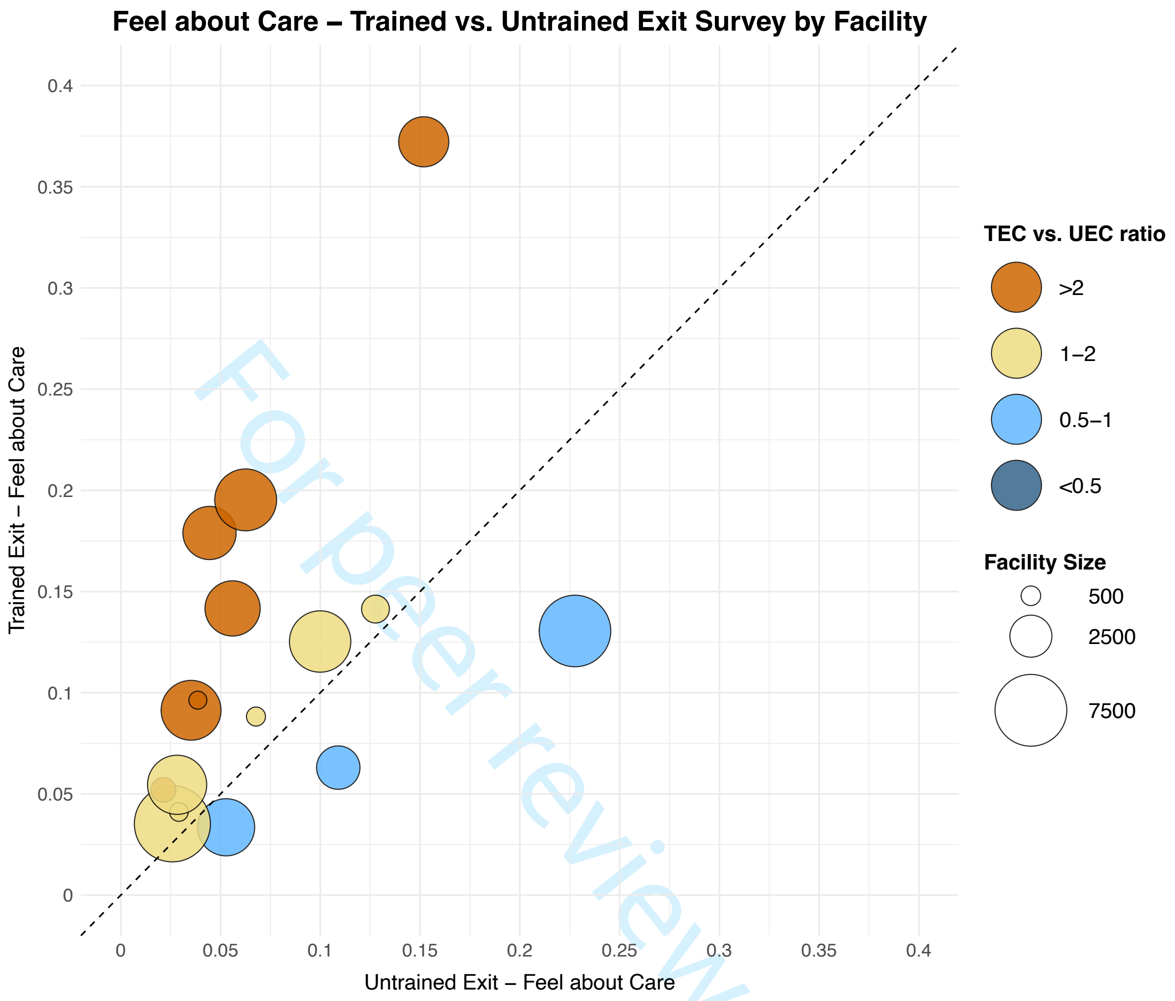

For peer review only - http://bmjopen.bmj.com/site/about/guidelines.xhtml

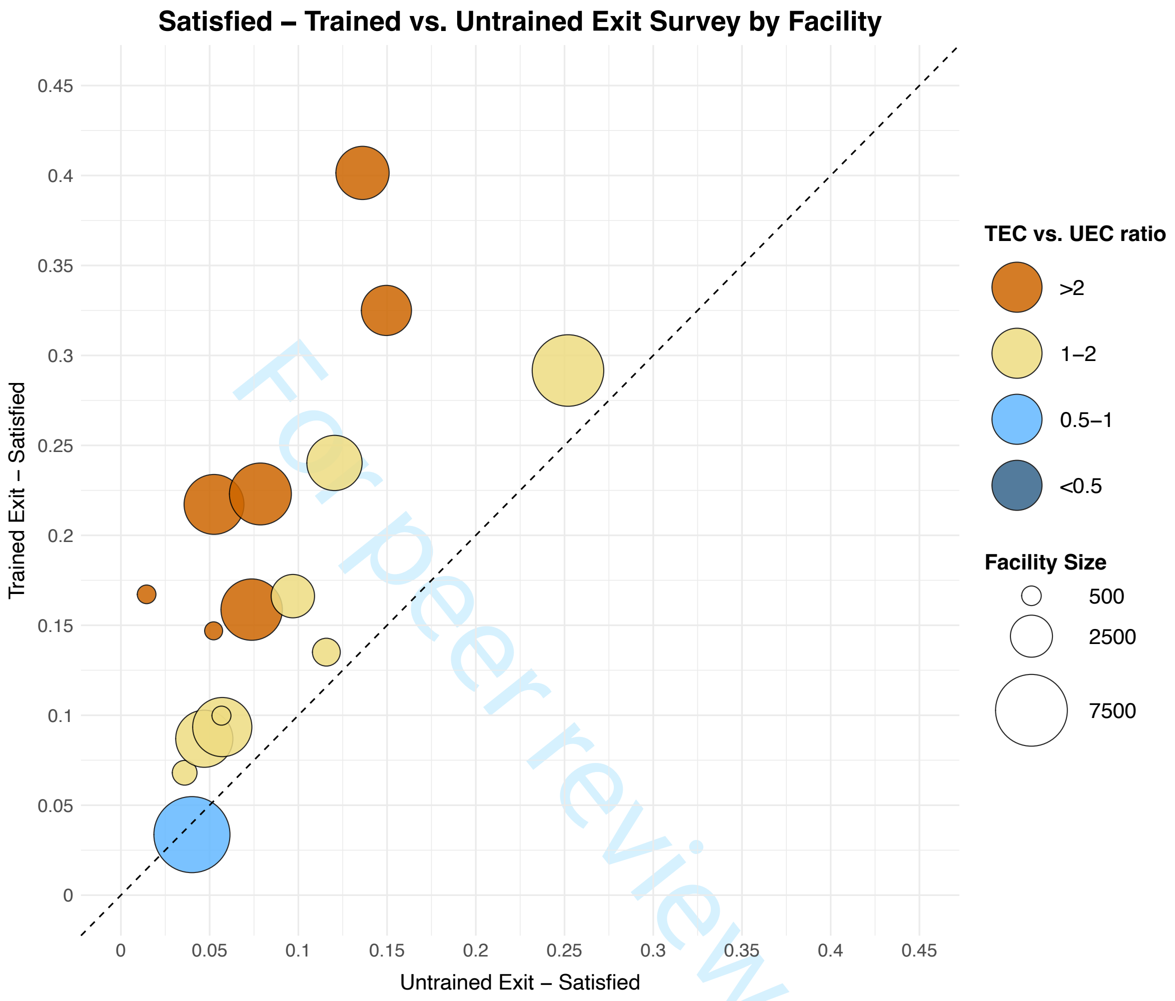

For peer review only - http://bmjopen.bmj.com/site/about/guidelines.xhtml

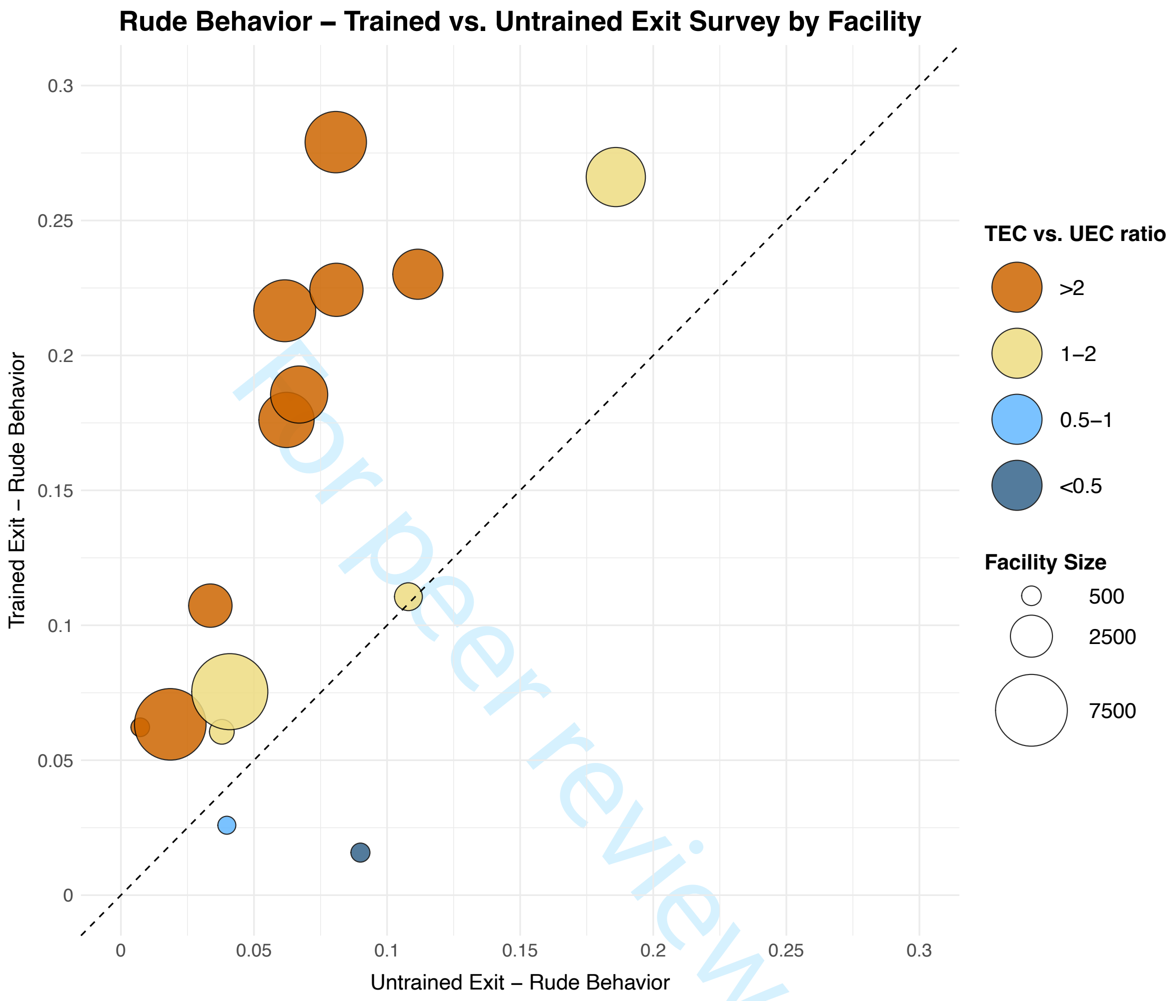

For peer review only

For peer review only - http://bmjopen.bmj.com/site/about/guidelines.xhtml

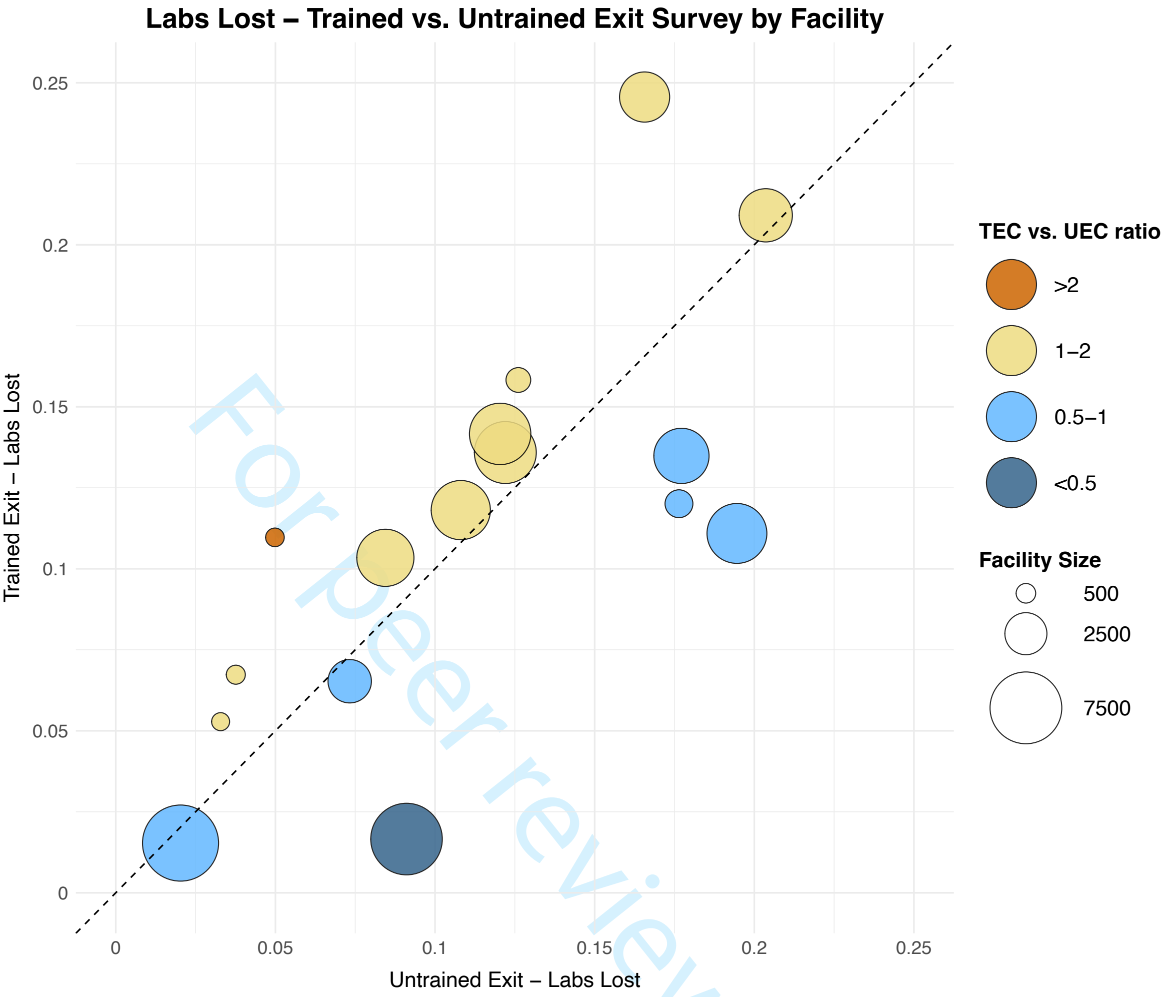

For peer review only

For peer review only - http://bmjopen.bmj.com/site/about/guidelines.xhtml

STROBE Statement—checklist of items that should be included in reports of observational studies

	Item No	Recommendation	Page No
**Title and abstract**	1	(*a*) Indicate the study's design with a commonly used term in the title or the abstract	1
		(*b*) Provide in the abstract an informative and balanced summary of what was done and what was found	1
**Introduction**			
Background/rationale	2	Explain the scientific background and rationale for the investigation being reported	3,4
Objectives	3	State specific objectives, including any prespecified hypotheses	3
**Methods**			
Study design	4	Present key elements of study design early in the paper	4,5
Setting	5	Describe the setting, locations, and relevant dates, including periods of recruitment, exposure, follow-up, and data collection	5
Participants	6	(*a*) *Cohort study*—Give the eligibility criteria, and the sources and methods of selection of participants. Describe methods of follow-up *Case-control study*—Give the eligibility criteria, and the sources and methods of case ascertainment and control selection. Give the rationale for the choice of cases and controls *Cross-sectional study*—Give the eligibility criteria, and the sources and methods of selection of participants	5,6
		(*b*) *Cohort study*—For matched studies, give matching criteria and number of exposed and unexposed *Case-control study*—For matched studies, give matching criteria and the number of controls per case	N/A
Variables	7	Clearly define all outcomes, exposures, predictors, potential confounders, and effect modifiers. Give diagnostic criteria, if applicable	5,7,8
Data sources/ measurement	8*	For each variable of interest, give sources of data and details of methods of assessment (measurement). Describe comparability of assessment methods if there is more than one group	*5-7*
Bias	9	Describe any efforts to address potential sources of bias	4
Study size	10	Explain how the study size was arrived at	8
Quantitative variables	11	Explain how quantitative variables were handled in the analyses. If applicable, describe which groupings were chosen and why	7
Statistical methods	12	(*a*) Describe all statistical methods, including those used to control for confounding	7
		(*b*) Describe any methods used to examine subgroups and interactions	7
		(*c*) Explain how missing data were addressed	N/A
		(*d*) *Cohort study*—If applicable, explain how loss to follow-up was addressed *Case-control study*—If applicable, explain how matching of cases and controls was addressed *Cross-sectional study*—If applicable, describe analytical methods taking account of sampling strategy	N/A
		(*e*) Describe any sensitivity analyses	N/A

Continued on next page

For peer review only - http://bmjopen.bmj.com/site/about/guidelines.xhtml

**Results**			
Participants	13*	(a) Report numbers of individuals at each stage of study—eg numbers potentially eligible, examined for eligibility, confirmed eligible, included in the study, completing follow-up, and analysed	9
		(b) Give reasons for non-participation at each stage	9
		(c) Consider use of a flow diagram	N/A
Descriptive data	14*	(a) Give characteristics of study participants (eg demographic, clinical, social) and information on exposures and potential confounders	9, 10
		(b) Indicate number of participants with missing data for each variable of interest	9,10
		(c) *Cohort study*—Summarise follow-up time (eg, average and total amount)	N/A
Outcome data	15*	*Cohort study*—Report numbers of outcome events or summary measures over time	N/A
		Case-control study—Report numbers in each exposure category, or summary measures of exposure	N/A
		Cross-sectional study—Report numbers of outcome events or summary measures	10
Main results	16	(*a*) Give unadjusted estimates and, if applicable, confounder-adjusted estimates and their precision (eg, 95% confidence interval). Make clear which confounders were adjusted for and why they were included	7,11
		(*b*) Report category boundaries when continuous variables were categorized	
		(*c*) If relevant, consider translating estimates of relative risk into absolute risk for a meaningful time period	
Other analyses	17	Report other analyses done—eg analyses of subgroups and interactions, and sensitivity analyses	12,14
**Discussion**			
Key results	18	Summarise key results with reference to study objectives	14,15
Limitations	19	Discuss limitations of the study, taking into account sources of potential bias or imprecision. Discuss both direction and magnitude of any potential bias	18
Interpretation	20	Give a cautious overall interpretation of results considering objectives, limitations, multiplicity of analyses, results from similar studies, and other relevant evidence	19,20
Generalisability	21	Discuss the generalisability (external validity) of the study results	18
**Other information**			
Funding	22	Give the source of funding and the role of the funders for the present study and, if applicable, for the original study on which the present article is based	20

*Give information separately for cases and controls in case-control studies and, if applicable, for exposed and unexposed groups in cohort and cross-sectional studies.

Note: An Explanation and Elaboration article discusses each checklist item and gives methodological background and published examples of transparent reporting. The STROBE checklist is best used in conjunction with this article (freely available on the Web sites of PLoS Medicine at http://www.plosmedicine.org/, Annals of Internal Medicine at http://www.annals.org/, and Epidemiology at http://www.epidem.com/). Information on the STROBE Initiative is available at www.strobe-statement.org.

For peer review only

For peer review only - http://bmjopen.bmj.com/site/about/guidelines.xhtml

BMJ Open

A Comparison of Patient Exit Interviews to Unannounced Standardised Patients for Assessing HIV Service Delivery in Zambia

Journal:	*BMJ Open*
Manuscript ID	bmjopen-2022-069086.R1
Article Type:	Original research
Date Submitted by the Author:	13-Mar-2023
Complete List of Authors:	Sikombe, Kombatende; Center for Infectious Disease Research in Zambia, Implementation Science Unit; London School of Hygiene and Tropical Medicine Faculty of Public Health and Policy, Department of Public Health, Environments and Society<br>Pry, Jake; Center for Infectious Disease Research in Zambia, Implementation Science Unit<br>Mody, Aaloke; Washington University in St Louis School of Medicine, Internal Medicine<br>Rice, Brian ; London School of Hygiene and Tropical Medicine Faculty of Public Health and Policy, Department of Public Health, Environments and Society<br>Bukankala, Chama; Center for Infectious Disease Research in Zambia, Research<br>Eshun-Wilson, Ingrid ; Washington University in St Louis School of Medicine, Internal Medicine<br>Mutale, Jacob; Center for Infectious Disease Research in Zambia, Implementation Science Unit<br>Simbeza, Sandra; Center for Infectious Disease Research in Zambia, Implementation Science Unit<br>Beres, Laura K ; Johns Hopkins University Bloomberg School of Public Health, Department of International Health<br>Mukamba, Njekwa; Center for Infectious Disease Research in Zambia, Social and Behavioural Science Research Group<br>Mukumbwa-Mwenechanya, Mpande; Center for Infectious Disease Research in Zambia, Implementation Science Unit<br>Mwamba, Daniel; Center for Infectious Disease Research in Zambia, Clinical Care Services<br>Sharma, Anjali; Center for Infectious Disease Research in Zambia, Social and Behavioural Science Research Group<br>Wringe, Alison; London School of Hygiene and Tropical Medicine Faculty of Epidemiology and Population Health<br>Hargreaves, James; London School of Hygiene and Tropical Medicine Faculty of Public Health and Policy, Department of Public Health, Environments and Society<br>Bolton-Moore, Carolyn; Center for Infectious Disease Research in Zambia, Clinical Care Services ; The University of Alabama at Birmingham Department of Medicine, Infectious Disease<br>Holmes, Charles; Georgetown University Medical Center, Center for Innovation in Global Health

	Sikazwe, Izukanji; Center for Infectious Disease Research in Zambia, Implementation Science Unit<br>Geng, Elvin; Washington University in St Louis School of Medicine, Internal Medicine
**Primary Subject Heading**:	Health services research
Secondary Subject Heading:	Epidemiology, Global health, Health services research, HIV/AIDS, Patient-centred medicine
Keywords:	Public health < INFECTIOUS DISEASES, Organisation of health services < HEALTH SERVICES ADMINISTRATION & MANAGEMENT, Quality in health care < HEALTH SERVICES ADMINISTRATION & MANAGEMENT, HIV & AIDS < INFECTIOUS DISEASES, PUBLIC HEALTH

SCHOLARONE™ Manuscripts

I, the Submitting Author has the right to grant and does grant on behalf of all authors of the Work (as defined in the below author licence), an exclusive licence and/or a non-exclusive licence for contributions from authors who are: i) UK Crown employees; ii) where BMJ has agreed a CC-BY licence shall apply, and/or iii) in accordance with the terms applicable for US Federal Government officers or employees acting as part of their official duties; on a worldwide, perpetual, irrevocable, royalty-free basis to BMJ Publishing Group Ltd ("BMJ") its licensees and where the relevant Journal is co-owned by BMJ to the co-owners of the Journal, to publish the Work in this journal and any other BMJ products and to exploit all rights, as set out in our licence.

The Submitting Author accepts and understands that any supply made under these terms is made by BMJ to the Submitting Author unless you are acting as an employee on behalf of your employer or a postgraduate student of an affiliated institution which is paying any applicable article publishing charge ("APC") for Open Access articles. Where the Submitting Author wishes to make the Work available on an Open Access basis (and intends to pay the relevant APC), the terms of reuse of such Open Access shall be governed by a Creative Commons licence – details of these licences and which Creative Commons licence will apply to this Work are set out in our licence referred to above.

Other than as permitted in any relevant BMJ Author's Self Archiving Policies, I confirm this Work has not been accepted for publication elsewhere, is not being considered for publication elsewhere and does not duplicate material already published. I confirm all authors consent to publication of this Work and authorise the granting of this licence.

For peer review only

A Comparison of Patient Exit Interviews to Unannounced Standardised Patients for Assessing HIV Service Delivery in Zambia

Kombatende Sikombe[1,2], Jake M Pry[1,3], Aaloke Mody[4], Brian Rice, Chama Bukankala[1], Ingrid Eshun-Wilson[4], Jacob Mutale[1], Sandra Simbeza[1], Laura K Beres[5], Njekwa Mukamba[1], Mpande Mukumbwa-Mwenechanya[1], Daniel Mwamba[1], Anjali Sharma[1], Alison Wringe[6], James Hargreaves[1], Carolyn Bolton Moore[1,7], Charles B Holmes[8], Izukanji Sikazwe[1], Elvin Geng[4]

[1]Centre for Infectious Disease Research in Zambia, Lusaka, Zambia, [2]Faculty of Public Health and Policy, London School of Hygiene & Tropical Medicine, London, UK, [3]Department of Public Health Sciences, University of California, Davis, CA, USA, [4]Division of Infectious Diseases, Washington University in St Louis, St. Louis, MO, USA, [5]Department of International Health, Johns Hopkins Bloomberg School of Public Health, Baltimore, MD, USA, [6]Faculty of Epidemiology and Population Health, London School of Hygiene and Tropical Medicine, London, UK, [7]Department of Medicine, School of Medicine, University of Alabama at Birmingham, Birmingham, AL, USA, [8]Center for Innovation in Global Health, Georgetown University, Washington, DC, USA.

Corresponding Author: Kombatende Sikombe
Affiliations: Centre for Infectious Disease Research in Zambia, Lusaka, Zambia, Faculty of Public Health and Policy, London School of Hygiene & Tropical Medicine, London, UK
Email Address: kombatende.sikombe@cidrz.org kombatende.sikombe@lshtm.ac.uk

For peer review only

ABSTRACT

Objectives: To compare the use of an unannounced standardised patient approach (e.g., mystery clients) to typical exit interviews for assessing patient experiences in HIV care (e.g., unfriendly interactions with providers, long-waiting times). We hypothesize standardized patients would report more negative experiences than typical exit interviews affected by social desirability bias.

Setting: Cross-sectional surveys in 16 government-operated HIV primary care clinics in Lusaka, Zambia providing antiretroviral-therapy (ART).

Participants: 3526 participants aged ≥18 years receiving ART participated in the exit surveys between August 2019 and November 2021.

Intervention: Systematic sample (every k^{th} file) of patients in clinic waiting area willing to be trained received pre-visit training and post-visit interviews. Providers were unaware of trained patients.

Outcome measures: We compared patient experience among patients that received a brief training prior to their care visit (explaining each patient experience construct in the exit survey, being anonymous, without manipulating behaviour) with those who did not undergo training on the survey prior to their visit.

Results: Among 3526 participants who participated in exit surveys, 2415 were untrained (56% female, median age 40 (IQR:32-47)) and 1111 were trained (50% female, median age 37 (IQR:31-45)). Compared to untrained, trained patients were more likely to report a negative care experience overall (adjusted Prevalence Ratio aPR for aggregate sum score: 1.64 [95% CI:1.39-1.94]), with a greater proportion reporting feeling unwelcomed by providers ([aPR]: 1.71 [95% CI:1.20-2.44]) and witnessing providers behaving rude (aPR: 2.28 [95% CI:1.63-3.19]).

Conclusion: Trained patients were more likely to identify sub-optimal care. They may have understood the items solicited better or felt empowered to be more critical., We trained existing patients, unlike studies that use “standardised patients” drawn from outside the patient population. This low-cost strategy could improve patient-centred service delivery elsewhere.

Trial registration: Assessment was nested within a parent study. www.pactr.org registered the parent study (PACTR202101847907585).

For peer review only

STRENGTHS AND LIMITATIONS OF THIS STUDY

- We demonstrate feasibility of a standardised patient (SP) approach designed to assess chronic care in which real, not simulated, patients are trained before their upcoming encounters.
- Traditional SP techniques require a trained simulated patient to visit multiple clinics, a strategy more appropriate for episodic care rather than chronic care.
- Modified SP approaches can address these rigorous and pragmatic challenges of integrating patient experience into chronic routine public health- a crucial indicator of quality for governments and funders.
- We trained patients to evaluate quality of care (e.g., waiting times, rude providers), and compared their responses to traditional untrained exit surveys in 16 facilities in Zambia.
- How to train remains a challenge as we did not recruit participants who were not able to read and write, perceived to have bad recall ability and potentially unable to comprehend.

For peer review only

BACKGROUND

Because of improved testing, linkage, and treatment to meet the global 95-95-95 treatment targets (95% of HIV-positive patients know their status, 95% are on treatment, and 95% have suppressed viral loads) [1], retention in care have become a major obstacle to improving Human Immunodeficiency Virus (HIV) treatment outcomes, and health systems in low-income settings like Zambia, have sought to shift their public health response by designing and delivering high quality and patient-centred HIV care [2–7]. Efforts to improve service quality and patient experience require systematic measurement of the patient experience to guide facility responses as poor patient experience has been shown to lead to disengagement from care [8–12]. Health policymakers and donors, such as the President's Emergency Plan for AIDS Relief (PEPFAR), have invested in clinical metrics to assess care quality in Zambia and the wider region, but to a lesser extent in non-clinical metrics like patient experience [13]. These metrics can be critical for guiding efforts to improve retention in care by ensuring an informed response to improving quality of care and patient centredness.

Accurate and pragmatic measurement of the patient experience poses a range of challenges. Patient experience exit surveys are prone to social desirability bias because of power dynamics in health care. Empirical studies of satisfaction, for example, are widely believed to over-estimate patient satisfaction [14]. This may be particularly true where provider-patient relationships are traditional and hierarchical. Delaying surveys for some time after the encounter is theorised to ameliorate social desirability bias, but in turn may exacerbate bias due to simple inability to remember — thus creating recall bias [7,15]. Other methods such as direct clinical observations of care pose practical difficulties [14,16]. For example, direct observations may be intrusive and

For peer review only

160 therefore may not reflect everyday functionality of a health facility. Care provided under direct
161 observations may be of higher quality as behaviour may be influenced by observation, a
162 phenomenon often known as the "Hawthorne effect" [14,16].

163 Standardised patients (SP), also known as "mystery clients" or "simulated patients" have
164 largely been used to assess quality of care in developed countries, as well as in assessing customer
165 service in the retail industry[17]. SP can be resource-intensive and require training, but reduce
166 potential for recall bias, social desirability, bias, and Hawthorne effects, providing an opportunity
167 for optimal assessment of patient satisfaction among people receiving HIV care [7,15,18]. They
168 have largely been used for episodic care where a highly skilled and well-trained person poses as a
169 client by making one visit to multiple facilities. This approach holds promise for assessing the
170 patient experience in HIV care but poses pragmatic challenges when assessing the quality of chronic
171 care in which a patient makes multiple visits and may compromise efficiency at, already
172 overburdened, facilities [19–24]. In this study, we report on the development and evaluation of a
173 modified SP approach in which we trained real patients (trained exit clients - TEC) to report on
174 certain characteristics of encounters, and rate key components of care such as waiting times,
175 communication, respectfulness of providers, and privacy.

176 # METHODS

177 **Study design & setting**

178 This study seeks to compare two different methods for assessing patient experience: standard
179 exit survey and those reported by patients who had a brief training on the items before the clinical
180 encounter and to whom the clinic was blinded. The assessment was nested within a parent study:
181 the Leveraging Person-Centred Public Health (PCPH) to improve HIV outcomes in Zambia study

For peer review only

(www.pactr.org PACTR202101847907585), a Stepped Wedge Cluster Randomised Trial that occurred between August 2019 and November 2021. The aim of the overall PCPH study was to assess the impact of introducing health care workers (HCW) to a patient-centred care (PCC) curriculum and mentoring them on PCC principles to improve retention and viral suppression in HIV care. In this nested sub-study, we compared cross sectional surveys of patient experience using two different survey methods: adapted standardised approach (Trained Exit Clients) vs traditional exit surveys.

Population

The sub-study reported here included 16 health facilities in Lusaka, Zambia, operated by the Ministry of Health (MOH) and receiving technical assistance from the Centre for Infectious Diseases Research in Zambia (CIDRZ) - a Zambian non-governmental organisation (NGO) as well as a part of the larger parent study. We surveyed adults aged 18 years and over who were accessing antiretroviral therapy (ART) at study facilities. Exit survey patients were selected in a systematic sample (every k^{th} file varied by facility size) at the time of exit from the clinic. Trained patients were recruited in the waiting room for their visit, underwent a brief training, and then answered survey questions on exit from their encounter. Participants attending an HIV care visit on the day, able to recall events and comprehend study participant recruitment details (as assessed using the comprehension assessment tool) and able to read and write (assessed using literacy tool) were eligible for inclusion.

Procedures and Measurements

Survey Instrument

For peer review only - http://bmjopen.bmj.com/site/about/guidelines.xhtml

For both survey methods, we developed a patient experience instrument based on a previously validated tool developed and used in Kenya: The Wachira Physician-Patient Communication Behaviours Scale [25–27]. This survey assessed elements of patient experience including how they were greeted, communicated to, and overall experience. We included additional questions to capture for example, patient reports of witnessing rude behaviour, receiving appropriate medications and availability of lab results. Prior to use in this study, we performed cognitive interviews among twenty participants to assess consistency in understanding questions in English, Bemba and Nyanja. Surveys were forward and back translated to ensure consistency across the three languages. The survey tools for trained and untrained clients were identical. Research assistants were trained by the first author in recruitment, training and administering of the TEC and UEC survey in all 16 facilities. The provincial and district health management teams were informed about the unannounced TEC survey as well as the UEC survey. The study team sensitised all facility staff at the start of the study, but HCWs were not aware of who specific TECs were.

Procedures for Trained and Untrained Exit Clients

Efforts to "standardise" assessment of the quality and nature of care in HIV care differs from most previously standardised patient or mystery client work in that HIV care is longitudinal as opposed to episodic or acute care. Under these circumstances, the more conventional standardised patient where a single trained actor can present to multiple different care facilities as a simulated patient with a defined set of symptoms or complaints to assess a single episode of care is not feasible. For example, a patient would have to either register as a new patient or have a false "file" introduced into the paper and electronic medical records — which was deemed infeasible and undesirable.

For peer review only

Instead of simulated patients, we recruited existing patients already receiving care at a particular facility and then subsequently trained them on the concepts of quality of care according to the MOH manual on Quality Improvement for HCWs in Zambia. To avoid disclosing their trained status, patients were recruited on the day of their visit prior to them entering the triage area (i.e., the first point of contact with HCWs). Those who consented underwent a single one-on-one training session for 40 to 60 minutes where they were sensitised to the study instrument (which was the same for both TEC and untrained exit clients (UEC)), the MOH care standards, and strategies on being natural yet observant during their clinic visit for that day according to the standard SP approach. These procedures were meant to ensure patients had a clear and uniform understanding on what they should expect during a high-quality patient visit and were attentive to these critical aspects relative to these standards. Immediately after this training, the TEC presented themselves to their facility and completed their visit as they normally would. After their clinic encounter, participants then completed the exit survey in a private area.

For the untrained exit surveys, we took a systematic (every kth, varied by facility size) sample among the patients leaving the facility after attending the clinic on the survey day. Patients were approached by study staff after the visit using a recruitment script to determine their eligibility and were administered the survey after granting consent in a private area.

For both trained and untrained clients, all interviews and surveys were conducted in either English, Bemba or Nyanja depending on the participant's preference. Given the extra time commitments required for the training, TEC participants were given K100 (~$5) for the time spent during training as well as a light snack during the survey administration.

Statistical Analysis

For peer review only - http://bmjopen.bmj.com/site/about/guidelines.xhtml

To assess the association between training and response for each question, we conducted unadjusted and adjusted Poisson regression for each question separately [28]. We then assessed the overall association between training and total sum score. We used descriptive statistics to characterise patient characteristics and report survey responses. In these analyses, most of the survey responses were reverse coded to identify when respondents reported a negative experience. Results for individual questions (binary response) represent prevalence ratios for reporting a lapse in care. To assess the sum score (count data) we used Poisson regression, estimating the rate ratio for reporting lapses in care. All models were adjusted, given potential differences in survey participants related to different recruitment strategies using mixed-effects regression, adjusted for age, sex, education, care status at the time (i.e., continuously retained in care versus returning to care after disengagement/ lost to follow up [LTFU]), secular time (using cubic splines), allowing random effects at the facility level. We present these results for the overall population as well as stratified by different pre-defined patient subgroups. Lastly, we used bubble plots to compare summary assessments of the patient experience at the facility-level using TECs versus UECs. All analyses were performed using STATA 14MP (StataCorp, College Station, TX, USA). This sub-study represents a secondary analysis and no formal power calculations were performed for this outcome.

Statement of Ethics Approval

Ethics approval to conduct this research was granted by the Zambian Ministry of Health, National Health Research Authority, and the institutional review boards of the University of Zambia (008-03-19), the University of Alabama at Birmingham (300003282) and the London School of Hygiene and Tropical Medicine (21384).

For peer review only - http://bmjopen.bmj.com/site/about/guidelines.xhtml

Patient and Public Involvement

Survey questions were developed through a cognitive process with recipients of care. Study implementation guidance was conducted as part of routine CIDRZ partnership with the Zambian MOH through a Human Centered Design workshop. CIDRZ engages with implementing partners and affected communities in health facilities, including people living with HIV often represented by neighbourhood health representatives. Although patients were not directly involved in the design of the parent study intervention or the analysis presented here, all study activities were guided by a Scientific Advisory Board with representation from the MOH and a representative of recipients of HIV care. Dissemination of study results is ongoing.

RESULTS

Characteristics of health facilities and patients

We approached 4375 clients (2955 in the untrained and 1420 in the trained), and 3526 participated, of which 2415 (55.2%) completed experience surveys as untrained exit clients (UEC) (56% female, median age was 40 years (interquartile range [IQR]:32-47 years)) and 1111 (32%) completed experience surveys as trained exit clients (TEC) (50% female with a median age 37 years (IQR:31-45 years). Reasons for non-participation included unavailability at the time due to other commitments. Sixteen percent (16%) of UECs and 40% of TECs who had been lost to care and were returning to care on the day of the survey. Education levels differed between UEC and TEC with 47% and 58% reporting completion of secondary level of education, respectively (Table 1). UEC and TEC were similar for HIV enrolment WHO stage with the largest proportion enrolling at WHO stage 1 and similar in terms of marital status.

Table 1. Socio-demographic characteristics of untrained exit and trained exit clients

For peer review only - http://bmjopen.bmj.com/site/about/guidelines.xhtml

Characteristics	Level	Untrained Exit Clients n=2415 (68%)	Trained Exit Clients n=1111 (32%)
**Sex, n (%)**			
	**Female**	1355 (56)	553 (50)
	**Male**	1060 (44)	558 (50)
**Age, Median (IQR)**			
		40 (32-47)	37 (31-45)
**Age category, n (%)**			
	**<30 years**	453 (19)	258 (23)
	**30-40 years**	828 (34)	416 (37)
	**40-50 years**	815 (34)	304 (27)
	**>50 years**	319 (13)	133 (12)
**Education category**			
	**None**	132 (5)	36 (3)
	**Primary**	654 (27)	166 (15)
	**Secondary**	1134 (47)	645 (58)
	**University**	150 (6)	100 (9)
	**Missing**	307 (13)	151 (14)
**HIV Enrollment Stage**			
	**WHO Stage 1**	1173 (49)	533 (48)
	**WHO Stage 2**	314 (13)	147 (13)
	**WHO Stage 3**	355 (15)	162 (15)
	**WHO Stage 4**	27 (1)	7 (1)
	**Missing**	546 (23)	262 (24)
**Care status at survey visit**			
	**In care**	2038 (84)	664 (60)
	**Returning to care**	377 (16)	447 (40)
**Marital Status**			
	**Single**	257 (11)	167 (15)
	**Married**	1361 (56)	575 (52)
	**Divorced**	248 (10)	108 (10)
	**Widowed**	173 (7)	81 (7)
	**Unknown**	41 (2)	20 (2)
	**Missing**	335 (14)	160 (14)
**Facility size**			
	**< 1000 patients**	591 (25)	245 (22)
	**1000-5000 patients**	897 (37)	485 (44)
	**> 5000 patients**	927 (38)	381 (34)

298

299 Table 2 shows the absolute responses for TEC and UEC. Although most patients reported a
300 good experience, across the questions between 5% and 25% of patients reported poor experiences

For peer review only - http://bmjopen.bmj.com/site/about/guidelines.xhtml

301 in care. For example, when asked if their HIV care provider gave them as much information about 302 their health as they wanted, 13.4% (UEC) vs 24.6% (TEC) of patients reported not being provided 303 with sufficient information about their health. Similarly, between 9.6% vs 18.8% patients reported 304 that their HIV care provider was not spending the right amount of time with them at their visit, and 305 6.8% vs 16.4% reported witnessing rude behaviour.

306

307 **Table 2. Survey responses by training status**

308

Factor	Level	Untrained Exit Client n (%)	Trained Exit Client n (%)
Did your HIV care provider greet you in a way that made you feel comfortable?	Yes	2249 (93.1)	980 (88.2)
	No	166 (6.9)	131 (11.8)
Did your HIV care provider listen to what you said?	Yes	2328 (96.4)	1039 (93.5)
	No	79 (3.3)	64 (5.8)
	Refused	8 (0.3)	8 (0.7)
Did your HIV care provider give you as much information about your health as you wanted?	Yes	2092 (86.6)	838 (75.4)
	No	323 (13.4)	273 (24.6)
Did your HIV care provider allow you to ask questions?	Yes	2082 (86.2)	887 (79.8)
	No	326 (13.5)	222 (20)
	Refused	7 (0.3)	2 (0.2)
Did your HIV care provider spend the right amount of time with you?	Yes	2179 (90.2)	900 (81)
	No	232 (9.6)	209 (18.8)
	Refused	4 (0.2)	2 (0.2)
Overall, how did you feel about the care you received today?	Happy	2231 (92.4)	983 (88.5)
	Unhappy	178 (7.4)	123 (11.1)
	Refused	6 (0.2)	5 (0.4)
Overall, were you satisfied with all your HIV care providers today?	Yes	2206 (91.4)	906 (81.5)
	No	208 (8.6)	202 (18.2)
	Refused	1 (0.0)	3 (0.3)
I witnessed HIV care providers behaving rudely during my visit today	No	2251 (93.2)	928 (83.5)
	Yes	163 (6.8)	182 (16.4)
	Refused	1 (0.0)	1 (0.1)
Were your lab results lost?	No	2143 (88.7)	985 (88.7)
	Yes	268 (11.1)	126 (11.3)
	Not picking up	4 (0.2)	0 (0)
Were you able to pick up your	Yes	2366 (98.0)	1087 (97.8)

For peer review only - http://bmjopen.bmj.com/site/about/guidelines.xhtml

**medicine today?**	No	48 (2.0)	24 (2.2)
	Not Picking Up Meds	1 (0.0)	0 (0)

Effects of training on response patterns: sum score and prevalence ratios

In adjusted models, TECs overall reported poor experiences in care: 1.64 times as frequently as UEC respondents (Sum Score Rate Ratio [RR]: 1.64 [95% CI: 1.39-1.94] (Fig 1, Supplementary Table S1), and reported an increased prevalence of poor experiences in care quality compared to untrained across almost all questions. For example; among TECs compared to UECs, there was an increased prevalence of reports of not being greeted in a way that made them feel welcome (adjusted Prevalence Ratio [aPR]: 1.71 [95% CI: 1.20-2.44]), reporting being dissatisfied with all their HIV care providers during their HIV care visit (aPR: 2.06 [95% CI: 1.61-2.63]) and witnessing any providers behaving rudely during their visit (aPR: 2.28 [95% CI: 1.63-3.19]) (Fig 1, Supplementary Table S1).

Impact of training across age, sex, and gender to differences in responses

In stratified analysis of the impact of training on the sum score, training was consistently associated with increased identification of poor experiences in care across all subgroups apart from those aged 50 years or older and those with no education. We also observed that training had a larger impact among females compared to males, those with a primary education only, and among individuals presenting at smaller facilities (Fig 2). We observed similarities in responses on the impact of training on different age categories, sex, care status and different levels of education when we looked at individual questions except for the question on providers spending the right amount of time where we found that females were twice as likely to report lapses with care compared to males (Supplementary Figure 1). Using TECs gave worse assessments of patient experience at the facility-level regardless of facility size compared to UECs (Fig 3, Supplementary Figure 2).

For peer review only - http://bmjopen.bmj.com/site/about/guidelines.xhtml

DISCUSSION

Disengaged patients often express a disconnect between their care expectations and the provider's style, hence experience is bound to vary across facilities [8]. This disconnect can lead to dissatisfaction with HIV services which can often lead to patients dropping out of care[8,11,29]. A brief training for patients living with HIV on how to evaluate the quality and experience of routine care changed patient experience reports compared to untrained patients using the same instrument. Patients who underwent a brief training identified more lapses in care across most questions. Women and young people were more likely to report critical responses after training - consistent with the idea that those who feel least empowered underwent the biggest change. Differences were also bigger for questions in which social desirability is likely to operate. For example, larger differences were observed for witnessing rude behaviour, while no differences were observed for more objective questions such as whether lab results were lost.

Improving HIV health outcomes requires new strategies that minimise methodological biases and includes everyone the patient encounters during their visit, including clinical officers, doctors, nurses, data clerks, and lay HCWs. Our TEC approach could contribute to getting a true reflection of how much value patients place on things such as effective communication, being greeted appropriately, or being treated with care and respect at all these different touch points. Involving patients in their own care and design of health services has been linked to improved HIV care retention and patient outcomes, such as higher viral suppression rates [30–32]. As progress is being made towards UNAIDS 95-95-95 targets, the global HIV sector is constantly reviewing priorities and challenges for optimal engagement in care [33,34]. Patient experience is a key indicator of

For peer review only - http://bmjopen.bmj.com/site/about/guidelines.xhtml

healthcare quality for meeting the 95-95-95 targets: delivering services patients need, can access, and address wider determinants of poor health. Clinicians and health systems must address HIV patients' needs from diagnosis to death to ensure healthy ageing and viral suppression. Other outcomes in Zambia [11,35,36] show that lifelong needs vary by facility, highlighting the importance of metrics that measure patient experience accurately. We have shown that it is feasible to involve patients in assessing the quality of care and this could potentially lead to involvement of patients in the redesign of healthcare services.

Because HIV care is longitudinal, SP, who are often used to evaluate episodic care, require highly skilled people to pose as a simulated patient making one visit to multiple clinics, posing practical implementation challenges in our setting[19–24]. Contrary to SP, we evaluated care quality without using simulated patients and administered the survey once among people in long term care. Using real patients instead of simulated ones drawn from outside the true patient population, we would argue, made our TEC approach more applicable and reproducible in clinical settings. We were able to record HCW behaviour in a typical HIV context using this concealment method, potentially reducing the impact of the Hawthorne effect. Our TECs also consistently identified more lapses in care, potentially reducing social desirability bias and ability to identify issues at the facility. Even though training takes time, the increased quality of our measurement allows one to perform fewer surveys. With traditional approaches like exit surveys, one would require a larger sample size, but this does not address bias [37].

Our findings are consistent with a study done in South Africa which found that non-clinical dimensions of care play a bigger role in determining an overall satisfactory experience for standardised patients when compared to untrained patients[37]. However, our findings may contradict previous suggestions that tailoring support to individuals to build skills and confidence

For peer review only

through patient activation can lead to trained/informed patients reporting a better experience than untrained/ uninformed [38]. TECs cared about the following non-clinical aspects of care: rude providers, being satisfied with HIV care providers, and spending enough time with providers. This finding is consistent with a previous study in Zambia, where patients reported rude HCWs deterring HIV care engagement [8,10,11]. This could mean that studies assessing patient experience with TEC could focus on a few questions to save time and resources. Questions like, "Did you pick up your medicine or lab results at your visit?" may not add much to a TEC survey because they are definitive, and training appears to influence subjective care dimensions.

Women TECs were generally more critical about the care they received and would likely provide a more accurate reflection of the health system, possibly because they have better health-seeking behaviour than men, which may be strongly influenced by local gender norms and health service structures designed to engage women of reproductive age [39]. There is some consistency with other findings that women may be more interested in their care than men, especially in facilities that provide integrated services for women and their children [9,40]. Despite longer wait times, women were more satisfied with integrated facilities [41]. In addition, middle-aged people between 40-50 benefited the most from training. Compared to older people over 50, younger people under 30 were less satisfied with the care they received and often felt they were not greeted by a HCW during their visit. This finding is consistent with cultural norms where younger people are less respected[42]. Given the current strategy of targeting young people, who account for most new infections, these findings suggest an important new approach to identifying what young people value most. Education level was among the strongest predictors of patient experience feedback. Well-educated patients were found to have a less critical/better HIV care visit experience compared to participants with lower levels of educational attainment. This difference in care experience report

For peer review only - http://bmjopen.bmj.com/site/about/guidelines.xhtml

may be associated, at least in part, with the HCW perception of the patient in the facility. Research conducted in Nigeria discovered that people with higher levels of education are frequently given better and more considerate treatment by HCW, hence limited by a form of discrimination/ socioeconomic status bias [43,44].

The observed effect of training on patient experience is likely multifaceted potentially stemming from increased attention and recall to the exit survey items which solicited a feeling of empowerment to be more critical of the care received. In future studies, patient activation should be measured as an outcome to see how training changes the patient's engagement with their care over time [38]. Further research is required into why women TECs reported poorer experiences with care than men. Other studies that have used SP to assess medical students' performance showed that women were more critical on certain aspects of care. These studies also recommend matching of SPs to clinicians by sex [45], something we were not able to do given the nature of our study in primary health facilities where we assessed interpersonal communication with HCWs at all levels. Perhaps our findings call for more investigation into the integration of women's services, such as family planning and children's services with HIV care given some studies have shown this can improve patient satisfaction.

Limitations

Our findings should be interpreted with caution due to the following limitations. Because this was the first time such a study was done, we recruited educated participants who were able to read and write, perceived to have good recall ability and were able to comprehend things. Our study was only done in Lusaka province in facilities that were largely urban except for one facility which was peri-urban hence it is hard to generalise these findings. Another limitation in our approach is the one-time cross-sectional nature of our measurements among people in long term HIV care. If more

For peer review only - http://bmjopen.bmj.com/site/about/guidelines.xhtml

measures were collected from each TEC, we may well see them being activated in a way that results in an improvement in their experience based on the skills they develop to seek better care from providers which ultimately would improve their retention in care. Despite its limitations, the TEC method provides valuable information about healthcare quality, even though it is limited to situations where "walk-ins" are permitted. Our approach only focused on real patients accessing care and we did not manipulate any patient files, so it is possible that some TECs were known to the facility as patients accessing chronic care. Our approach does require a trained interviewer to speak with TECs after their visits, but this is not any different to what already exists. In future, it may be worth using the domains in the national HIV guidelines as the gold standard, but we did not do this as our aim was to come up with a low-cost approach that can easily be rolled out. In addition, the concept of patient centred care is still catching on in Zambia. Our TEC approach can be used to further the knowledge in provider attitudes to other relatively new approaches to delivering quality HIV care such as differentiated service delivery (DSD) for stable patients by assessing whether HCWs follow guidelines when offering this [34]. We also see an opportunity to assess provider patient communication of viral load laboratory results by use of a universal script for each TEC to assess if they are communicated to and if unsuppressed but adherent, what procedures followed.

Conclusion

TEC offers pragmatic methods for health systems in low-income countries to assess non-clinical dimensions of care (communication, respect, and autonomy) which are grounded on the concept of health-system responsiveness and could be critical to the transformation of low-quality health systems to high quality ones[46]. Hawthorne effects and social desirability biases may be mitigated using TECs. We were able to capture HCWs behaviour in a normal day to day low middle

For peer review only

income setting using similar approaches recommended by King and colleagues that minimise harm to HCWs and SPs [15]. Our findings suggest that TECs provide a more critical appraisal of some aspects of the quality of HIV care. It provides new insights in the Zambian context on what patients' value when they interact with the health system. This could be important given the need to reduce loss to follow up among new ART clients who disengage within the first 6 months of treatment due to a bad first encounter with the health system. Our TEC approach could be used to assess reengagement interventions. The fact that TECs had a better understanding of the items solicited or felt empowered to be more critical shows that the training we provided worked. This low-cost method could be reproduced in other routine settings and presents an opportunity to further institutionalise patient centred care by evaluating what happens at the point of contact between the patient, the health facility, and the health provider. The implications are that it provides an opportunity to improve HIV care, meet patients' expectations and can serve as a monitoring tool for healthcare performance. Coupled with the recent approaches to client led monitoring in HIV care, our approach can be used to enhance decision making that considers patients' involvement.

For peer review only

For peer review only - http://bmjopen.bmj.com/site/about/guidelines.xhtml

Data Availability Statement

The Government of Zambia allows data sharing when applicable local conditions are satisfied. In this case, the data from the study will be made available to any interested researchers upon request. The CIDRZ Ethics and Compliance Committee is responsible for approving such request. To request data access, one must write to the Secretary to the Committee/Head of Research Operations, Mrs. Hope Chinganya (Hope.Chinganya@cidrz.org) mentioning the intended use for the data. The Committee will then facilitate review and authorization to release the data as requested. Data requests must include contact information, a research project title, and a description of the intended use.

Funding

This work was supported, in whole or in part, by the Bill & Melinda Gates Foundation [INV-010563]. Under the grant conditions of the Foundation, a Creative Commons Attribution 4.0 Generic License has already been assigned to the Author Accepted Manuscript version that might arise from this submission. This work was also supported by the National Center for Advancing Translational Sciences (grant KL2 TR002346 to Dr Mody) and the National Institute of Allergy and Infectious Diseases (grant K24 AI134413 to Dr Geng). The funders had no role in study design, data collection and analysis, decision to publish, or preparation of the manuscript.

Disclaimer

The contents of this paper are the sole responsibility of the authors and do not necessarily reflect the views of the Bill & Melinda Gates Foundation.

Competing interest: Non declared

For peer review only - http://bmjopen.bmj.com/site/about/guidelines.xhtml

Acknowledgements

We would like to thank recipients of care for taking part in this study. We would like to thank the Zambian Ministry of Health, the US President's Emergency Plan for AIDS Relief (PEPFAR) through the US Centers for Disease Control and Prevention/Zambia (CDC) and the Centre for Infectious Disease Research in Zambia for their leadership in ensuring those in HIV care continue to receive life-saving HIV treatment. We would also like to thank the healthcare workers who continued to faithfully deliver HIV care in the face of the COVID-19 pandemic

Contributors

KS: guarantor, lead author, conducted all analyses, led data management activities, field coordination of activities and designed data collection tools. JMP: field coordination of data, assisted with analysis and revising it critically for important intellectual content, designed data collection tools. AM: assisted with analysis, framing, and revising it critically for important intellectual content. BR: final approval for publication, assisted with framing and revising it critically for important intellectual content CB: field coordination of data quality processes and data acquisition IEW: drafted statistical analysis plan, assisted with conceptualisation and interpretation of data. JM: assisted with data acquisition and cleaning, field coordination of data quality processes. SS: led intervention implementation, project administration and data curation. LKB, NM, AS: cognitively tested data collection tools, assisted with conceptualization, underlying data processes, and assisted with writing and data interpretation. DM, MM-M: advised implementation details. AW, JH: assisted with framing and revising it critically for important intellectual content. CBM: lead for underlying data processes and assisted with funding acquisition. CBH: funding acquisition, assisted with conceptualisation and advised regarding intervention implementation details. IS: funding

For peer review only

For peer review only - http://bmjopen.bmj.com/site/about/guidelines.xhtml

acquisition, assisted with conceptualisation and manuscript writing. EHG: funding acquisition, led conceptualisation and advised on all analyses, final approval for publication.

Statement of Ethics Approval

Ethics approval to conduct this research was granted by the Zambian Ministry of Health, National Health Research Authority, and the institutional review boards of the University of Zambia (008-03-19), the University of Alabama at Birmingham (300003282) and the London School of Hygiene and Tropical Medicine (21384).

For peer review only

REFERENCES

1 UNAIDS JUNP on H-G. *In Danger: UNAIDS Global AIDS Update 2022*. 2022. doi:10.18356/9789210019798

2 Institute of Medicine & Committee on Quality of Health Care in America. *Crossing the Quality Chasm: A New Health System for the 21st Century*. 2001. doi:10.1136/bmj.323.7322.1192

3 World Health Organization. Maintaining and Improving Quality of Care Within HIV Clinical Services. 2019. http://apps.who.int/bookorders. (accessed 11 Mar 2020).

4 Edmonds KA, Aspiras OG, Rose JP, *et al.* Cross-sectional evaluation of perceived health care provider engagement, self-efficacy, and ART adherence in people living with HIV/AIDS. *AIDS Care* 2021;**33**:154–8. doi:10.1080/09540121.2019.1703889

5 Bakken S, Holzemer WL, Brown MA, *et al.* Relationships between perception of engagement with health care provider and demographic characteristics, health status, and adherence to therapeutic regimen in persons with HIV/AIDS. *AIDS Patient Care STDS* 2000;**14**:189–97. doi:10.1089/108729100317795

6 Okoli C, Brough G, Allan B, *et al.* Shared Decision Making Between Patients and Healthcare Providers and its Association with Favorable Health Outcomes Among People Living with HIV. *AIDS Behav* 2021;**25**:1384–95. doi:10.1007/s10461-020-02973-4

7 Kwan A, Daniels B, Bergkvist S, *et al.* Use of standardised patients for healthcare quality research in low- and middle-income countries. *BMJ Glob Health* 2019;**4**:e001669. doi:10.1136/bmjgh-2019-001669

8 Mwamba C, Sharma A, Mukamba N, *et al.* 'They care rudely!': resourcing and relational health system factors that influence retention in care for people living with HIV in Zambia. *BMJ Glob Health* 2018;**3**:e001007. doi:10.1136/bmjgh-2018-001007

9 Topp SM, Mwamba C, Sharma A, *et al.* Rethinking retention: Mapping interactions between multiple factors that influence long-term engagement in HIV care. *PLoS One* 2018;**13**:e0193641.https://doi.org/10.1371/journal.pone.0193641

10 Zanolini A, Sikombe K, Sikazwe I, *et al.* Understanding preferences for HIV care and treatment in Zambia: Evidence from a discrete choice experiment among patients who have been lost to follow-up. *PLoS Med* 2018;**15**:e1002636. doi:10.1371/JOURNAL.PMED.1002636

11 Sikazwe I, Eshun-Wilson I, Sikombe K, *et al.* Patient-reported Reasons for Stopping Care or Switching Clinics in Zambia: A Multi-site, Regionally Representative estimate using a multi-stage sampling-based approach in Zambia. *Clinical Infectious Diseases* Published Online First: 2020. doi:10.1093/cid/ciaa1501

12 Mody A, Sikombe K, Beres LK, *et al.* Profiles of HIV Care Disruptions Among Adult Patients Lost to Follow-up in Zambia. *JAIDS Journal of Acquired Immune Deficiency Syndromes* 2020;**Publish Ah**. doi:10.1097/qai.0000000000002530

13 Porter LE, Bouey PD, Curtis S, *et al.* Beyond indicators: Advances in global HIV monitoring and evaluation during the PEPFAR era. *J Acquir Immune Defic Syndr (1988)* 2012;**60**. doi:10.1097/QAI.0b013e31825cf345

14 MILLER FRANCO L, FRANCO C, KUMWENDA N, *et al.* Methods for assessing quality of provider performance in developing countries. *International Journal for Quality in Health Care* 2002;**14**:17–24. doi:10.1093/intqhc/14.suppl_1.17

15 King JJC, Das J, Kwan A, *et al.* How to do (or not to do) … using the standardized patient method to measure clinical quality of care in LMIC health facilities. *Health Policy Plan* 2019;**34**:625–34. doi:10.1093/heapol/czz078

For peer review only - http://bmjopen.bmj.com/site/about/guidelines.xhtml

16 Onishi J, Gupta S, Peters DH. Comparative analysis of exit interviews and direct clinical observations in Pediatric Ambulatory Care Services in Afghanistan. *International Journal for Quality in Health Care* 2011;**23**:76–82. doi:10.1093/intqhc/mzq074

17 Luck J, Peabody JW. Using standardised patients to measure physicians' practice: validation study using audio recordings. *BMJ* 2002;**325**:679. doi:10.1136/bmj.325.7366.679

18 Beullens J, Rethans JJ, Goedhuys J, *et al.* The use of standardized patients in research in general practice. *Fam Pract* 1997;**14**:58–62. doi:10.1093/fampra/14.1.58

19 Weiner SJ, Schwartz A. Directly observed care: Can unannounced standardized patients address a gap in performance measurement? *J Gen Intern Med* 2014;**29**:1183–7. doi:10.1007/s11606-014-2860-7

20 Nestel D, Bearman M. *Simulated Patient Methodology: Theory, Evidence and Practice*. 2014. doi:10.1002/9781118760673

21 Larsen A, Wilson KS, Kinuthia J, *et al.* Standardised patient encounters to improve quality of counselling for pre-exposure prophylaxis (PrEP) in adolescent girls and young women (AGYW) in Kenya: study protocol of a cluster randomised controlled trial. *BMJ Open* 2020;**10**:e035689–e035689. doi:10.1136/bmjopen-2019-035689

22 Daniels B, Dolinger A, Bedoya G, *et al.* Use of standardised patients to assess quality of healthcare in Nairobi, Kenya: A pilot, cross-sectional study with international comparisons. *BMJ Glob Health* Published Online First: 2017. doi:10.1136/bmjgh-2017-000333

23 Zabar S, Hanley K, Stevens D, *et al.* Unannounced standardized patients: A promising method of assessing patient-centered care in your health care system. *BMC Health Serv Res* 2014;**14**. doi:10.1186/1472-6963-14-157

24 Boffa J, Moyo S, Chikovore J, *et al.* Quality of care for tuberculosis and HIV in the private health sector: a cross-sectional, standardised patient study in South Africa. *BMJ Glob Health* 2021;**6**:e005250. doi:10.1136/bmjgh-2021-005250

25 Wachira J, Middlestadt S, Reece M, *et al.* Psychometric assessment of a physician-patient communication behaviors scale: The perspective of adult HIV patients in Kenya. *AIDS Res Treat* 2013;**2013**. doi:10.1155/2013/706191

26 Campbell C, Lockyer J, Laidlaw T, *et al.* Assessment of a matched-pair instrument to examine doctor–patient communication skills in practising doctors. *Med Educ* 2007;**41**:123–9. doi:https://doi.org/10.1111/j.1365-2929.2006.02657.x

27 Bieber C, Müller KG, Nicolai J, *et al.* How Does Your Doctor Talk with You? Preliminary Validation of a Brief Patient Self-Report Questionnaire on the Quality of Physician–Patient Interaction. *J Clin Psychol Med Settings* 2010;**17**:125–36. doi:10.1007/s10880-010-9189-0

28 Zou G. A Modified Poisson Regression Approach to Prospective Studies with Binary Data. *Am J Epidemiol* 2004;**159**:702–6. doi:10.1093/aje/kwh090

29 Shabalala FS, Vernooij E, Pell C, *et al.* Understanding reasons for discontinued antiretroviral treatment among clients in test and treat: a qualitative study in Swaziland. *J Int AIDS Soc* 2018;**21**:e25120. doi:10.1002/jia2.25120

30 Grimsrud A, Bygrave H, Doherty M, *et al.* Reimagining HIV service delivery : the role of differentiated care from prevention to suppression. *J Acquir Immune Defic Syndr* 2016;:10–2. doi:10.7448/IAS.19.1.21484

31 Camlin CS, Neilands TB, Odeny TA, *et al.* Patient-reported factors associated with reengagement among HIV-infected patients disengaged from care in East Africa. *AIDS* 2015;:1. doi:10.1097/QAD.0000000000000931

For peer review only - http://bmjopen.bmj.com/site/about/guidelines.xhtml

32 Geng EH, Bangsberg DR, Musinguzi N, *et al.* Understanding Reasons for and Outcomes of Patients Lost to Follow-Up in Antiretroviral Therapy Programs in Africa Through a Sampling-Based Approach. *JAIDS Journal of Acquired Immune Deficiency Syndromes* 2010;**53**:405–11. doi:10.1097/QAI.0b013e3181b843f0
33 Ford N, Geng E, Ellman T, *et al.* Emerging priorities for HIV service delivery. *PLoS Med* 2020;**17**:e1003028. doi:10.1371/JOURNAL.PMED.1003028
34 Ehrenkranz P, Grimsrud A, Holmes CB, *et al.* Expanding the Vision for Differentiated Service Delivery: A Call for More Inclusive and Truly Patient-Centered Care for People Living With HIV. *J Acquir Immune Defic Syndr* 2021;**86**:147. doi:10.1097/QAI.0000000000002549
35 Holmes CB, Sikazwe I, Sikombe K, *et al.* Estimated mortality on HIV treatment among active patients and patients lost to follow-up in 4 provinces of Zambia: Findings from a multistage sampling-based survey. *PLoS Med* 2018;**15**:e1002489. doi:10.1371/journal.pmed.1002489
36 Sikazwe I, Eshun-Wilson I, Sikombe K, *et al.* Retention and viral suppression in a cohort of HIV patients on antiretroviral therapy in Zambia: Regionally representative estimates using a multistage-sampling-based approach. *PLoS Med* 2019;**16**:1–17. doi:10.1371/journal.pmed.1002811
37 Hompashe DM, Gerdtham U-G, Christian CS, *et al.* 'The nurse did not even greet me': how informed versus non-informed patients evaluate health systems responsiveness in South Africa. *BMJ Glob Health* 2021;**6**:e004360. doi:10.1136/bmjgh-2020-004360
38 Hibbard JH, Greene J. What The Evidence Shows About Patient Activation: Better Health Outcomes And Care Experiences; Fewer Data On Costs. *Health Aff* 2013;**32**:207–14. doi:10.1377/hlthaff.2012.1061
39 Colvin CJ. Strategies for engaging men in HIV services. Lancet HIV. 2019;**6**:e191–200. doi:10.1016/S2352-3018(19)30032-3
40 Kerkhoff AD, Sikombe K, Eshun-Wilson I, *et al.* Mortality estimates by age and sex among persons living with HIV after ART initiation in Zambia using electronic medical records supplemented with tracing a sample of lost patients: A cohort study. *PLoS Med* 2020;**17**:e1003107.https://doi.org/10.1371/journal.pmed.1003107
41 Narasimhan M, Yeh PT, Haberlen S, *et al.* Integration of HIV testing services into family planning services: a systematic review. *Reprod Health* 2019;**16**:61. doi:10.1186/s12978-019-0714-9
42 Godia PM, Olenja JM, Lavussa JA, *et al.* Sexual reproductive health service provision to young people in Kenya; Health service providers' experiences. *BMC Health Serv Res* 2013;**13**:1–13. doi:10.1186/1472-6963-13-476/TABLES/2
43 Roberts BW, Puri NK, Trzeciak CJ, *et al.* Socioeconomic, racial and ethnic differences in patient experience of clinician empathy: Results of a systematic review and meta-analysis. *PLoS One* 2021;**16**:e0247259.https://doi.org/10.1371/journal.pone.0247259
44 Aloh HE, Onwujekwe OE, Aloh OG, *et al.* Impact of socioeconomic status on patient experience on quality of care for ambulatory healthcare services in tertiary hospitals in Southeast Nigeria. *BMC Health Serv Res* 2020;**20**:473. doi:10.1186/s12913-020-05332-0
45 Humphrey-Murto S, Touchie C, Wood TJ, *et al.* Does the gender of the standardised patient influence candidate performance in an objective structured clinical examination? *Med Educ* 2009;**43**:521–5. doi:10.1111/j.1365-2923.2009.03336.x
46 Kruk ME, Gage AD, Arsenault C, *et al.* High-quality health systems in the Sustainable Development Goals era: time for a revolution. Lancet Glob Health. 2018;**6**:e1196–252. doi:10.1016/S2214-109X(18)30386-3

For peer review only

Figure 1. Forest plot comparing responses from Trained Exit Clients (TEC) relative to Untrained Exit Clients (UEC) on 10 measures of clinic experience. Points indicate the rate ratio (for sum score) or prevalence ratio (for all others) for identifying a lapse in care in TEC surveys as compared to UEC. The sum score represents the total number of binary responses (yes vs no) across all clients in one group shown as a rate ratio. The red line indicates a rate or prevalence ratio of 1 and values greater than this indicates more lapses in care identified in TECs. Results are based on mixed-effects models adjusted for age, sex, education with a random effect at the facility.

Figure 2. Impact of Training on Identifying Care Lapses Stratified by Subgroups (N=3480). When all questions were collapsed into a Sum score among TEC, females were more likely to report lapses in care quality than males. We observed some level of interaction for care status, age category, education category and facility size.

Figure 3. Bubble plot showing Trained Exit Sum Score vs Untrained Exit Sum Score. Each bubble represents a single facility's performance. Each bubble's size indicates the number of patients at each facility with larger bubbles corresponding to larger facilities. The horizontal position notes the Untrained Exit Sum Score for all questions against the facility, and the vertical position notes the Trained Exit sum score at the same facility.

For peer review only

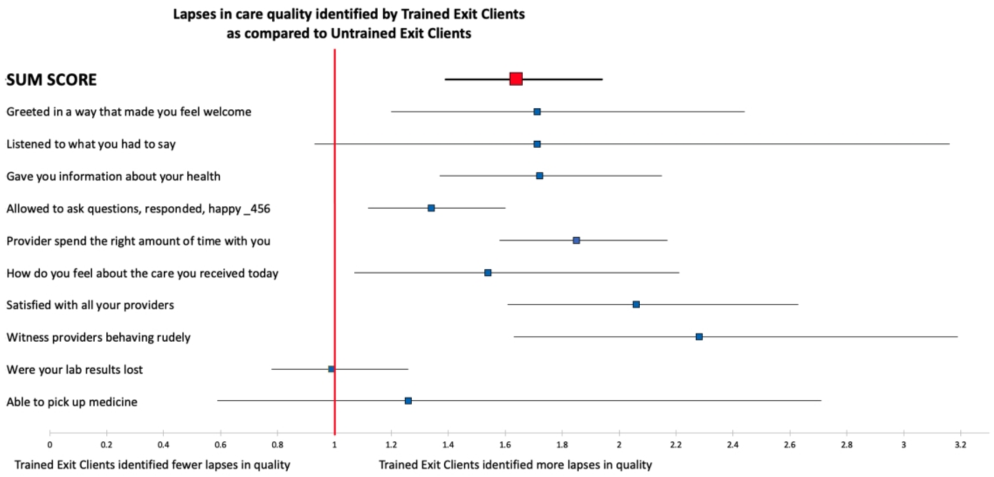

674x330mm (72 x 72 DPI)

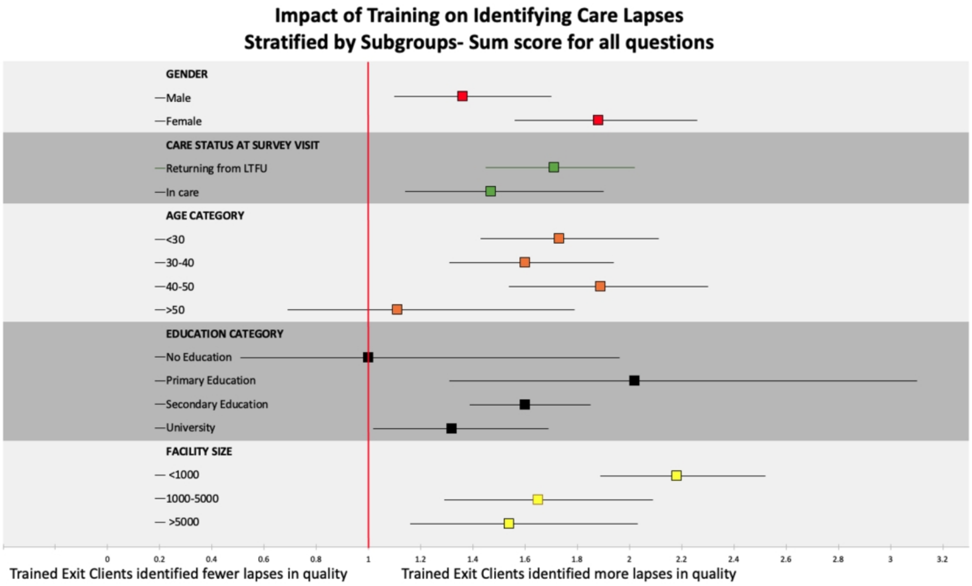

629x374mm (72 x 72 DPI)

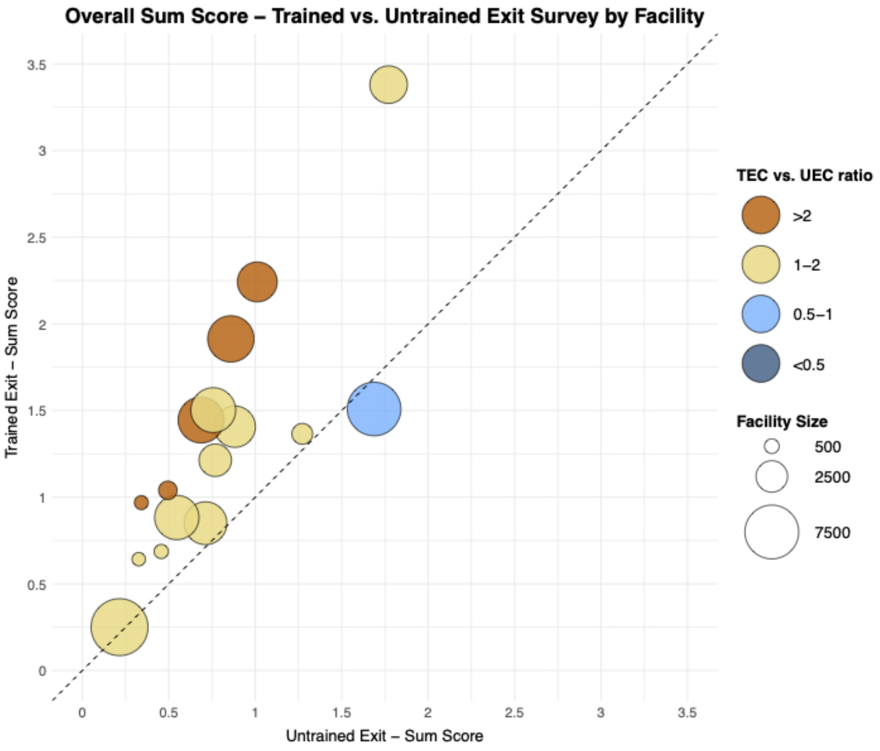

279x215mm (72 x 72 DPI)

Supplementary Tables S1

Supplementary Table S1. Mixed effect Poisson regression comparing 10 questions for Trained Exit Clients vs Untrained Exit Clients. Adjusted for age, sex, education, and study period.

Trained Exit Clients	Prevalence ratio (PR) Unadjusted	P value	95% Confidence Interval (CI)	PR-Adjusted	P value	95% Confidence Interval (CI)	N
Sum score (Rate ratio)	1.73	<0.01	1.47-2.02	1.64	<0.01	1.39-1.94	3480
Did your HIV care provider greet you in a way that made you feel comfortable?	1.74	0.01	1.24-2.44	1.71	<0.01	1.20-2.44	3526
Did your HIV care provider listen to what you said?	1.77	0.09	0.91-3.45	1.71	0.09	0.93-3.16	3510
Did your HIV care provider give you as much information about your health as you wanted?	1.82	<0.01	1.43-2.33	1.72	<0.01	1.37-2.15	3526
Did your HIV care provider allow you to ask questions?	1.44	<0.01	1.20-1.73	1.34	<0.01	1.12-1.6	3517
Did your HIV care provider spend the right amount of time with you?	1.94	<0.01	1.66-2.27	1.85	<0.01	1.58-2.17	3520
Overall, how did you feel about the care you received today?	1.51	0.02	1.06-2.16	1.54	0.02	1.07-2.21	3515
Overall, were you satisfied with all your HIV care providers today?	2.12	<0.01	1.68-2.66	2.06	<0.01	1.61-2.63	3522
I witnessed HIV care providers behaving rudely during my visit today	2.39	<0.01	1.73-3.32	2.28	<0.01	1.63-3.19	3524
Were your lab results lost?	0.99	0.98	0.84-1.19	0.99	0.93	0.78-1.26	3522
Were you able to pick up your medicine today?	1.04	0.90	0.57-1.89	1.26	0.55	0.59-2.71	3525

For peer review only

For peer review only

Supplementary Figure 1.

Impact of Training on Identifying Care Lapses Stratified by Subgroups for 10 questions. We observed some level of interaction for care status, age category, education category and facility size. Panel **a)** Greet you in a way that made you feel comfortable **b)** Listen to what you said **c)** Give you as much information about your health as you wanted **d)** Allowed you to ask questions, responded, happy q456 **e)** spend the right amount of time with you **f)** feel about the care you received today **g)** satisfied with all your HIV care providers today **h)** witnessed HIV care providers behaving rudely during my visit today **i)** lost lab results **j)** pick up meds

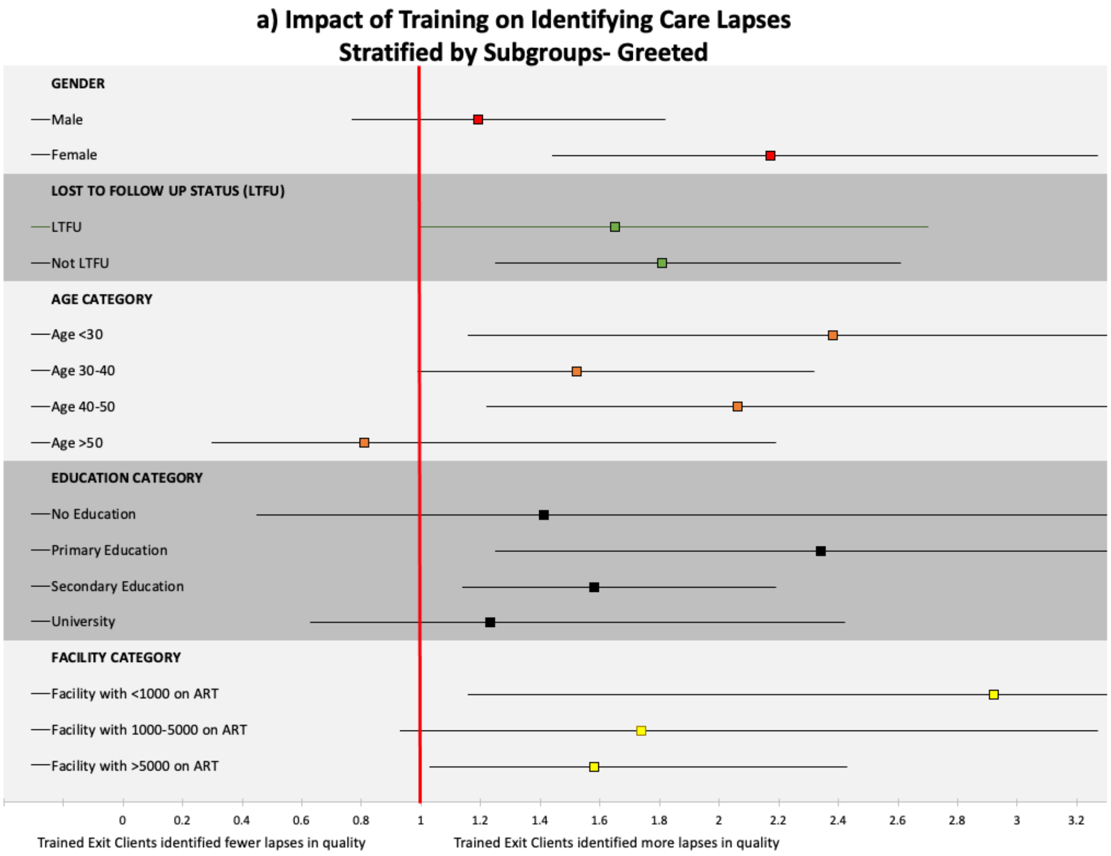

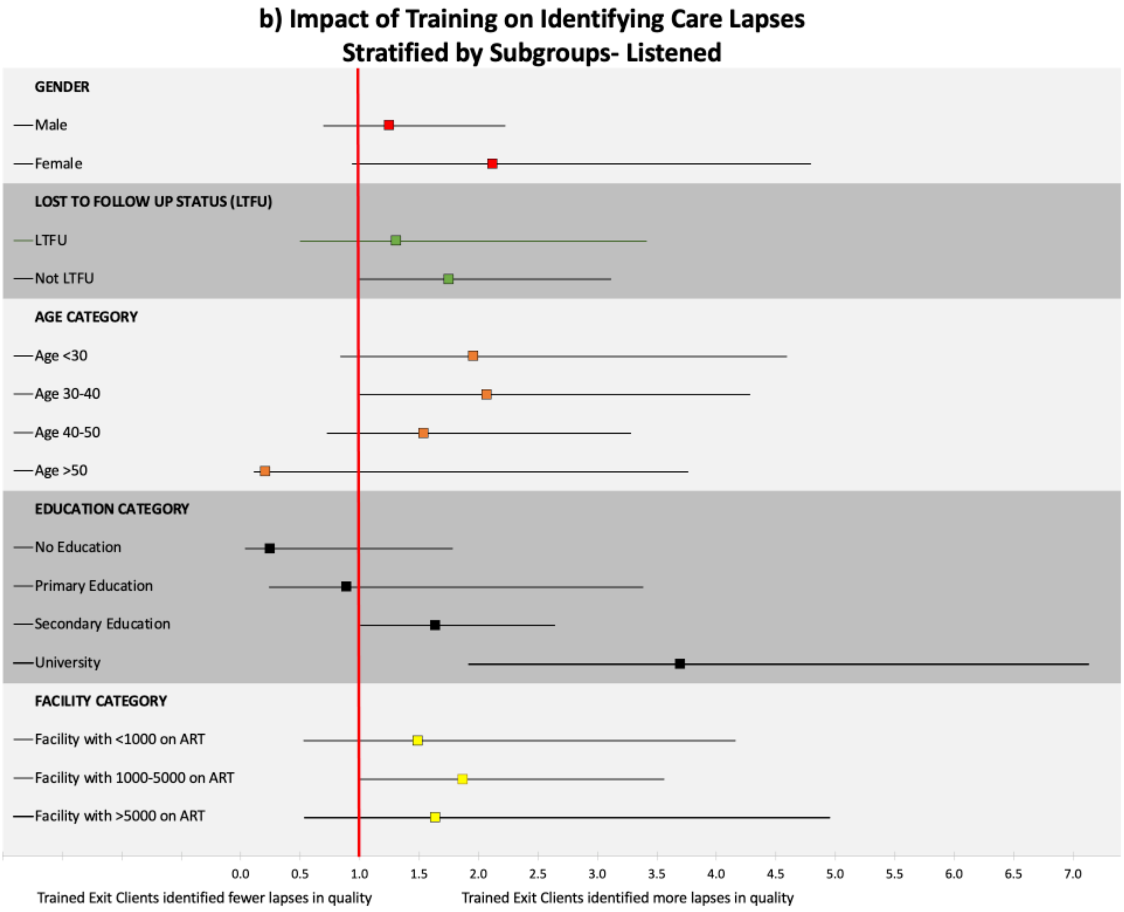
b) Impact of Training on Identifying Care Lapses
Stratified by Subgroups- Listened
GENDER
Male
Female
LOST TO FOLLOW UP STATUS (LTFU)
LTFU
Not LTFU
AGE CATEGORY
Age <30
Age 30-40
Age 40-50
Age >50
EDUCATION CATEGORY
No Education
Primary Education
Secondary Education
University
FACILITY CATEGORY
Facility with <1000 on ART
Facility with 1000-5000 on ART
Facility with >5000 on ART
0.0 0.5 1.0 1.5 2.0 2.5 3.0 3.5 4.0 4.5 5.0 5.5 6.0 6.5 7.0
Trained Exit Clients identified fewer lapses in quality
Trained Exit Clients identified more lapses in quality

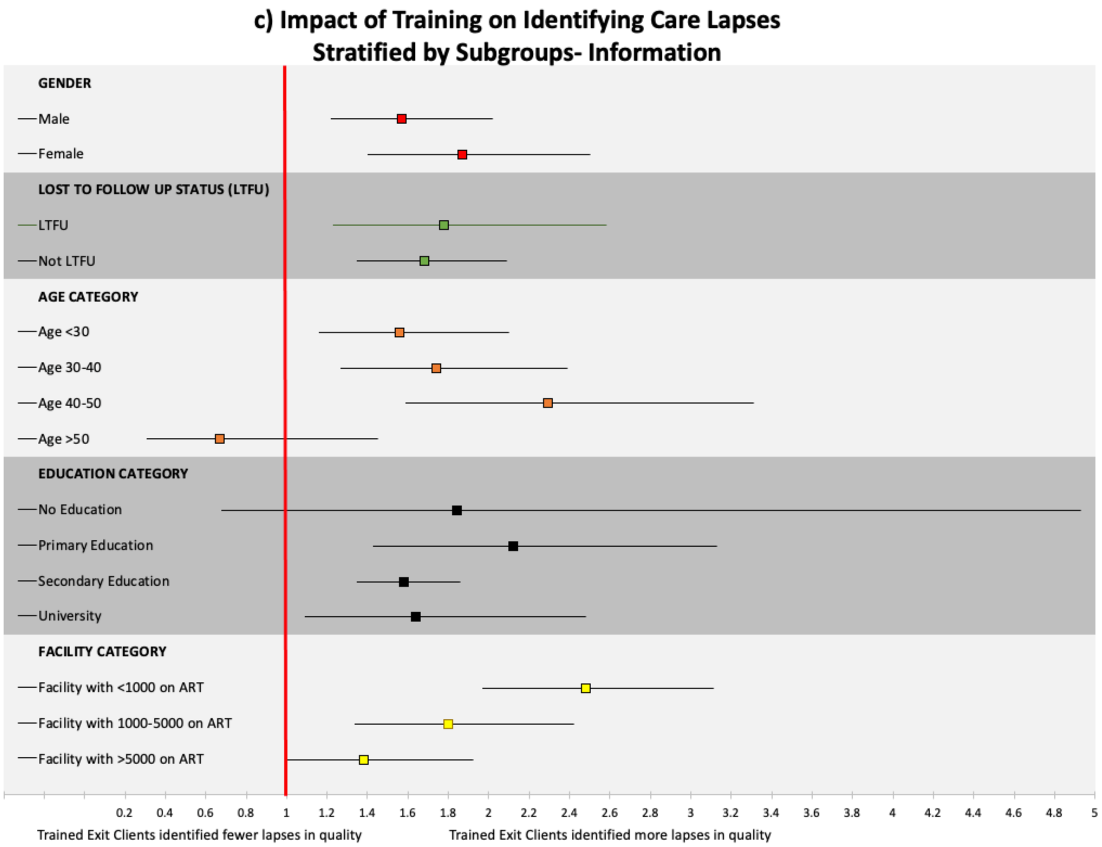
c) Impact of Training on Identifying Care Lapses
Stratified by Subgroups- Information
GENDER
Male
Female
LOST TO FOLLOW UP STATUS (LTFU)
LTFU
Not LTFU
AGE CATEGORY
Age <30
Age 30-40
Age 40-50
Age >50
EDUCATION CATEGORY
No Education
Primary Education
Secondary Education
University
FACILITY CATEGORY
Facility with <1000 on ART
Facility with 1000-5000 on ART
Facility with >5000 on ART
0.2 0.4 0.6 0.8 1 1.2 1.4 1.6 1.8 2 2.2 2.4 2.6 2.8 3 3.2 3.4 3.6 3.8 4 4.2 4.4 4.6 4.8 5
Trained Exit Clients identified fewer lapses in quality
Trained Exit Clients identified more lapses in quality

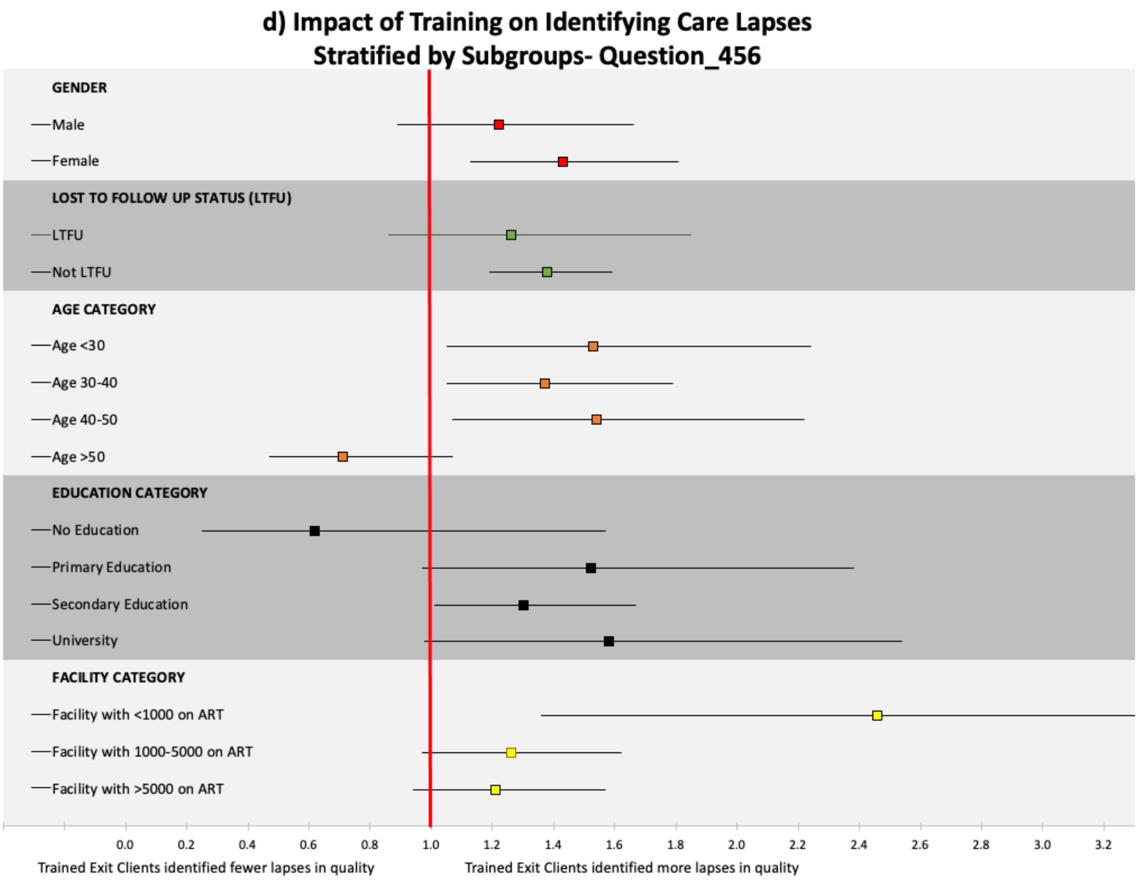
d) Impact of Training on Identifying Care Lapses
Stratified by Subgroups- Question_456
GENDER
Male
Female
LOST TO FOLLOW UP STATUS (LTFU)
LTFU
Not LTFU
AGE CATEGORY
Age <30
Age 30-40
Age 40-50
Age >50
EDUCATION CATEGORY
No Education
Primary Education
Secondary Education
University
FACILITY CATEGORY
Facility with <1000 on ART
Facility with 1000-5000 on ART
Facility with >5000 on ART
0.0 0.2 0.4 0.6 0.8 1.0 1.2 1.4 1.6 1.8 2.0 2.2 2.4 2.6 2.8 3.0 3.2
Trained Exit Clients identified fewer lapses in quality
Trained Exit Clients identified more lapses in quality

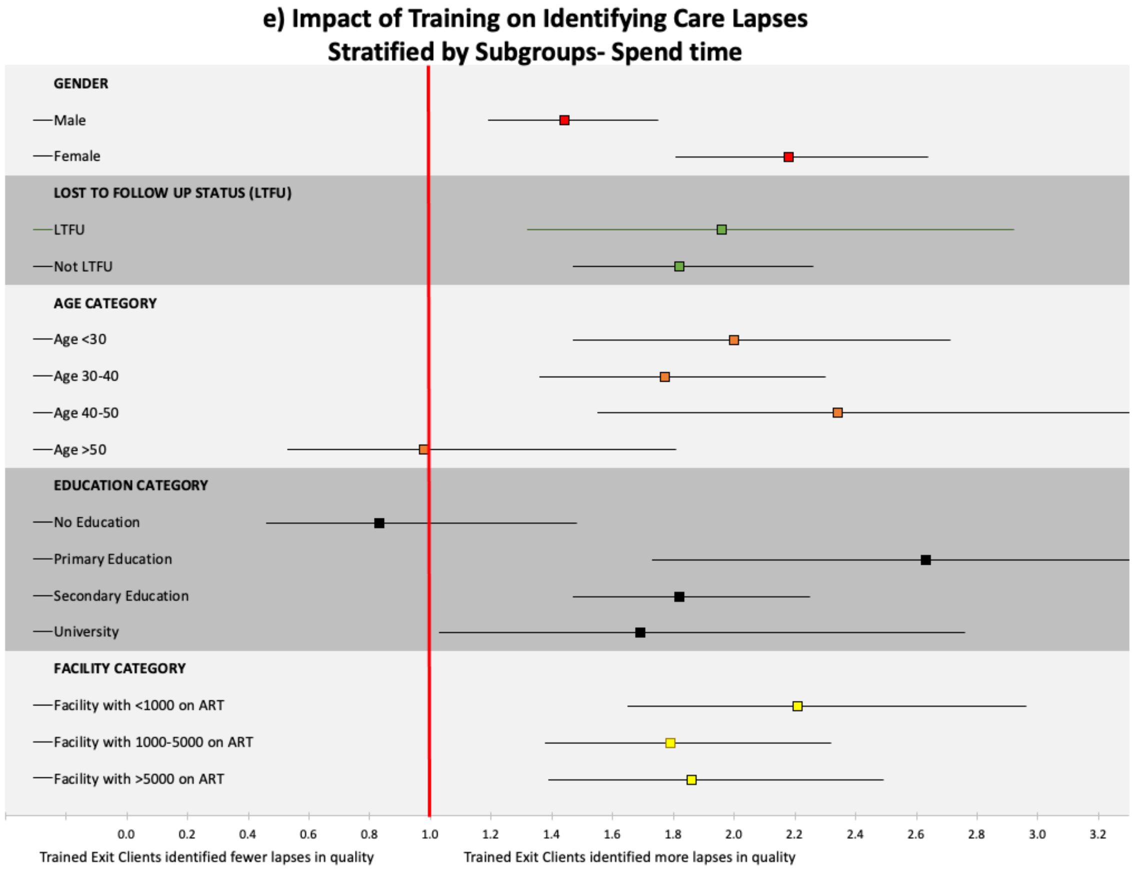
e) Impact of Training on Identifying Care Lapses
Stratified by Subgroups- Spend time
GENDER
Male
Female
LOST TO FOLLOW UP STATUS (LTFU)
LTFU
Not LTFU
AGE CATEGORY
Age <30
Age 30-40
Age 40-50
Age >50
EDUCATION CATEGORY
No Education
Primary Education
Secondary Education
University
FACILITY CATEGORY
Facility with <1000 on ART
Facility with 1000-5000 on ART
Facility with >5000 on ART
0.0 0.2 0.4 0.6 0.8 1.0 1.2 1.4 1.6 1.8 2.0 2.2 2.4 2.6 2.8 3.0 3.2
Trained Exit Clients identified fewer lapses in quality
Trained Exit Clients identified more lapses in quality

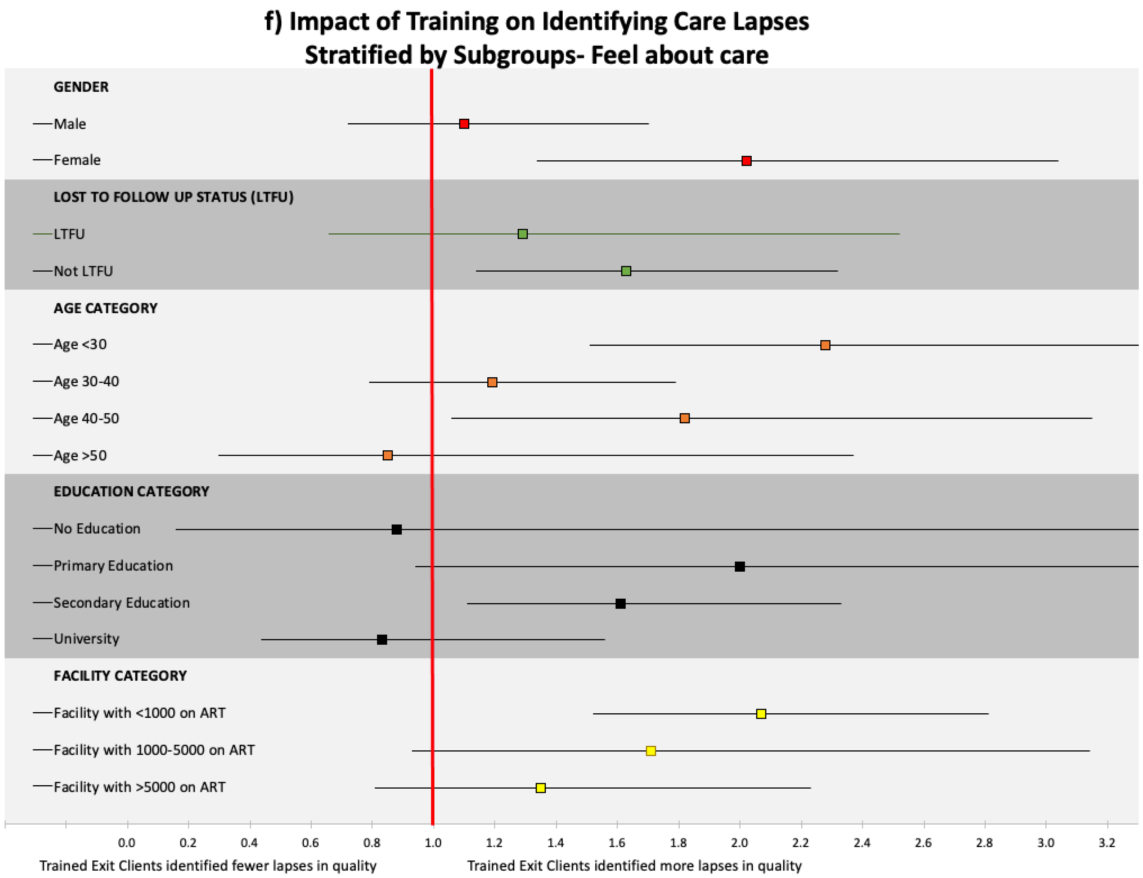

For peer review only

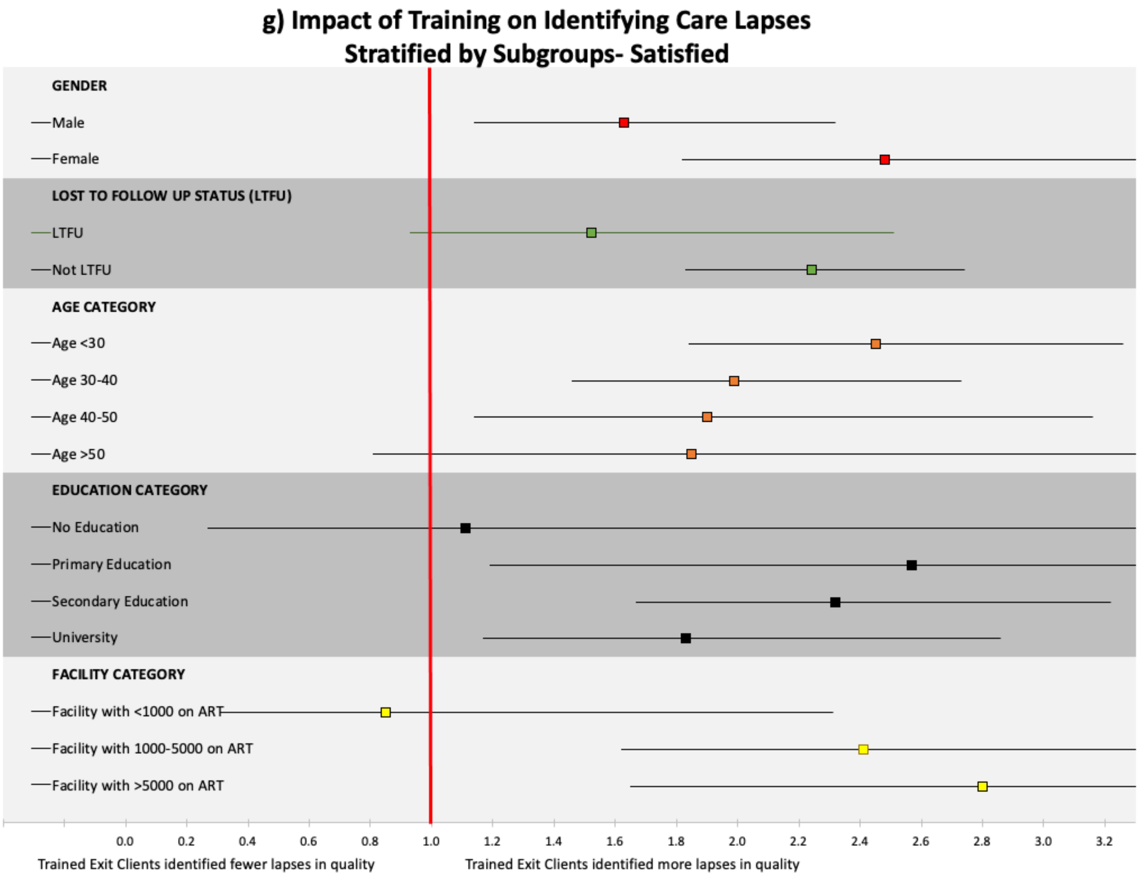

For peer review only

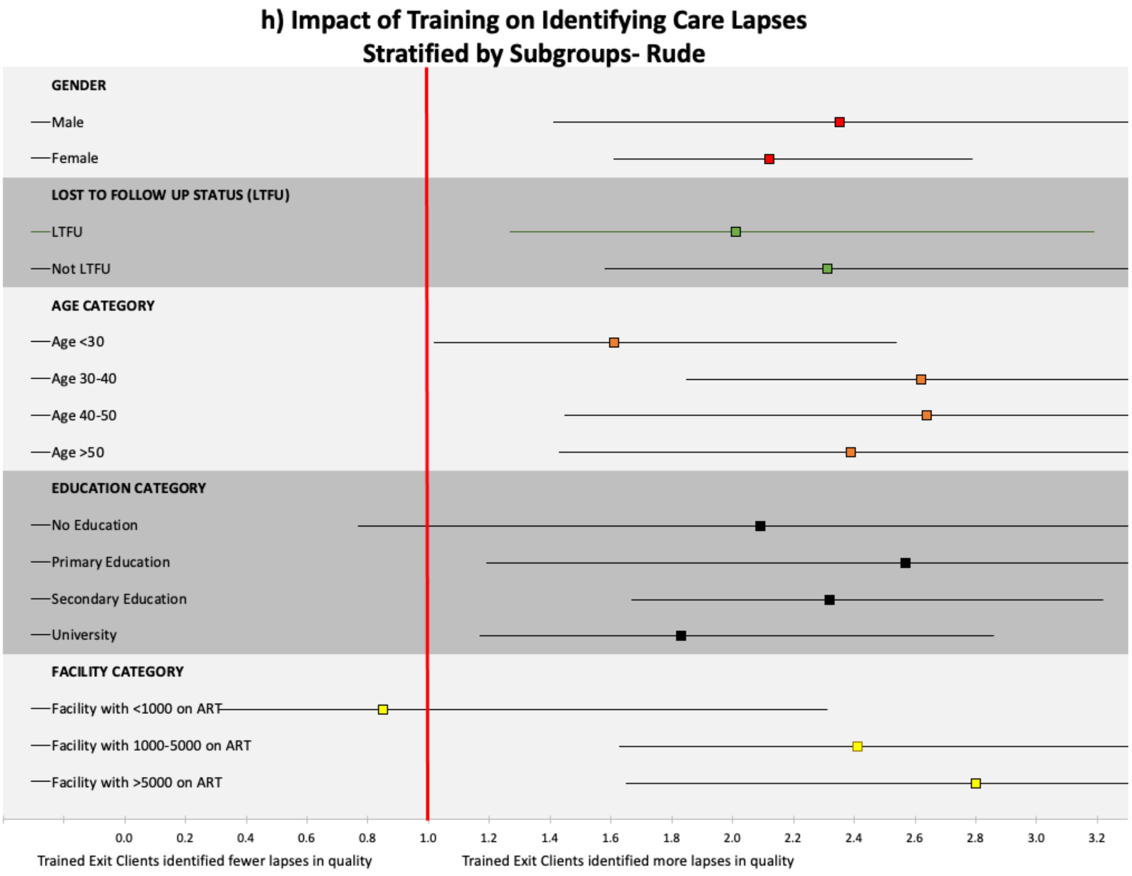
h) Impact of Training on Identifying Care Lapses
Stratified by Subgroups- Rude
GENDER
Male
Female
LOST TO FOLLOW UP STATUS (LTFU)
LTFU
Not LTFU
AGE CATEGORY
Age <30
Age 30-40
Age 40-50
Age >50
EDUCATION CATEGORY
No Education
Primary Education
Secondary Education
University
FACILITY CATEGORY
Facility with <1000 on ART
Facility with 1000-5000 on ART
Facility with >5000 on ART
0.0 0.2 0.4 0.6 0.8 1.0 1.2 1.4 1.6 1.8 2.0 2.2 2.4 2.6 2.8 3.0 3.2
Trained Exit Clients identified fewer lapses in quality
Trained Exit Clients identified more lapses in quality

view only

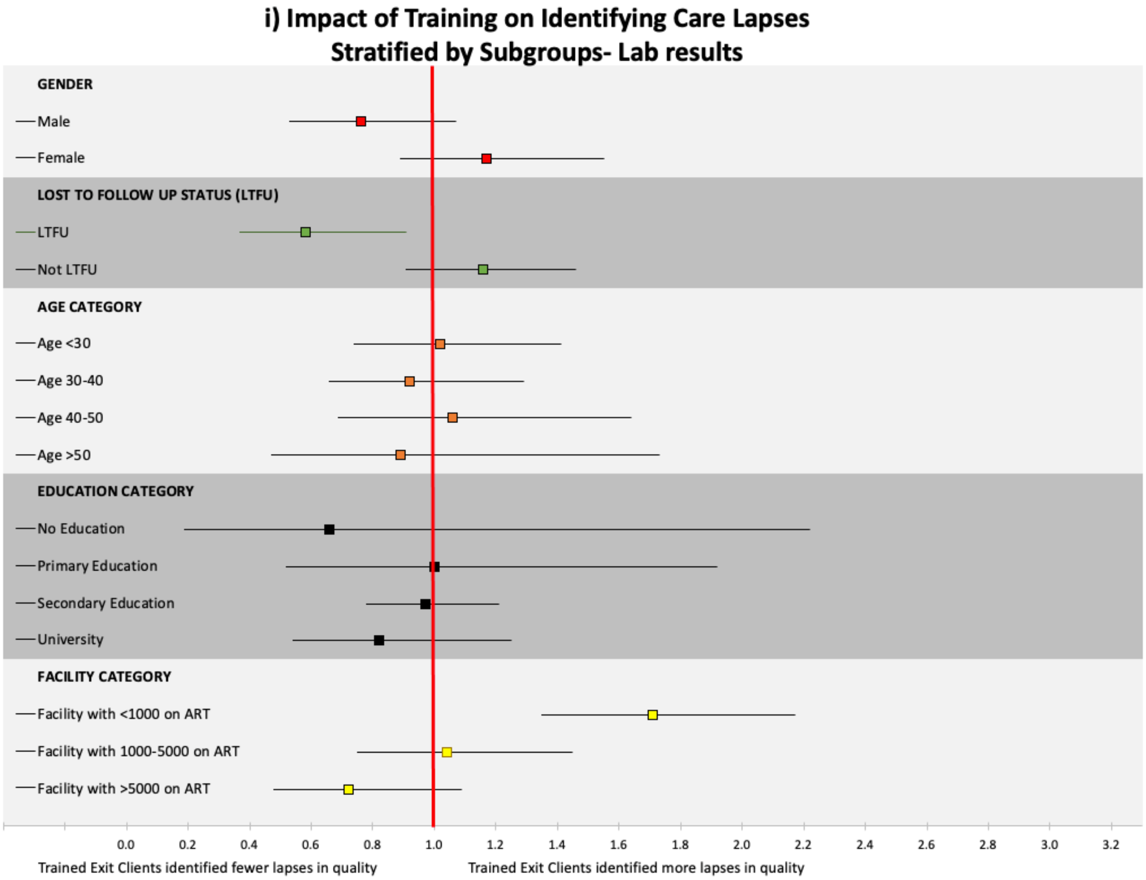
i) Impact of Training on Identifying Care Lapses
Stratified by Subgroups- Lab results
GENDER
Male
Female
LOST TO FOLLOW UP STATUS (LTFU)
LTFU
Not LTFU
AGE CATEGORY
Age <30
Age 30-40
Age 40-50
Age >50
EDUCATION CATEGORY
No Education
Primary Education
Secondary Education
University
FACILITY CATEGORY
Facility with <1000 on ART
Facility with 1000-5000 on ART
Facility with >5000 on ART
0.0 0.2 0.4 0.6 0.8 1.0 1.2 1.4 1.6 1.8 2.0 2.2 2.4 2.6 2.8 3.0 3.2
Trained Exit Clients identified fewer lapses in quality
Trained Exit Clients identified more lapses in quality

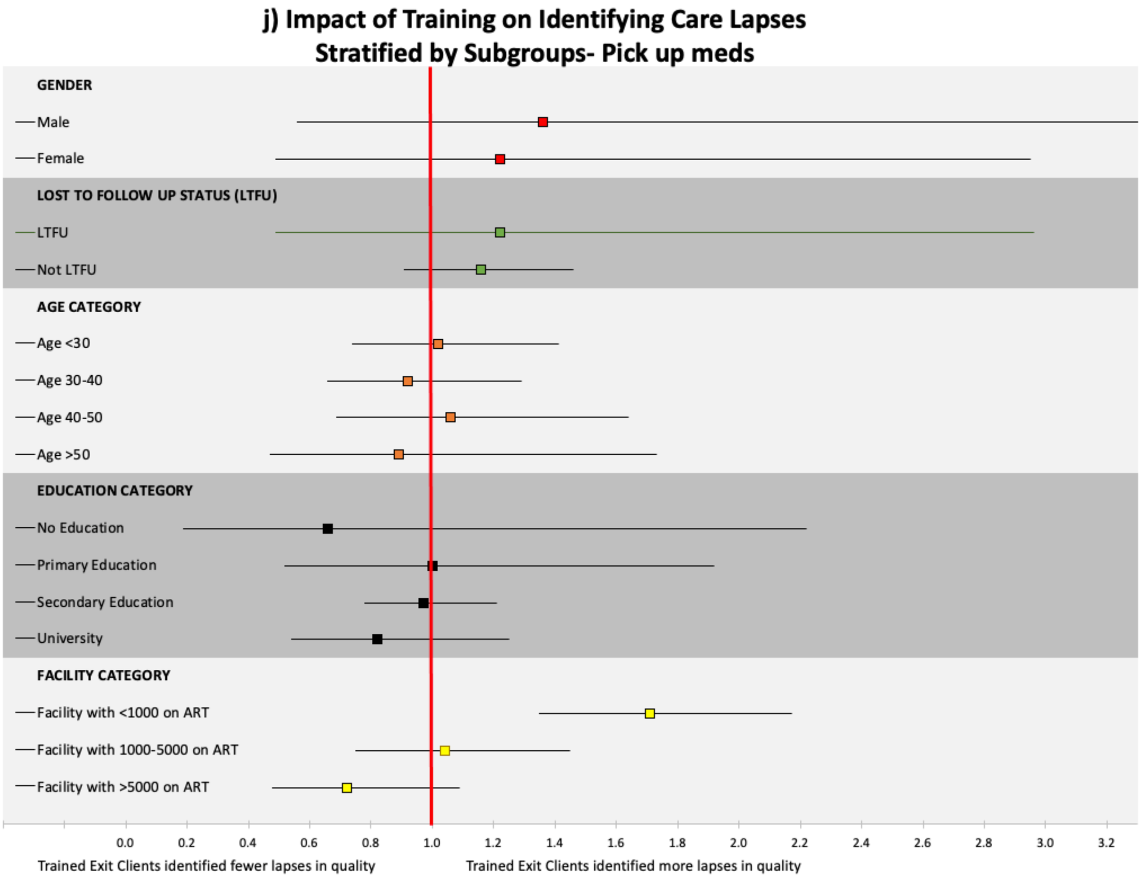
j) Impact of Training on Identifying Care Lapses
Stratified by Subgroups- Pick up meds
GENDER
Male
Female
LOST TO FOLLOW UP STATUS (LTFU)
LTFU
Not LTFU
AGE CATEGORY
Age <30
Age 30-40
Age 40-50
Age >50
EDUCATION CATEGORY
No Education
Primary Education
Secondary Education
University
FACILITY CATEGORY
Facility with <1000 on ART
Facility with 1000-5000 on ART
Facility with >5000 on ART
0.0 0.2 0.4 0.6 0.8 1.0 1.2 1.4 1.6 1.8 2.0 2.2 2.4 2.6 2.8 3.0 3.2
Trained Exit Clients identified fewer lapses in quality
Trained Exit Clients identified more lapses in quality

Supplementary Figure 2

Supplementary Figure 2. Bubble plot showing Trained Exit Sum Score vs Untrained Exit Sum Score. Each bubble represents a single facilities performance. Each bubble's size indicates the number of patients at each facility with larger bubbles corresponding to larger facilities. The horizontal position notes the Untrained Exit Sum Score for all questions against the facility, and the vertical position notes the Trained Exit sum score at the same facility.

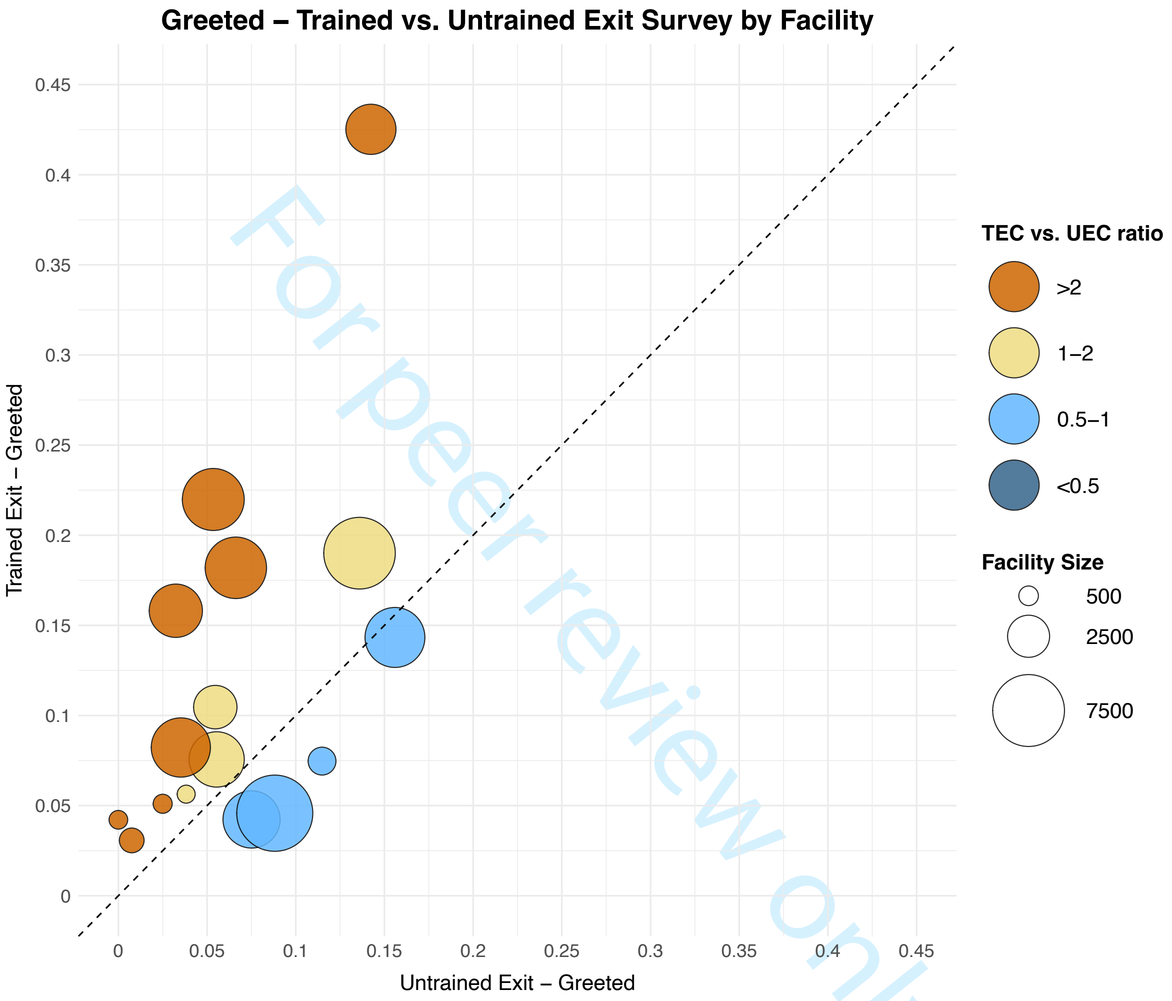

For peer review only

For peer review only - http://bmjopen.bmj.com/site/about/guidelines.xhtml

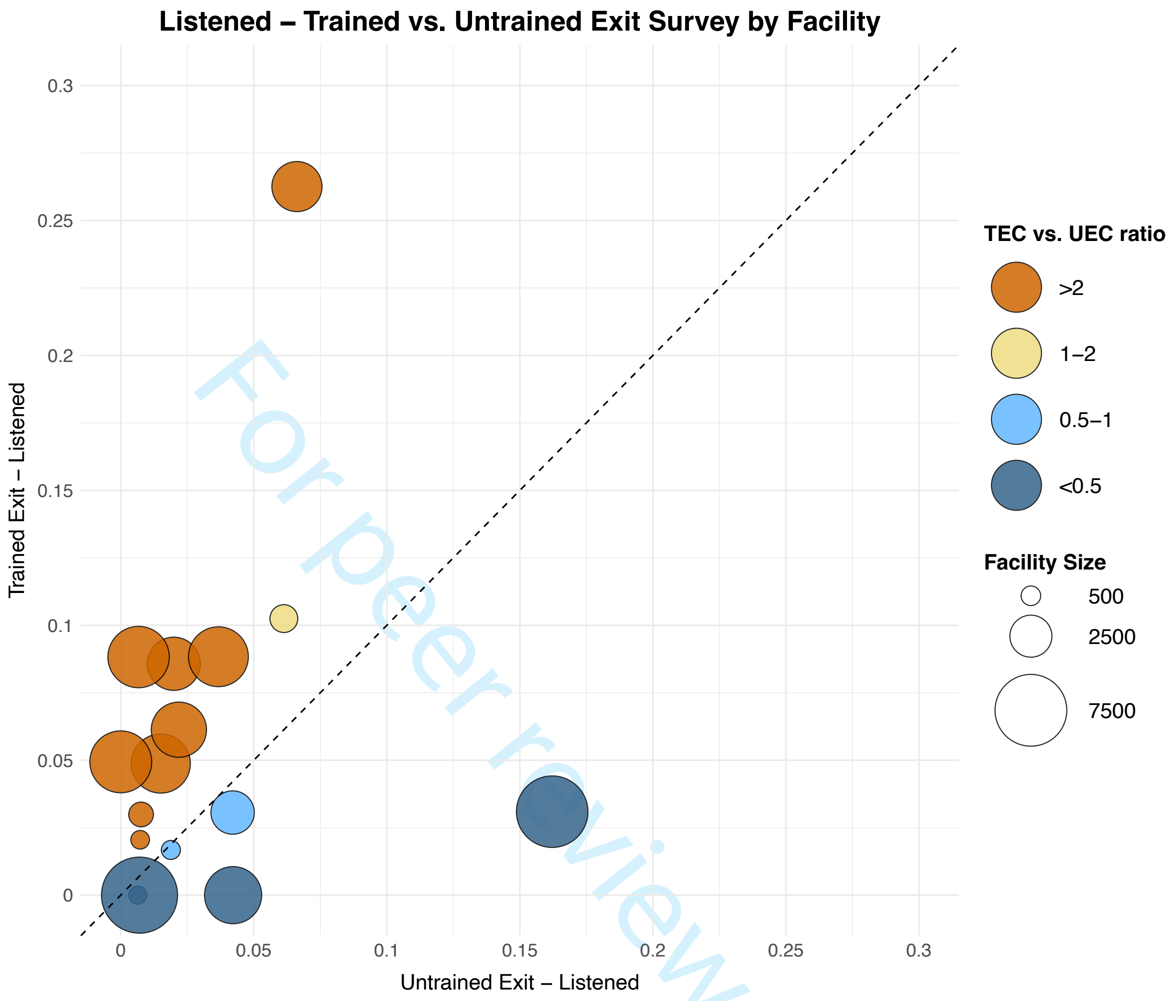

For peer review only

For peer review only - http://bmjopen.bmj.com/site/about/guidelines.xhtml

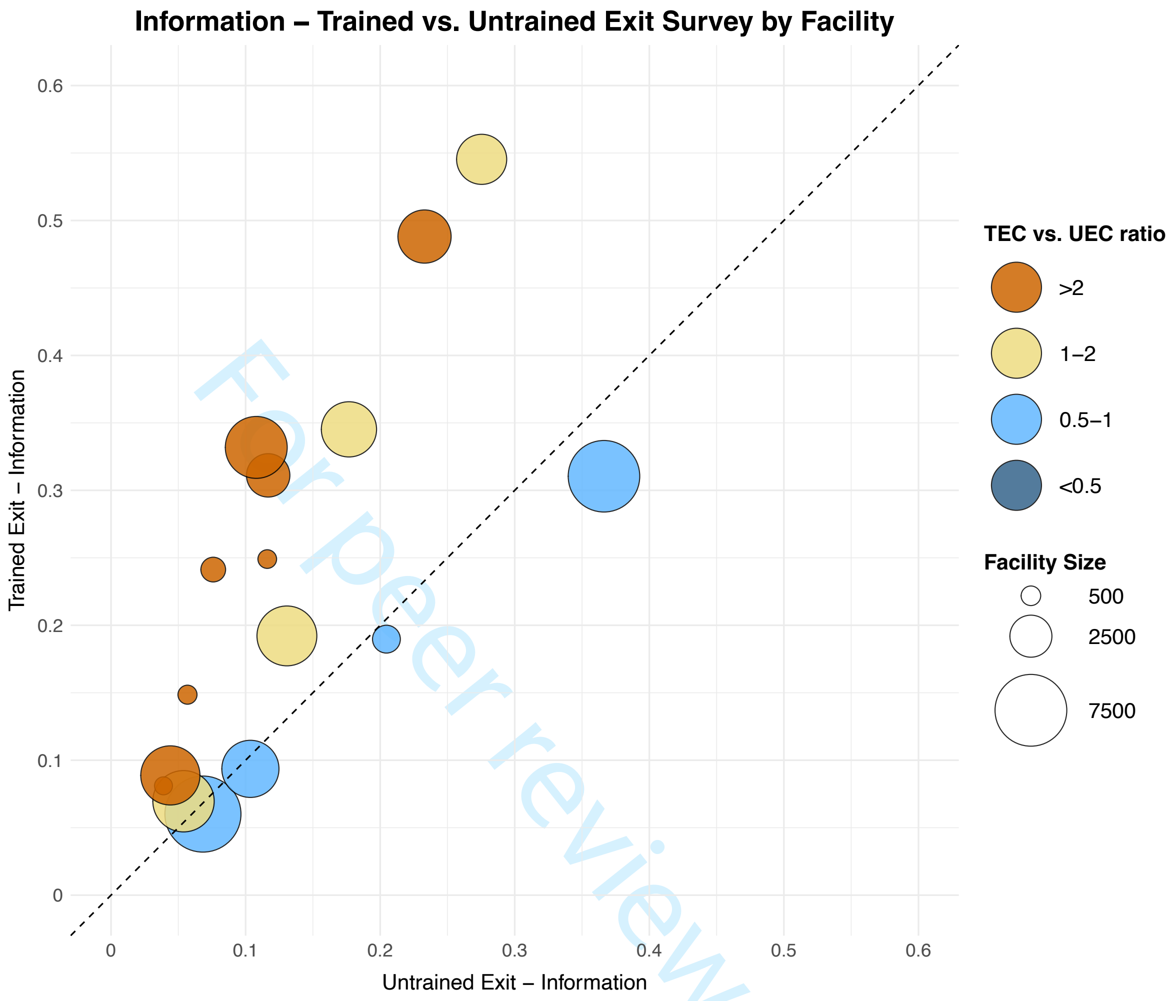

For peer review only

3

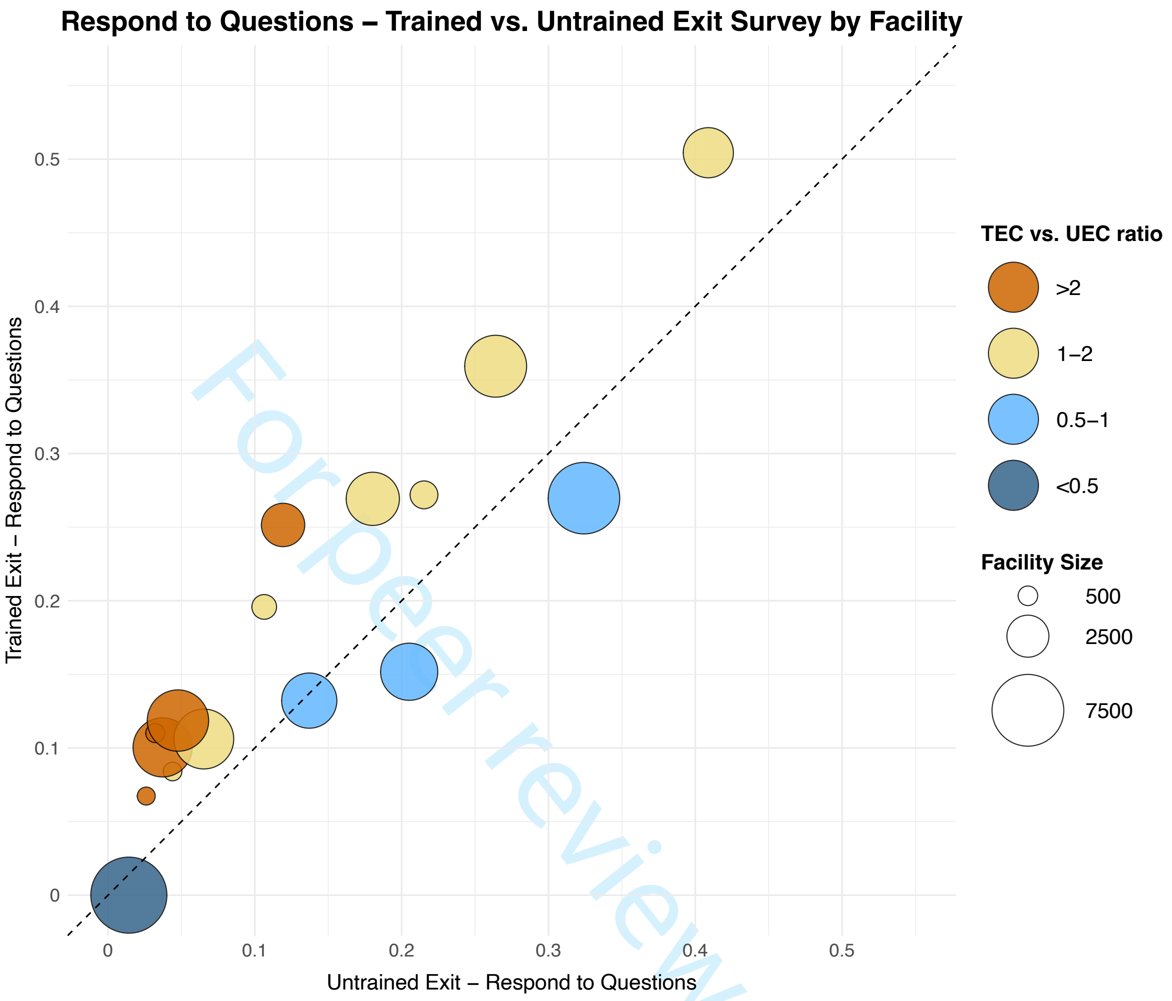

For peer review only

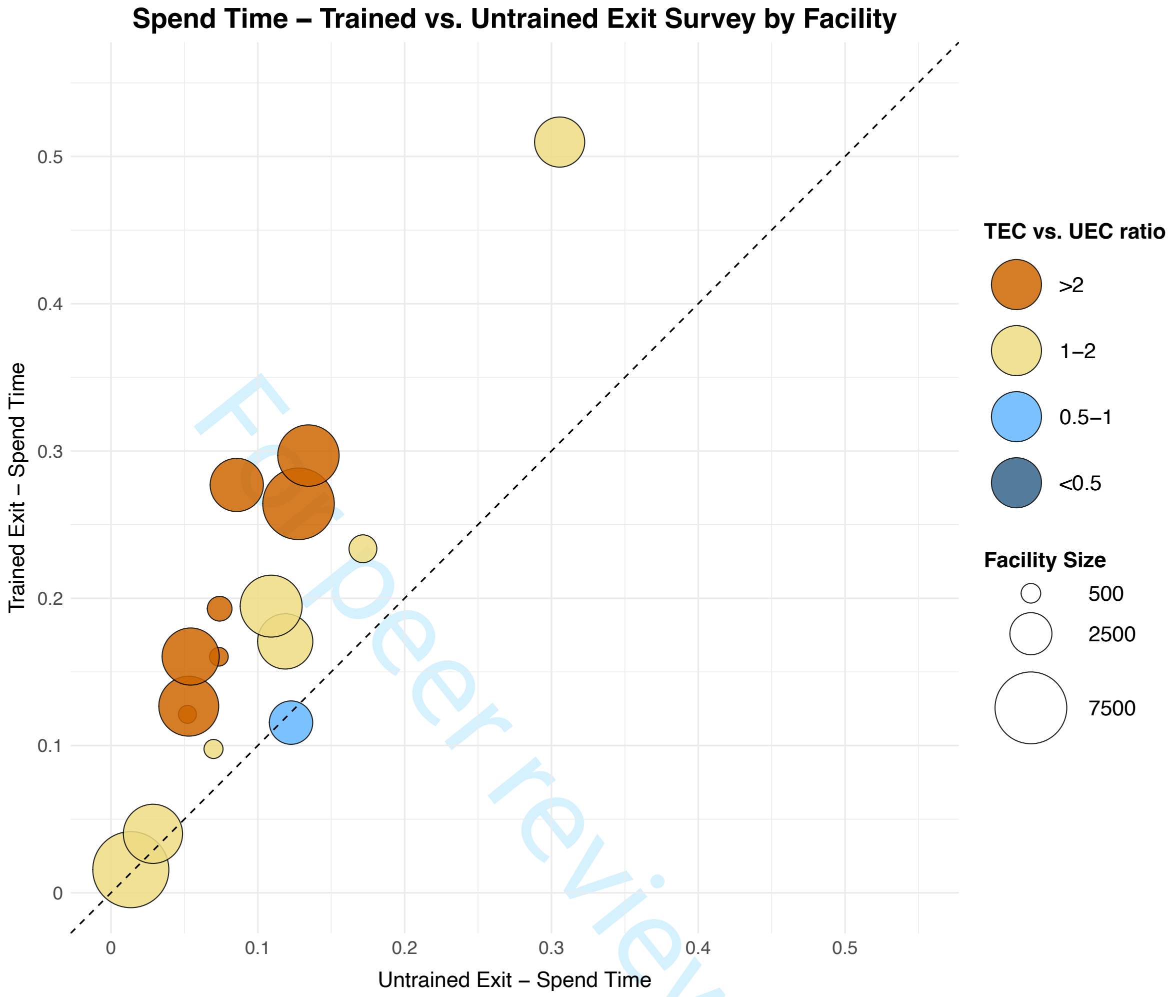

For peer review only

For peer review only - http://bmjopen.bmj.com/site/about/guidelines.xhtml

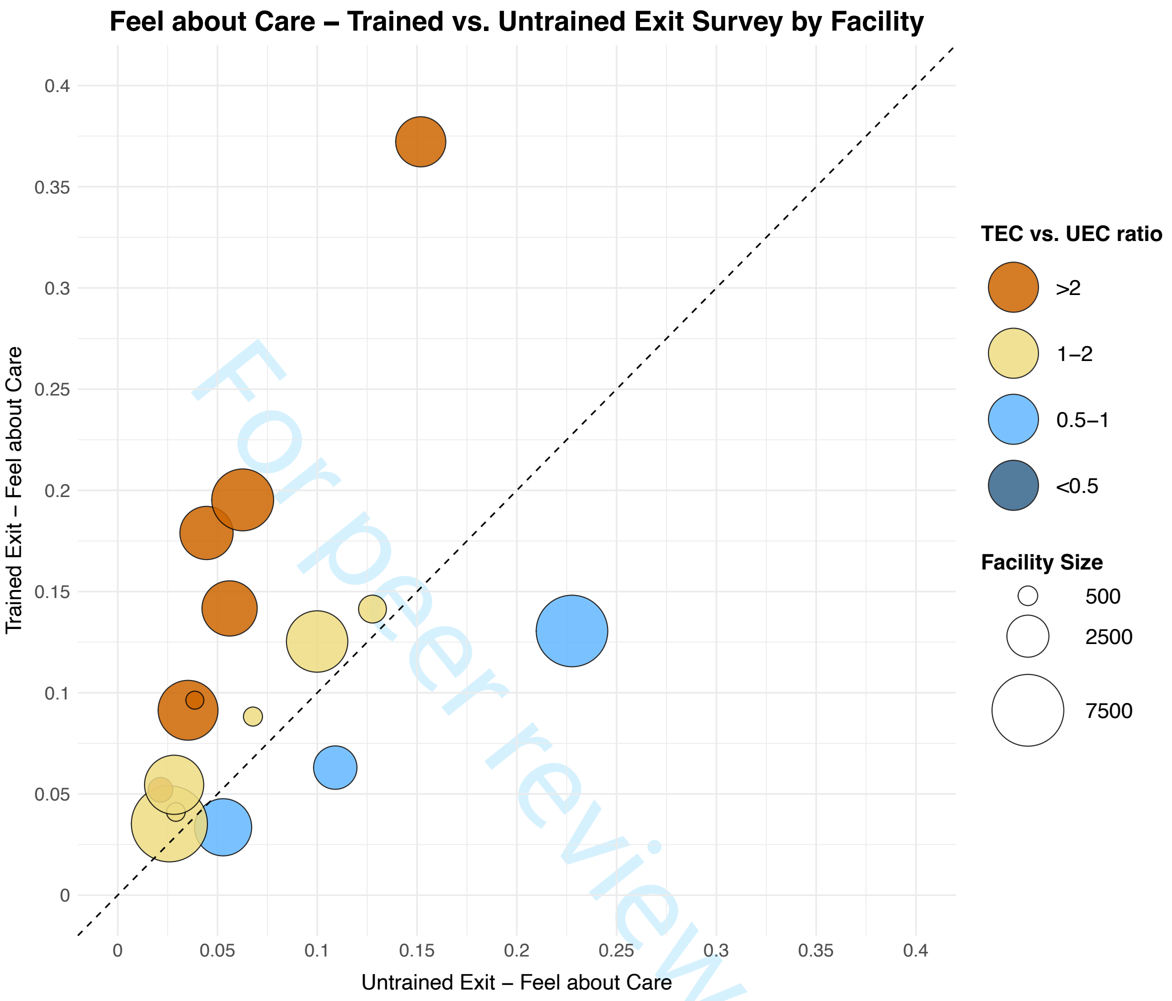

For peer review only

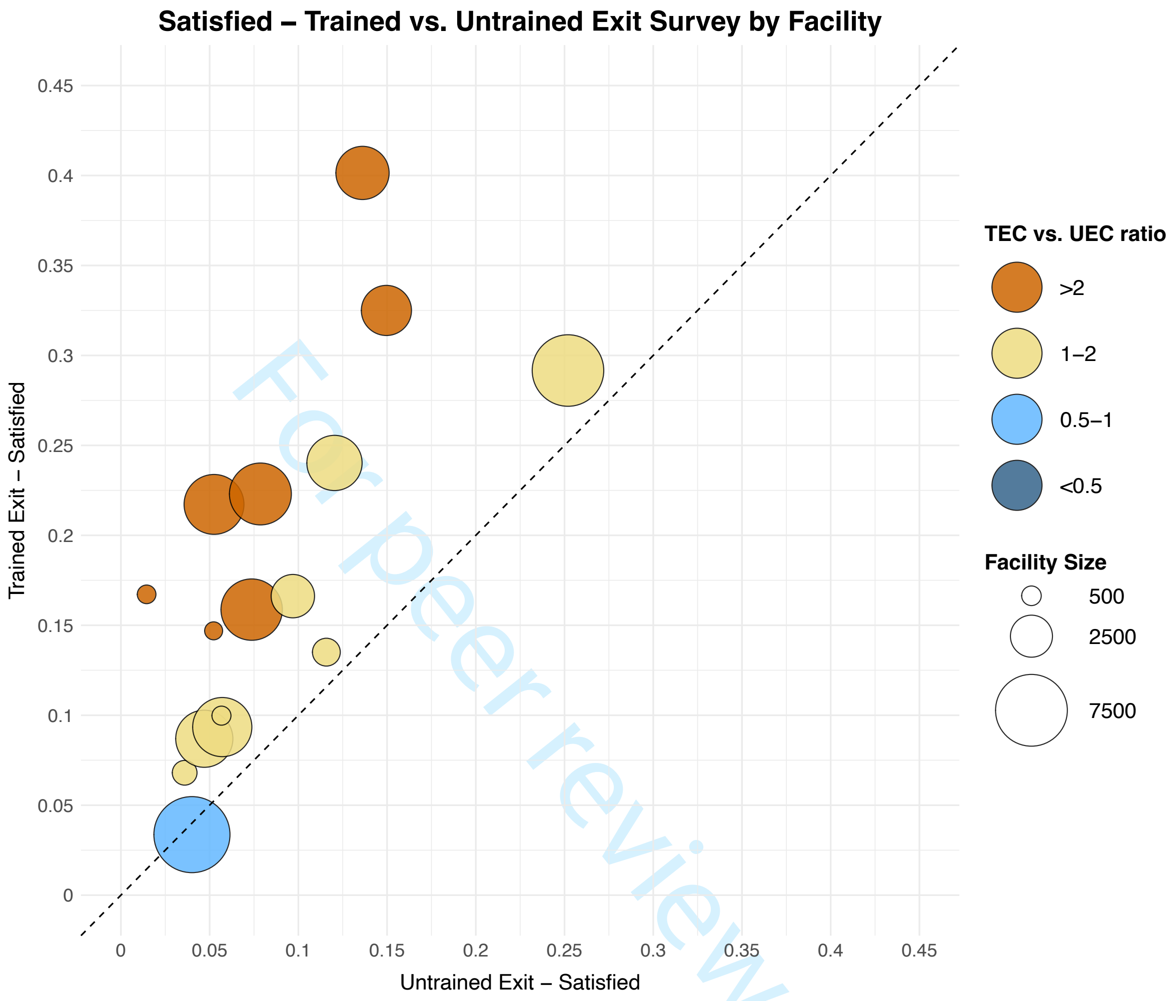

For peer review only

For peer review only - http://bmjopen.bmj.com/site/about/guidelines.xhtml

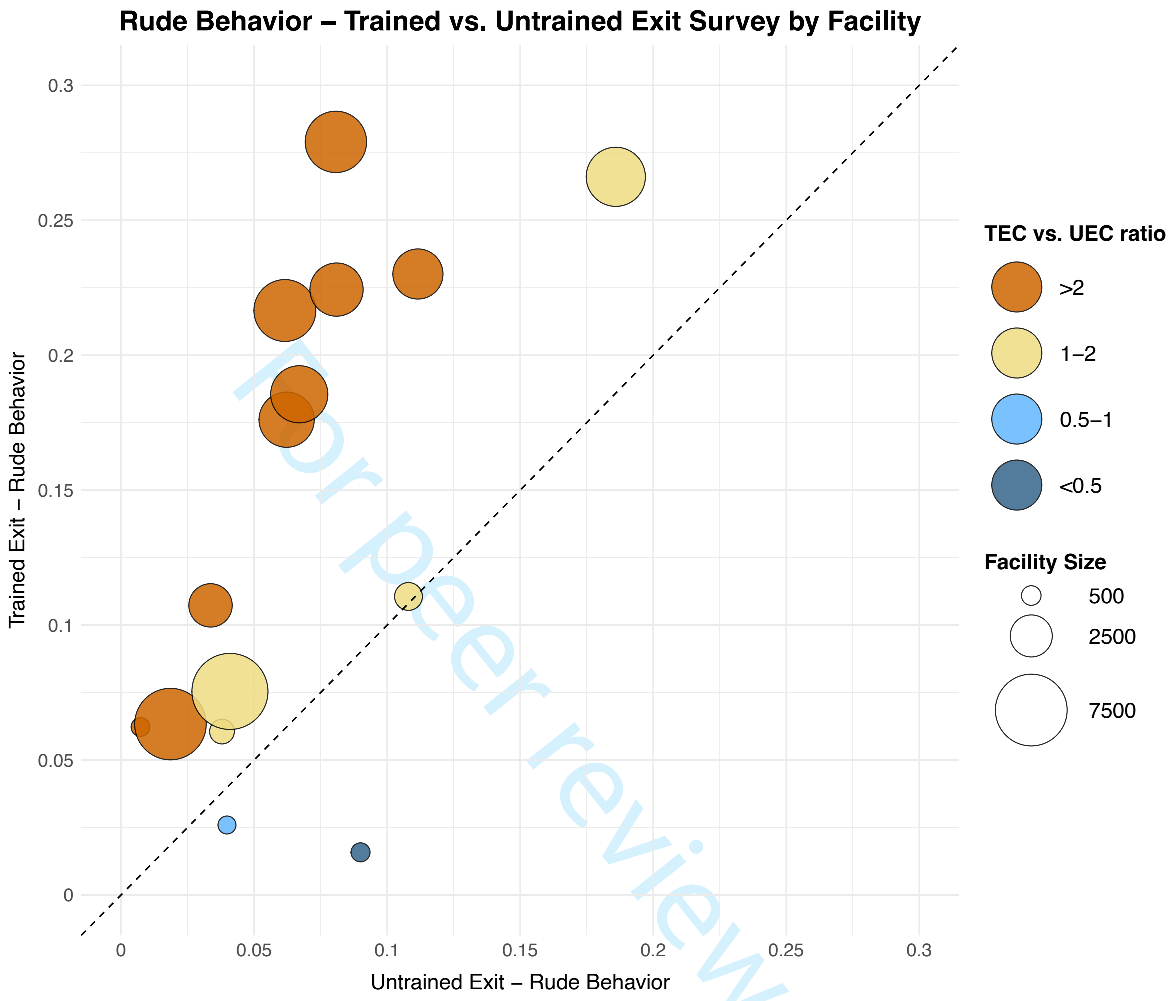

For peer review only

For peer review only - http://bmjopen.bmj.com/site/about/guidelines.xhtml

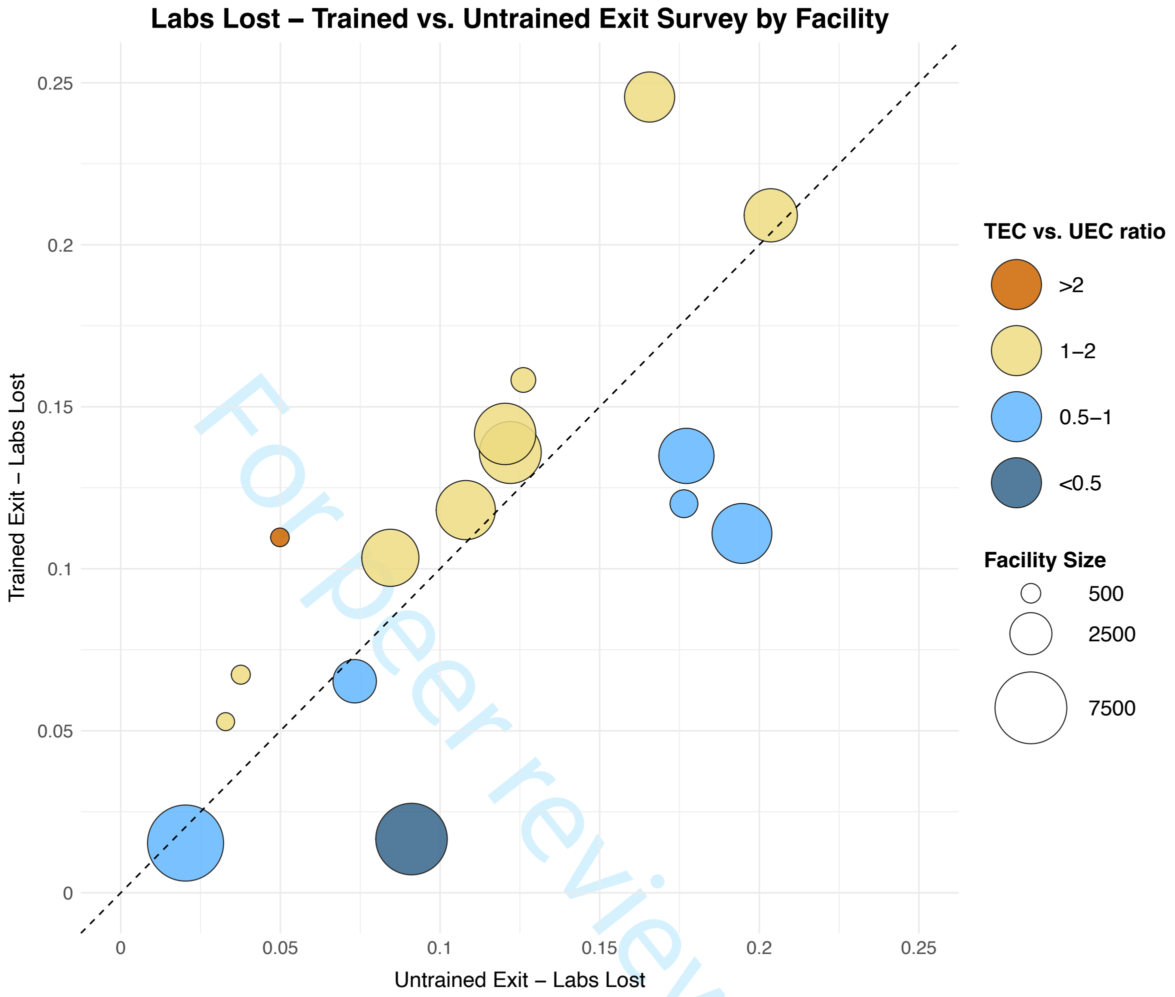

For peer review only

For peer review only - http://bmjopen.bmj.com/site/about/guidelines.xhtml

STROBE Statement—checklist of items that should be included in reports of observational studies

	Item No	Recommendation	Page No
**Title and abstract**	1	(*a*) Indicate the study's design with a commonly used term in the title or the abstract	1
		(*b*) Provide in the abstract an informative and balanced summary of what was done and what was found	1
**Introduction**			
Background/rationale	2	Explain the scientific background and rationale for the investigation being reported	3,4
Objectives	3	State specific objectives, including any prespecified hypotheses	3
**Methods**			
Study design	4	Present key elements of study design early in the paper	4,5
Setting	5	Describe the setting, locations, and relevant dates, including periods of recruitment, exposure, follow-up, and data collection	5
Participants	6	(*a*) *Cohort study*—Give the eligibility criteria, and the sources and methods of selection of participants. Describe methods of follow-up *Case-control study*—Give the eligibility criteria, and the sources and methods of case ascertainment and control selection. Give the rationale for the choice of cases and controls *Cross-sectional study*—Give the eligibility criteria, and the sources and methods of selection of participants	5,6
		(*b*) *Cohort study*—For matched studies, give matching criteria and number of exposed and unexposed *Case-control study*—For matched studies, give matching criteria and the number of controls per case	N/A
Variables	7	Clearly define all outcomes, exposures, predictors, potential confounders, and effect modifiers. Give diagnostic criteria, if applicable	5,7,8
Data sources/ measurement	8*	For each variable of interest, give sources of data and details of methods of assessment (measurement). Describe comparability of assessment methods if there is more than one group	*5-7*
Bias	9	Describe any efforts to address potential sources of bias	4
Study size	10	Explain how the study size was arrived at	8
Quantitative variables	11	Explain how quantitative variables were handled in the analyses. If applicable, describe which groupings were chosen and why	7
Statistical methods	12	(*a*) Describe all statistical methods, including those used to control for confounding	7
		(*b*) Describe any methods used to examine subgroups and interactions	7
		(*c*) Explain how missing data were addressed	N/A
		(*d*) *Cohort study*—If applicable, explain how loss to follow-up was addressed *Case-control study*—If applicable, explain how matching of cases and controls was addressed *Cross-sectional study*—If applicable, describe analytical methods taking account of sampling strategy	N/A
		(*e*) Describe any sensitivity analyses	N/A

Continued on next page

For peer review only - http://bmjopen.bmj.com/site/about/guidelines.xhtml

**Results**			
Participants	13*	(a) Report numbers of individuals at each stage of study—eg numbers potentially eligible, examined for eligibility, confirmed eligible, included in the study, completing follow-up, and analysed	9
		(b) Give reasons for non-participation at each stage	9
		(c) Consider use of a flow diagram	N/A
Descriptive data	14*	(a) Give characteristics of study participants (eg demographic, clinical, social) and information on exposures and potential confounders	9, 10
		(b) Indicate number of participants with missing data for each variable of interest	9,10
		(c) *Cohort study*—Summarise follow-up time (eg, average and total amount)	N/A
Outcome data	15*	*Cohort study*—Report numbers of outcome events or summary measures over time	N/A
		Case-control study—Report numbers in each exposure category, or summary measures of exposure	N/A
		Cross-sectional study—Report numbers of outcome events or summary measures	10
Main results	16	(*a*) Give unadjusted estimates and, if applicable, confounder-adjusted estimates and their precision (eg, 95% confidence interval). Make clear which confounders were adjusted for and why they were included	7,11
		(*b*) Report category boundaries when continuous variables were categorized	
		(*c*) If relevant, consider translating estimates of relative risk into absolute risk for a meaningful time period	
Other analyses	17	Report other analyses done—eg analyses of subgroups and interactions, and sensitivity analyses	12,14
**Discussion**			
Key results	18	Summarise key results with reference to study objectives	14,15
Limitations	19	Discuss limitations of the study, taking into account sources of potential bias or imprecision. Discuss both direction and magnitude of any potential bias	18
Interpretation	20	Give a cautious overall interpretation of results considering objectives, limitations, multiplicity of analyses, results from similar studies, and other relevant evidence	19,20
Generalisability	21	Discuss the generalisability (external validity) of the study results	18
**Other information**			
Funding	22	Give the source of funding and the role of the funders for the present study and, if applicable, for the original study on which the present article is based	20

*Give information separately for cases and controls in case-control studies and, if applicable, for exposed and unexposed groups in cohort and cross-sectional studies.

Note: An Explanation and Elaboration article discusses each checklist item and gives methodological background and published examples of transparent reporting. The STROBE checklist is best used in conjunction with this article (freely available on the Web sites of PLoS Medicine at http://www.plosmedicine.org/, Annals of Internal Medicine at http://www.annals.org/, and Epidemiology at http://www.epidem.com/). Information on the STROBE Initiative is available at www.strobe-statement.org.

For peer review only

BMJ Open

A Comparison of Patient Exit Interviews to Unannounced Standardised Patients for Assessing HIV Service Delivery in Zambia nested within a Cluster Randomised Trial

Journal:	*BMJ Open*
Manuscript ID	bmjopen-2022-069086.R2
Article Type:	Original research
Date Submitted by the Author:	19-Jun-2023
Complete List of Authors:	Sikombe, Kombatende; Center for Infectious Disease Research in Zambia, Implementation Science Unit; London School of Hygiene and Tropical Medicine Faculty of Public Health and Policy, Department of Public Health, Environments and Society<br>Pry, Jake; Center for Infectious Disease Research in Zambia, Implementation Science Unit<br>Mody, Aaloke; Washington University in St Louis School of Medicine, Internal Medicine<br>Rice, Brian ; London School of Hygiene and Tropical Medicine Faculty of Public Health and Policy, Department of Public Health, Environments and Society<br>Bukankala, Chama; Center for Infectious Disease Research in Zambia, Research<br>Eshun-Wilson, Ingrid ; Washington University in St Louis School of Medicine, Internal Medicine<br>Mutale, Jacob; Center for Infectious Disease Research in Zambia, Implementation Science Unit<br>Simbeza, Sandra; Center for Infectious Disease Research in Zambia, Implementation Science Unit<br>Beres, Laura K ; Johns Hopkins University Bloomberg School of Public Health, Department of International Health<br>Mukamba, Njekwa; Center for Infectious Disease Research in Zambia, Social and Behavioural Science Research Group<br>Mukumbwa-Mwenechanya, Mpande; Center for Infectious Disease Research in Zambia, Implementation Science Unit<br>Mwamba, Daniel; Center for Infectious Disease Research in Zambia, Clinical Care Services<br>Sharma, Anjali; Center for Infectious Disease Research in Zambia, Social and Behavioural Science Research Group<br>Wringe, Alison; London School of Hygiene and Tropical Medicine Faculty of Epidemiology and Population Health<br>Hargreaves, James; London School of Hygiene and Tropical Medicine Faculty of Public Health and Policy, Department of Public Health, Environments and Society<br>Bolton-Moore, Carolyn; Center for Infectious Disease Research in Zambia, Clinical Care Services ; The University of Alabama at Birmingham Department of Medicine, Infectious Disease<br>Holmes, Charles; Georgetown University Medical Center, Center for Innovation in Global Health

	Sikazwe, Izukanji; Center for Infectious Disease Research in Zambia, Implementation Science Unit<br>Geng, Elvin; Washington University in St Louis School of Medicine, Internal Medicine
**Primary Subject Heading**:	Health services research
Secondary Subject Heading:	Epidemiology, Global health, Health services research, HIV/AIDS, Patient-centred medicine
Keywords:	Public health < INFECTIOUS DISEASES, Organisation of health services < HEALTH SERVICES ADMINISTRATION & MANAGEMENT, Quality in health care < HEALTH SERVICES ADMINISTRATION & MANAGEMENT, HIV & AIDS < INFECTIOUS DISEASES, PUBLIC HEALTH

I, the Submitting Author has the right to grant and does grant on behalf of all authors of the Work (as defined in the below author licence), an exclusive licence and/or a non-exclusive licence for contributions from authors who are: i) UK Crown employees; ii) where BMJ has agreed a CC-BY licence shall apply, and/or iii) in accordance with the terms applicable for US Federal Government officers or employees acting as part of their official duties; on a worldwide, perpetual, irrevocable, royalty-free basis to BMJ Publishing Group Ltd ("BMJ") its licensees and where the relevant Journal is co-owned by BMJ to the co-owners of the Journal, to publish the Work in this journal and any other BMJ products and to exploit all rights, as set out in our licence.

The Submitting Author accepts and understands that any supply made under these terms is made by BMJ to the Submitting Author unless you are acting as an employee on behalf of your employer or a postgraduate student of an affiliated institution which is paying any applicable article publishing charge ("APC") for Open Access articles. Where the Submitting Author wishes to make the Work available on an Open Access basis (and intends to pay the relevant APC), the terms of reuse of such Open Access shall be governed by a Creative Commons licence – details of these licences and which Creative Commons licence will apply to this Work are set out in our licence referred to above.

Other than as permitted in any relevant BMJ Author's Self Archiving Policies, I confirm this Work has not been accepted for publication elsewhere, is not being considered for publication elsewhere and does not duplicate material already published. I confirm all authors consent to publication of this Work and authorise the granting of this licence.

For peer review only

A Comparison of Patient Exit Interviews to Unannounced Standardised Patients for Assessing HIV Service Delivery in Zambia nested within a Cluster Randomised Trial

Kombatende Sikombe[1,2], Jake M Pry[1,3], Aaloke Mody[4], Brian Rice, Chama Bukankala[1], Ingrid Eshun-Wilson[4], Jacob Mutale[1], Sandra Simbeza[1], Laura K Beres[5], Njekwa Mukamba[1], Mpande Mukumbwa-Mwenechanya[1], Daniel Mwamba[1], Anjali Sharma[1], Alison Wringe[6], James Hargreaves[1], Carolyn Bolton Moore[1,7], Charles B Holmes[8], Izukanji Sikazwe[1], Elvin Geng[4]

[1]Centre for Infectious Disease Research in Zambia, Lusaka, Zambia, [2]Faculty of Public Health and Policy, London School of Hygiene & Tropical Medicine, London, UK, [3]Department of Public Health Sciences, University of California, Davis, CA, USA, [4]Division of Infectious Diseases, Washington University in St Louis, St. Louis, MO, USA, [5]Department of International Health, Johns Hopkins Bloomberg School of Public Health, Baltimore, MD, USA, [6]Faculty of Epidemiology and Population Health, London School of Hygiene and Tropical Medicine, London, UK, [7]Department of Medicine, School of Medicine, University of Alabama at Birmingham, Birmingham, AL, USA, [8]Center for Innovation in Global Health, Georgetown University, Washington, DC, USA.

Corresponding Author: Kombatende Sikombe
Affiliations: Centre for Infectious Disease Research in Zambia, Lusaka, Zambia, Faculty of Public Health and Policy, London School of Hygiene & Tropical Medicine, London, UK
Email Address: kombatende.sikombe@cidrz.org kombatende.sikombe@lshtm.ac.uk

For peer review only - http://bmjopen.bmj.com/site/about/guidelines.xhtml

ABSTRACT

Objectives: To compare unannounced standardised patient approach (e.g., mystery clients) to typical exit interviews for assessing patient experiences in Human Immunodeficiency Virus (HIV) care (e.g., unfriendly providers, long-waiting times). We hypothesize standardized patients would report more negative experiences than typical exit interviews affected by social desirability bias.

Setting: Cross-sectional surveys in 16 government-operated HIV primary care clinics in Lusaka, Zambia providing antiretroviral-therapy (ART).

Participants: 3526 participants aged ≥18 years receiving ART participated in the exit surveys between August 2019 and November 2021.

Intervention: Systematic sample (every k^{th} file) of patients in clinic waiting area willing to be trained received pre-visit training and post-visit interviews. Providers were unaware of trained patients.

Outcome measures: We compared patient experience among patients that received a brief training prior to their care visit (explaining each patient experience construct in the exit survey, being anonymous, without manipulating behaviour) with those who did not undergo training on the survey prior to their visit.

Results: Among 3526 participants who participated in exit surveys, 2415 were untrained (56% female, median age 40 (inter quartile range [IQR]:32-47)) and 1111 were trained (50% female, median age 37 (IQR:31-45)). Compared to untrained, trained patients were more likely to report a negative care experience overall (adjusted Prevalence Ratio aPR for aggregate sum score: 1.64 [95% CI:1.39-1.94]), with a greater proportion reporting feeling unwelcomed by providers ([aPR]: 1.71 [95% CI:1.20-2.44]) and witnessing providers behaving rude (aPR: 2.28 [95% CI:1.63-3.19]).

Conclusion: Trained patients were more likely to identify sub-optimal care. They may have understood the items solicited better or felt empowered to be more critical., We trained existing patients, unlike studies that use "standardised patients" drawn from outside the patient population. This low-cost strategy could improve patient-centred service delivery elsewhere.

Trial registration: Assessment was nested within a parent study. www.pactr.org registered the parent study (PACTR202101847907585).

For peer review only

STRENGTHS AND LIMITATIONS OF THIS STUDY

- This study utilised standardised patients (SP) to assess chronic care in which actual, rather than simulated patients were trained before their upcoming clinic visits.
- Traditional SP techniques require a trained simulated patient to visit multiple clinics, a strategy more appropriate for episodic care.
- Modified SP approaches can address the challenge of integrating patient experience into routine public health, a crucial quality indicator for governments and funders.
- We trained patients to assess care quality (e.g., waiting times, rude providers), and compared their responses to traditional untrained exit surveys in 16 facilities in Zambia.
- Training remains challenging as we did not include participants who were illiterate, had poor recall ability, or potentially struggled with comprehension.

BACKGROUND

For peer review only - http://bmjopen.bmj.com/site/about/guidelines.xhtml

Because of improved testing, linkage, and treatment to meet the global 95-95-95 treatment targets (95% of HIV-positive patients know their status, 95% are on treatment, and 95% have suppressed viral loads) [1], retention in care have become a major obstacle to improving Human Immunodeficiency Virus (HIV) treatment outcomes, and health systems in low-income settings like Zambia, have sought to shift their public health response by designing and delivering high quality and patient-centred HIV care [2–7]. Efforts to improve service quality and patient experience require systematic measurement of the patient experience to guide facility responses as poor patient experience has been shown to lead to disengagement from care [8–12]. Health policymakers and donors, such as the President's Emergency Plan for AIDS Relief (PEPFAR), have invested in clinical metrics to assess care quality in Zambia and the wider region, but to a lesser extent in non-clinical metrics like patient experience [13]. These metrics can be critical for guiding efforts to improve retention in care by ensuring an informed response to improving quality of care and patient centredness.

Accurate and pragmatic measurement of the patient experience poses a range of challenges. Patient experience exit surveys are prone to social desirability bias because of power dynamics in health care. Empirical studies of satisfaction, for example, are widely believed to over-estimate patient satisfaction [14]. This may be particularly true where provider-patient relationships are traditional and hierarchical. Delaying surveys for some time after the encounter is theorised to ameliorate social desirability bias, but in turn may exacerbate bias due to simple inability to remember — thus creating recall bias [7,15]. Other methods such as direct clinical observations of care pose practical difficulties [14,16]. For example, direct observations may be intrusive and therefore may not reflect everyday functionality of a health facility. Care provided under direct

For peer review only - http://bmjopen.bmj.com/site/about/guidelines.xhtml

observations may be of higher quality as behaviour may be influenced by observation, a phenomenon often known as the “Hawthorne effect” [14,16].

Standardised patients (SP), also known as “mystery clients” or “simulated patients” have largely been used to assess quality of care in developed countries, as well as in assessing customer service in the retail industry[17]. SP can be resource-intensive and require training, but reduce potential for recall bias, social desirability, bias, and Hawthorne effects, providing an opportunity for optimal assessment of patient satisfaction among people receiving HIV care [7,15,18]. They have largely been used for episodic care where a highly skilled and well-trained person poses as a client by making one visit to multiple facilities. This approach holds promise for assessing the patient experience in HIV care but poses pragmatic challenges when assessing the quality of chronic care in which a patient makes multiple visits and may compromise efficiency at, already overburdened, facilities [19–24]. In this study, we report on the development and evaluation of a modified SP approach in which we trained real patients (trained exit clients - TEC) to report on certain characteristics of encounters, and rate key components of care such as waiting times, communication, respectfulness of providers, and privacy.

METHODS

Study design & setting

This study seeks to compare two different methods for assessing patient experience: standard exit survey and those reported by patients who had a brief training on the items before the clinical encounter and to whom the clinic was blinded. The assessment was nested within a parent study: the Leveraging Person-Centred Public Health (PCPH) to improve HIV outcomes in Zambia study (www.pactr.org PACTR202101847907585), a Stepped Wedge Cluster Randomised Trial that

For peer review only - http://bmjopen.bmj.com/site/about/guidelines.xhtml

occurred between August 2019 and November 2021. The aim of the overall PCPH study was to assess the impact of introducing health care workers (HCW) to a patient-centred care (PCC) curriculum and mentoring them on PCC principles to improve retention and viral suppression in HIV care. In this nested sub-study, we compared cross sectional surveys of patient experience using two different survey methods: adapted standardised approach (Trained Exit Clients) vs traditional exit surveys.

Population

The sub-study reported here included 16 health facilities in Lusaka, Zambia, operated by the Ministry of Health (MOH) and receiving technical assistance from the Centre for Infectious Diseases Research in Zambia (CIDRZ) - a Zambian non-governmental organisation (NGO) as well as a part of the larger parent study. We surveyed adults aged 18 years and over who were accessing antiretroviral therapy (ART) at study facilities. Exit survey patients were selected in a systematic sample (every k^{th} file varied by facility size) at the time of exit from the clinic. Trained patients were recruited in the waiting room for their visit, underwent a brief training, and then answered survey questions on exit from their encounter. Participants attending an HIV care visit on the day, able to recall events and comprehend study participant recruitment details (as assessed using the comprehension assessment tool) and able to read and write (assessed using literacy tool) were eligible for inclusion.

Procedures and Measurements

Survey Instrument

For both survey methods, we developed a patient experience instrument based on a previously validated tool developed and used in Kenya: The Wachira Physician-Patient Communication

For peer review only - http://bmjopen.bmj.com/site/about/guidelines.xhtml

For peer review only

Behaviours Scale [25–27]. This survey assessed elements of patient experience including how they were greeted, communicated to, and overall experience. We included additional questions to capture for example, patient reports of witnessing rude behaviour, receiving appropriate medications and availability of lab results. Prior to use in this study, we performed cognitive interviews among twenty participants to assess consistency in understanding questions in English, Bemba and Nyanja. Surveys were forward and back translated to ensure consistency across the three languages. The survey tools for trained and untrained clients were identical. Research assistants were trained by the first author in recruitment, training and administering of the TEC and UEC survey in all 16 facilities. The provincial and district health management teams were informed about the unannounced TEC survey as well as the UEC survey. The study team sensitised all facility staff at the start of the study, but HCWs were not aware of who specific TECs were.

Procedures for Trained and Untrained Exit Clients

Efforts to "standardise" assessment of the quality and nature of care in HIV care differs from most previously standardised patient or mystery client work in that HIV care is longitudinal as opposed to episodic or acute care. Under these circumstances, the more conventional standardised patient where a single trained actor can present to multiple different care facilities as a simulated patient with a defined set of symptoms or complaints to assess a single episode of care is not feasible. For example, a patient would have to either register as a new patient or have a false "file" introduced into the paper and electronic medical records — which was deemed infeasible and undesirable.

Instead of simulated patients, we recruited existing patients already receiving care at a particular facility and then subsequently trained them on the concepts of quality of care according to the MOH manual on Quality Improvement for HCWs in Zambia. To avoid disclosing their trained status,

For peer review only - http://bmjopen.bmj.com/site/about/guidelines.xhtml

patients were recruited on the day of their visit prior to them entering the triage area (i.e., the first point of contact with HCWs). Those who consented underwent a single one-on-one training session for 40 to 60 minutes where they were sensitised to the study instrument (which was the same for both TEC and untrained exit clients (UEC)), the MOH care standards, and strategies on being natural yet observant during their clinic visit for that day according to the standard SP approach. These procedures were meant to ensure patients had a clear and uniform understanding on what they should expect during a high-quality patient visit and were attentive to these critical aspects relative to these standards. Immediately after this training, the TEC presented themselves to their facility and completed their visit as they normally would. After their clinic encounter, participants then completed the exit survey in a private area.

For the untrained exit surveys, we took a systematic (every k^{th}, varied by facility size) sample among the patients leaving the facility after attending the clinic on the survey day. Patients were approached by study staff after the visit using a recruitment script to determine their eligibility and were administered the survey after granting consent in a private area.

For both trained and untrained clients, all interviews and surveys were conducted in either English, Bemba or Nyanja depending on the participant's preference. Given the extra time commitments required for the training, TEC participants were given K100 (~$5) for the time spent during training as well as a light snack during the survey administration.

Statistical Analysis

To assess the association between training and response for each question, we conducted unadjusted and adjusted Poisson regression for each question separately [28]. We then assessed the overall association between training and total sum score. We used descriptive statistics to

For peer review only - http://bmjopen.bmj.com/site/about/guidelines.xhtml

characterise patient characteristics and report survey responses. In these analyses, most of the survey responses were reverse coded to identify when respondents reported a negative experience. Results for individual questions (binary response) represent prevalence ratios for reporting a lapse in care. To assess the sum score (count data) we used Poisson regression, estimating the rate ratio for reporting lapses in care. All models were adjusted, given potential differences in survey participants related to different recruitment strategies using mixed-effects regression, adjusted for age, sex, education, care status at the time (i.e., continuously retained in care versus returning to care after disengagement/ lost to follow up [LTFU]), secular time (using cubic splines), allowing random effects at the facility level. We present these results for the overall population as well as stratified by different pre-defined patient subgroups. Lastly, we used bubble plots to compare summary assessments of the patient experience at the facility-level using TECs versus UECs. All analyses were performed using STATA 14MP (StataCorp, College Station, TX, USA). This sub-study represents a secondary analysis and no formal power calculations were performed for this outcome.

Statement of Ethics Approval

Ethics approval to conduct this research was granted by the Zambian Ministry of Health, National Health Research Authority, and the institutional review boards of the University of Zambia (008-03-19), the University of Alabama at Birmingham (300003282) and the London School of Hygiene and Tropical Medicine (21384).

Patient and Public Involvement

Survey questions were developed through a cognitive process with recipients of care. Study implementation guidance was conducted as part of routine CIDRZ partnership with the Zambian

For peer review only

For peer review only - http://bmjopen.bmj.com/site/about/guidelines.xhtml

MOH through a Human Centered Design workshop. CIDRZ engages with implementing partners and affected communities in health facilities, including people living with HIV often represented by neighbourhood health representatives. Although patients were not directly involved in the design of the parent study intervention or the analysis presented here, all study activities were guided by a Scientific Advisory Board with representation from the MOH and a representative of recipients of HIV care. Dissemination of study results is ongoing.

RESULTS

Characteristics of health facilities and patients

We approached 4375 clients (2955 in the untrained and 1420 in the trained), and 3526 participated, of which 2415 (55.2%) completed experience surveys as untrained exit clients (UEC) (56% female, median age was 40 years (interquartile range [IQR]:32-47 years)) and 1111 (32%) completed experience surveys as trained exit clients (TEC) (50% female with a median age 37 years (IQR:31-45 years). Reasons for non-participation included unavailability at the time due to other commitments. Sixteen percent (16%) of UECs and 40% of TECs who had been lost to care and were returning to care on the day of the survey. Education levels differed between UEC and TEC with 47% and 58% reporting completion of secondary level of education, respectively (Table 1). UEC and TEC were similar for HIV enrolment WHO stage with the largest proportion enrolling at WHO stage 1 and similar in terms of marital status.

Table 1. Socio-demographic characteristics of untrained exit and trained exit clients

Characteristics	Level	Untrained Exit Clients n=2415 (68%)	Trained Exit Clients n=1111 (32%)
**Sex, n (%)**			
	**Female**	1355 (56)	553 (50)
	**Male**	1060 (44)	558 (50)

For peer review only - http://bmjopen.bmj.com/site/about/guidelines.xhtml

**Age, Median (IQR)**			
		40 (32-47)	37 (31-45)
**Age category, n (%)**			
	**<30 years**	453 (19)	258 (23)
	**30-40 years**	828 (34)	416 (37)
	**40-50 years**	815 (34)	304 (27)
	**>50 years**	319 (13)	133 (12)
**Education category**			
	**None**	132 (5)	36 (3)
	**Primary**	654 (27)	166 (15)
	**Secondary**	1134 (47)	645 (58)
	**University**	150 (6)	100 (9)
	**Missing**	307 (13)	151 (14)
**HIV Enrollment Stage**			
	**WHO Stage 1**	1173 (49)	533 (48)
	**WHO Stage 2**	314 (13)	147 (13)
	**WHO Stage 3**	355 (15)	162 (15)
	**WHO Stage 4**	27 (1)	7 (1)
	**Missing**	546 (23)	262 (24)
**Care status at survey visit**			
	**In care**	2038 (84)	664 (60)
	**Returning to care**	377 (16)	447 (40)
**Marital Status**			
	**Single**	257 (11)	167 (15)
	**Married**	1361 (56)	575 (52)
	**Divorced**	248 (10)	108 (10)
	**Widowed**	173 (7)	81 (7)
	**Unknown**	41 (2)	20 (2)
	**Missing**	335 (14)	160 (14)
**Facility size**			
	**< 1000 patients**	591 (25)	245 (22)
	**1000-5000 patients**	897 (37)	485 (44)
	**> 5000 patients**	927 (38)	381 (34)

Table 2 shows the absolute responses for TEC and UEC. Although most patients reported a good experience, across the questions between 5% and 25% of patients reported poor experiences in care. For example, when asked if their HIV care provider gave them as much information about their health as they wanted, 13.4% (UEC) vs 24.6% (TEC) of patients reported not being provided with sufficient information about their health. Similarly, between 9.6% vs 18.8% patients reported

For peer review only

For peer review only - http://bmjopen.bmj.com/site/about/guidelines.xhtml

that their HIV care provider was not spending the right amount of time with them at their visit, and 6.8% vs 16.4% reported witnessing rude behaviour.

Table 2. Survey responses by training status

Factor	Level	Untrained Exit Client n (%)	Trained Exit Client n (%)
**Did your HIV care provider greet you in a way that made you feel comfortable?**	Yes	2249 (93.1)	980 (88.2)
	No	166 (6.9)	131 (11.8)
**Did your HIV care provider listen to what you said?**	Yes	2328 (96.4)	1039 (93.5)
	No	79 (3.3)	64 (5.8)
	Refused	8 (0.3)	8 (0.7)
**Did your HIV care provider give you as much information about your health as you wanted?**	Yes	2092 (86.6)	838 (75.4)
	No	323 (13.4)	273 (24.6)
**Did your HIV care provider allow you to ask questions?**	Yes	2082 (86.2)	887 (79.8)
	No	326 (13.5)	222 (20)
	Refused	7 (0.3)	2 (0.2)
**Did your HIV care provider spend the right amount of time with you?**	Yes	2179 (90.2)	900 (81)
	No	232 (9.6)	209 (18.8)
	Refused	4 (0.2)	2 (0.2)
**Overall, how did you feel about the care you received today?**	Happy	2231 (92.4)	983 (88.5)
	Unhappy	178 (7.4)	123 (11.1)
	Refused	6 (0.2)	5 (0.4)
**Overall, were you satisfied with all your HIV care providers today?**	Yes	2206 (91.4)	906 (81.5)
	No	208 (8.6)	202 (18.2)
	Refused	1 (0.0)	3 (0.3)
**I witnessed HIV care providers behaving rudely during my visit today**	No	2251 (93.2)	928 (83.5)
	Yes	163 (6.8)	182 (16.4)
	Refused	1 (0.0)	1 (0.1)
**Were your lab results lost?**	No	2143 (88.7)	985 (88.7)
	Yes	268 (11.1)	126 (11.3)
	Not picking up	4 (0.2)	0 (0)
**Were you able to pick up your medicine today?**	Yes	2366 (98.0)	1087 (97.8)
	No	48 (2.0)	24 (2.2)
	Not Picking Up Meds	1 (0.0)	0 (0)

Effects of training on response patterns: sum score and prevalence ratios

For peer review only - http://bmjopen.bmj.com/site/about/guidelines.xhtml

In adjusted models, TECs overall reported poor experiences in care: 1.64 times as frequently as UEC respondents (Sum Score Rate Ratio [RR]: 1.64 [95% CI: 1.39-1.94] (Fig 1, Supplementary Table S1), and reported an increased prevalence of poor experiences in care quality compared to untrained across almost all questions. For example; among TECs compared to UECs, there was an increased prevalence of reports of not being greeted in a way that made them feel welcome (adjusted Prevalence Ratio [aPR]: 1.71 [95% CI: 1.20-2.44]), reporting being dissatisfied with all their HIV care providers during their HIV care visit (aPR: 2.06 [95% CI: 1.61-2.63]) and witnessing any providers behaving rudely during their visit (aPR: 2.28 [95% CI: 1.63-3.19]) (Fig 1, Supplementary Table S1).

Impact of training across age, sex, and gender to differences in responses

In stratified analysis of the impact of training on the sum score, training was consistently associated with increased identification of poor experiences in care across all subgroups apart from those aged 50 years or older and those with no education. We also observed that training had a larger impact among females compared to males, those with a primary education only, and among individuals presenting at smaller facilities (Fig 2). We observed similarities in responses on the impact of training on different age categories, sex, care status and different levels of education when we looked at individual questions except for the question on providers spending the right amount of time where we found that females were twice as likely to report lapses with care compared to males (Supplementary Figure 1). Using TECs gave worse assessments of patient experience at the facility-level regardless of facility size compared to UECs (Fig 3, Supplementary Figure 2).

DISCUSSION

For peer review only - http://bmjopen.bmj.com/site/about/guidelines.xhtml
For peer review only

Disengaged patients often express a disconnect between their care expectations and the provider's style, hence experience is bound to vary across facilities [8]. This disconnect can lead to dissatisfaction with HIV services which can often lead to patients dropping out of care[8,11,29]. A brief training for patients living with HIV on how to evaluate the quality and experience of routine care changed patient experience reports compared to untrained patients using the same instrument. Patients who underwent a brief training identified more lapses in care across most questions. Women and young people were more likely to report critical responses after training - consistent with the idea that those who feel least empowered underwent the biggest change. Differences were also bigger for questions in which social desirability is likely to operate. For example, larger differences were observed for witnessing rude behaviour, while no differences were observed for more objective questions such as whether lab results were lost.

Improving HIV health outcomes requires new strategies that minimise methodological biases and includes everyone the patient encounters during their visit, including clinical officers, doctors, nurses, data clerks, and lay HCWs. Our TEC approach could contribute to getting a true reflection of how much value patients place on things such as effective communication, being greeted appropriately, or being treated with care and respect at all these different touch points. Involving patients in their own care and design of health services has been linked to improved HIV care retention and patient outcomes, such as higher viral suppression rates [30–32]. As progress is being made towards UNAIDS 95-95-95 targets, the global HIV sector is constantly reviewing priorities and challenges for optimal engagement in care [33,34]. Patient experience is a key indicator of healthcare quality for meeting the 95-95-95 targets: delivering services patients need, can access, and address wider determinants of poor health. Clinicians and health systems must address HIV patients' needs from diagnosis to death to ensure healthy ageing and viral suppression. Other

For peer review only - http://bmjopen.bmj.com/site/about/guidelines.xhtml

outcomes in Zambia [11,35,36] show that lifelong needs vary by facility, highlighting the importance of metrics that measure patient experience accurately. We have shown that it is feasible to involve patients in assessing the quality of care and this could potentially lead to involvement of patients in the redesign of healthcare services.

Because HIV care is longitudinal, SP, who are often used to evaluate episodic care, require highly skilled people to pose as a simulated patient making one visit to multiple clinics, posing practical implementation challenges in our setting[19–24]. Contrary to SP, we evaluated care quality without using simulated patients and administered the survey once among people in long term care. Using real patients instead of simulated ones drawn from outside the true patient population, we would argue, made our TEC approach more applicable and reproducible in clinical settings. We were able to record HCW behaviour in a typical HIV context using this concealment method, potentially reducing the impact of the Hawthorne effect. Our TECs also consistently identified more lapses in care, potentially reducing social desirability bias and ability to identify issues at the facility. Even though training takes time, the increased quality of our measurement allows one to perform fewer surveys. With traditional approaches like exit surveys, one would require a larger sample size, but this does not address bias [37].

Our findings are consistent with a study done in South Africa which found that non-clinical dimensions of care play a bigger role in determining an overall satisfactory experience for standardised patients when compared to untrained patients[37]. However, our findings may contradict previous suggestions that tailoring support to individuals to build skills and confidence through patient activation can lead to trained/informed patients reporting a better experience than untrained/ uninformed [38]. TECs cared about the following non-clinical aspects of care: rude providers, being satisfied with HIV care providers, and spending enough time with providers. This

For peer review only - http://bmjopen.bmj.com/site/about/guidelines.xhtml

finding is consistent with a previous study in Zambia, where patients reported rude HCWs deterring HIV care engagement [8,10,11]. This could mean that studies assessing patient experience with TEC could focus on a few questions to save time and resources. Questions like, "Did you pick up your medicine or lab results at your visit?" may not add much to a TEC survey because they are definitive, and training appears to influence subjective care dimensions.

Women TECs were generally more critical about the care they received and would likely provide a more accurate reflection of the health system, possibly because they have better health-seeking behaviour than men, which may be strongly influenced by local gender norms and health service structures designed to engage women of reproductive age [39]. There is some consistency with other findings that women may be more interested in their care than men, especially in facilities that provide integrated services for women and their children [9,40]. Despite longer wait times, women were more satisfied with integrated facilities [41]. In addition, middle-aged people between 40-50 benefited the most from training. Compared to older people over 50, younger people under 30 were less satisfied with the care they received and often felt they were not greeted by a HCW during their visit. This finding is consistent with cultural norms where younger people are less respected[42]. Given the current strategy of targeting young people, who account for most new infections, these findings suggest an important new approach to identifying what young people value most. Education level was among the strongest predictors of patient experience feedback. Well-educated patients were found to have a less critical/better HIV care visit experience compared to participants with lower levels of educational attainment. This difference in care experience report may be associated, at least in part, with the HCW perception of the patient in the facility. Research conducted in Nigeria discovered that people with higher levels of education are frequently given

For peer review only - http://bmjopen.bmj.com/site/about/guidelines.xhtml

better and more considerate treatment by HCW, hence limited by a form of discrimination/ socioeconomic status bias [43,44].

The observed effect of training on patient experience is likely multifaceted potentially stemming from increased attention and recall to the exit survey items which solicited a feeling of empowerment to be more critical of the care received. In future studies, patient activation should be measured as an outcome to see how training changes the patient's engagement with their care over time [38]. Further research is required into why women TECs reported poorer experiences with care than men. Other studies that have used SP to assess medical students' performance showed that women were more critical on certain aspects of care. These studies also recommend matching of SPs to clinicians by sex [45], something we were not able to do given the nature of our study in primary health facilities where we assessed interpersonal communication with HCWs at all levels. Perhaps our findings call for more investigation into the integration of women's services, such as family planning and children's services with HIV care given some studies have shown this can improve patient satisfaction.

Limitations

Our findings should be interpreted with caution due to the following limitations. Because this was the first time such a study was done, we recruited educated participants who were able to read and write, perceived to have good recall ability and were able to comprehend things. Our study was only done in Lusaka province in facilities that were largely urban except for one facility which was peri-urban hence it is hard to generalise these findings. Another limitation in our approach is the one-time cross-sectional nature of our measurements among people in long term HIV care. If more measures were collected from each TEC, we may well see them being activated in a way that results in an improvement in their experience based on the skills they develop to seek better care from

For peer review only

For peer review only - http://bmjopen.bmj.com/site/about/guidelines.xhtml

providers which ultimately would improve their retention in care. Despite its limitations, the TEC method provides valuable information about healthcare quality, even though it is limited to situations where "walk-ins" are permitted. Our approach only focused on real patients accessing care and we did not manipulate any patient files, so it is possible that some TECs were known to the facility as patients accessing chronic care. Our approach does require a trained interviewer to speak with TECs after their visits, but this is not any different to what already exists. In future, it may be worth using the domains in the national HIV guidelines as the gold standard, but we did not do this as our aim was to come up with a low-cost approach that can easily be rolled out. In addition, the concept of patient centred care is still catching on in Zambia. Our TEC approach can be used to further the knowledge in provider attitudes to other relatively new approaches to delivering quality HIV care such as differentiated service delivery (DSD) for stable patients by assessing whether HCWs follow guidelines when offering this [34]. We also see an opportunity to assess provider patient communication of viral load laboratory results by use of a universal script for each TEC to assess if they are communicated to and if unsuppressed but adherent, what procedures followed.

Conclusion

TEC offers pragmatic methods for health systems in low-income countries to assess non-clinical dimensions of care (communication, respect, and autonomy) which are grounded on the concept of health-system responsiveness and could be critical to the transformation of low-quality health systems to high quality ones[46]. Hawthorne effects and social desirability biases may be mitigated using TECs. We were able to capture HCWs behaviour in a normal day to day low middle income setting using similar approaches recommended by King and colleagues that minimise harm to HCWs and SPs [15]. Our findings suggest that TECs provide a more critical appraisal of some

For peer review only

aspects of the quality of HIV care. It provides new insights in the Zambian context on what patients' value when they interact with the health system. This could be important given the need to reduce loss to follow up among new ART clients who disengage within the first 6 months of treatment due to a bad first encounter with the health system. Our TEC approach could be used to assess reengagement interventions. The fact that TECs had a better understanding of the items solicited or felt empowered to be more critical shows that the training we provided worked. This low-cost method could be reproduced in other routine settings and presents an opportunity to further institutionalise patient centred care by evaluating what happens at the point of contact between the patient, the health facility, and the health provider. The implications are that it provides an opportunity to improve HIV care, meet patients' expectations and can serve as a monitoring tool for healthcare performance. Coupled with the recent approaches to client led monitoring in HIV care, our approach can be used to enhance decision making that considers patients' involvement.

For peer review only

For peer review only - http://bmjopen.bmj.com/site/about/guidelines.xhtml

Data Availability Statement

The Government of Zambia allows data sharing when applicable local conditions are satisfied. In this case, the data from the study will be made available to any interested researchers upon request. The CIDRZ Ethics and Compliance Committee is responsible for approving such requests. To request data access, one must write to the Secretary to the Committee/Head of Research Operations, Mrs. Hope Chinganya (Hope.Chinganya@cidrz.org) mentioning the intended use for the data. The Committee will then facilitate review and authorization to release the data as requested. Data requests must include contact information, a research project title, and a description of the intended use.

Funding

This work was supported, in whole or in part, by the Bill & Melinda Gates Foundation [INV-010563]. Under the grant conditions of the Foundation, a Creative Commons Attribution 4.0 Generic License has already been assigned to the Author Accepted Manuscript version that might arise from this submission. This work was also supported by the National Center for Advancing Translational Sciences (grant KL2 TR002346 to Dr Mody) and the National Institute of Allergy and Infectious Diseases (grant K24 AI134413 to Dr Geng). The funders had no role in study design, data collection and analysis, decision to publish, or preparation of the manuscript.

Disclaimer

The contents of this paper are the sole responsibility of the authors and do not necessarily reflect the views of the Bill & Melinda Gates Foundation.

Competing interest: Non declared

For peer review only

For peer review only - http://bmjopen.bmj.com/site/about/guidelines.xhtml

Acknowledgements

We would like to thank recipients of care for taking part in this study. We would like to thank the Zambian Ministry of Health, the US President's Emergency Plan for AIDS Relief (PEPFAR) through the US Centers for Disease Control and Prevention/Zambia (CDC) and the Centre for Infectious Disease Research in Zambia for their leadership in ensuring those in HIV care continue to receive life-saving HIV treatment. We would also like to thank the healthcare workers who continued to faithfully deliver HIV care in the face of the COVID-19 pandemic

Contributors

KS: guarantor, lead author, conducted all analyses, led data management activities, field coordination of activities and designed data collection tools. JMP: field coordination of data, assisted with analysis and revising it critically for important intellectual content, designed data collection tools. AM: assisted with analysis, framing, and revising it critically for important intellectual content. BR: final approval for publication, assisted with framing and revising it critically for important intellectual content CB: field coordination of data quality processes and data acquisition IEW: drafted statistical analysis plan, assisted with conceptualisation and interpretation of data. JM: assisted with data acquisition and cleaning, field coordination of data quality processes. SS: led intervention implementation, project administration and data curation. LKB, NM, AS: cognitively tested data collection tools, assisted with conceptualization, underlying data processes, and assisted with writing and data interpretation. DM, MM-M: advised implementation details. AW, JH: assisted with framing and revising it critically for important intellectual content. CBM: lead for underlying data processes and assisted with funding acquisition. CBH: funding acquisition, assisted with conceptualisation and advised regarding intervention implementation details. IS: funding

For peer review only

acquisition, assisted with conceptualisation and manuscript writing. EHG: funding acquisition, led conceptualisation and advised on all analyses, final approval for publication.

Statement of Ethics Approval

Ethics approval to conduct this research was granted by the Zambian Ministry of Health, National Health Research Authority, and the institutional review boards of the University of Zambia (008-03-19), the University of Alabama at Birmingham (300003282) and the London School of Hygiene and Tropical Medicine (21384).

Figure 1. Forest plot comparing responses from Trained Exit Clients (TEC) relative to Untrained Exit Clients (UEC) on 10 measures of clinic experience. Points indicate the rate ratio (for sum score) or prevalence ratio (for all others) for identifying a lapse in care in TEC surveys as compared to UEC. The sum score represents the total number of binary responses (yes vs no) across all clients in one group shown as a rate ratio. The red line indicates a rate or prevalence ratio of 1 and values greater than this indicates more lapses in care identified in TECs. Results are based on mixed-effects models adjusted for age, sex, education with a random effect at the facility.

Figure 2. Impact of Training on Identifying Care Lapses Stratified by Subgroups (N=3480). When all questions were collapsed into a Sum score among TEC, females were more likely to report lapses in care quality than males. We observed some level of interaction for care status, age category, education category and facility size.

Figure 3. Bubble plot showing Trained Exit Sum Score vs Untrained Exit Sum Score. Each bubble represents a single facility's performance. Each bubble’s size indicates the number of patients at each facility with larger bubbles corresponding to larger facilities. The horizontal position notes the Untrained Exit Sum Score for all questions against the facility, and the vertical position notes the Trained Exit sum score at the same facility.

For peer review only

REFERENCES

1 UNAIDS JUNP on H-G. *In Danger: UNAIDS Global AIDS Update 2022*. 2022. doi:10.18356/9789210019798

2 Institute of Medicine & Committee on Quality of Health Care in America. *Crossing the Quality Chasm: A New Health System for the 21st Century*. 2001. doi:10.1136/bmj.323.7322.1192

3 World Health Organization. Maintaining and Improving Quality of Care Within HIV Clinical Services. 2019. http://apps.who.int/bookorders. (accessed 11 Mar 2020).

4 Edmonds KA, Aspiras OG, Rose JP, *et al.* Cross-sectional evaluation of perceived health care provider engagement, self-efficacy, and ART adherence in people living with HIV/AIDS. *AIDS Care* 2021;**33**:154–8. doi:10.1080/09540121.2019.1703889

5 Bakken S, Holzemer WL, Brown MA, *et al.* Relationships between perception of engagement with health care provider and demographic characteristics, health status, and adherence to therapeutic regimen in persons with HIV/AIDS. *AIDS Patient Care STDS* 2000;**14**:189–97. doi:10.1089/108729100317795

6 Okoli C, Brough G, Allan B, *et al.* Shared Decision Making Between Patients and Healthcare Providers and its Association with Favorable Health Outcomes Among People Living with HIV. *AIDS Behav* 2021;**25**:1384–95. doi:10.1007/s10461-020-02973-4

7 Kwan A, Daniels B, Bergkvist S, *et al.* Use of standardised patients for healthcare quality research in low- and middle-income countries. *BMJ Glob Health* 2019;**4**:e001669. doi:10.1136/bmjgh-2019-001669

8 Mwamba C, Sharma A, Mukamba N, *et al.* 'They care rudely!': resourcing and relational health system factors that influence retention in care for people living with HIV in Zambia. *BMJ Glob Health* 2018;**3**:e001007. doi:10.1136/bmjgh-2018-001007

9 Topp SM, Mwamba C, Sharma A, *et al.* Rethinking retention: Mapping interactions between multiple factors that influence long-term engagement in HIV care. *PLoS One* 2018;**13**:e0193641.https://doi.org/10.1371/journal.pone.0193641

10 Zanolini A, Sikombe K, Sikazwe I, *et al.* Understanding preferences for HIV care and treatment in Zambia: Evidence from a discrete choice experiment among patients who have been lost to follow-up. *PLoS Med* 2018;**15**:e1002636. doi:10.1371/JOURNAL.PMED.1002636

11 Sikazwe I, Eshun-Wilson I, Sikombe K, *et al.* Patient-reported Reasons for Stopping Care or Switching Clinics in Zambia: A Multi-site, Regionally Representative estimate using a multi-stage sampling-based approach in Zambia. *Clinical Infectious Diseases* Published Online First: 2020. doi:10.1093/cid/ciaa1501

12 Mody A, Sikombe K, Beres LK, *et al.* Profiles of HIV Care Disruptions Among Adult Patients Lost to Follow-up in Zambia. *JAIDS Journal of Acquired Immune Deficiency Syndromes* 2020;**Publish Ah**. doi:10.1097/qai.0000000000002530

13 Porter LE, Bouey PD, Curtis S, *et al.* Beyond indicators: Advances in global HIV monitoring and evaluation during the PEPFAR era. *J Acquir Immune Defic Syndr (1988)* 2012;**60**. doi:10.1097/QAI.0b013e31825cf345

14 MILLER FRANCO L, FRANCO C, KUMWENDA N, *et al.* Methods for assessing quality of provider performance in developing countries. *International Journal for Quality in Health Care* 2002;**14**:17–24. doi:10.1093/intqhc/14.suppl_1.17

15 King JJC, Das J, Kwan A, *et al.* How to do (or not to do) … using the standardized patient method to measure clinical quality of care in LMIC health facilities. *Health Policy Plan* 2019;**34**:625–34. doi:10.1093/heapol/czz078

For peer review only - http://bmjopen.bmj.com/site/about/guidelines.xhtml

16 Onishi J, Gupta S, Peters DH. Comparative analysis of exit interviews and direct clinical observations in Pediatric Ambulatory Care Services in Afghanistan. *International Journal for Quality in Health Care* 2011;**23**:76–82. doi:10.1093/intqhc/mzq074
17 Luck J, Peabody JW. Using standardised patients to measure physicians' practice: validation study using audio recordings. *BMJ* 2002;**325**:679. doi:10.1136/bmj.325.7366.679
18 Beullens J, Rethans JJ, Goedhuys J, *et al.* The use of standardized patients in research in general practice. *Fam Pract* 1997;**14**:58–62. doi:10.1093/fampra/14.1.58
19 Weiner SJ, Schwartz A. Directly observed care: Can unannounced standardized patients address a gap in performance measurement? *J Gen Intern Med* 2014;**29**:1183–7. doi:10.1007/s11606-014-2860-7
20 Nestel D, Bearman M. *Simulated Patient Methodology: Theory, Evidence and Practice*. 2014. doi:10.1002/9781118760673
21 Larsen A, Wilson KS, Kinuthia J, *et al.* Standardised patient encounters to improve quality of counselling for pre-exposure prophylaxis (PrEP) in adolescent girls and young women (AGYW) in Kenya: study protocol of a cluster randomised controlled trial. *BMJ Open* 2020;**10**:e035689–e035689. doi:10.1136/bmjopen-2019-035689
22 Daniels B, Dolinger A, Bedoya G, *et al.* Use of standardised patients to assess quality of healthcare in Nairobi, Kenya: A pilot, cross-sectional study with international comparisons. *BMJ Glob Health* Published Online First: 2017. doi:10.1136/bmjgh-2017-000333
23 Zabar S, Hanley K, Stevens D, *et al.* Unannounced standardized patients: A promising method of assessing patient-centered care in your health care system. *BMC Health Serv Res* 2014;**14**. doi:10.1186/1472-6963-14-157
24 Boffa J, Moyo S, Chikovore J, *et al.* Quality of care for tuberculosis and HIV in the private health sector: a cross-sectional, standardised patient study in South Africa. *BMJ Glob Health* 2021;**6**:e005250. doi:10.1136/bmjgh-2021-005250
25 Wachira J, Middlestadt S, Reece M, *et al.* Psychometric assessment of a physician-patient communication behaviors scale: The perspective of adult HIV patients in Kenya. *AIDS Res Treat* 2013;**2013**. doi:10.1155/2013/706191
26 Campbell C, Lockyer J, Laidlaw T, *et al.* Assessment of a matched-pair instrument to examine doctor−patient communication skills in practising doctors. *Med Educ* 2007;**41**:123–9. doi:https://doi.org/10.1111/j.1365-2929.2006.02657.x
27 Bieber C, Müller KG, Nicolai J, *et al.* How Does Your Doctor Talk with You? Preliminary Validation of a Brief Patient Self-Report Questionnaire on the Quality of Physician–Patient Interaction. *J Clin Psychol Med Settings* 2010;**17**:125–36. doi:10.1007/s10880-010-9189-0
28 Zou G. A Modified Poisson Regression Approach to Prospective Studies with Binary Data. *Am J Epidemiol* 2004;**159**:702–6. doi:10.1093/aje/kwh090
29 Shabalala FS, Vernooij E, Pell C, *et al.* Understanding reasons for discontinued antiretroviral treatment among clients in test and treat: a qualitative study in Swaziland. *J Int AIDS Soc* 2018;**21**:e25120. doi:10.1002/jia2.25120
30 Grimsrud A, Bygrave H, Doherty M, *et al.* Reimagining HIV service delivery : the role of differentiated care from prevention to suppression. *J Acquir Immune Defic Syndr* 2016;:10–2. doi:10.7448/IAS.19.1.21484
31 Camlin CS, Neilands TB, Odeny TA, *et al.* Patient-reported factors associated with reengagement among HIV-infected patients disengaged from care in East Africa. *AIDS* 2015;:1. doi:10.1097/QAD.0000000000000931

For peer review only - http://bmjopen.bmj.com/site/about/guidelines.xhtml

32 Geng EH, Bangsberg DR, Musinguzi N, *et al.* Understanding Reasons for and Outcomes of Patients Lost to Follow-Up in Antiretroviral Therapy Programs in Africa Through a Sampling-Based Approach. *JAIDS Journal of Acquired Immune Deficiency Syndromes* 2010;**53**:405–11. doi:10.1097/QAI.0b013e3181b843f0

33 Ford N, Geng E, Ellman T, *et al.* Emerging priorities for HIV service delivery. *PLoS Med* 2020;**17**:e1003028. doi:10.1371/JOURNAL.PMED.1003028

34 Ehrenkranz P, Grimsrud A, Holmes CB, *et al.* Expanding the Vision for Differentiated Service Delivery: A Call for More Inclusive and Truly Patient-Centered Care for People Living With HIV. *J Acquir Immune Defic Syndr* 2021;**86**:147. doi:10.1097/QAI.0000000000002549

35 Holmes CB, Sikazwe I, Sikombe K, *et al.* Estimated mortality on HIV treatment among active patients and patients lost to follow-up in 4 provinces of Zambia: Findings from a multistage sampling-based survey. *PLoS Med* 2018;**15**:e1002489. doi:10.1371/journal.pmed.1002489

36 Sikazwe I, Eshun-Wilson I, Sikombe K, *et al.* Retention and viral suppression in a cohort of HIV patients on antiretroviral therapy in Zambia: Regionally representative estimates using a multistage-sampling-based approach. *PLoS Med* 2019;**16**:1–17. doi:10.1371/journal.pmed.1002811

37 Hompashe DM, Gerdtham U-G, Christian CS, *et al.* 'The nurse did not even greet me': how informed versus non-informed patients evaluate health systems responsiveness in South Africa. *BMJ Glob Health* 2021;**6**:e004360. doi:10.1136/bmjgh-2020-004360

38 Hibbard JH, Greene J. What The Evidence Shows About Patient Activation: Better Health Outcomes And Care Experiences; Fewer Data On Costs. *Health Aff* 2013;**32**:207–14. doi:10.1377/hlthaff.2012.1061

39 Colvin CJ. Strategies for engaging men in HIV services. Lancet HIV. 2019;**6**:e191–200. doi:10.1016/S2352-3018(19)30032-3

40 Kerkhoff AD, Sikombe K, Eshun-Wilson I, *et al.* Mortality estimates by age and sex among persons living with HIV after ART initiation in Zambia using electronic medical records supplemented with tracing a sample of lost patients: A cohort study. *PLoS Med* 2020;**17**:e1003107.https://doi.org/10.1371/journal.pmed.1003107

41 Narasimhan M, Yeh PT, Haberlen S, *et al.* Integration of HIV testing services into family planning services: a systematic review. *Reprod Health* 2019;**16**:61. doi:10.1186/s12978-019-0714-9

42 Godia PM, Olenja JM, Lavussa JA, *et al.* Sexual reproductive health service provision to young people in Kenya; Health service providers' experiences. *BMC Health Serv Res* 2013;**13**:1–13. doi:10.1186/1472-6963-13-476/TABLES/2

43 Roberts BW, Puri NK, Trzeciak CJ, *et al.* Socioeconomic, racial and ethnic differences in patient experience of clinician empathy: Results of a systematic review and meta-analysis. *PLoS One* 2021;**16**:e0247259.https://doi.org/10.1371/journal.pone.0247259

44 Aloh HE, Onwujekwe OE, Aloh OG, *et al.* Impact of socioeconomic status on patient experience on quality of care for ambulatory healthcare services in tertiary hospitals in Southeast Nigeria. *BMC Health Serv Res* 2020;**20**:473. doi:10.1186/s12913-020-05332-0

45 Humphrey-Murto S, Touchie C, Wood TJ, *et al.* Does the gender of the standardised patient influence candidate performance in an objective structured clinical examination? *Med Educ* 2009;**43**:521–5. doi:10.1111/j.1365-2923.2009.03336.x

46 Kruk ME, Gage AD, Arsenault C, *et al.* High-quality health systems in the Sustainable Development Goals era: time for a revolution. Lancet Glob Health. 2018;**6**:e1196–252. doi:10.1016/S2214-109X(18)30386-3

For peer review only - http://bmjopen.bmj.com/site/about/guidelines.xhtml

For peer review only

4

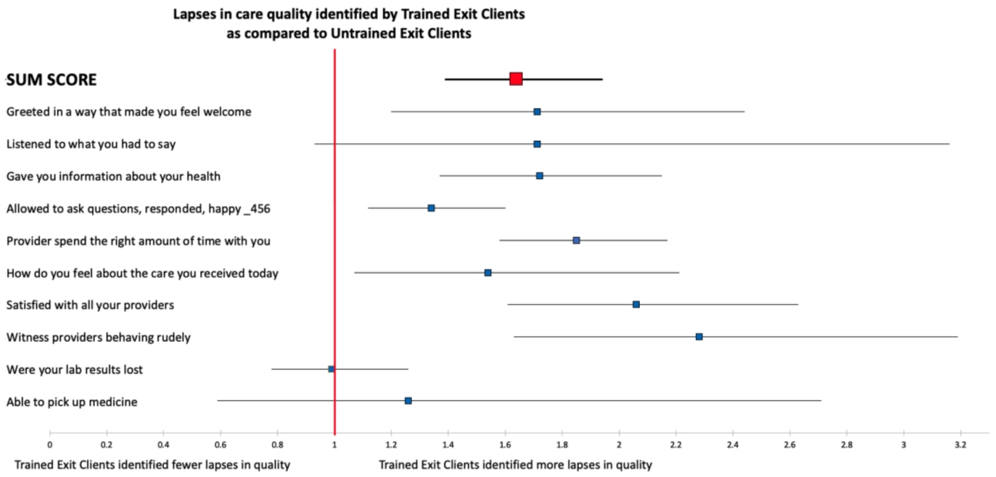

674x330mm (72 x 72 DPI)

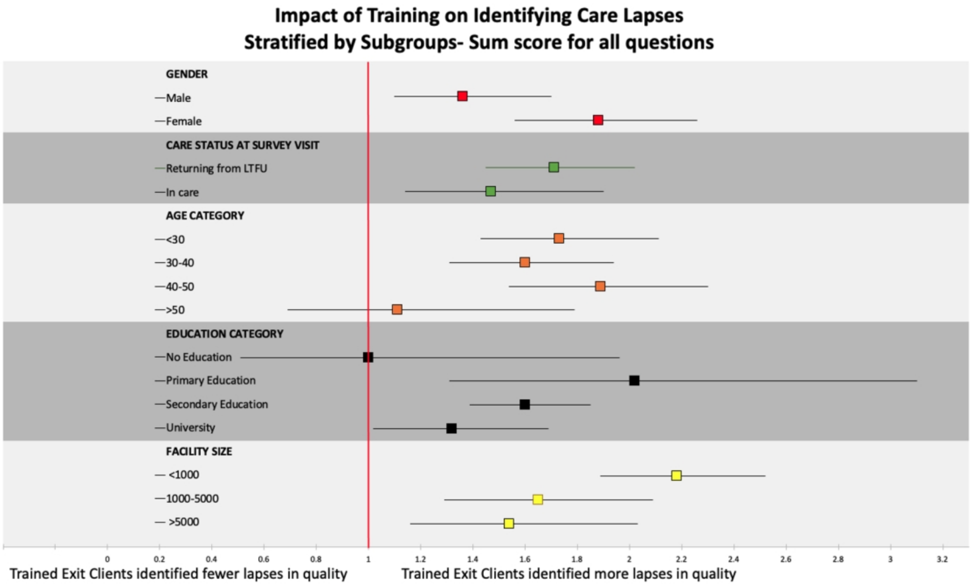

629x374mm (72 x 72 DPI)

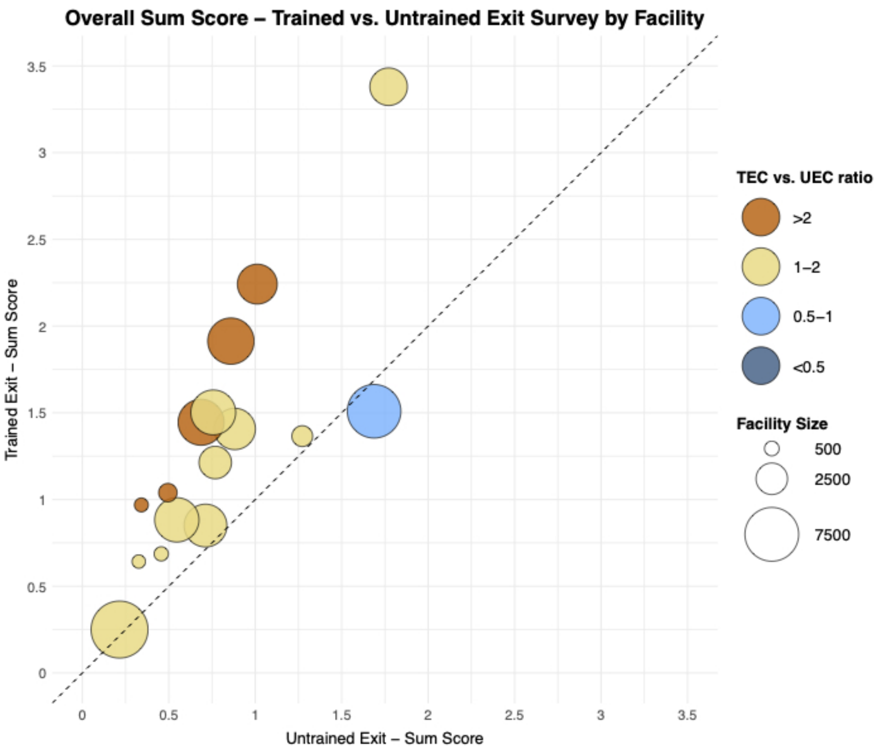

279x215mm (72 x 72 DPI)

Supplementary Tables S1

Supplementary Table S1. Mixed effect Poisson regression comparing 10 questions for Trained Exit Clients vs Untrained Exit Clients. Adjusted for age, sex, education, and study period.

**Trained Exit Clients**	**Prevalence ratio (PR) Unadjusted**	**P value**	**95%** Confidence Interval **(CI)**	**PR-Adjusted**	**P value**	**95%** Confidence Interval **(CI)**	**N**
**Sum score (Rate ratio)**	1.73	<0.01	1.47-2.02	1.64	<0.01	1.39-1.94	3480
**Did your HIV care provider greet you in a way that made you feel comfortable?**	1.74	0.01	1.24-2.44	1.71	<0.01	1.20-2.44	3526
**Did your HIV care provider listen to what you said?**	1.77	0.09	0.91-3.45	1.71	0.09	0.93-3.16	3510
**Did your HIV care provider give you as much information about your health as you wanted?**	1.82	<0.01	1.43-2.33	1.72	<0.01	1.37-2.15	3526
**Did your HIV care provider allow you to ask questions?**	1.44	<0.01	1.20-1.73	1.34	<0.01	1.12-1.6	3517
**Did your HIV care provider spend the right amount of time with you?**	1.94	<0.01	1.66-2.27	1.85	<0.01	1.58-2.17	3520
**Overall, how did you feel about the care you received today?**	1.51	0.02	1.06-2.16	1.54	0.02	1.07-2.21	3515
**Overall, were you satisfied with all your HIV care providers today?**	2.12	<0.01	1.68-2.66	2.06	<0.01	1.61-2.63	3522
**I witnessed HIV care providers behaving rudely during my visit today**	2.39	<0.01	1.73-3.32	2.28	<0.01	1.63-3.19	3524
**Were your lab results lost?**	0.99	0.98	0.84-1.19	0.99	0.93	0.78-1.26	3522
**Were you able to pick up your medicine today?**	1.04	0.90	0.57-1.89	1.26	0.55	0.59-2.71	3525

For peer review only

For peer review only

Supplementary Figure 1.

Impact of Training on Identifying Care Lapses Stratified by Subgroups for 10 questions. We observed some level of interaction for care status, age category, education category and facility size. Panel **a)** Greet you in a way that made you feel comfortable **b)** Listen to what you said **c)** Give you as much information about your health as you wanted **d)** Allowed you to ask questions, responded, happy q456 **e)** spend the right amount of time with you **f)** feel about the care you received today **g)** satisfied with all your HIV care providers today **h)** witnessed HIV care providers behaving rudely during my visit today **i)** lost lab results **j)** pick up meds

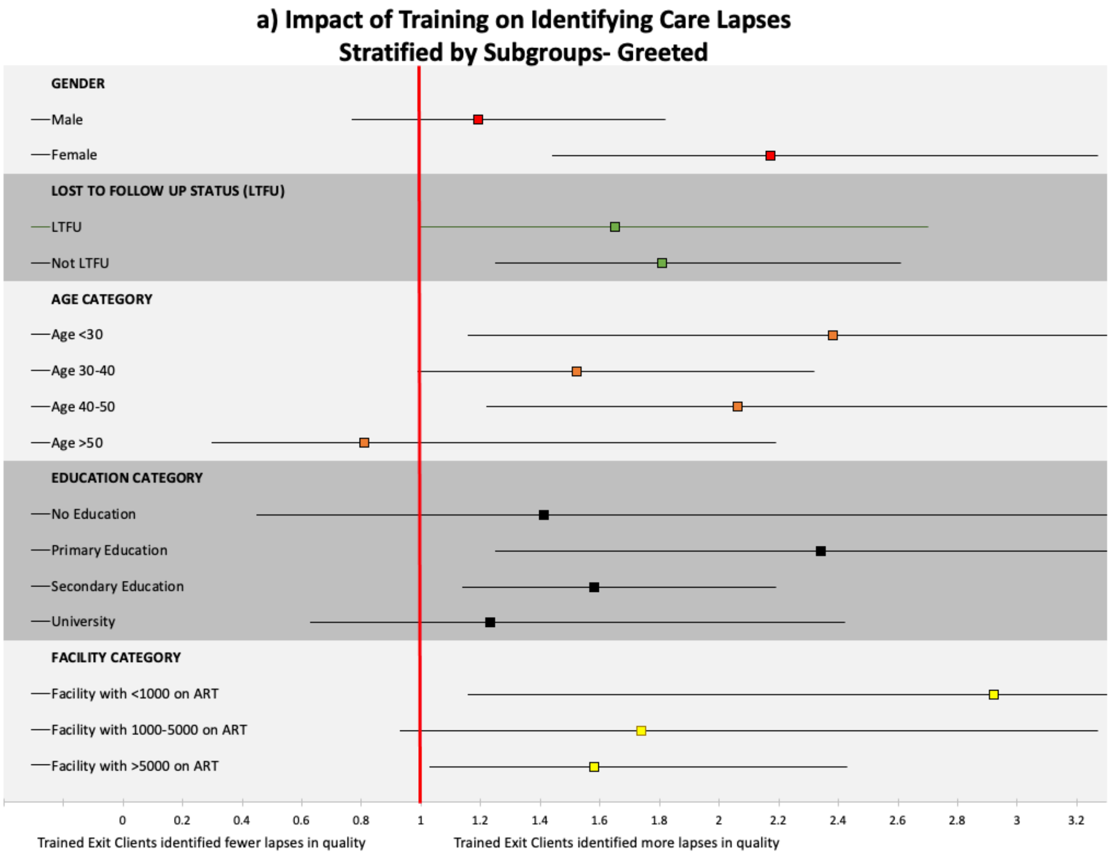

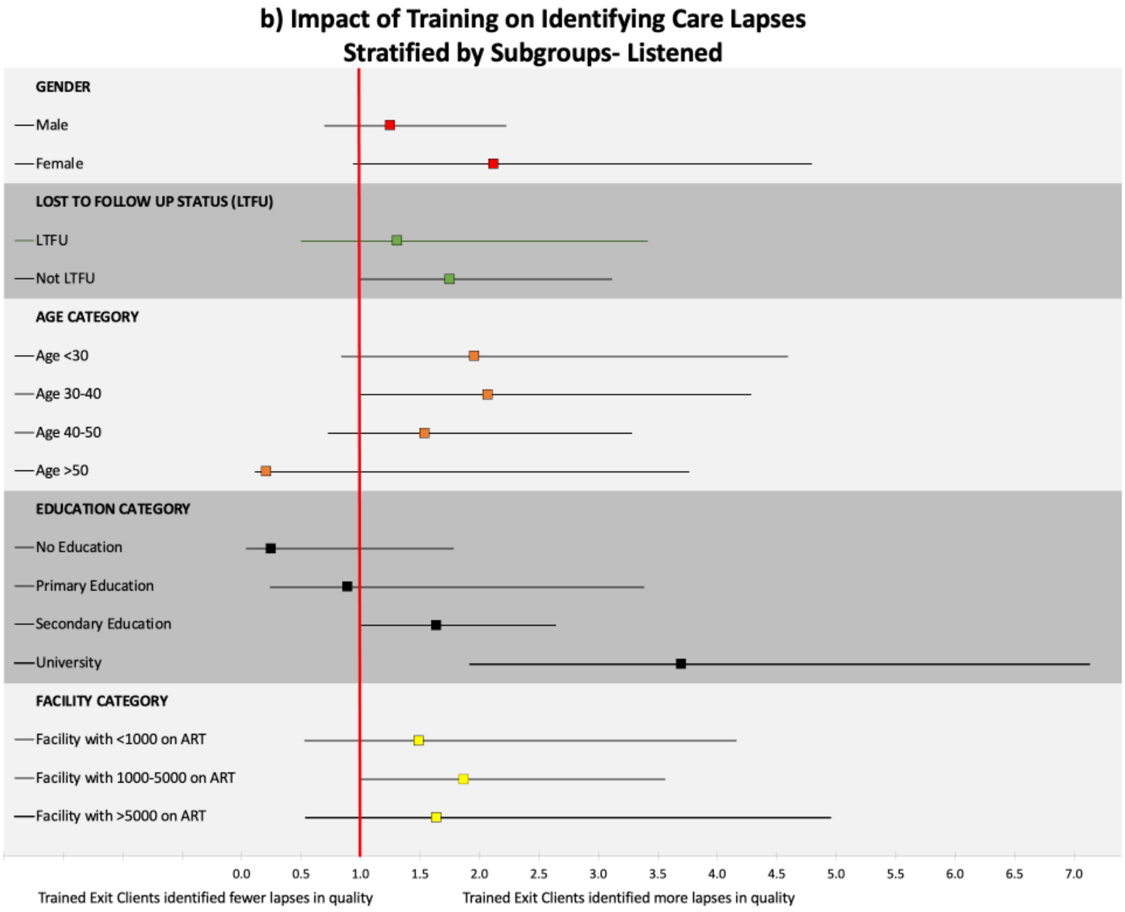
b) Impact of Training on Identifying Care Lapses
Stratified by Subgroups- Listened
GENDER
Male
Female
LOST TO FOLLOW UP STATUS (LTFU)
LTFU
Not LTFU
AGE CATEGORY
Age <30
Age 30-40
Age 40-50
Age >50
EDUCATION CATEGORY
No Education
Primary Education
Secondary Education
University
FACILITY CATEGORY
Facility with <1000 on ART
Facility with 1000-5000 on ART
Facility with >5000 on ART
0.0 0.5 1.0 1.5 2.0 2.5 3.0 3.5 4.0 4.5 5.0 5.5 6.0 6.5 7.0
Trained Exit Clients identified fewer lapses in quality
Trained Exit Clients identified more lapses in quality

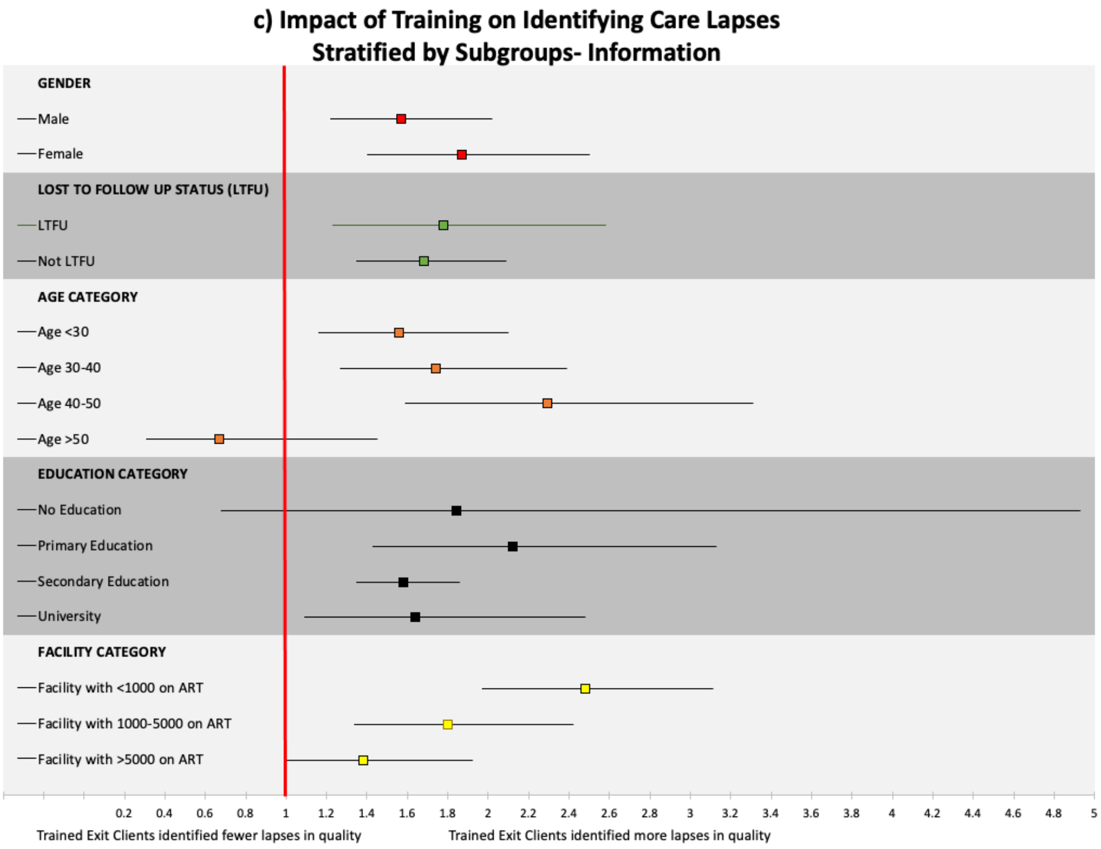
c) Impact of Training on Identifying Care Lapses
Stratified by Subgroups- Information
GENDER
Male
Female
LOST TO FOLLOW UP STATUS (LTFU)
LTFU
Not LTFU
AGE CATEGORY
Age <30
Age 30-40
Age 40-50
Age >50
EDUCATION CATEGORY
No Education
Primary Education
Secondary Education
University
FACILITY CATEGORY
Facility with <1000 on ART
Facility with 1000-5000 on ART
Facility with >5000 on ART
0.2 0.4 0.6 0.8 1 1.2 1.4 1.6 1.8 2 2.2 2.4 2.6 2.8 3 3.2 3.4 3.6 3.8 4 4.2 4.4 4.6 4.8 5
Trained Exit Clients identified fewer lapses in quality
Trained Exit Clients identified more lapses in quality

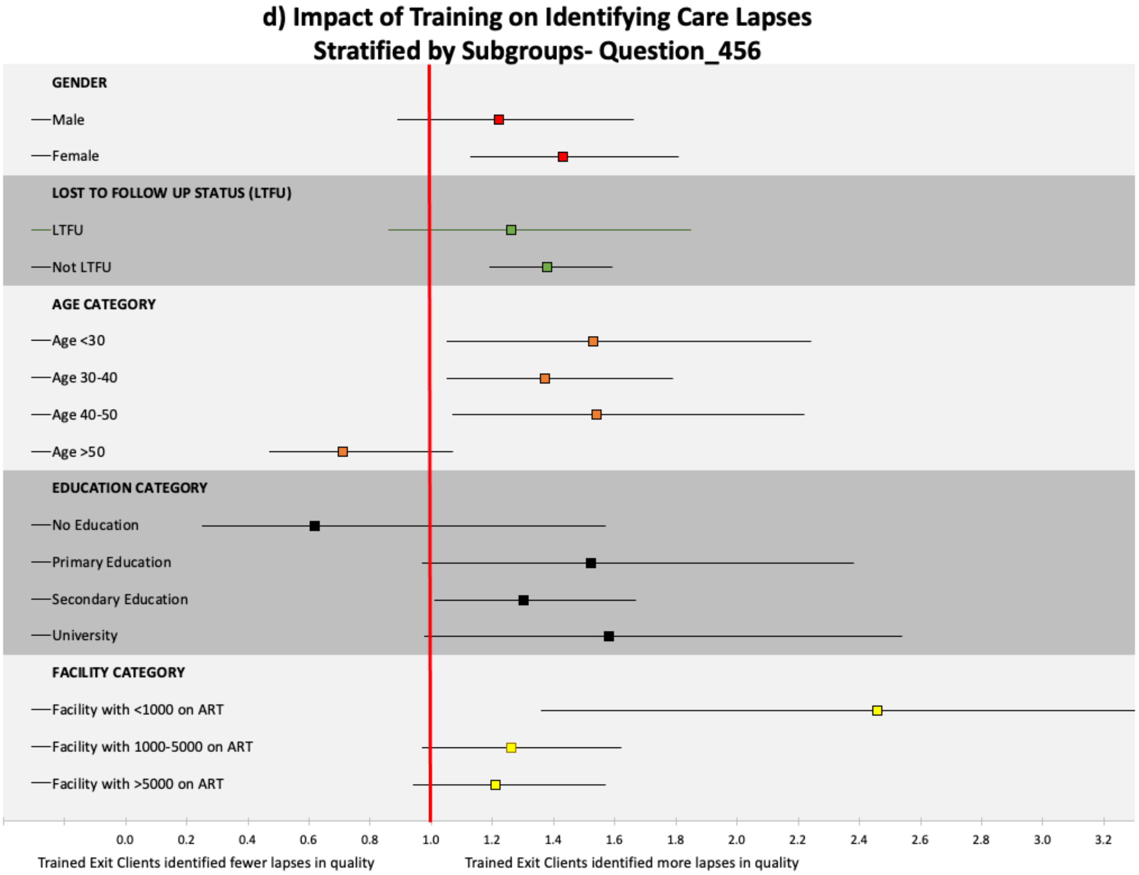
d) Impact of Training on Identifying Care Lapses
Stratified by Subgroups- Question_456
GENDER
Male
Female
LOST TO FOLLOW UP STATUS (LTFU)
LTFU
Not LTFU
AGE CATEGORY
Age <30
Age 30-40
Age 40-50
Age >50
EDUCATION CATEGORY
No Education
Primary Education
Secondary Education
University
FACILITY CATEGORY
Facility with <1000 on ART
Facility with 1000-5000 on ART
Facility with >5000 on ART
0.0 0.2 0.4 0.6 0.8 1.0 1.2 1.4 1.6 1.8 2.0 2.2 2.4 2.6 2.8 3.0 3.2
Trained Exit Clients identified fewer lapses in quality
Trained Exit Clients identified more lapses in quality

view only

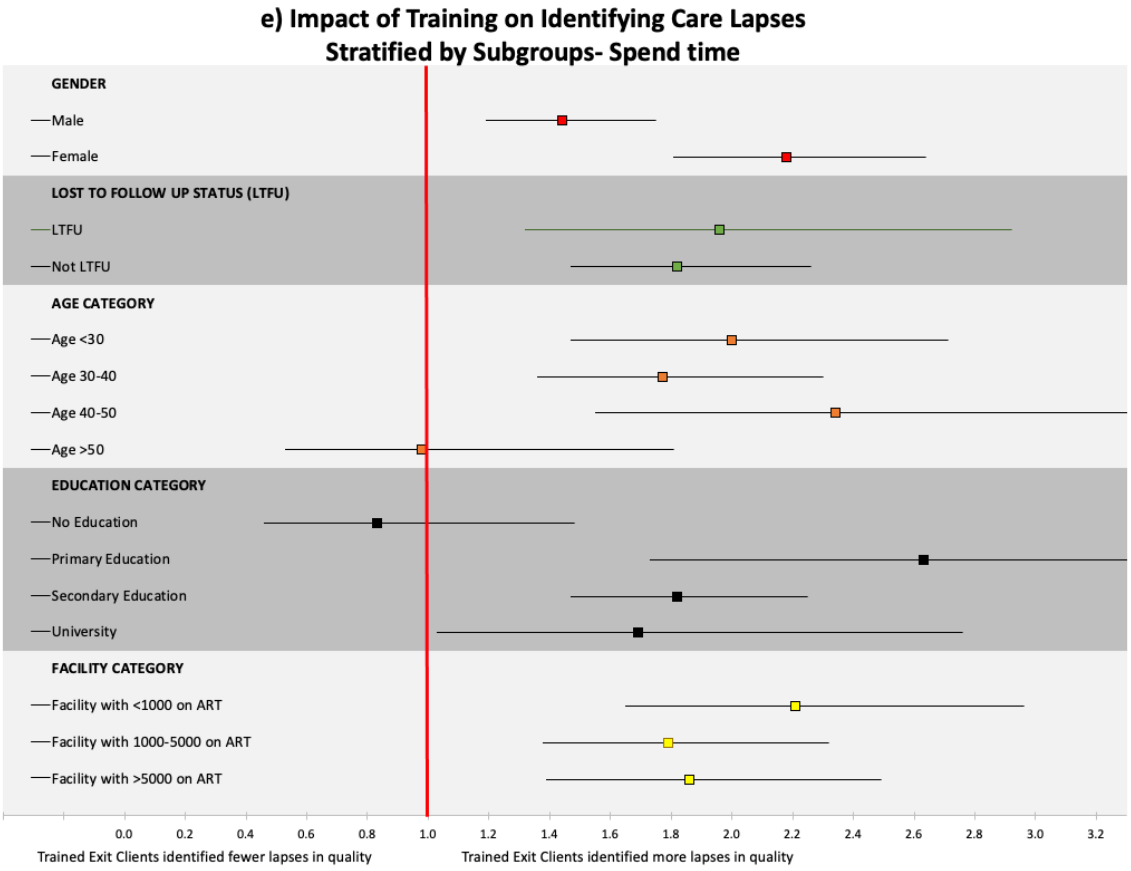
e) Impact of Training on Identifying Care Lapses
Stratified by Subgroups- Spend time
GENDER
Male
Female
LOST TO FOLLOW UP STATUS (LTFU)
LTFU
Not LTFU
AGE CATEGORY
Age <30
Age 30-40
Age 40-50
Age >50
EDUCATION CATEGORY
No Education
Primary Education
Secondary Education
University
FACILITY CATEGORY
Facility with <1000 on ART
Facility with 1000-5000 on ART
Facility with >5000 on ART
0.0 0.2 0.4 0.6 0.8 1.0 1.2 1.4 1.6 1.8 2.0 2.2 2.4 2.6 2.8 3.0 3.2
Trained Exit Clients identified fewer lapses in quality
Trained Exit Clients identified more lapses in quality

f) Impact of Training on Identifying Care Lapses
Stratified by Subgroups- Feel about care

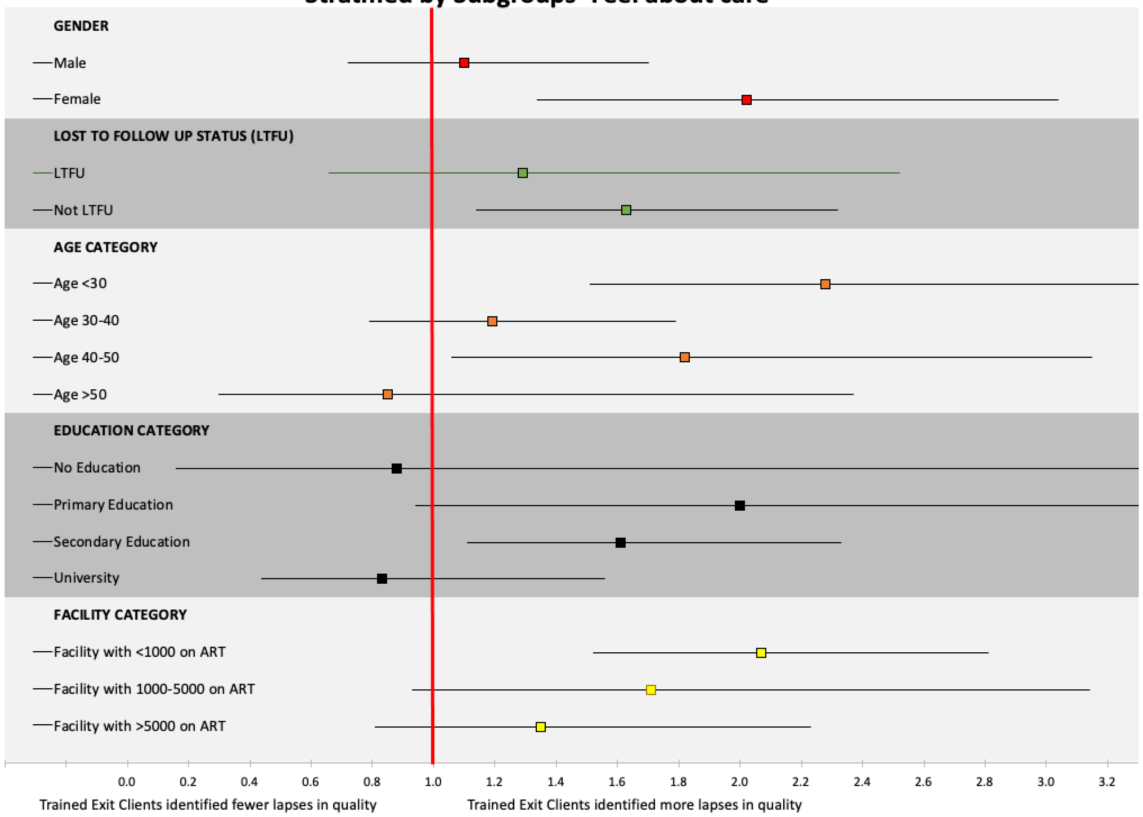

view only

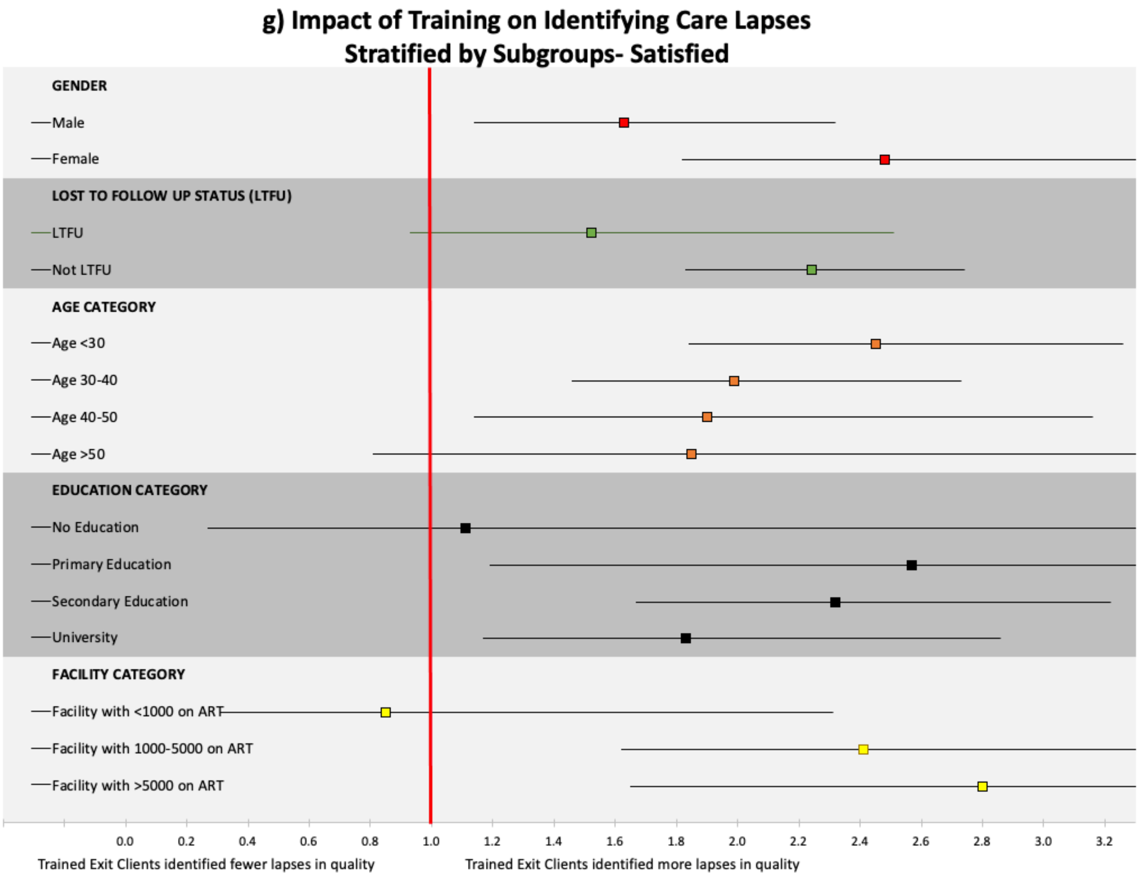
g) Impact of Training on Identifying Care Lapses
Stratified by Subgroups- Satisfied
GENDER
Male
Female
LOST TO FOLLOW UP STATUS (LTFU)
LTFU
Not LTFU
AGE CATEGORY
Age <30
Age 30-40
Age 40-50
Age >50
EDUCATION CATEGORY
No Education
Primary Education
Secondary Education
University
FACILITY CATEGORY
Facility with <1000 on ART
Facility with 1000-5000 on ART
Facility with >5000 on ART
0.0 0.2 0.4 0.6 0.8 1.0 1.2 1.4 1.6 1.8 2.0 2.2 2.4 2.6 2.8 3.0 3.2
Trained Exit Clients identified fewer lapses in quality
Trained Exit Clients identified more lapses in quality

view only

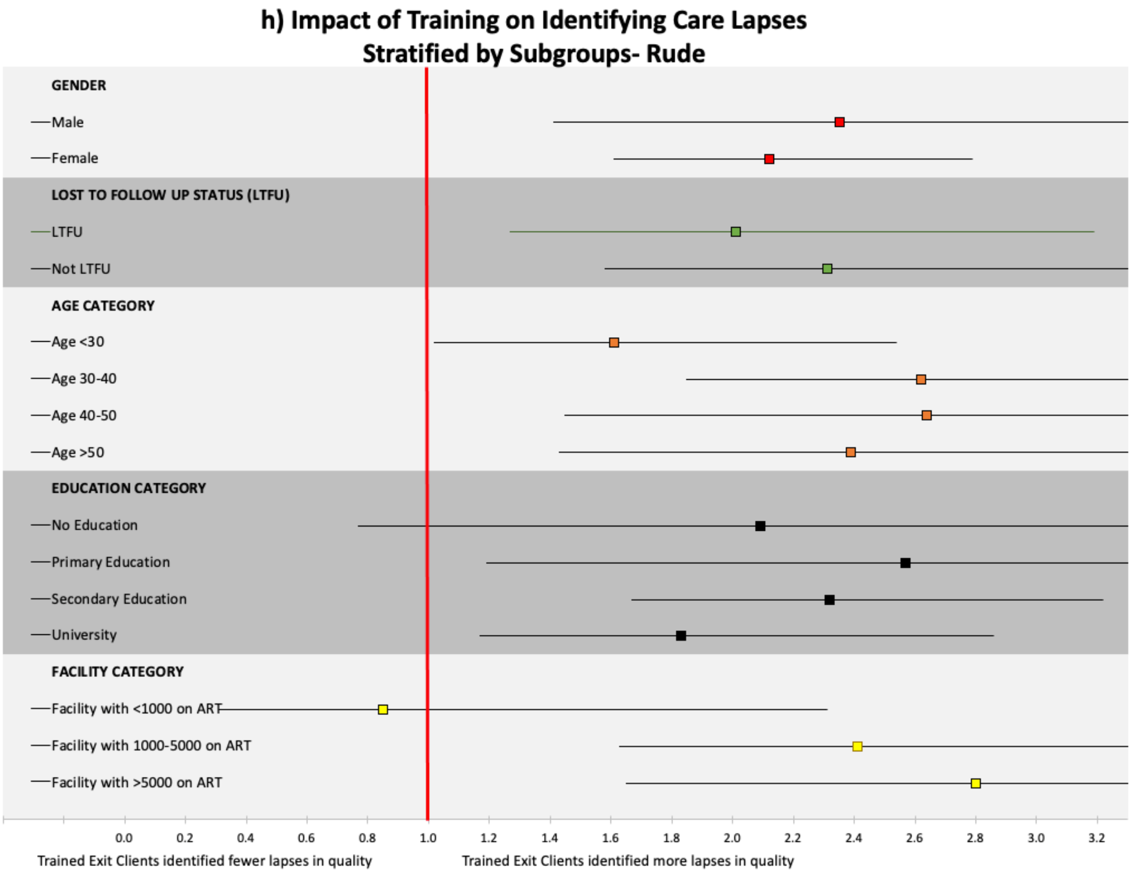
h) Impact of Training on Identifying Care Lapses
Stratified by Subgroups- Rude
GENDER
Male
Female
LOST TO FOLLOW UP STATUS (LTFU)
LTFU
Not LTFU
AGE CATEGORY
Age <30
Age 30-40
Age 40-50
Age >50
EDUCATION CATEGORY
No Education
Primary Education
Secondary Education
University
FACILITY CATEGORY
Facility with <1000 on ART
Facility with 1000-5000 on ART
Facility with >5000 on ART
0.0 0.2 0.4 0.6 0.8 1.0 1.2 1.4 1.6 1.8 2.0 2.2 2.4 2.6 2.8 3.0 3.2
Trained Exit Clients identified fewer lapses in quality
Trained Exit Clients identified more lapses in quality

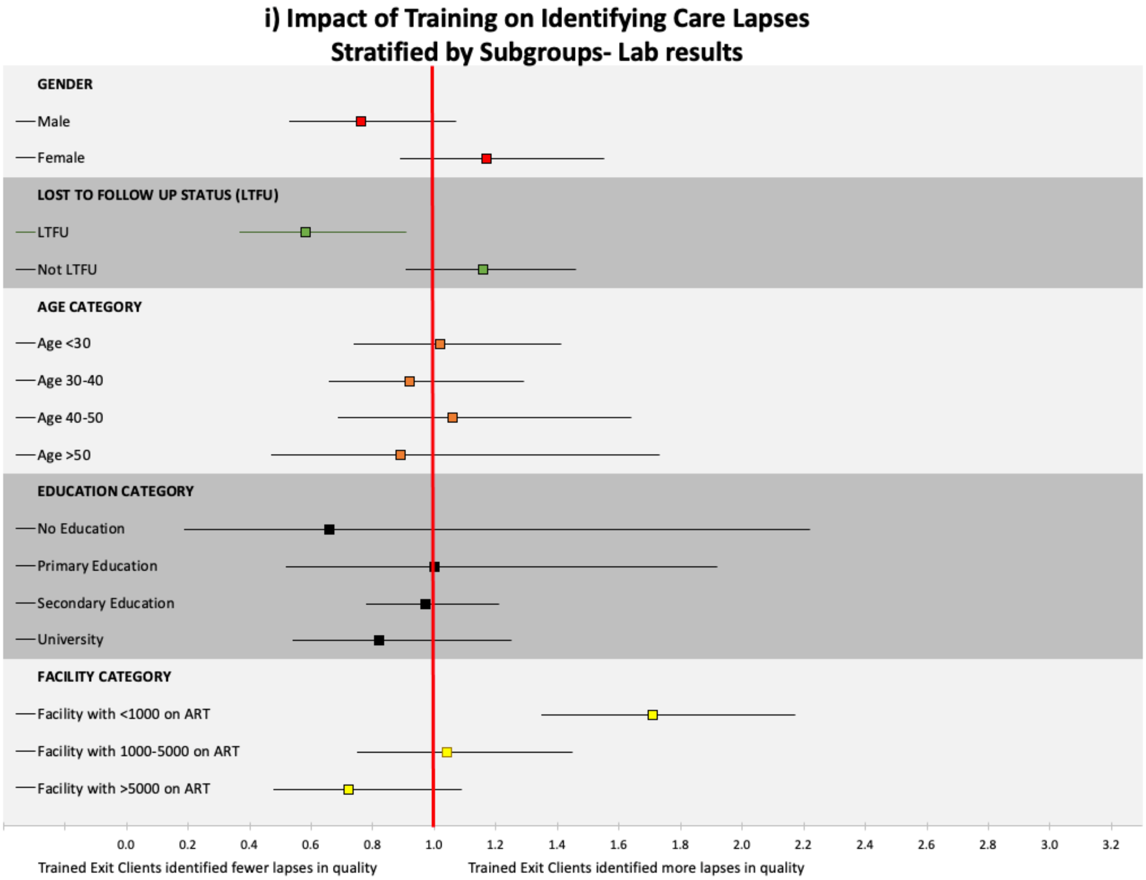
i) Impact of Training on Identifying Care Lapses
Stratified by Subgroups- Lab results
GENDER
Male
Female
LOST TO FOLLOW UP STATUS (LTFU)
LTFU
Not LTFU
AGE CATEGORY
Age <30
Age 30-40
Age 40-50
Age >50
EDUCATION CATEGORY
No Education
Primary Education
Secondary Education
University
FACILITY CATEGORY
Facility with <1000 on ART
Facility with 1000-5000 on ART
Facility with >5000 on ART
0.0 0.2 0.4 0.6 0.8 1.0 1.2 1.4 1.6 1.8 2.0 2.2 2.4 2.6 2.8 3.0 3.2
Trained Exit Clients identified fewer lapses in quality
Trained Exit Clients identified more lapses in quality

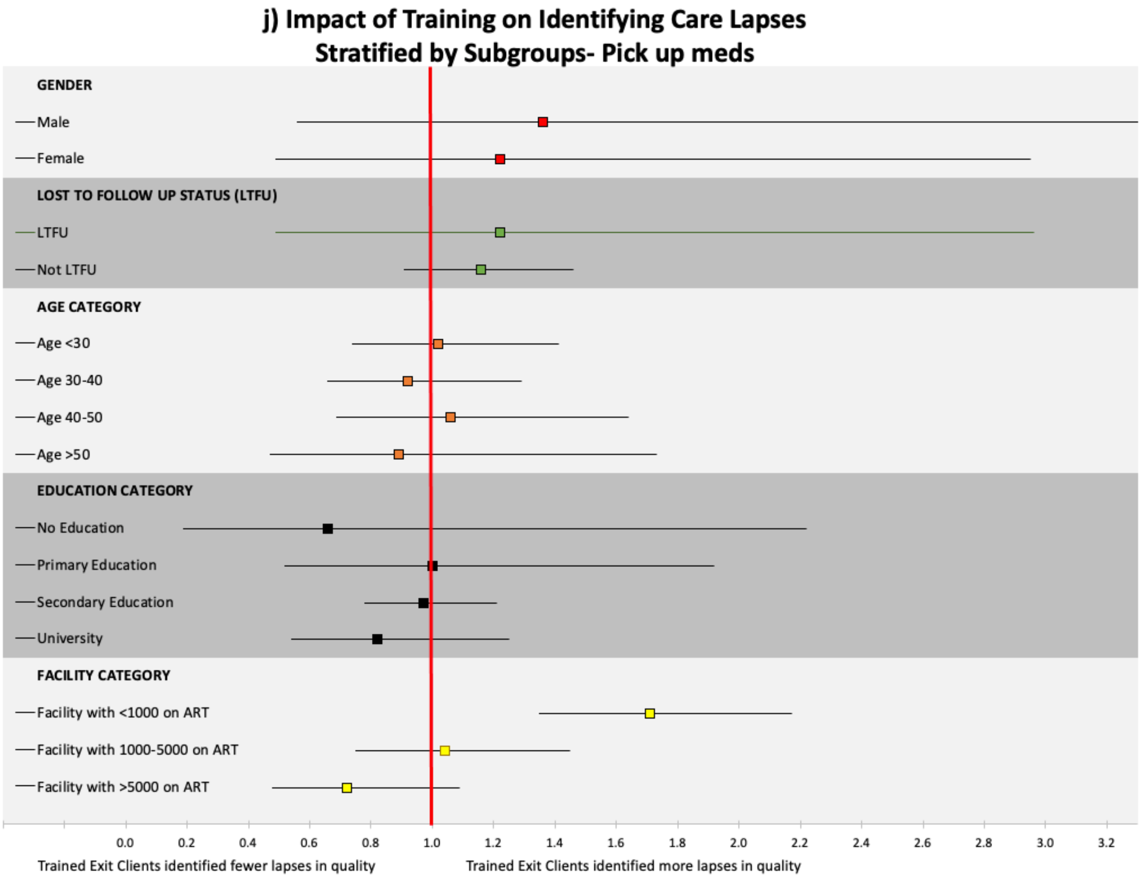
j) Impact of Training on Identifying Care Lapses
Stratified by Subgroups- Pick up meds
GENDER
Male
Female
LOST TO FOLLOW UP STATUS (LTFU)
LTFU
Not LTFU
AGE CATEGORY
Age <30
Age 30-40
Age 40-50
Age >50
EDUCATION CATEGORY
No Education
Primary Education
Secondary Education
University
FACILITY CATEGORY
Facility with <1000 on ART
Facility with 1000-5000 on ART
Facility with >5000 on ART
0.0 0.2 0.4 0.6 0.8 1.0 1.2 1.4 1.6 1.8 2.0 2.2 2.4 2.6 2.8 3.0 3.2
Trained Exit Clients identified fewer lapses in quality
Trained Exit Clients identified more lapses in quality

Supplementary Figure 2

Supplementary Figure 2. Bubble plot showing Trained Exit Sum Score vs Untrained Exit Sum Score. Each bubble represents a single facilities performance. Each bubble's size indicates the number of patients at each facility with larger bubbles corresponding to larger facilities. The horizontal position notes the Untrained Exit Sum Score for all questions against the facility, and the vertical position notes the Trained Exit sum score at the same facility.

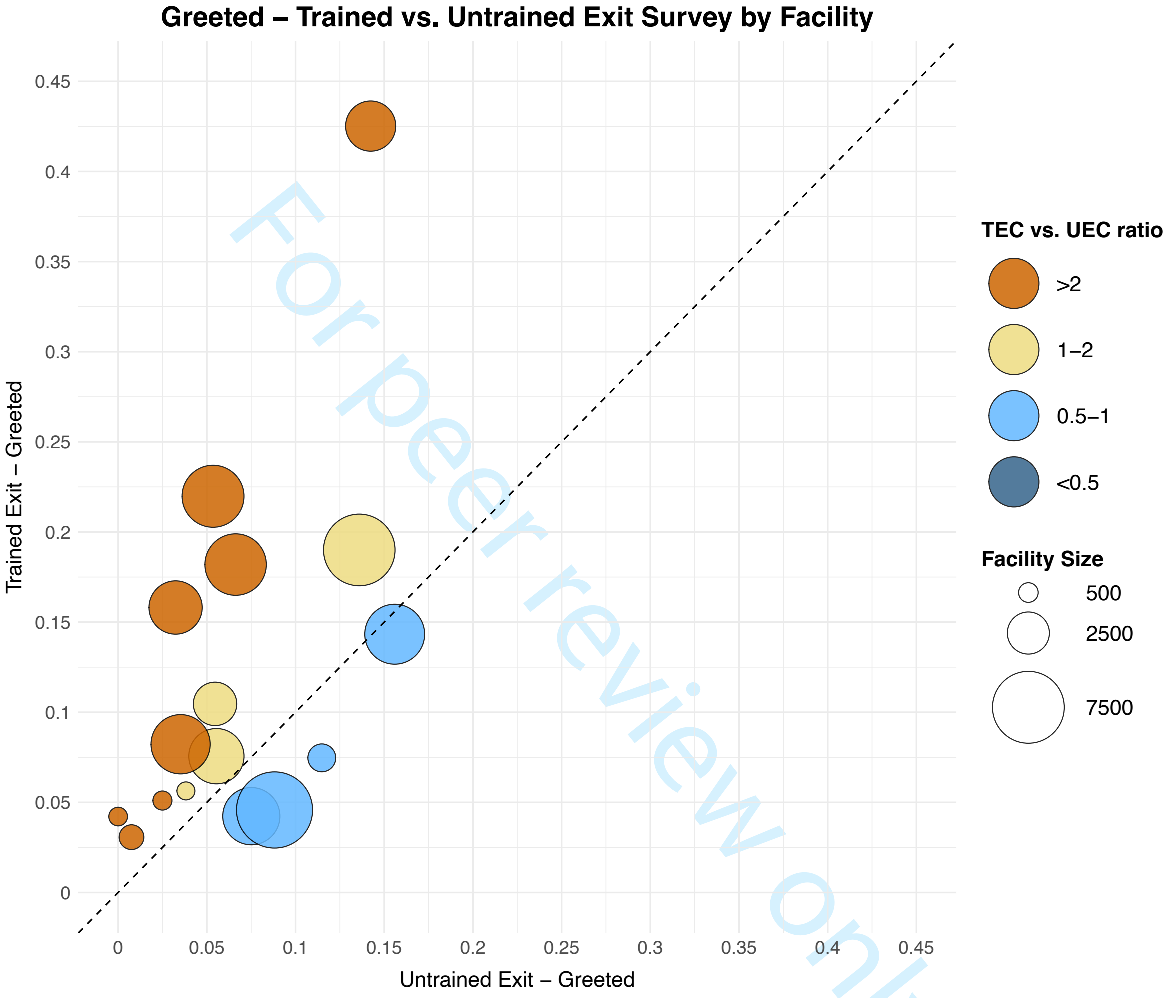

For peer review only

For peer review only - http://bmjopen.bmj.com/site/about/guidelines.xhtml

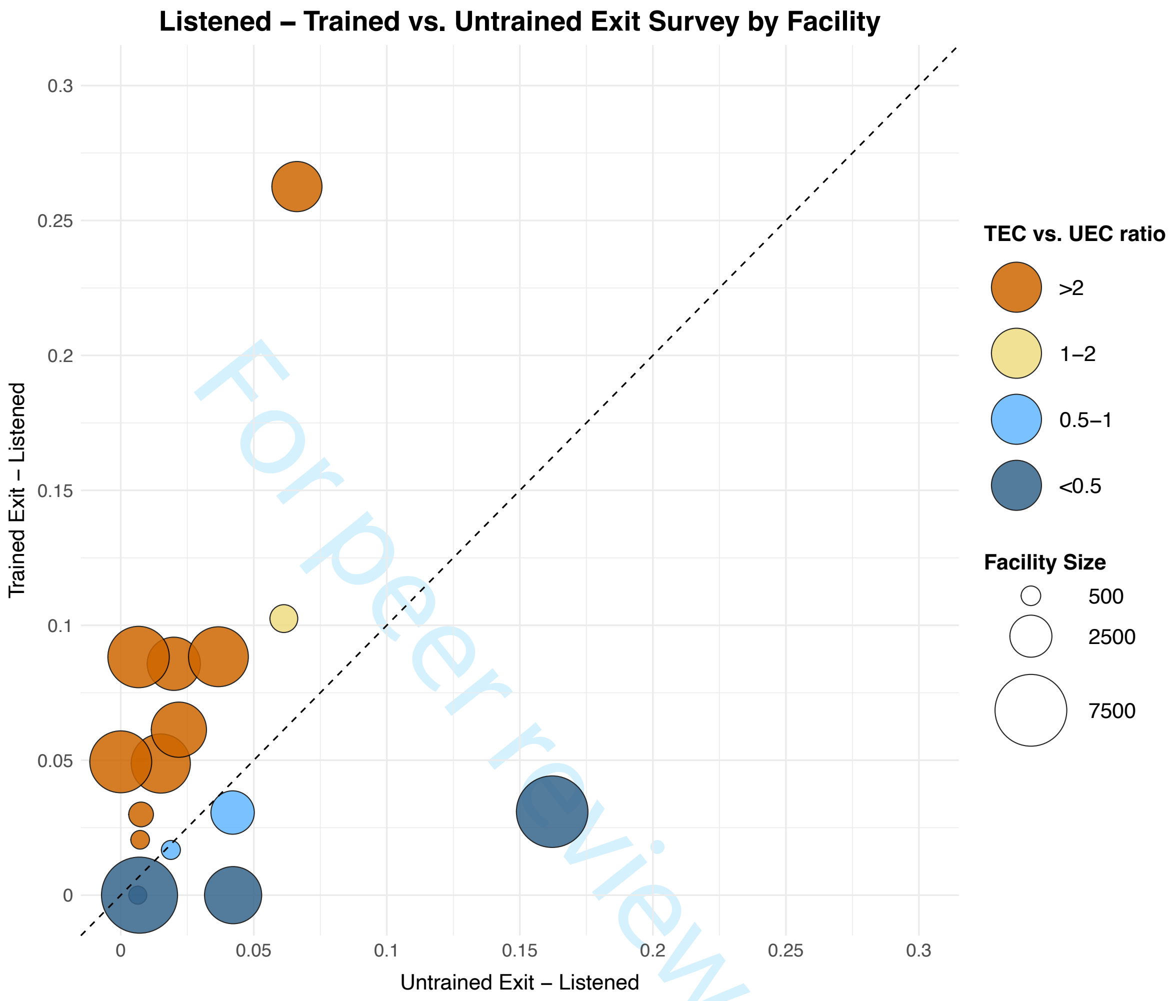

For peer review only

2

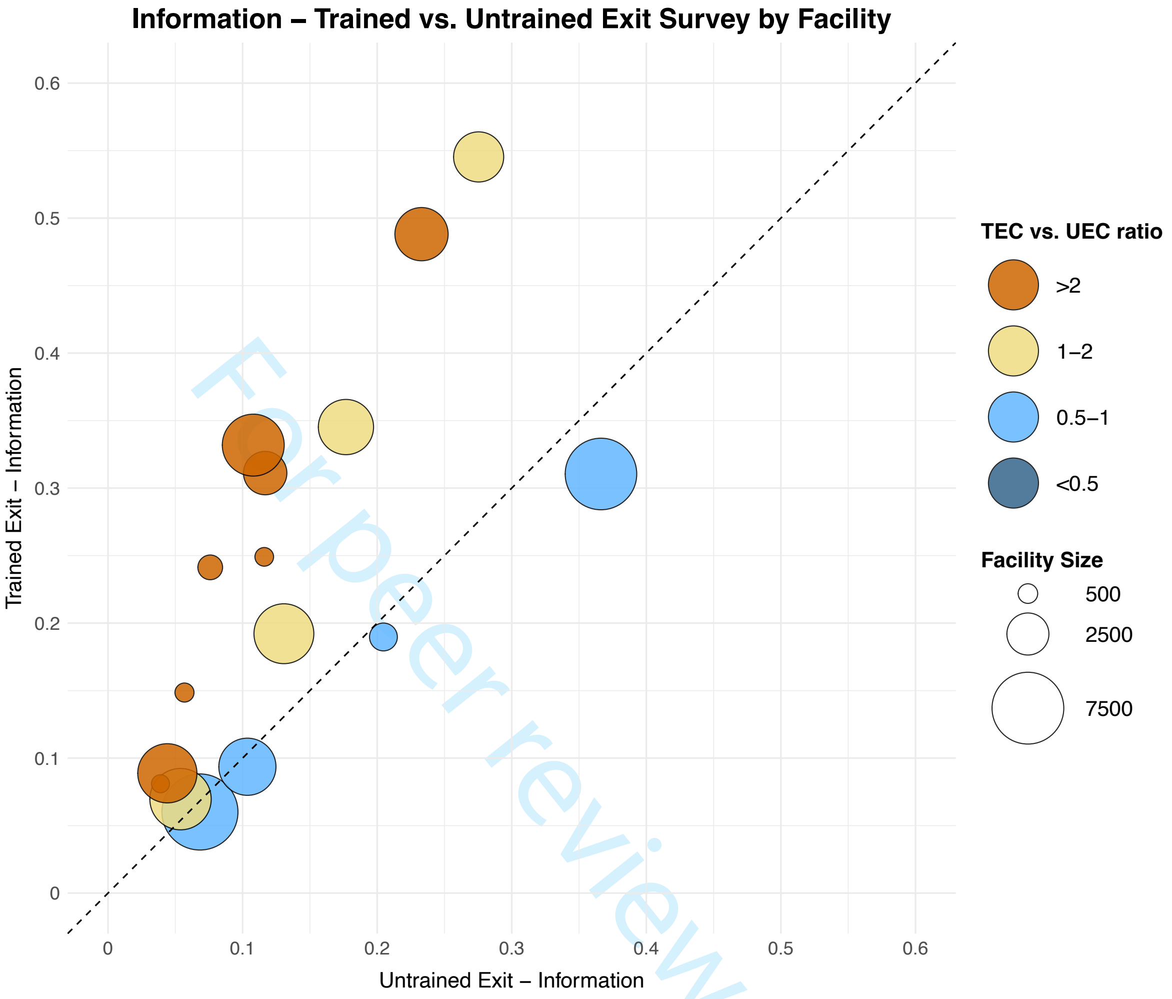

For peer review only

3

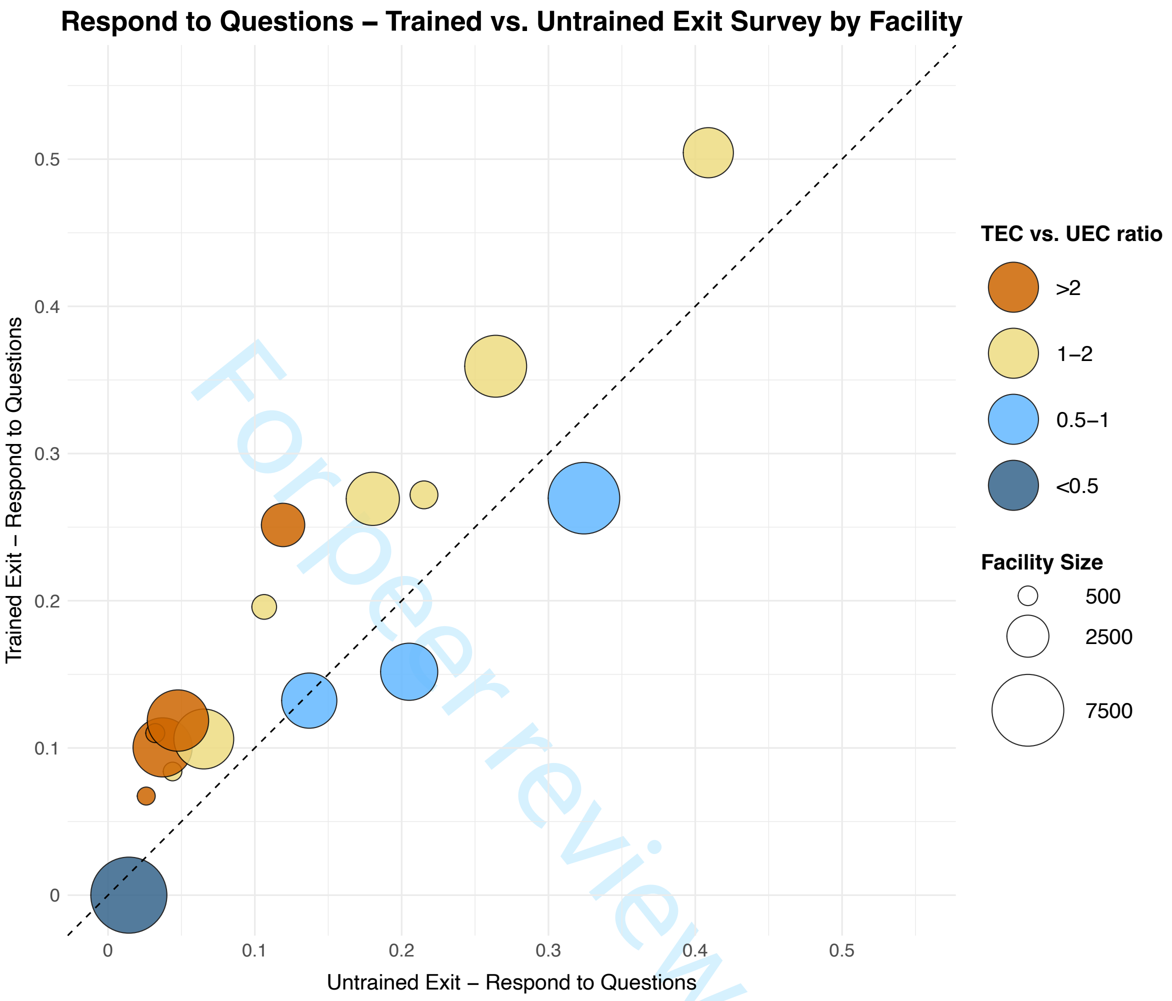

For peer review only

For peer review only - http://bmjopen.bmj.com/site/about/guidelines.xhtml

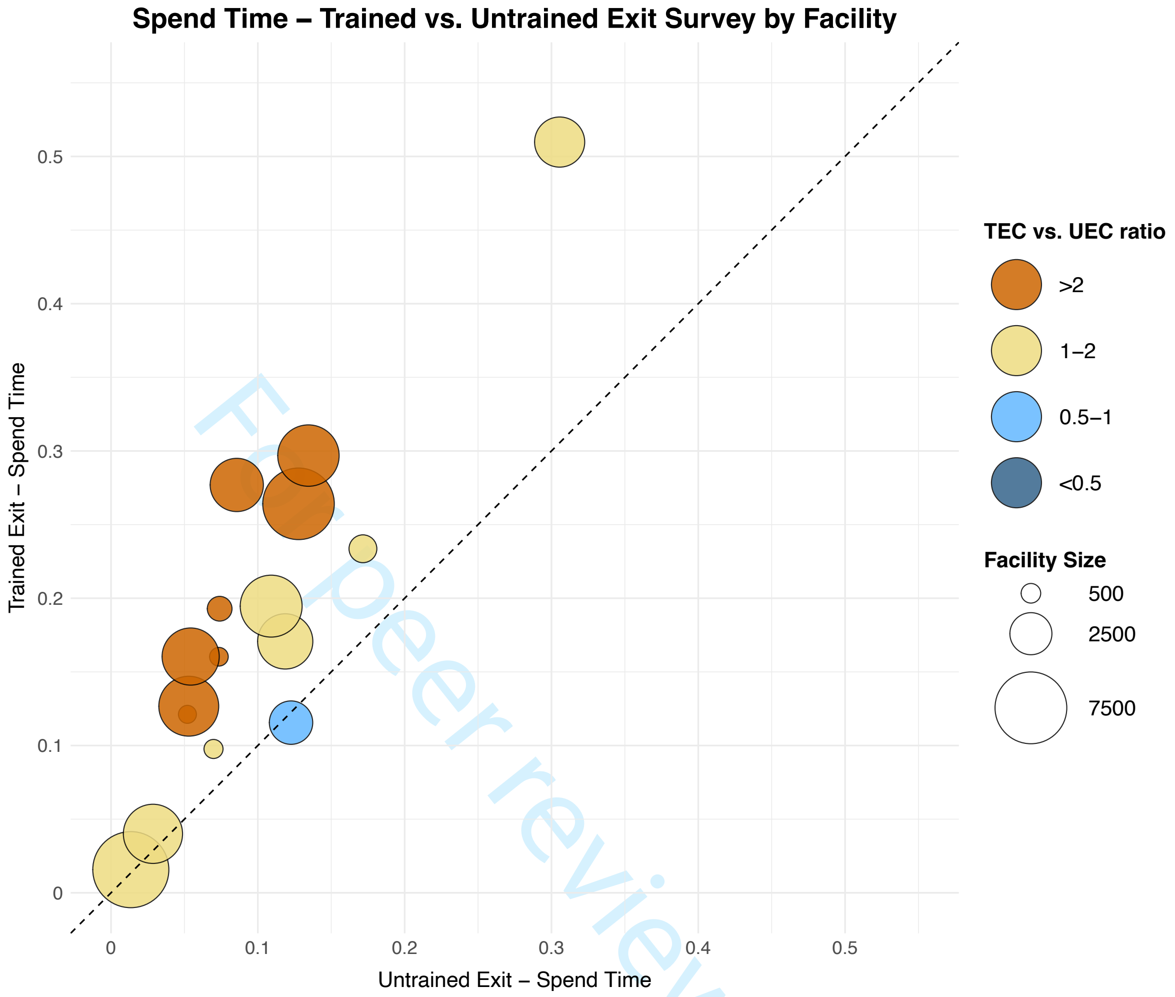

For peer review only

For peer review only - http://bmjopen.bmj.com/site/about/guidelines.xhtml

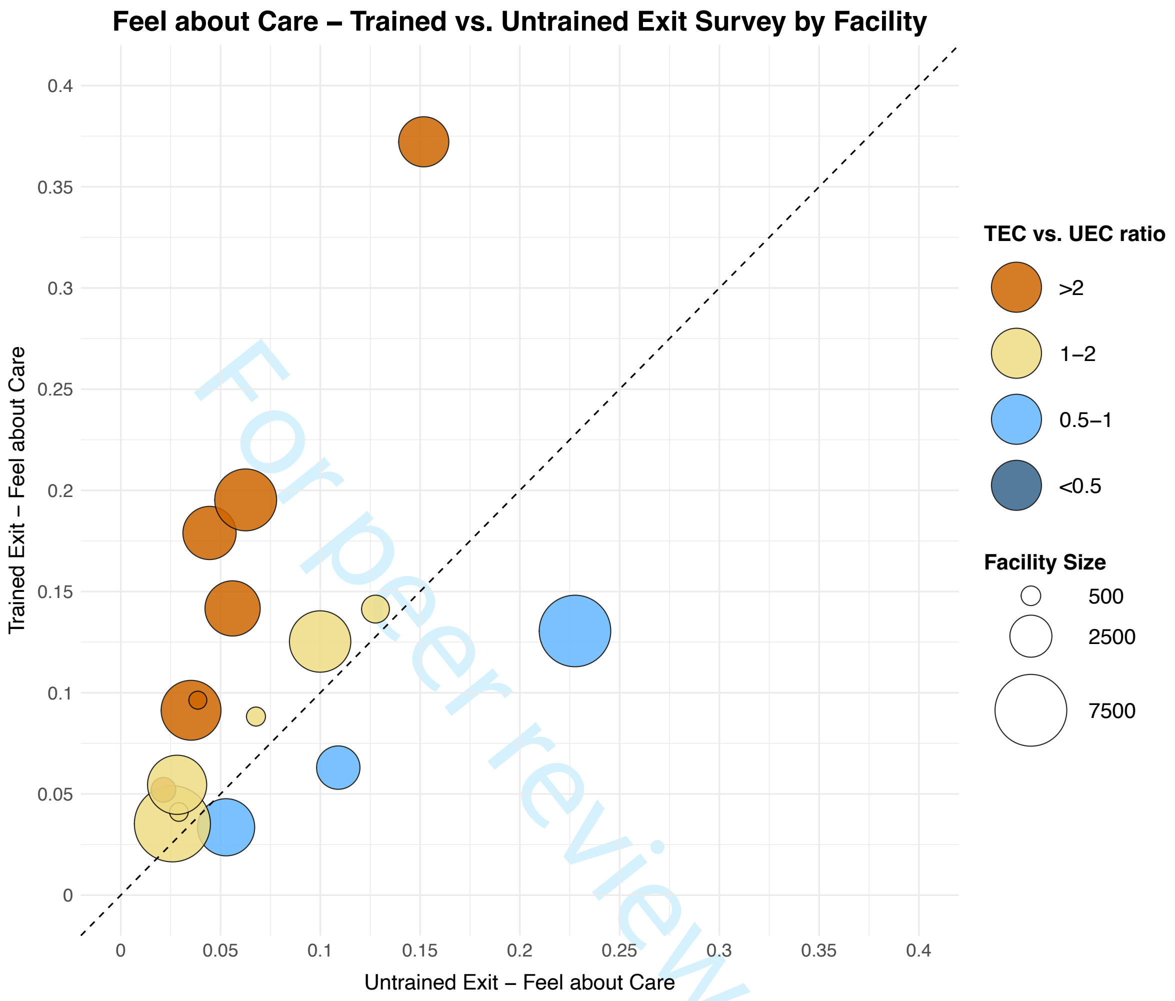

For peer review only

For peer review only - http://bmjopen.bmj.com/site/about/guidelines.xhtml

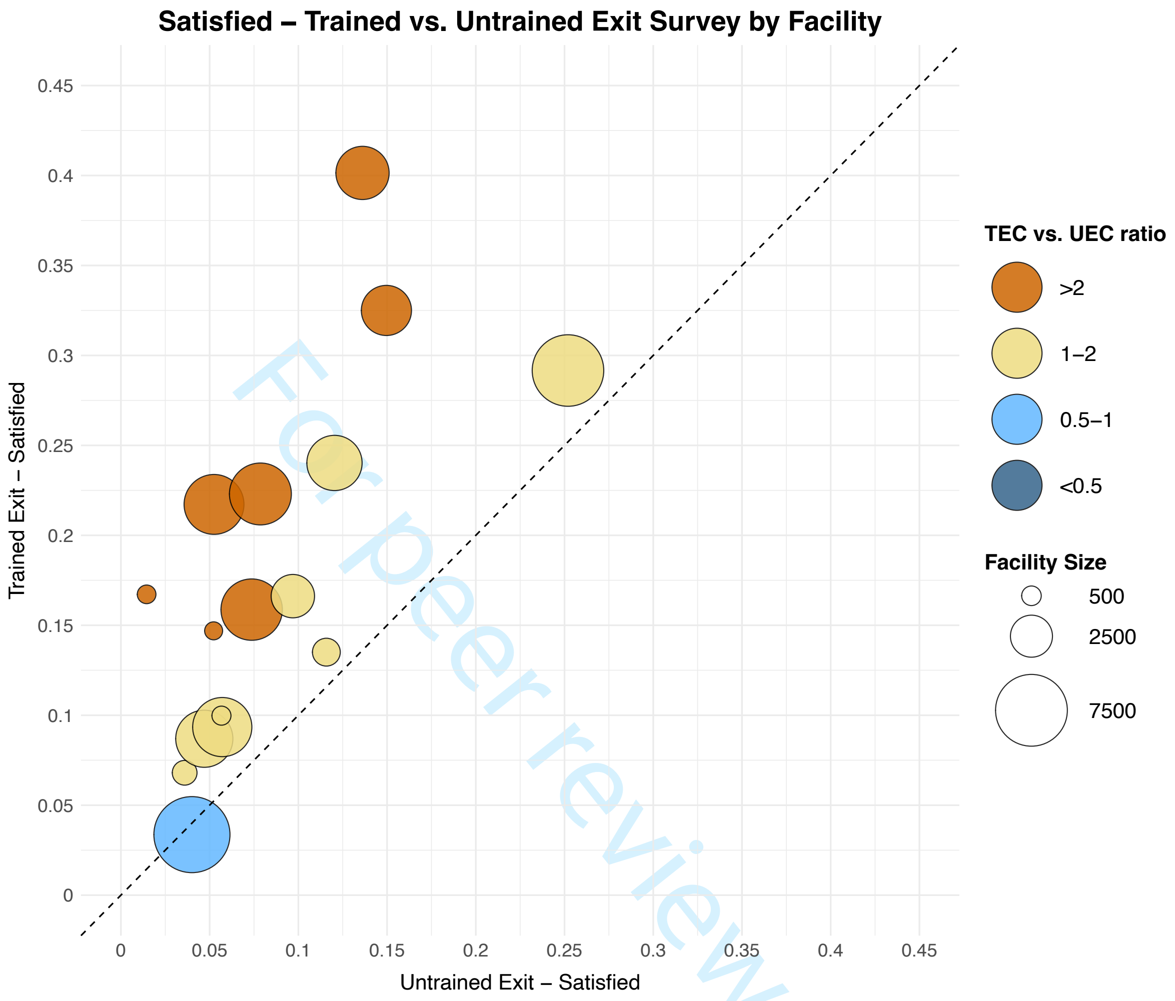

For peer review only

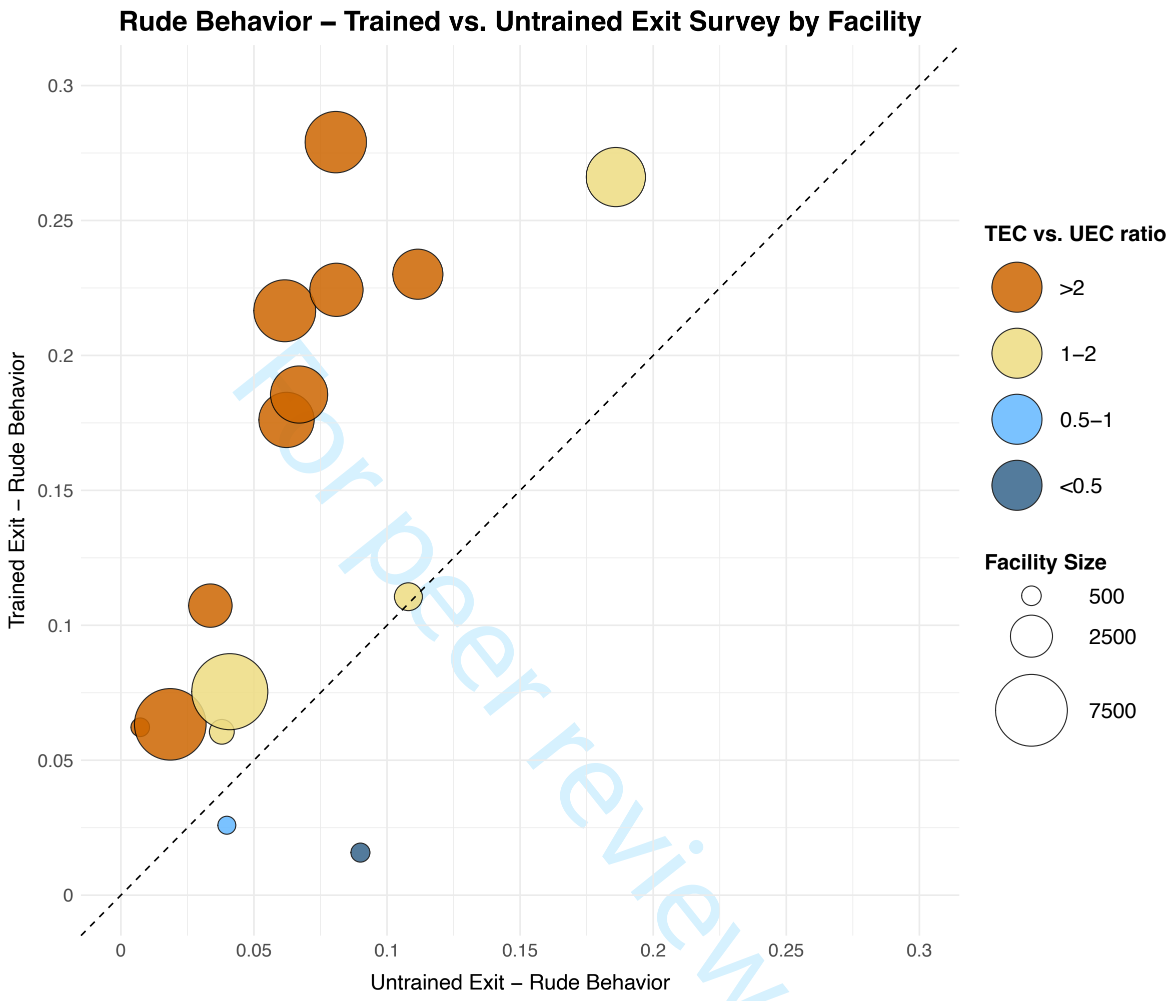

For peer review only - http://bmjopen.bmj.com/site/about/guidelines.xhtml

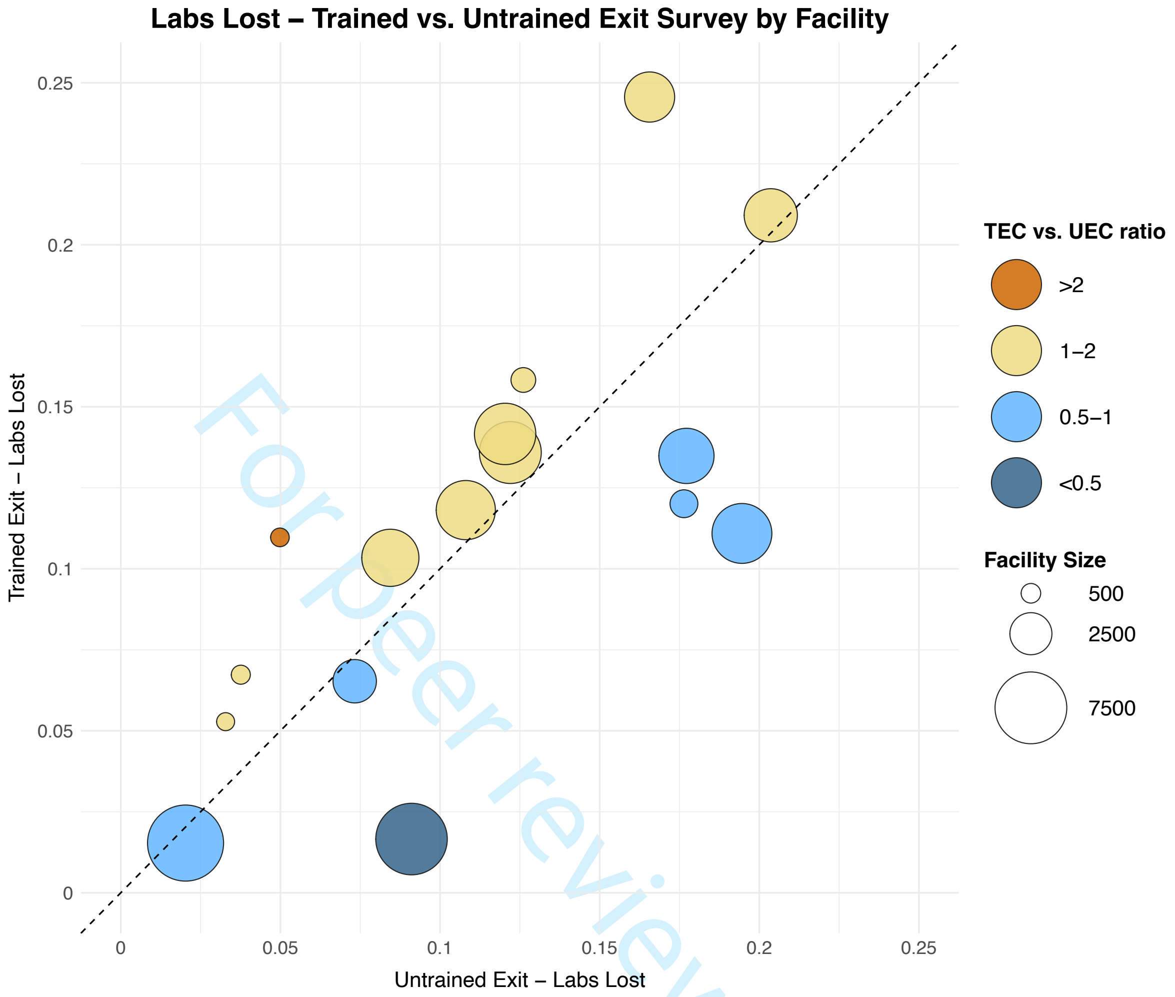

For peer review only

For peer review only - http://bmjopen.bmj.com/site/about/guidelines.xhtml

STROBE Statement—checklist of items that should be included in reports of observational studies

	Item No	Recommendation	Page No
**Title and abstract**	1	(*a*) Indicate the study's design with a commonly used term in the title or the abstract	1
		(*b*) Provide in the abstract an informative and balanced summary of what was done and what was found	1
**Introduction**			
Background/rationale	2	Explain the scientific background and rationale for the investigation being reported	3,4
Objectives	3	State specific objectives, including any prespecified hypotheses	3
**Methods**			
Study design	4	Present key elements of study design early in the paper	4,5
Setting	5	Describe the setting, locations, and relevant dates, including periods of recruitment, exposure, follow-up, and data collection	5
Participants	6	(*a*) *Cohort study*—Give the eligibility criteria, and the sources and methods of selection of participants. Describe methods of follow-up<br>*Case-control study*—Give the eligibility criteria, and the sources and methods of case ascertainment and control selection. Give the rationale for the choice of cases and controls<br>*Cross-sectional study*—Give the eligibility criteria, and the sources and methods of selection of participants	5,6
		(*b*) *Cohort study*—For matched studies, give matching criteria and number of exposed and unexposed<br>*Case-control study*—For matched studies, give matching criteria and the number of controls per case	N/A
Variables	7	Clearly define all outcomes, exposures, predictors, potential confounders, and effect modifiers. Give diagnostic criteria, if applicable	5,7,8
Data sources/ measurement	8*	For each variable of interest, give sources of data and details of methods of assessment (measurement). Describe comparability of assessment methods if there is more than one group	*5-7*
Bias	9	Describe any efforts to address potential sources of bias	4
Study size	10	Explain how the study size was arrived at	8
Quantitative variables	11	Explain how quantitative variables were handled in the analyses. If applicable, describe which groupings were chosen and why	7
Statistical methods	12	(*a*) Describe all statistical methods, including those used to control for confounding	7
		(*b*) Describe any methods used to examine subgroups and interactions	7
		(*c*) Explain how missing data were addressed	N/A
		(*d*) *Cohort study*—If applicable, explain how loss to follow-up was addressed<br>*Case-control study*—If applicable, explain how matching of cases and controls was addressed<br>*Cross-sectional study*—If applicable, describe analytical methods taking account of sampling strategy	N/A
		(*e*) Describe any sensitivity analyses	N/A

Continued on next page

For peer review only - http://bmjopen.bmj.com/site/about/guidelines.xhtml

For peer review only

**Results**			
Participants	13*	(a) Report numbers of individuals at each stage of study—eg numbers potentially eligible, examined for eligibility, confirmed eligible, included in the study, completing follow-up, and analysed	9
		(b) Give reasons for non-participation at each stage	9
		(c) Consider use of a flow diagram	N/A
Descriptive data	14*	(a) Give characteristics of study participants (eg demographic, clinical, social) and information on exposures and potential confounders	9, 10
		(b) Indicate number of participants with missing data for each variable of interest	9,10
		(c) *Cohort study*—Summarise follow-up time (eg, average and total amount)	N/A
Outcome data	15*	*Cohort study*—Report numbers of outcome events or summary measures over time	N/A
		Case-control study—Report numbers in each exposure category, or summary measures of exposure	N/A
		Cross-sectional study—Report numbers of outcome events or summary measures	10
Main results	16	(*a*) Give unadjusted estimates and, if applicable, confounder-adjusted estimates and their precision (eg, 95% confidence interval). Make clear which confounders were adjusted for and why they were included	7,11
		(*b*) Report category boundaries when continuous variables were categorized	
		(*c*) If relevant, consider translating estimates of relative risk into absolute risk for a meaningful time period	
Other analyses	17	Report other analyses done—eg analyses of subgroups and interactions, and sensitivity analyses	12,14
**Discussion**			
Key results	18	Summarise key results with reference to study objectives	14,15
Limitations	19	Discuss limitations of the study, taking into account sources of potential bias or imprecision. Discuss both direction and magnitude of any potential bias	18
Interpretation	20	Give a cautious overall interpretation of results considering objectives, limitations, multiplicity of analyses, results from similar studies, and other relevant evidence	19,20
Generalisability	21	Discuss the generalisability (external validity) of the study results	18
**Other information**			
Funding	22	Give the source of funding and the role of the funders for the present study and, if applicable, for the original study on which the present article is based	20

*Give information separately for cases and controls in case-control studies and, if applicable, for exposed and unexposed groups in cohort and cross-sectional studies.

Note: An Explanation and Elaboration article discusses each checklist item and gives methodological background and published examples of transparent reporting. The STROBE checklist is best used in conjunction with this article (freely available on the Web sites of PLoS Medicine at http://www.plosmedicine.org/, Annals of Internal Medicine at http://www.annals.org/, and Epidemiology at http://www.epidem.com/). Information on the STROBE Initiative is available at www.strobe-statement.org.

For peer review only
